# Advancing the Mechanosensitivity of Atropisomeric Diarylethene Mechanophores through a Lever-Arm Effect

**DOI:** 10.1021/jacs.4c13480

**Published:** 2025-01-10

**Authors:** Cijun Zhang, Tatiana B. Kouznetsova, Boyu Zhu, Liam Sweeney, Max Lancer, Ivan Gitsov, Stephen L. Craig, Xiaoran Hu

**Affiliations:** † Department of Chemistry, BioInspired Institute, Syracuse University, Syracuse, New York 13244, United States; ‡ Department of Chemistry, Duke University, Durham, North Carolina 27708, United States; § Department of Chemistry, The Michael M. Szwarc Polymer Research Institute, State University of New York - ESF, Syracuse, New York 13210, United States; ∥ Department of Biomedical and Chemical Engineering, BioInspired Institute, Syracuse University, Syracuse, New York 13244, United States

## Abstract

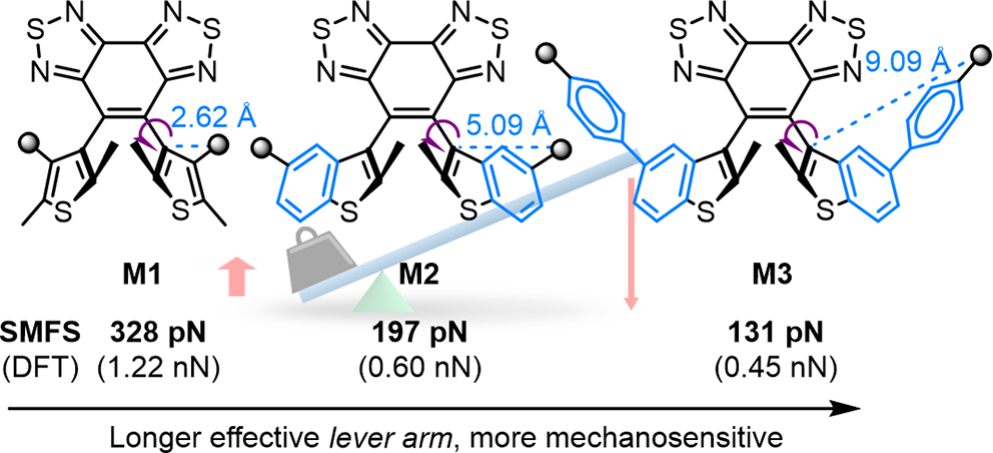

Understanding structure–mechanical activity relationships (SMARs) in polymer mechanochemistry is essential for the rational design of mechanophores with desired properties, yet SMARs in noncovalent mechanical transformations remain relatively underexplored. In this study, we designed a subset of diarylethene mechanophores based on a lever-arm hypothesis and systematically investigated their mechanical activity toward a noncovalent-yet-chemical conversion of atropisomer stereochemistry. Results from Density functional theory (DFT) calculations, single-molecule force spectroscopy (SMFS) measurements, and ultrasonication experiments collectively support the lever-arm hypothesis and confirm the exceptional sensitivity of chemo-mechanical coupling in these atropisomers. Notably, the transition force for the diarylethene **M3** featuring extended 5-phenylbenzo[*b*]thiophene aryl groups is determined to be 131 pN ± 4 pN by SMFS. This value is lower than those typically recorded for other mechanically induced chemical processes, highlighting its exceptional sensitivity to low-magnitude forces. This work contributes a fundamental understanding of chemo-mechanical coupling in atropisomeric configurational mechanophores and paves the way for designing highly sensitive mechanochemical processes that could facilitate the study of nanoscale mechanical behaviors across scientific disciplines.

## Introduction

**Figure 1:**
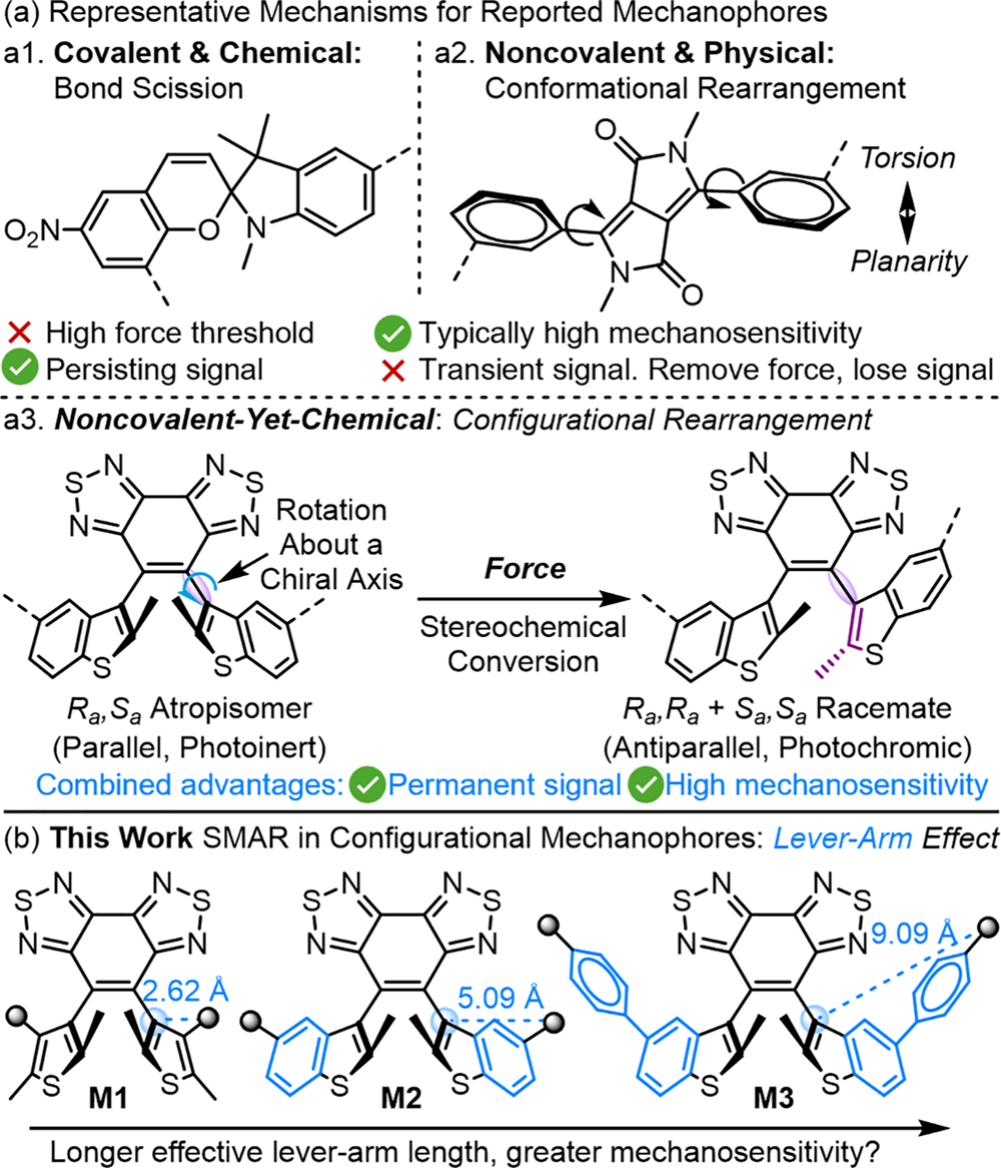
(a) Representative mechanisms for mechanically induced molecular transformations. (b) This study introduces a lever-arm effect that enables fine-tuning mechanical reactivity in force-triggered atropisomerization.

Mechanophores are molecular units that undergo specific structure and property changes under mechanical force,^1−3^ allowing for molecular-level insights into mechanical behaviors. This “mechanophore” concept traditionally refers to mechanically induced *chemical* transformations, such as the cleavage of azo moieties^4^ or ring-opening of spiropyrans (Figure 1a).^5−7^ Those covalent mechanochemical processes typically involve homolytic cleavage,^8−13^ heterolytic cleavage,^14,15^ pericyclic reactions,^16−24^ or metal–coordinate bond cleavage.^25−27^ Kauzmann and Eyring extended the transition state theory to cases where a constant external force acts along the reaction pathway,^28,29^ shedding light on the fundamentals of covalent mechanochemistry. It is understood that the application of force decreases the activation energy (*E*_act_) of a reaction by coupling mechanical work to the nuclear movements that occur along the reaction coordinate.^29−32^ Bell’s approximate theory^33^ further states that an applied force *F* changes a reaction’s energy barrier by Δ*E*_act_ = −*F*ΔR, where ΔR is the change in the distance between the atoms (to which *F* is applied) from the reactant state to the transition state along the reaction coordinate. This approximate theory assumes the force only linearly reduces the activation energy, without otherwise distorting structures or altering reaction pathways. More comprehensive theoretical treatments are now well established,^34−38^ but the Kauzmann/Eyring/Bell framework remains a useful construction for qualitatively interpreting many mechanochemical structure–activity effects.

The “mechanophore” concept has expanded to include units that respond to mechanical forces through *noncovalent*, *physical* means such as conformational rearrangements (Figure 1a).^39,40^ These noncovalent transformations typically respond to lower-magnitude forces than those required to break covalent bonds (0.24 to several nN on the time scale of 0.1 s, as characterized by single-molecule force spectroscopy).^22,23,41−48^ Thus, the exploration of noncovalent mechanical transformations has attracted increasing interest. For example, Weder and Sagara have introduced rotaxane- and cyclophane-based mechanophores where force affects the spatial alignment between chromophores and alters their photoluminescent properties.^49−52^ Saito and co-workers have pioneered “flapping” mechanophores that undergo conformational planarization under mechanical stimulation that extends the conjugation length.^53,54^ Moreover, the research groups of Matile,^55−58^ Sommer (Figure 1a),^59^ and Lu^60^ have innovated twisted conjugated systems that planarize and gain conjugation efficiency under force. Other strategies for force-induced noncovalent processes include mechanical manipulation of supramolecular interactions in synthetic and biomaterials,^61−63^ and metal–ligand dissociation.^64^ However, most noncovalent physical changes provide transient signals that disappear when the force is removed.

The development of mechanophores that are both highly mechanosensitive and capable of permanently recording mechanical activation events could enhance the study of nanoscale mechanical processes. Recently, our group has introduced a diarylethene configurational mechanophore (Figure 1a) that undergoes a *noncovalent-yet-chemical* conversion of atropisomer stereochemistry upon mechanical stimulation, transitioning from a parallel form to its antiparallel diastereomers.^65,66^ This stereochemical conversion permanently alters molecular symmetry and turns on chemical reactivity toward a subsequent photochemical electrocyclization reaction. Density functional theory (DFT) calculations using the constrained geometries simulate external force (CoGEF) method and estimate its peak force *F*_max_ at 0.6 nN, significantly lower than is typical of mechanically induced covalent chemical reactions that have been evaluated using CoGEF.^67−69^ This mechanophore also showed faster activation rates in solution-phase ultrasonication experiments compared to a benchmark anthracene-maleimide mechanophore. These initial findings underscore the force-stereochemistry coupling as a promising mechanism for developing high-sensitivity mechanochemical transformations, but a quantitative measure of the transition force required to drive the stereochemical conversion at a given rate has yet to be determined.

Understanding structure–mechanical activity relationships (SMARs) in polymer mechanochemistry is essential for the rational design of mechanophores with desired properties. To date, SMAR studies have predominantly focused on covalent mechanochemistry. For example, the stereochemistry, regiochemistry, and substituent effects in various mechanophore scaffolds, such as benzocyclobutane,^20,70^ cyclobutene,^71,72^ gem-dihalocyclopropanes,^44^ naphthopyran,^73−75^ furan-maleimide,^76,77^ spiropyran,^6,7,46^ and the more recent pterodactylane mechanophores,^78^ have been shown to significantly influence their chemo-mechanical coupling. One key mechanism for enhancing mechanochemical reactivity is a “lever-arm” effect, where variations in the polymer backbone structure and/or the structure of the handles connecting mechanophore and polymer act like a molecular crowbar that can modulate mechanophore activity by changing the ΔR parameter associated with Bell theory.^42,72,79−82^ However, SMARs in noncovalent mechanical transformations remain underexplored in polymer mechanochemistry.^83^

**Figure 2:**
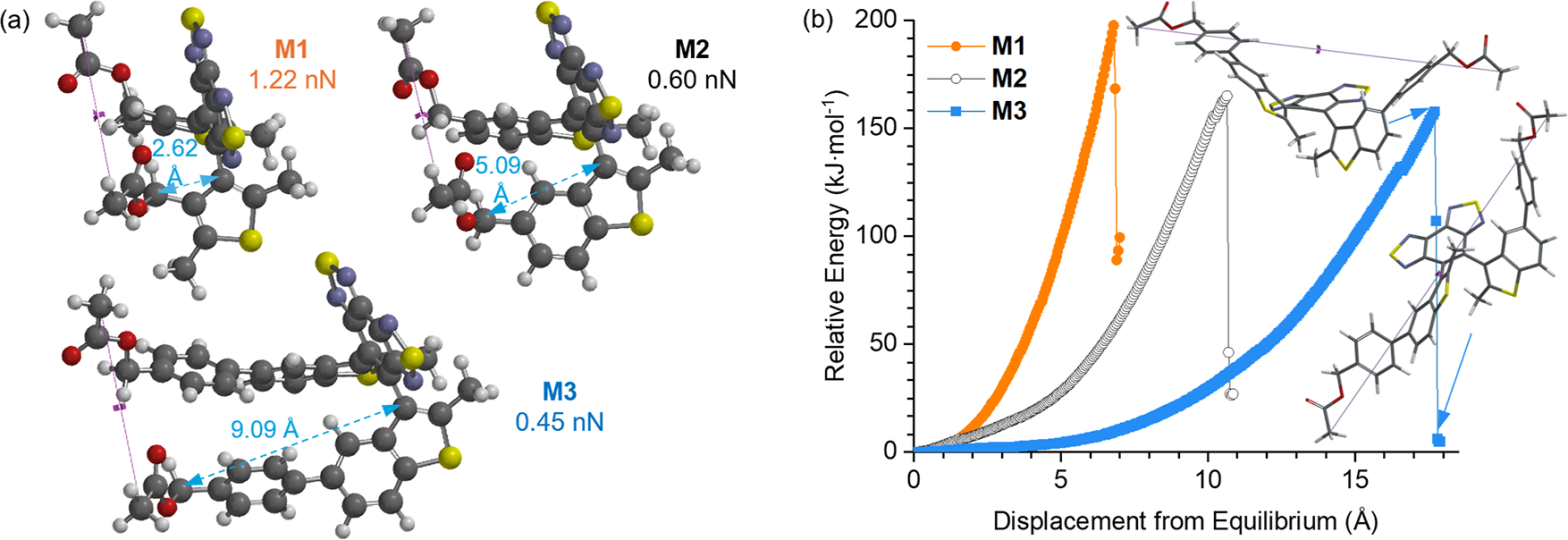
(a) DFT-calculated structures of model mechanophores in equilibrium geometry. (b) CoGEF calculations (B3LYP/6-31G*) predict the mechanical stereochemical conversion of parallel diarylethenes to their antiparallel forms. Elongating the constrained distance results in distortion of the dihedral angle between the benzothiadiazole bridge and the side-arm aryl groups and eventually leads to a sudden rotation of one side-arm aryl plane around the BBT-aryl σ bond, inducing a flip at that chirality axis. **M3** structures corresponding to the data point indicated by the arrow are shown (see the SI for details).

We are particularly interested in developing a fundamental understanding of the chemo-mechanical coupling in the recently introduced configurational mechanophores.^65,66^ In this study, we quantified the transition forces (F*) for mechanical atropisomerization using single-molecule force spectroscopy (SMFS) for the first time to characterize mechanophores **M1-M3** (Figure 1b). The previously reported **M2** structure showed an F* of 197 pN ± 12 pN, corroborating its high mechanical activity suggested by earlier indirect evidence.^65^ Using Bell’s approximation as an intuitive framework,^33^ we hypothesized a “lever-arm effect” to fine-tune the mechanochemical reactivity in diarylethene atropisomers. For a subset of mechanophores **M1**-**M3** (Figures 1b and 2a) which undergo mechanistically similar transformation of atropisomer stereochemistry, **M3** features the longest rigid structure—the “lever arm”—between the polymer attachment site (where force is applied) and the rotational chiral axis (the “fulcrum”), maximizing the ΔR and thus, requiring the smallest force to adequately reduce the activation barrier for atropisomerization. Conversely, **M1** comprises the shortest “lever arm” and requires the highest force. This anticipated activity trend is confirmed by DFT calculations, SMFS, and ultrasonication experiments. Remarkably, for **M3**, which incorporates the longest lever arm, its F* is further reduced to 131 pN ± 4 pN, a 33% reduction from the record of previously reported **M2** structure. This study offers fundamental insights into the chemo-mechanical coupling between atropisomer stereochemistry and force and provides design principles for highly sensitive mechanochemical transformations which could enable the study of previously unobserved nanoscale mechanical processes.

## DFT Calculations

Configurational mechanophores **M1**-**M3** are designed to comprise the same sterically bulky benzobis(thiadiazole) (BBT) bridge, while their side-arm aryl groups are rationally varied to adjust the effective length of the rigid “lever-arm” structures between the polymer anchoring site and the rotational chiral axis. The distances between the polymer attachment site and the rotational chiral axis for **M1**, **M2**, and **M3** in their DFT-predicted equilibrium geometry (indicated by blue arrows in Figure 2a) are measured to be 2.62, 5.09, and 9.09 Å, respectively. Their truncated models are subjected to DFT calculations using the CoGEF technique to simulate the force-induced atropisomerization.^84^ The distance between two terminal atoms (purple lines in Figure 2a), where force is applied, is fixed. Starting from the equilibrium geometry, this distance is incrementally increased, with the molecule’s energy minimized after each step. For all three model mechanophores, elongating the constrained distance results in distortion of the dihedral angle between the benzothiadiazole bridge and the side-arm aryl groups, along with bond elongation along the force transduction axis. Eventually, this leads to a sudden rotation of one side-arm aryl plane around the BBT-aryl σ bond, inducing a flip at that chirality axis. As a result, the parent achiral *S*_a_*,R*_a_ parallel diarylethenes are transformed into their antiparallel diastereomers. The force-driven rotation for DFT models of **M1**, **M2**, and **M3** exhibits peak CoGEF *F*_max_ values of 1.22 nN, 0.60 nN, and 0.45 nN, respectively. These results align with the lever-arm hypothesis stating that an increase in the effective length of the lever arm enhances mechanical activity. **M3** exhibits the lowest CoGEF-estimated *F*_max_ value, a further decrease of 25% from **M2** in our earlier study,^65,66^ whose *F*_max_ value was already lower than that estimated by CoGEF for other mechanically induced chemical reactions to the best of our knowledge.^67^ As a static quantum method, CoGEF neglects the thermal effects and tends to overestimate the peak force *F*_max_ compared to the transition force measured from SMFS experiments (*vide infra*),^85^ but previous studies^67^ have validated CoGEF as a useful framework to compare the relative activity of mechanophores.

## Results and Discussion

**Figure 3:**
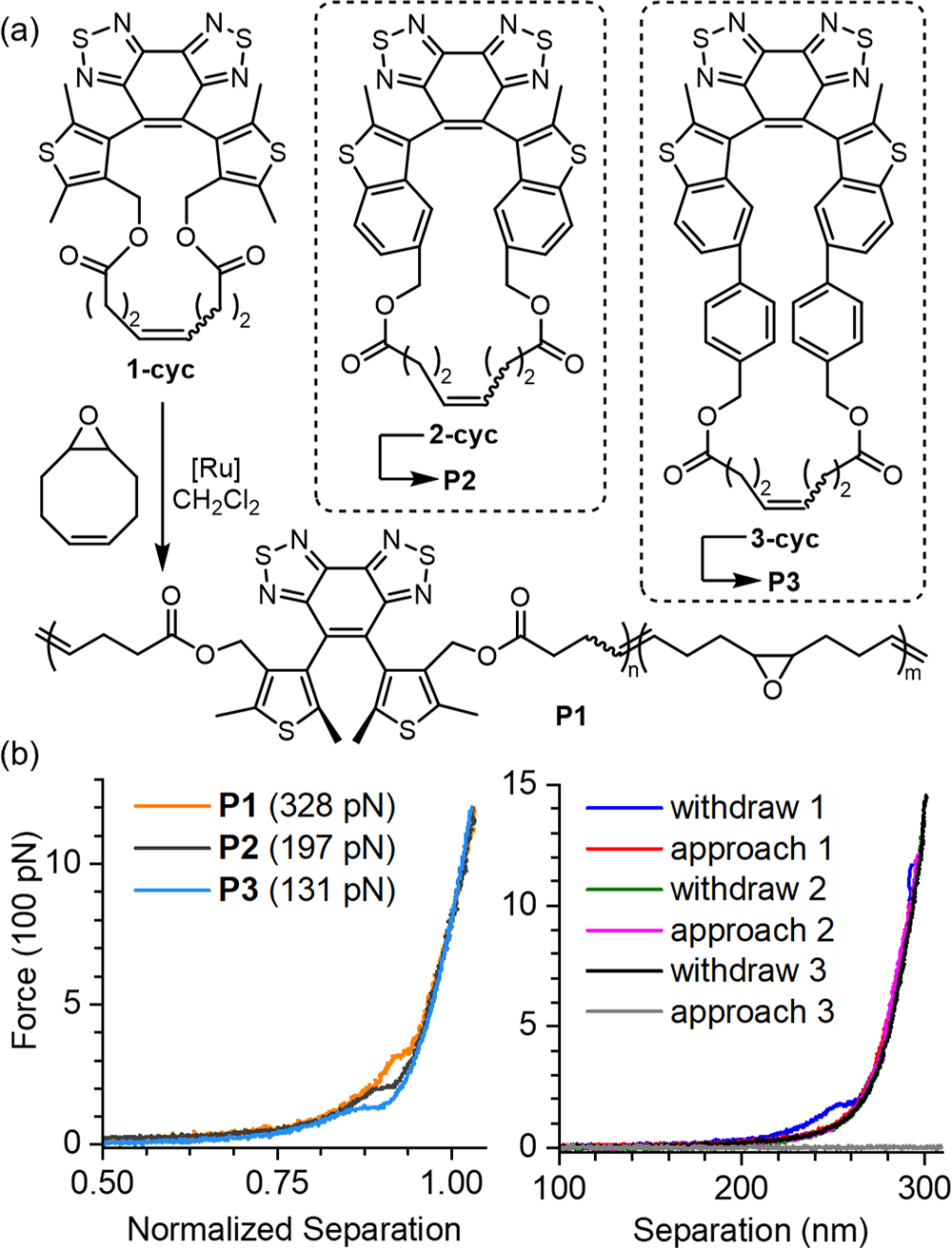
(a) Synthetic scheme for multimechanophore copolymers **P1**-**P3**. (b) Overlay of representative force–extension curves obtained for **P1**-**P3**. Curves are normalized to the corresponding extension at 0.8 nN force. (c) Multicycle SMFS experiment of **P2** shows a characteristic plateau in the first withdraw, corresponding to the stereochemical conversion from parallel diarylethenes to the antiparallel. No plateau is observed in subsequent cycles.

We synthesized macrocyclic mechanophores and their copolymers **P1**-**P3** for SMFS studies (Figures 3a and Supporting Information). The copolymerization of mechanophores with cyclooctene epoxide units is a common strategy to increase the adhesion of the copolymer to the tip of atomic force microscopy. The reactivity of macrocyclic mechanophore monomers toward ring-opening metathesis polymerization (ROMP) is low, presumably because of the sterically bulky diarylethene structures in the macrocycles.^86^ Using optimized conditions, we prepared **P1**-**P3** with molecular weights around 50 kg/mol comprising about 5 mol % of mechanophore units. Multimechanophore ROMP copolymers **P1**-**P3** were deposited onto a surface by evaporation of a dilute polymer solution in THF. Approach/withdraw cycles of the AFM tip at a velocity of 300 nm·s^–1^ resulted in force–extension curves that display characteristic transitions corresponding to the mechanical conversion of atropisomer stereochemistry in the mechanophore units. Remarkably, **P1**-**P3** displayed distinct plateaus at 328 pN ± 10 pN, 197 pN ± 12 pN, and 131 pN ± 4 pN, respectively, in their force–extension curves, aligning with our “lever-arm” hypothesis and CoGEF calculations (Figure 3b, left). The same macromolecule chains subjected to multiple cycles of tip retraction only exhibit the characteristic plateau in the first cycle, while curves from subsequent cycles lack this characteristic plateau and they essentially overlap (Figure 3b, right). These multicycle SMFS results evidence that the stereochemical conversion of mechanophore units in the copolymers was irreversibly completed in the first cycle without bond scission. Putting the SMFS results into context, the F* required for the most mechanosensitive covalent mechanophores known to date like spiropyran is around 240 pN, as determined by SMFS.^6,35,48,67^ Comparable magnitude of forces determined by single-molecule measurements has been reported in mechanobiology systems, such as the unzipping of hybridized dsDNA (about 300 pN),^87^ unfolding of individual immunoglobulin domains (about 150–300 pN),^88^ and disruption of antibody–antigen interactions (about 150 pN).^89^ The ability of configurational mechanophores to irreversibly respond to low-magnitude forces uniquely positions them as a potent technology for permanently recording mechanical activation history and enabling the study of previously unobservable mechanical behaviors in synthetic and biological materials.

**Figure 4:**
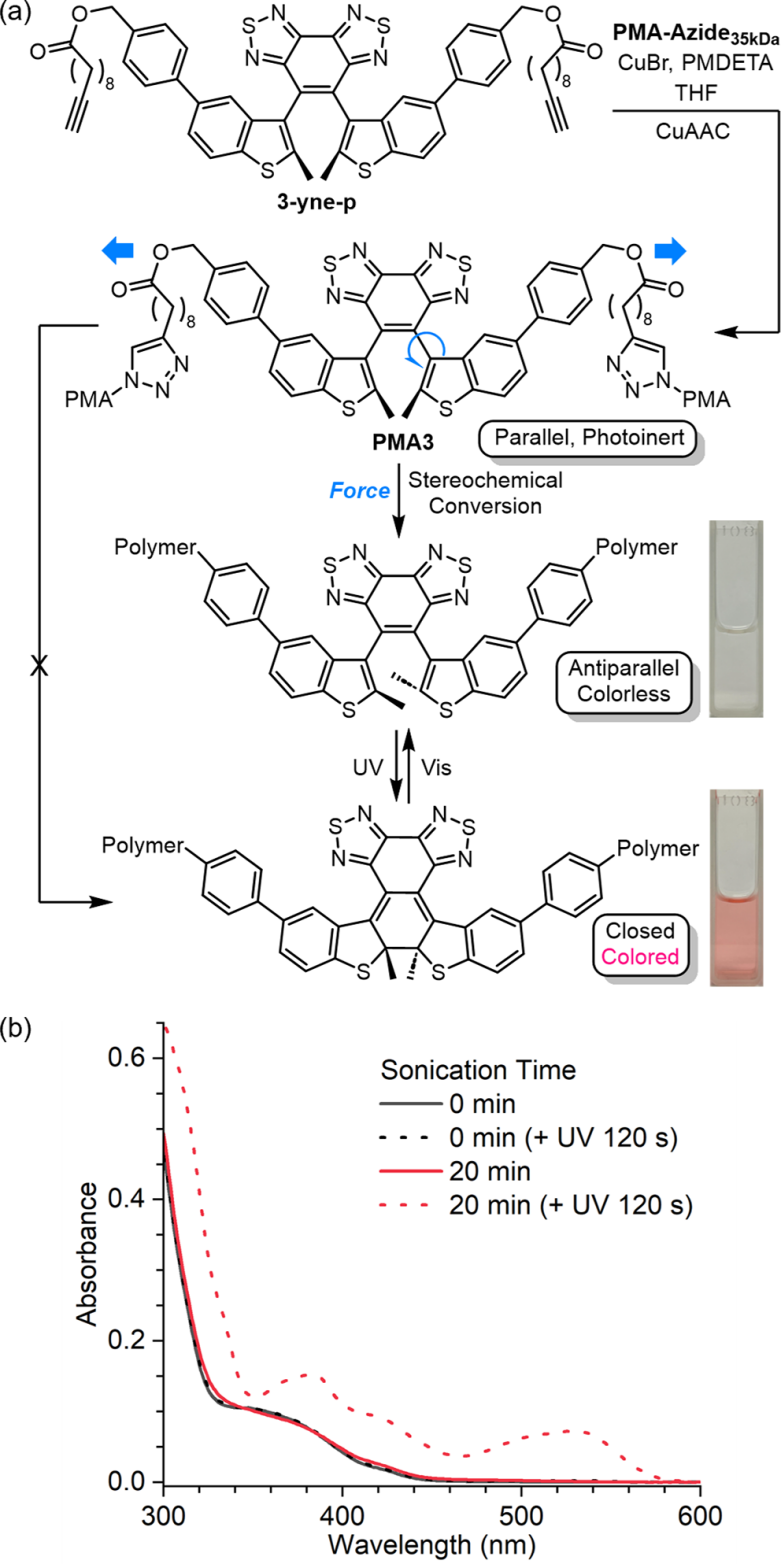
(a) Synthesis of **PMA3** containing a chain-centered mechanophore, and its stimuli-responsive properties. Force-triggered atropisomerization of the parallel diarylethene generates a racemate of antiparallel isomers, but only one antiparallel isomer is shown for simplicity. (b) Ultrasonication-dependent photochromism of **PMA3**.

Further, we systematically compared the activation rates of chain-centered mechanophores through solution-phase ultrasonication experiments. Ultrasound acoustic field causes pressure variation in the solution and generates rapidly collapsing cavitation, inducing a solvodynamic shear force field that transduces force to mechanophores covalently embedded in the backbone of dissolved polymers.^3^ Force is maximized at the midpoint of the polymer chain, and longer chains experience greater force. Rates of mechanophore conversion in ultrasonication experiments are frequently used as a measure to assess the relative reactivities among different mechanophores.^71,77,90,91^ When mechanophores are incorporated at the center of linear polymers with identical lengths, faster activation rates suggest higher mechanical reactivity. We synthesized **PMA1**-**PMA3** containing chain-centered mechanophores **M1**-**M3** by tethering bis-functionalized mechanophores to identical azide-functionalized poly(methyl acrylate) polymers (**PMA-Azide**, Mn^NMR^ = 34.7 kg/mol) through CuAAC click chemistry (Figure 4a and SI).^92^ This method guarantees uniform chain lengths for **PMA1**-**PMA3**, ensuring that all chain-centered mechanophores experience similar force environments under standard ultrasonication treatments. Solvodynamic force converts the photoinert parallel diarylethenes to their photoswitchable antiparallel diastereomers, as illustrated by the sonication-dependent photoactivity of **PMA3** (Figure 4b). A solution of **PMA3** (15 mL, 1.0 mg/mL in acetonitrile) was initially colorless and remained colorless after UV exposure. In contrast, UV irradiation (λ = 365 nm) turned the ultrasound-activated polymer sample into a red color, with an absorption peak emerging at around 530 nm. Moreover, the ultrasonicated **PMA3** solution could be switched between the colored and colorless forms reversibly under UV and visible irradiation, matching our previous findings.^65^ We observed minimal fatigue after six cycles of UV irradiation at 365 nm and four cycles at 254 nm (Figure S12). **PMA1** and **PMA2** exhibit similar sonication-dependent photochromic properties (SI Sections 4 and 5).

**Figure 5:**
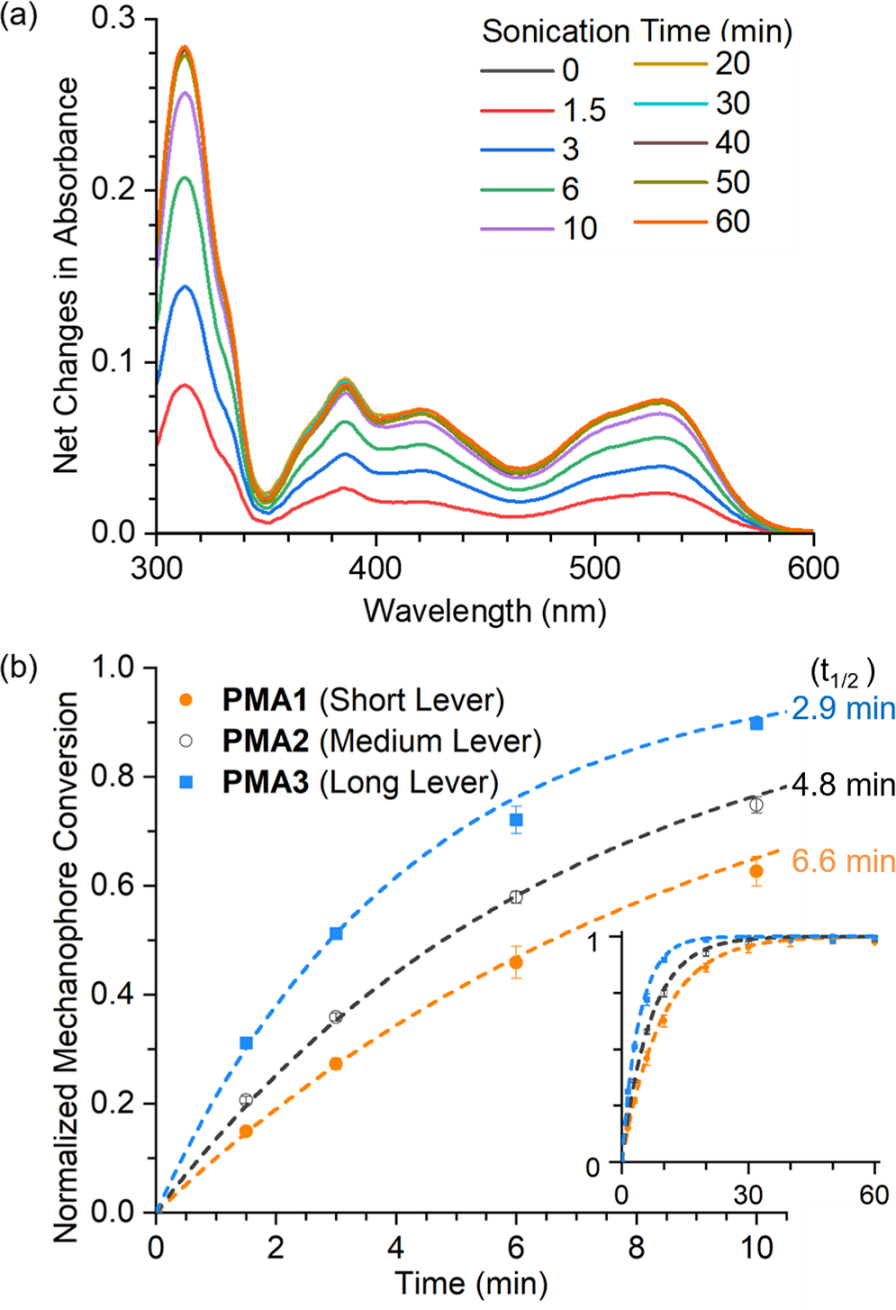
(a) UV-induced absorbance changes (photostationary-state spectrum minus non-photoirradiated spectrum, see Figure S11 for data processing) for sonicated **PMA3** solutions. (b) Time-course sonomechanical activation of **PMA1**-**PMA3** fitted into pseudo-first-order rate expressions (Figure S13).

This photoactivity change provides a convenient readout to monitor the ultrasound-mediated mechanochemical activation of **PMA1**-**PMA3** by measuring their photostationary-state absorbance with UV–vis spectroscopy (Figure 5a and Figures S9–S11). A polymer solution was subjected to standard ultrasonication conditions, and aliquots of the solution were removed and analyzed after each duration of ultrasonication. All initial polymer solutions containing the parallel diarylethene mechanophores were colorless and photoinert. Exposing the sonicated solutions to UV irradiation (λ = 365 nm) leads to the development of a red color due to the photoexcitation of converted mechanophores, with an absorption peak at around 510–530 nm. Their photostationary-state absorption were measured as a function of ultrasonication time (Figures S9–S11), with the UV-induced absorbance changes for sonicated **PMA3** solutions shown in Figure 5a as a representative example. Their peak absorbance values in the visible region were used to calculate the percentage mechanochemical conversion. By fitting the ultrasonication-dependent conversion to a first-order rate expression (Figures 5b and S13), the rate constants of the pseudo-first-order sonomechanical reactions are calculated to be 0.105 ± 0.003, 0.145 ± 0.003, and 0.239 ± 0.007 min^–1^ for **PMA1**-**PMA3**, respectively. The trend of these sonomechanical activation rates is consistent with the mechanosensitivity trend among **M1**-**M3** observed in our SMFS and CoGEF results, collectively supporting the lever-arm SMAR hypothesis.

**Figure 6:**
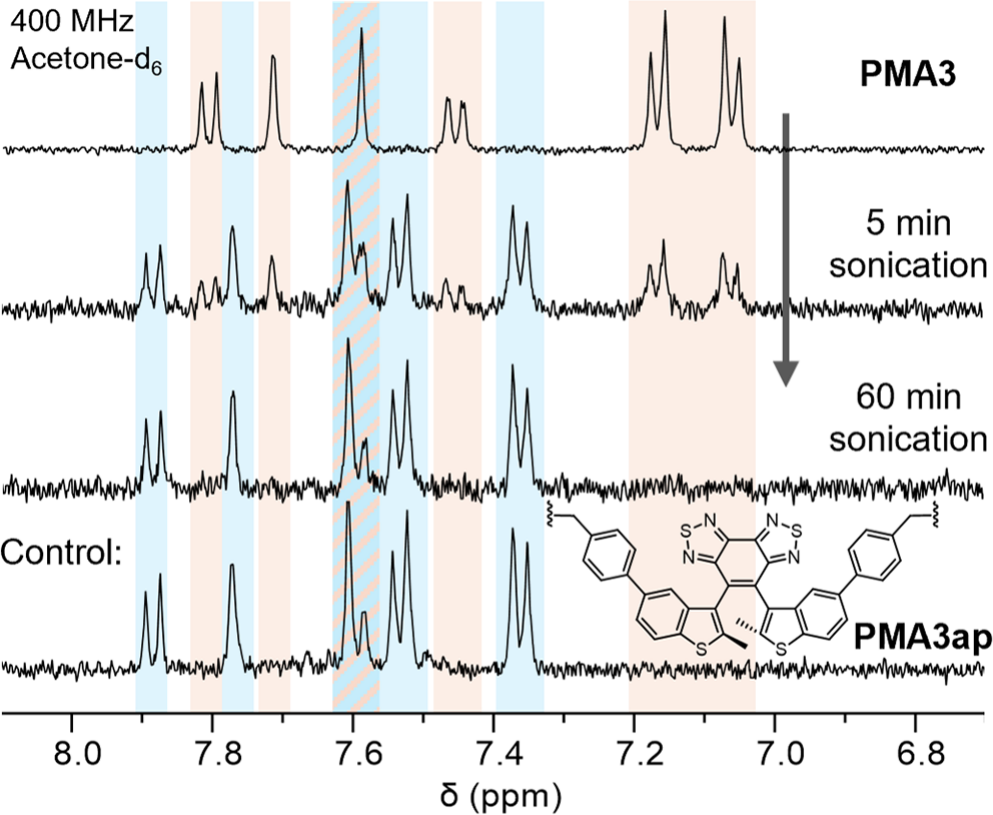
Partial ^1^H NMR spectra of **PMA3** (acetone-*d*_6_) subjected to different ultrasonication conditions: no sonication (top trace), 5 and 60 min of ultrasonication (2nd and 3rd traces, respectively). The bottom trace corresponds to a separately synthesized control polymer **PMA3ap** incorporating an antiparallel diarylethene. Orange shade: parallel diarylethene; blue shade: antiparallel diarylethene; blended: overlapped peaks.

NMR-measured mechanical conversions of **PMA1**-**PMA3** align with results from UV–vis studies. Polymers were sonicated in acetonitrile (15 mL, 2 mg/mL) and isolated, and their structures were analyzed using NMR spectroscopy. As a representative, Figure 6 shows the ^1^H NMR spectrum of **PMA3** subjected to ultrasonication: a new set of resonances (blue shade) emerged, consistent with the structure of antiparallel diarylethenes observed in a separately synthesized control polymer **PMA3ap**. NMR results indicate that sonication for 5 min converts approximately 40.2%, 52.5%, and 63.7% of **PMA1**, **PMA2**, and **PMA3**, respectively (see Figures S14–S16). These NMR results align with the pseudo-first-order kinetics determined by UV–vis for **PMA1**-**PMA3** (Figures 5b and S13), which predicted conversions of 40.8, 51.6, and 69.8% after the same ultrasonication duration, respectively.

The mechanical conversion of all three polymers **PMA1**-**PMA3** rapidly approached completion after about 20 min ultrasonication, whereas there were minor changes in their polymer molecular weights over the same period as indicated by size exclusion chromatography (SEC) (Figure S17). This highlights another feature of the diarylethene mechanophores that their noncovalent-yet-chemical transformation signals stress without sacrificial bond scission, minimizing the impact on the intrinsic properties of the polymer matrix. Additionally, control polymers containing **M1**-**M3** at PMA chain ends remained photoinert before and after identical ultrasound treatments, confirming the mechanical origin of the observed changes from ultrasonication experiments (Figure S18).

To test the thermal stability of diarylethene atropisomers, solutions of synthetic intermediates **1-yne-p**, **2-yne-p**, and **3-yne-p** (comprising **M1**-**M3** moieties, respectively) in DMSO-*d*_6_ were heated to 100 °C for 12 h. Subsequent NMR analysis revealed negligible shifts in their resonances (Figures S19–S21), demonstrating these atropisomers’ excellent thermal stability even at elevated temperatures. Antiparallel diarylethenes also exhibited excellent stability under similar thermal conditions (Figures S22–S24). These experimental results corroborate with DFT-predicted high rotational barriers of 218, 182, and 178 kJ/mol for the thermal atropisomerization of model **M1**-**M3** structures (Figure S4), respectively. The excellent thermal stability of parallel diarylethene mechanophores and their inherent photoinertness make them well suited as molecular force sensors with high specificity to mechanical stimuli,^38^ offering advantages over mechanophores that are either thermally (e.g., diarylbibenzofuranone)^93^ or photochemically active (e.g., spiropyran).^94^

## Conclusions

In summary, this study unambiguously establishes the exceptional mechanosensitivity of chemo-mechanical coupling in diarylethene atropisomers, as evidenced through both computational and experimental methods. The F* value for the previously introduced **M2** structure is 197 pN ± 12 pN as determined by SMFS. This value is lower than those typically observed in other mechanically induced chemical reactions studied to date by SMFS, corroborating our previous indirect findings. Additionally and importantly, we introduce an intuitive “lever-arm” effect that allows for the fine-tuning of the mechanical reactivity in diarylethene configurational mechanophores, leading to the development of a new mechanophore **M3** which exhibits further increased mechanosensitivity with an F* value of 131 pN ± 4 pN. These atropisomeric diarylethene mechanophores also feature excellent thermal stability and are inherently photoinert, making them well suited as molecular force probes with high specificity to mechanical stimuli. This study lays the groundwork for exploring the SMAR in this mechanistically distinct class of atropisomeric configurational mechanophores. It also paves the way for designing highly sensitive and irreversible mechanochemical processes that are crucial for understanding nanoscale mechanical behaviors in various synthetic and biological materials.

## Supporting Information Available

Experimental details; synthetic procedures; DFT calculations; SMFS data; SEC data; UV–vis; and NMR spectra (PDF)
