# Supplementary material for: Advancing the Mechanosensitivity of Atropisomeric Diarylethene Mechanophores through a Lever-Arm Effect
Source: J Am Chem Soc. 2025 Jan 10;147(3):2502–9. doi: 10.1021/jacs.4c13480 (PMC11760174; doi:10.1021/jacs.4c13480)
Supplement: Supplementary file 1 — ja4c13480_si_001.pdf [file ja4c13480_si_001.pdf]

# Supporting Information

# Advancing the Mechanosensitivity of Atropisomeric Diarylethene Mechanophores Through a Lever-Arm Effect

Cijun Zhang,† Tatiana B. Kouznetsova,‡ Boyu Zhu,† Liam Sweeney,† Max Lancer,† Ivan Gitsov,§,⁑ Stephen L. Craig,‡ and Xiaoran Hu*,†

†Department of Chemistry, BioInspired Institute, Syracuse University, Syracuse, New York 13244, United States

‡Department of Chemistry, Duke University, Durham, North Carolina 27708, United States

§Department of Chemistry, The Michael M. Szwarc Polymer Research Institute, State University of New York - ESF, Syracuse, New York 13210, United States

⁑Department of Biomedical and Chemical Engineering, BioInspired Institute, Syracuse University, Syracuse, New York 13244, United States

*To whom all correspondence should be addressed: Dr. Xiaoran Hu, E-mail: xhu156@syr.edu

## Contents

1. **General Considerations** .......... 1
2. **DFT Calculations** .......... 1
3. **Single-Molecule Force Spectroscopy (SMFS) Experiments** .......... 4
4. **Investigation of the Photochromic Properties of Antiparallel Diarylethenes** .......... 6
5. **Investigation of Mechanical Activation Through Solution-Phase Ultrasonication** .......... 8
6. **Investigation of the Thermal Stability of Atropisomeric Diarylethenes** .......... 18
7. **Synthetic Details** .......... 21
8. **NMR Spectra** .......... 54

## 1. General Considerations

All reactions were carried out under standard air-free conditions in a nitrogen gas atmosphere with magnetic stirring, unless otherwise stated. Commercially purchased reactants and solvents were used without further purification unless specified. Flash chromatography was performed using a Biotage Isolera System with Yamazen Corp. universal silica gel columns (Pore Size 60 angstroms, Particle Size 40-63 microns).

NMR spectra were recorded on a Bruker Avance III HD 400 MHz spectrometer or Bruker Avance III HD 800 MHz spectrometer. $^{1}$H NMR spectra are referenced to residual protonated solvent (7.26 ppm for $CHCl_3$). $^{13}$C NMR spectra are referenced to solvent signals (77.16 ppm for $CHCl_3$). Multiplicity abbreviations: s = singlet, d = doublet, t = triplet, q = quartet, dd = doublet of doublets, ABq = AB quartet, m = multiplet, br = broad.

Mass spectra were acquired using a DART-SVP (Direct Analysis in Real Time) ion source (IonSense, Saugus, MA) coupled to an Exactive Orbitrap mass spectrometer (Thermo Scientific, Bremen, Germany) at the Cornell Chemistry Mass Spectrometry Facility.

Molecular weight distributions of polymers were measured in the Michael M. Szwarc Polymer Research Institute at the State University of New York – ESF using Waters size-exclusion chromatography (SEC) line at 40°C in THF with a flow rate of 0.8 mL/min. The molecular weight characteristics were calculated using calibration with fourteen poly(styrene) standards (Polymer Standards Service) and OmniSEC 5.0 software (Malvern).

Solution optical spectra were acquired in quartz cuvettes. Electronic absorbance spectra were obtained using an Evolution 201 UV-visible spectrophotometer in double-beam mode with a solvent-containing cuvette for background subtraction.

Ultrasonication experiments were conducted using a Vibra Cell 505 liquid processor equipped with a 13 mm full wave solid probe (254 mm long, Sonics, part #630-0217), sonochemical adapter (Sonics, part #830-00014), and a 10-50 mL reaction vessel (Sonics, part #830-00012). All sample solutions were purged with argon for 20 minutes before ultrasonication. Argon gas bubbling was continued throughout ultrasonication experiments. The reaction vessel was immersed in an ice bath during ultrasonication. Ultrasound treatments were performed in pulse mode (1s on/2s off) with 20% amplitude. Reported sonication times refer to the "sonication-on" time.

UV irradiations at 365 nm or 254 nm were carried out using a hand-held UV lamp (Chemglass, part # CLS-1625). Visible light irradiations were performed using an iPhone flashlight.

## 2. DFT Calculations

Density Functional Theory (DFT) calculations were performed using the constrained geometries simulate external force (CoGEF) technique on Spartan '20 at the B3LYP/6-31G* level of theory. (*J. Chem. Phys.* **2000**, *112*, 7307-7312; *J. Am. Chem. Soc.* **2020**, *142*, 16364-16381). A truncated

diarylethene structure was initially equilibrated, and then the distance between the two terminal methyl groups was incrementally increased in small steps (0.05 Å per step). The energy of the molecule was minimized at each step, and the force at each elongation step was calculated from the slope of the energy-strain curve.

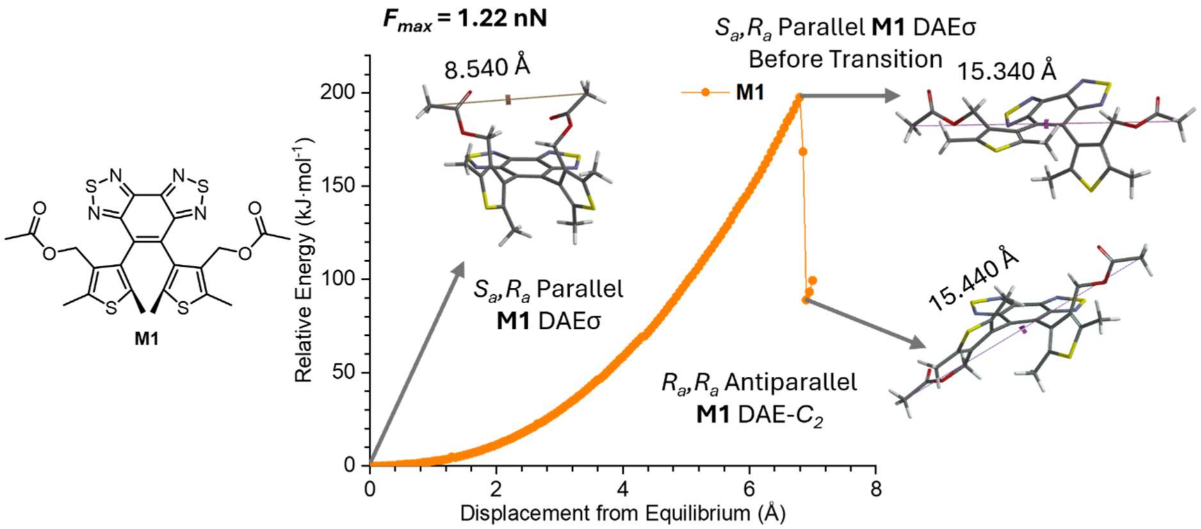


**Figure S1.** The chemical structure of the model mechanophore **M1** and its DFT calculated structures under different constrained conditions as indicated by the arrow. The maximum force 1.22 nN was calculated from the slope of the curve.

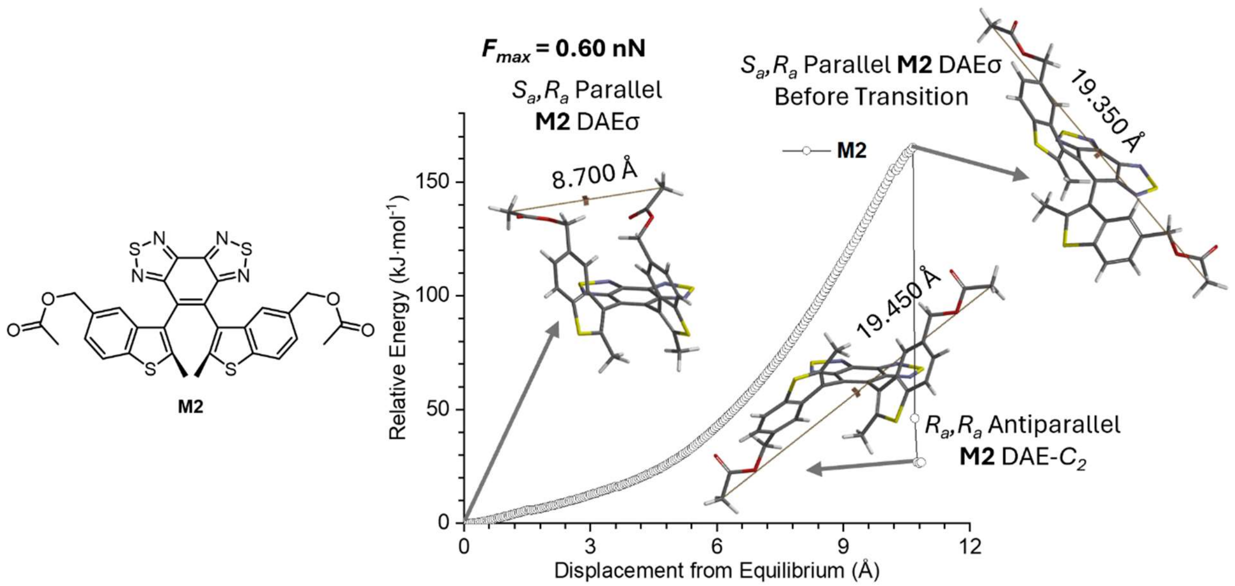


**Figure S2.** The chemical structure of the model mechanophore **M2** and its DFT calculated structures under different constrained conditions as indicated by the arrow. The maximum force 0.60 nN was calculated from the slope of the curve.

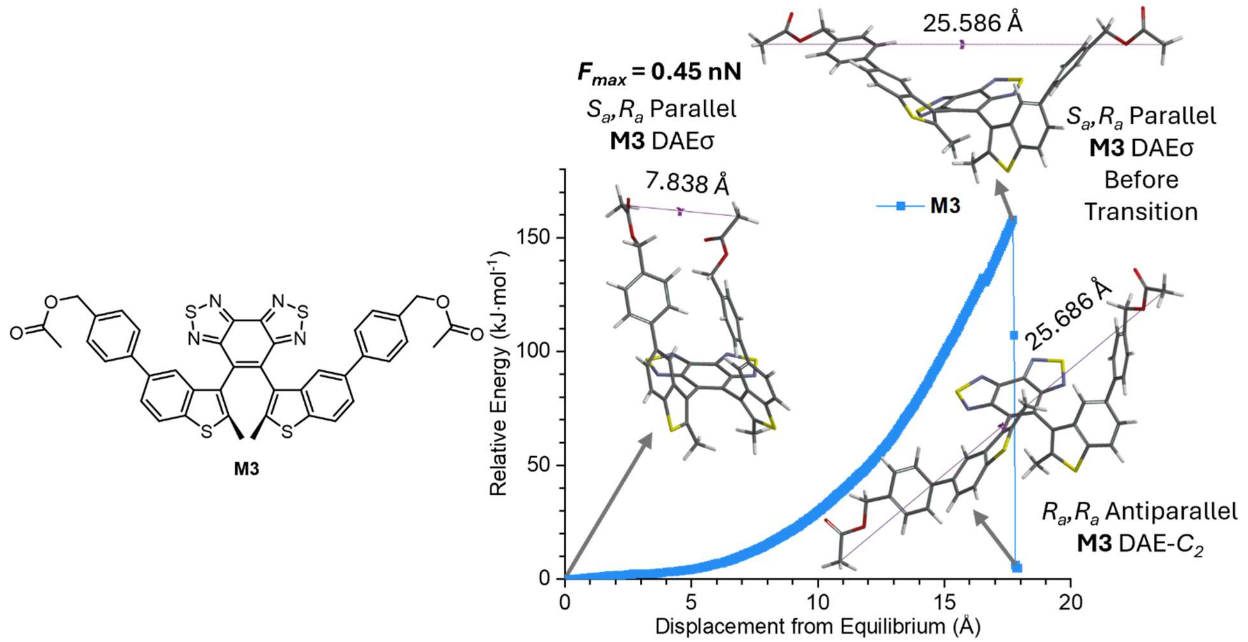


**Figure S3.** The chemical structure of the model mechanophore **M3** and its DFT calculated structures under different constrained conditions as indicated by the arrow. The maximum force 0.45 nN was calculated from the slope of the curve.

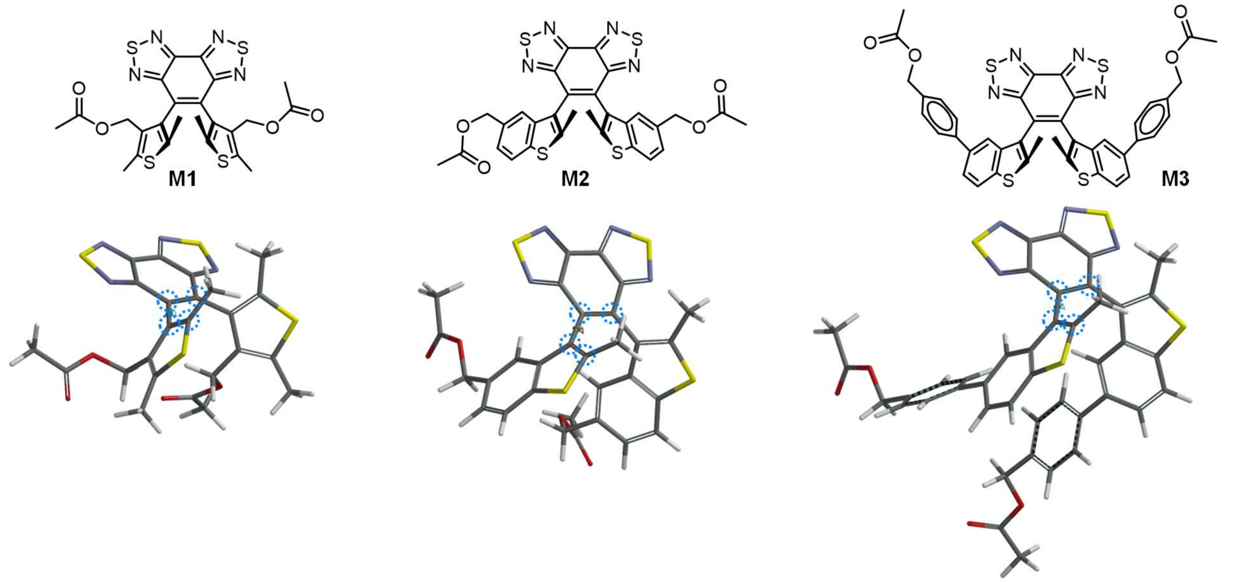


**Figure S4.** The dihedral angle between the ethene bridge and the aryl ring is increased in small incremental steps (1˚ per step) from DFT-calculated equilibrium geometry (as indicated by the blue circles in Figure S4), and the energy of the molecule is minimized at each step. The peak relative energy (i.e., the thermal rotational barrier) is calculated to be 218 kJ/mol, 182 kJ/mol, and 178 kJ/mol for **M1**-**M3**, respectively. DFT calculations were conducted at the B3LYP/6-31G* level of theory.

## 3. Single-Molecule Force Spectroscopy (SMFS) Experiments

Single-molecule force spectroscopy experiments were conducted on a home-built instrument. Sharp Microlever Silicon probes (MSNL) and Silicon Nitride probes (PNP-DB) were purchased from Bruker (Camarillo, CA) and NanoAndMore USA (Watsonville, CA) correspondingly. Cantilever spring constants were calibrated in air using thermal vibration method. Silicon wafers, cut into 1x1 cm pieces were cleaned by submerging into hot piranha solution (1:3 $H_2SO_4$:$H_2O_2$) for 30 min, thoroughly rinsed with DI water and dried under a stream of nitrogen. Probes were cleaned by rinsing with DCM and methanol, after which they were placed into UVO cleaner (Jelight 42) for 15 min. To improve polymer attachment, Si surfaces and probes were further functionalized by submerging into a 4% solution of (3-Aminopropyl)triethoxysilane in methanol for 25 min and 7 min, respectively. Subsequently, they were rinsed by methanol and annealed at 110 °C for 30 min. PNP probes were used without further modification. 20 µL of dilute polymer solution (0.1 mg/mL) in THF was deposited on the surface and allowed to evaporate, after which the surface was rinsed with THF to remove non-attached polymer chains and placed on an AFM stage. Experiments were conducted in a semi-enclosed fluid cell in toluene. During the acquisition a 500 Hz low-pass filter was used to reduce noise. Collection and processing of data was done using Matlab. For each polymer type experiments were conducted using several sample preparations and probes.

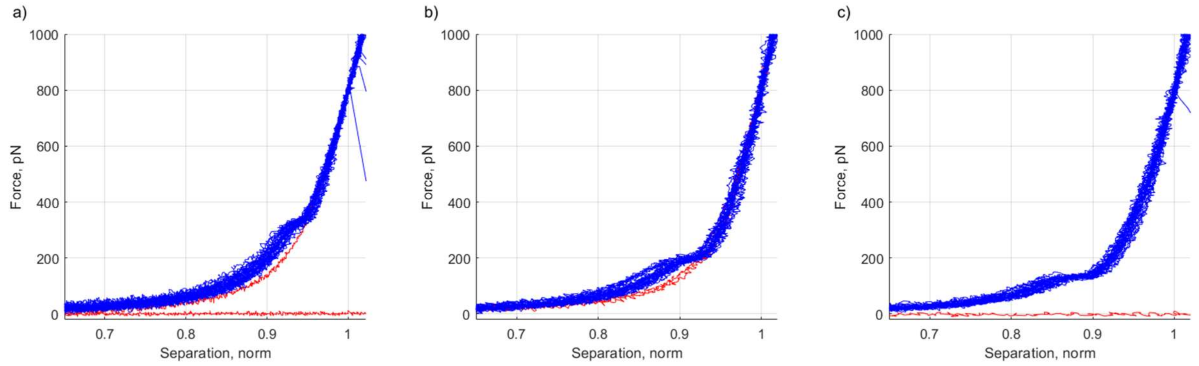


**Figure S5.** All SMFS data obtained for the polymers **P1** (a), **P2** (b) and **P3** (c), normalized at 0.8 nN force. Characteristic transition force values were determined as an inflection point on the transition 'plateau' as described previously (*J. Am. Chem. Soc.* **2015**, *137*, 6148–6151).

**Table S1.** List of SMFS pulls for each polymer sample and fitted transition force values and lengths.

| P1 | | | P2 | | | P3 | | |
|---|---|---|---|---|---|---|---|---|
| **N** | **Ftr (pN)** | **L1 (nm)** | **N** | **Ftr (pN)** | **L1 (nm)** | **N** | **Ftr (pN)** | **L1 (nm)** |
| 1 | 322.5 | 257 | 1 | 198.2 | 158.1 | 1 | 135.7 | 136.9 |
| 2 | 331.5 | 259.3 | 2 | 194.3 | 105 | 2 | 142.2 | 124.8 |
| 3 | 335.4 | 289 | 3 | 196.2 | 186.4 | 3 | 130.5 | 190.6 |
| 4 | 371 | 196.5 | 4 | 198.9 | 177.4 | 4 | 127.5 | 108.4 |
| 5 | 336.4 | 158.8 | 5 | 206.5 | 106.6 | 5 | 133 | 259.4 |
| 6 | 333.7 | 235.4 | 6 | 207.1 | 185.5 | 6 | 131.3 | 151.7 |
| 7 | 332.8 | 303.4 | 7 | 203.4 | 291.9 | 7 | 131.7 | 187.7 |
| 8 | 329.8 | 195.7 | 8 | 210.1 | 216.3 | 8 | 135 | 155.3 |
| 9 | 327.8 | 432.9 | 9 | 200.6 | 227.1 | 9 | 129 | 139.8 |
| 10 | 319 | 213.5 | 10 | 201.2 | 93.1 | 10 | 129 | 246.9 |
| 11 | 327.8 | 289.2 | 11 | 194.6 | 172.5 | 11 | 135.2 | 167.5 |
| 12 | 327 | 178.4 | 12 | 197.5 | 305 | 12 | 133.2 | 109.1 |
| 13 | 324.4 | 125.9 | 13 | 204.4 | 108.5 | 13 | 129.1 | 97.8 |
| 14 | 320.5 | 269.5 | 14 | 214.4 | 180.3 | 14 | 128.4 | 109.6 |
| 15 | 327.7 | 413.2 | 15 | 215.6 | 177.3 | 15 | 130.5 | 214 |
| 16 | 325.7 | 155.7 | 16 | 223.7 | 157.9 | 16 | 128.3 | 94.4 |
| 17 | 318.1 | 424.4 | 17 | 191.6 | 151.3 | 17 | 131.9 | 152 |
| 18 | 320.7 | 512.6 | 18 | 181.4 | 244.4 | 18 | 122.8 | 85.2 |
| 19 | 333 | 253.7 | 19 | 189.8 | 91.9 | 19 | 129.8 | 137.5 |
| 20 | 327.9 | 335.5 | 20 | 191.8 | 218.2 | 20 | 134.6 | 153 |
| 21 | 326.2 | 330.4 | 21 | 202.9 | 173.6 | 21 | 128.3 | 167.8 |
| 22 | 325.1 | 395.9 | 22 | 184.2 | 316.8 | 22 | 128.7 | 144.6 |
| 23 | 309.5 | 202.2 | 23 | 190.9 | 195.6 | 23 | 131.9 | 125 |
| 24 | 316.4 | 105.2 | 24 | 189.7 | 93.3 | | | |
| 25 | 327.6 | 215.8 | 25 | 183.3 | 382.9 | | | |
| 26 | 333.3 | 220.6 | 26 | 168 | 254.1 | | | |
| 27 | 331.4 | 166 | 27 | 181.2 | 142.4 | | | |

## 4. Investigation of the Photochromic Properties of Antiparallel Diarylethenes

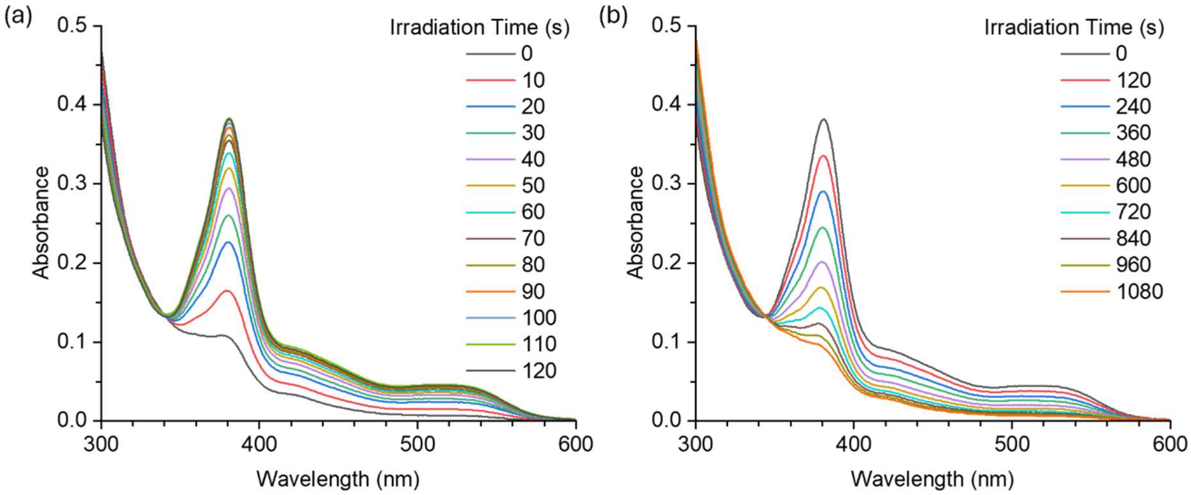


**Figure S6.** (a) The photochromism of a 2 mg/mL solution of **PMA1ap** in THF under 365 nm UV irradiation. The photostationary state (PSS) was achieved after about 100 s irradiation. (b) Subsequent visible-light irradiation of this polymer solution resulted in the ring-opening reaction and discoloration. The color completely disappeared after about 960 s visible irradiation using a white flashlight.

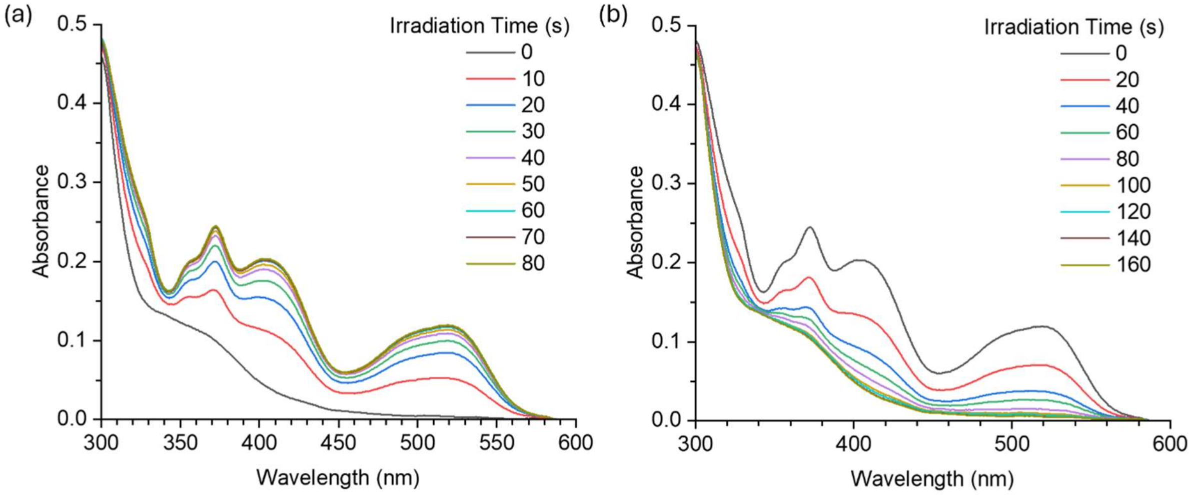


**Figure S7.** (a) The photochromism of a 2mg/mL solution of **PMA2ap** in THF under 365 nm UV irradiation. The PSS was achieved after about 80 s irradiation. (b) Subsequent visible-light irradiation of this polymer solution resulted in the ring-opening reaction and discoloration. The color completely disappeared after about 160 s visible irradiation using a white flashlight.

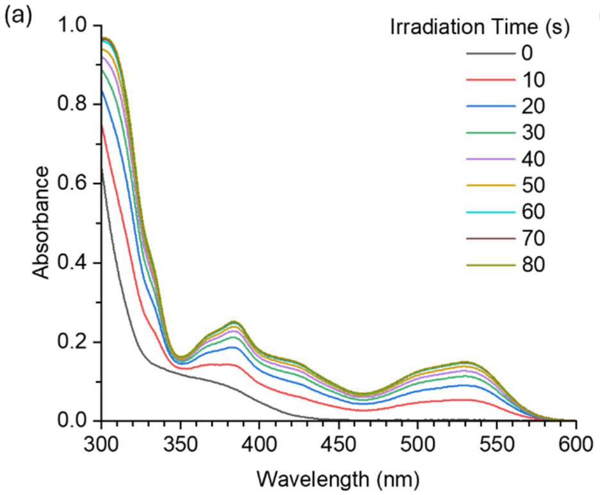


**Figure S8.** (a) The photochromism of a 2 mg/mL solution of **PMA3ap** in THF under 365 nm UV irradiation. The PSS was achieved after about 80 s irradiation. (b) Subsequent visible-light irradiation of this polymer solution resulted in the ring-opening reaction and discoloration. The color completely disappeared after about 160 s visible irradiation using a white flashlight.

## 5. Investigation of Mechanical Activation Through Solution-Phase Ultrasonication

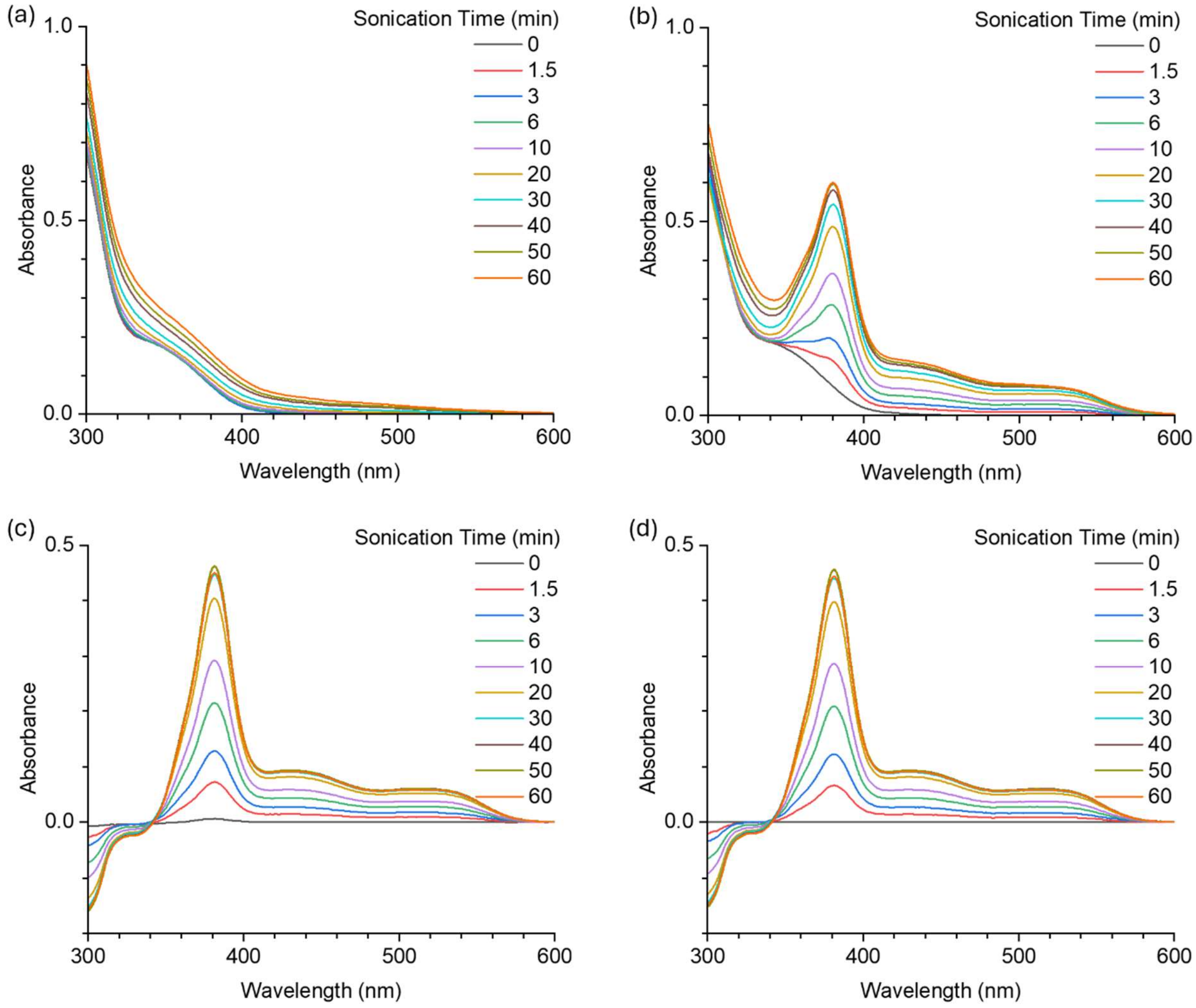


**Figure S9.** A representative solution phase ultrasonication of **PMA1** monitored by UV-vis. (a) The absorbance of a 2 mg/mL acetonitrile solution of **PMA1** gradually increased as sonochemical byproducts accumulated during ultrasonication. (b) Absorbance of ultrasonicated **PMA1** in photostationary state (PSS) as a function of ultrasonication time. To highlight the photochromic portion of **PMA1** (corresponding to the mechanochemically activated mechanophores) from the background, we generated plot (c) by subtracting each curve in (a) from the corresponding curves in (b). Plot (c) clearly shows an increase in the concentration of force-activated, photoswitchable form of **PMA1** over the course of ultrasonication. Further subtracting the 0 min sonication curve from each curve in (c) generates plot (d). Data in plot (d), along with that from two additional repeating experiments, is used to calculate mechanical activation rates in Figure S13. PSS was achieved by 120 s irradiation with a portable UV lamp at 365 nm.

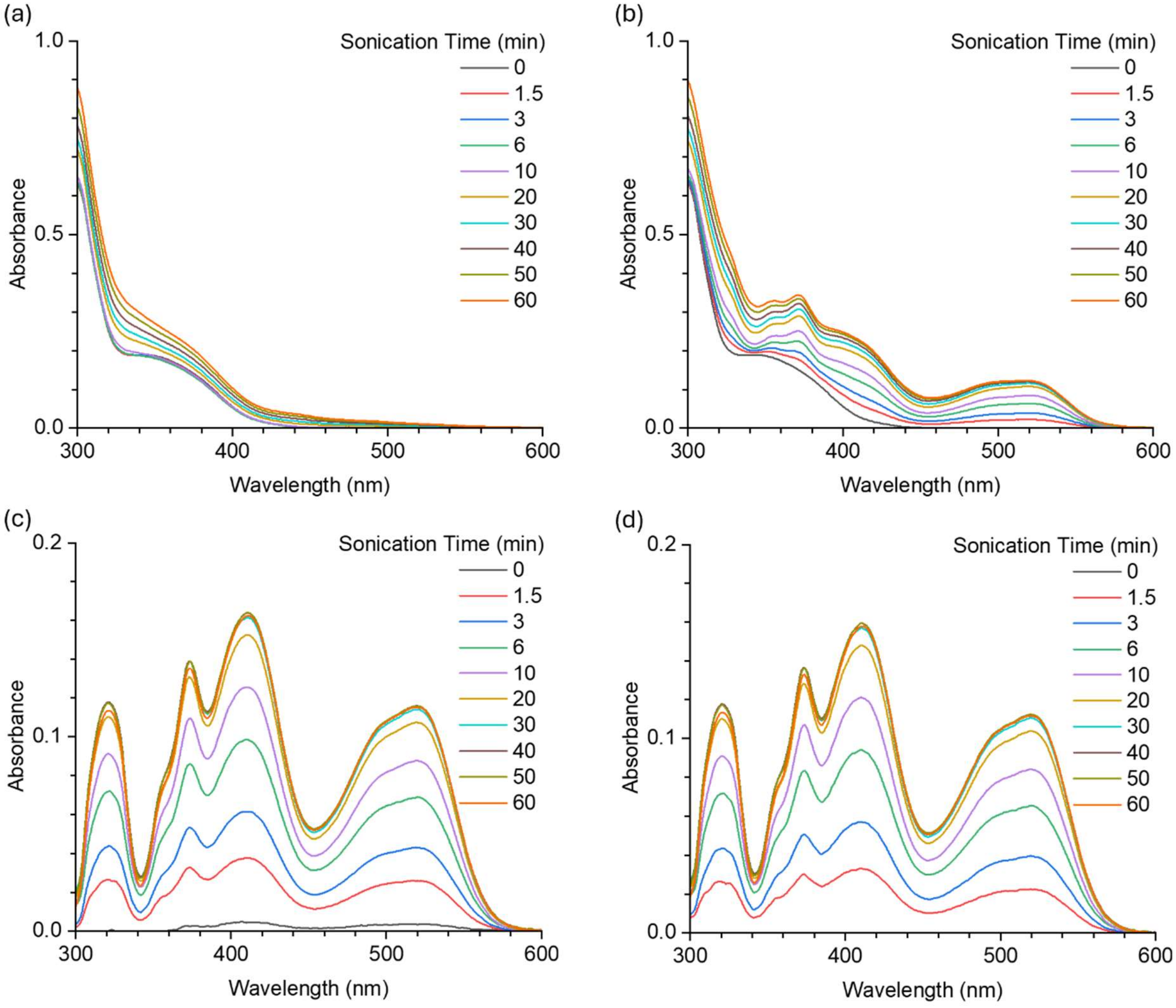


**Figure S10.** A representative solution phase ultrasonication of **PMA2** monitored by UV-vis. (a) The absorbance of a 2 mg/mL acetonitrile solution of **PMA2** gradually increased as sonochemical byproducts accumulated during ultrasonication. (b) Absorbance of ultrasonicated **PMA2** in PSS as a function of ultrasonication time. To highlight the photochromic portion of **PMA2** (corresponding to the mechanochemically activated mechanophores) from the background, we generated plot (c) by subtracting each curve in (a) from the corresponding curves in (b). Plot (c) clearly shows an increase in the concentration of force-activated, photoswitchable form of **PMA2** over the course of ultrasonication. Further subtracting the 0 min sonication curve from each curve in (c) generates plot (d). Data in plot (d), along with that from two additional repeating experiments, is used to calculate mechanical activation rates in Figure S13. PSS was achieved by 120 s irradiation with a portable UV lamp at 365 nm

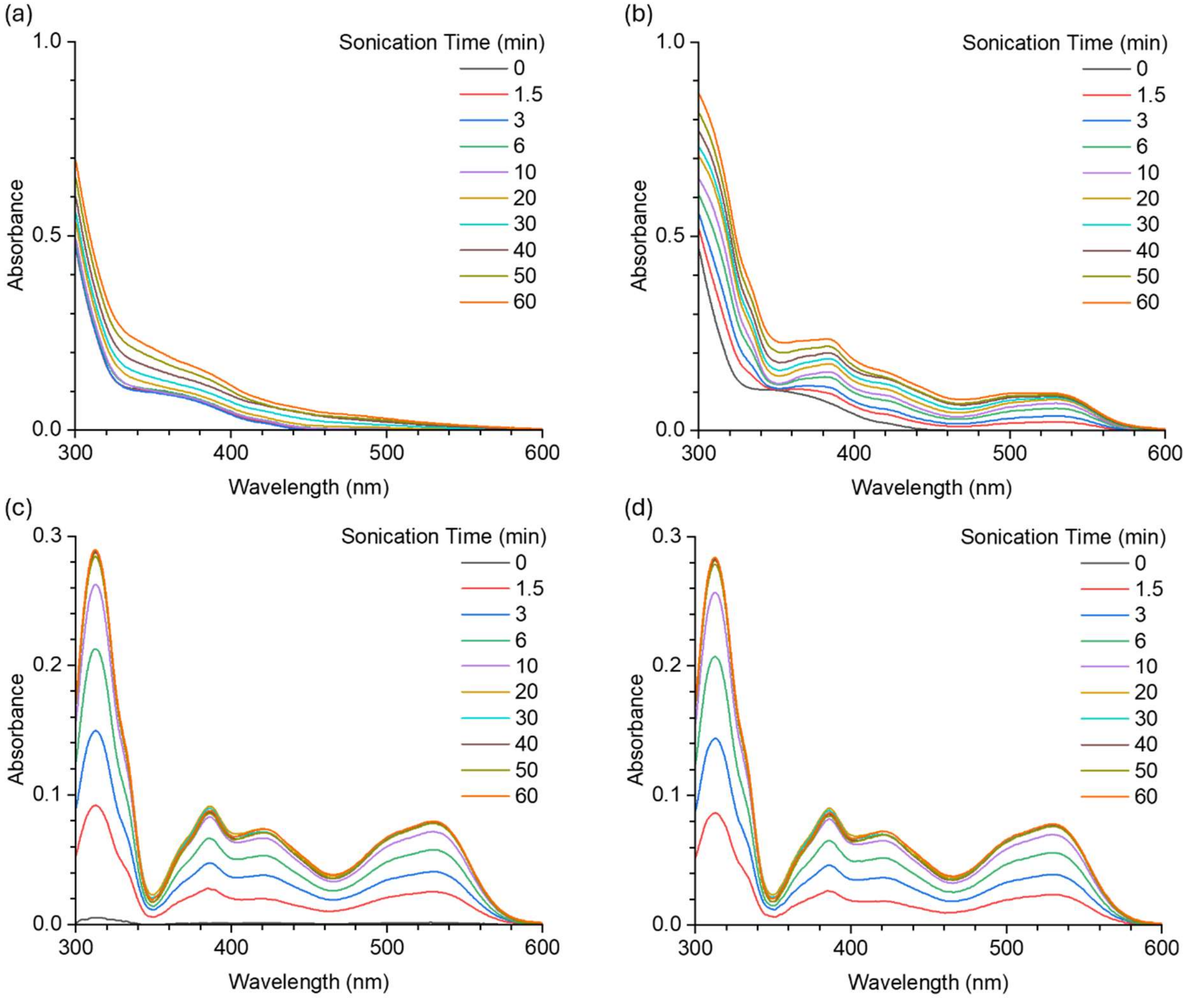


**Figure S11.** A representative solution phase ultrasonication of **PMA3** monitored by UV-vis. (a) The absorbance of a 1 mg/mL acetonitrile solution of **PMA3** gradually increased as sonochemical byproducts accumulated during ultrasonication. (b) Absorbance of ultrasonicated **PMA3** in PSS as a function of ultrasonication time. To highlight the photochromic portion of **PMA3** (corresponding to the mechanochemically activated mechanophores) from the background, we generated plot (c) by subtracting each curve in (a) from the corresponding curves in (b). Plot (c) clearly shows an increase in the concentration of force-activated, photoswitchable form of **PMA3** over the course of ultrasonication. Further subtracting the 0 min sonication curve from each curve in (c) generates plot (d). Data in plot (d), along with that from two additional repeating experiments, is used to calculate mechanical activation rates in Figure S13. PSS was achieved by 120 s irradiation with a portable UV lamp at 365 nm.

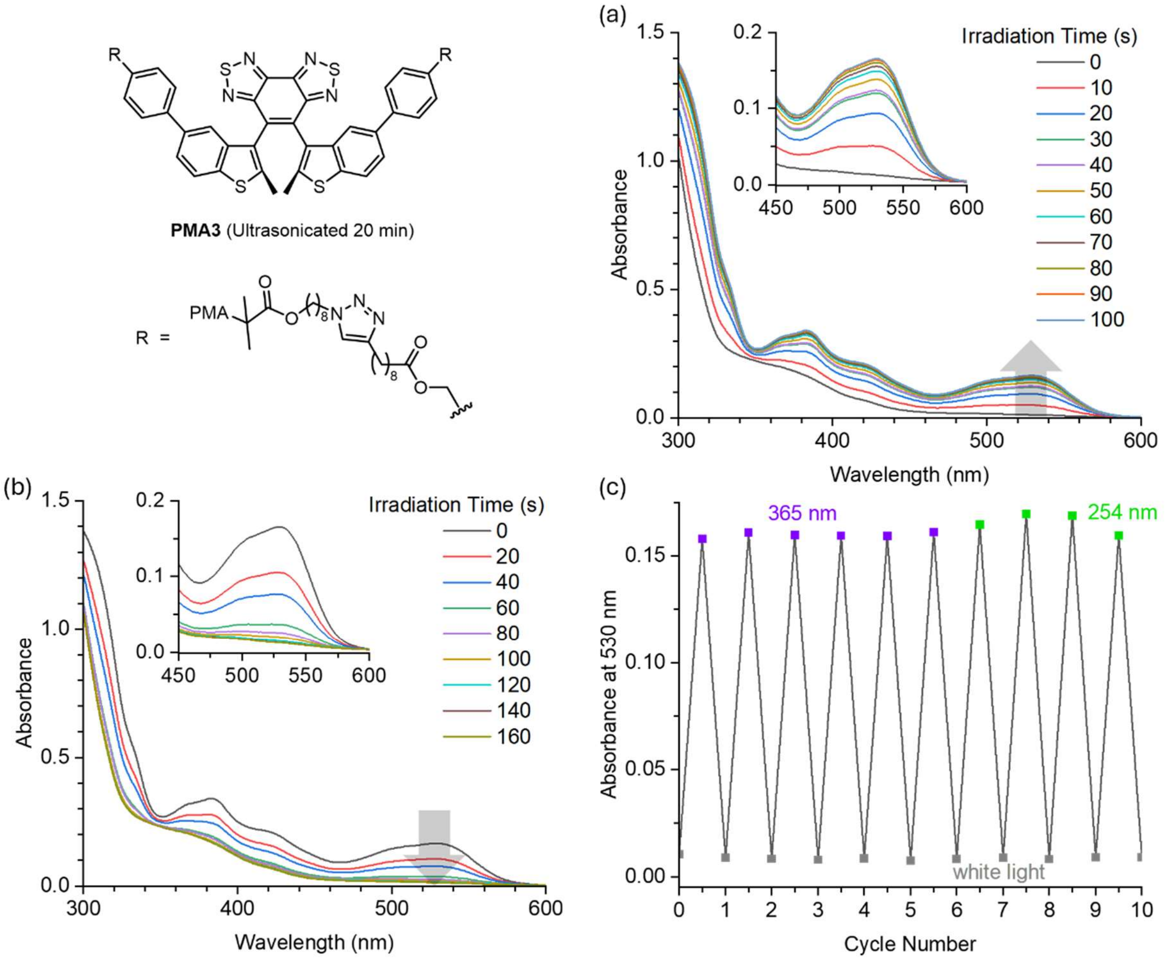


**Figure S12.** The chemical structure of polymer **PMA3** and its photochemical property after 20 min ultrasonication (2 mg/mL in acetonitrile) characterized by UV-vis spectroscopy. (a) Photochromism of the **PMA3** solution under 365 nm UV irradiation. The PSS was achieved after about 80 s irradiation. (b) Subsequent visible-light irradiation of this polymer solution resulted in the ring-opening reaction and discoloration. The color completely disappeared after about 160 s visible irradiation using a white flashlight. (c) Reversible photochromism of the sonicated **PMA3** solution under UV (λ = 365 nm or 254 nm) and visible light. The absorbance was monitored at the absorption peak of the ring-closed diarylethene at 530 nm.

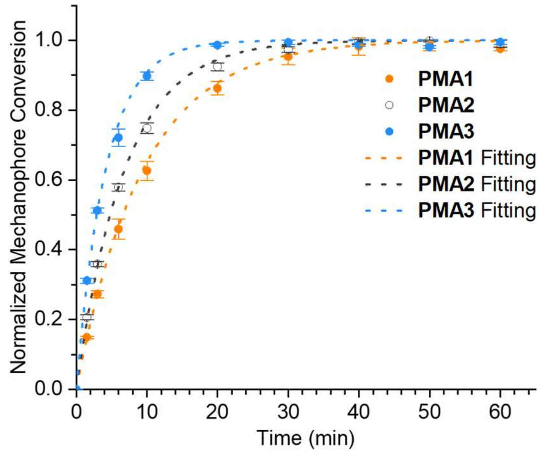


| Polymer | **PMA1** | **PMA2** | **PMA3** |
|---|---|---|---|
| k ($min^{-1}$) | 0.10526 ± 0.00346 | 0.14501 ± 0.0028 | 0.23915 ± 0.00721 |
| Reduced Chi-Sqr | 3.0647 | 1.56661 | 4.26242 |
| R-Square (COD) | 0.99952 | 0.99994 | 0.99983 |
| Adj. R-Square | 0.99952 | 0.99994 | 0.99983 |
| $t_{1/2}$ (min) | 6.6 ± 0.2 | 4.8 ± 0.1 | 2.9 ± 0.1 |

**Figure S13.** Comparison of the activation rates of **PMA1**, **PMA2** and **PMA3** in solution phase ultrasonication experiments. The percentage activation of **PMA1** is calculated and plotted based on results from Figure S9d (using peak absorbance at 513 nm) along with that from two additional repeating trials, employing a first-order rate expression $y = 1 - e^{-k*x}$ for normalization. The activation of **PMA2** is plotted analogously using peak absorbance at 520 nm from Figure S10d and two repeats. The activation of **PMA3** is plotted analogously using peak absorbance at 530 nm from Figure S11d and two repeats. Error bars represent the standard deviations for three trials for each material.

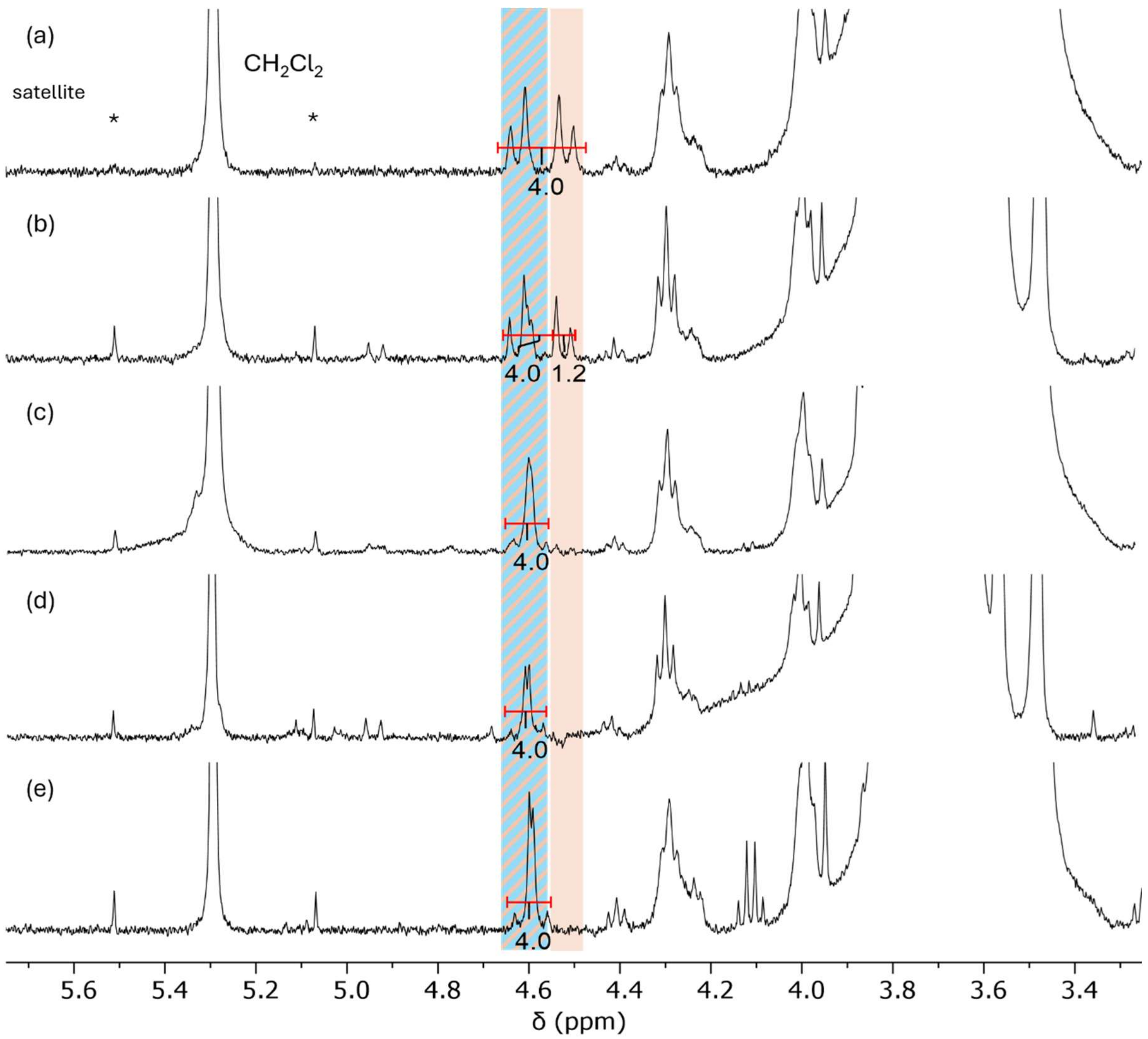


**Figure S14.** Partial $^1$H NMR spectra (400 MHz, Chloroform-d) of **PMA1** (a), **PMA1** after 5min ultrasonication (b), **PMA1** after 45 min ultrasonication (c), **PMA1** after 60 min ultrasonication (d), and control polymer **PMA1ap** (e). The spectra provide direct evidence for the U/S-triggered conversion of the parallel mechanophore to its antiparallel diastereomers. Solutions of **PMA1** (2 mg/mL) were subjected to standard ultrasonication conditions with indicated time, concentrated, and precipitated into cold methanol to afford the ultrasonicated polymer sample for NMR analysis. The conversion at 5 min is around 40.2%, which is consistent with the calculated conversion (40.8%) from the kinetic equation given in Figure S13. Orange shade indicates peaks assigned to **PMA1**, blue shade indicates peaks assigned to **PMA1ap**, and blue+orange shade indicates overlapped peaks.

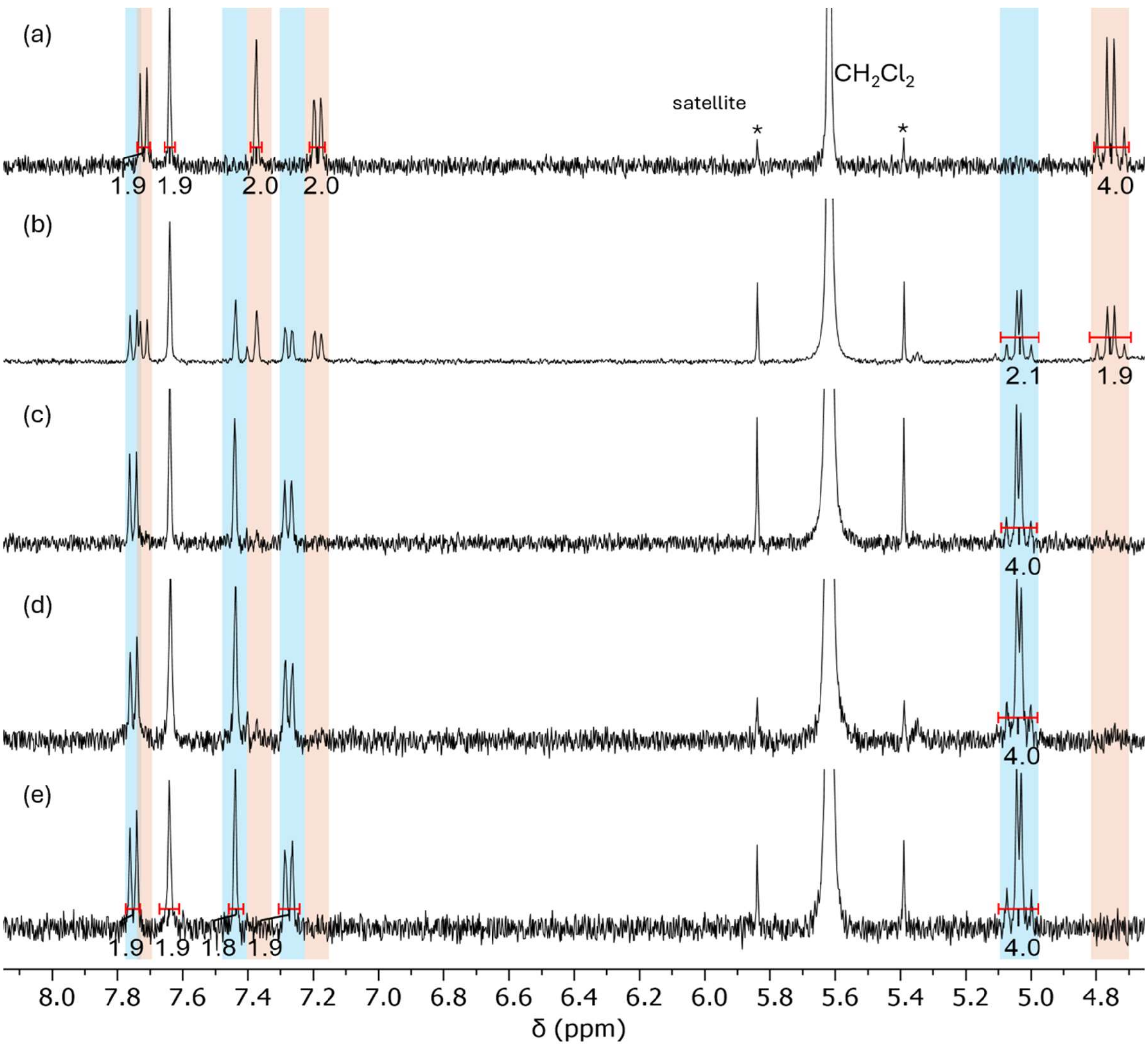


**Figure S15.** Partial $^1$H NMR spectra (400 MHz, Acetone-$d_6$) of **PMA2** (a), **PMA2** after 5min ultrasonication (b), **PMA2** after 45 min ultrasonication (c), **PMA2** after 60 min ultrasonication (d), and control polymer **PMA2ap** (e). The spectra provide direct evidence for the U/S-triggered conversion of the parallel mechanophore to its antiparallel diastereomers. Solutions of **PMA2** (2 mg/mL) were subjected to standard ultrasonication conditions with indicated time, concentrated, and precipitated into cold methanol to afford the ultrasonicated polymer sample for NMR analysis. The conversion at 5 min is around 52.5%, which is consistent the calculated conversion (51.6%) from the kinetic equation given in Figure S13. Orange shade indicates peaks assigned to **PMA2**, blue shade indicates peaks assigned to **PMA2ap**, and blue+orange shade indicates overlapped peaks.

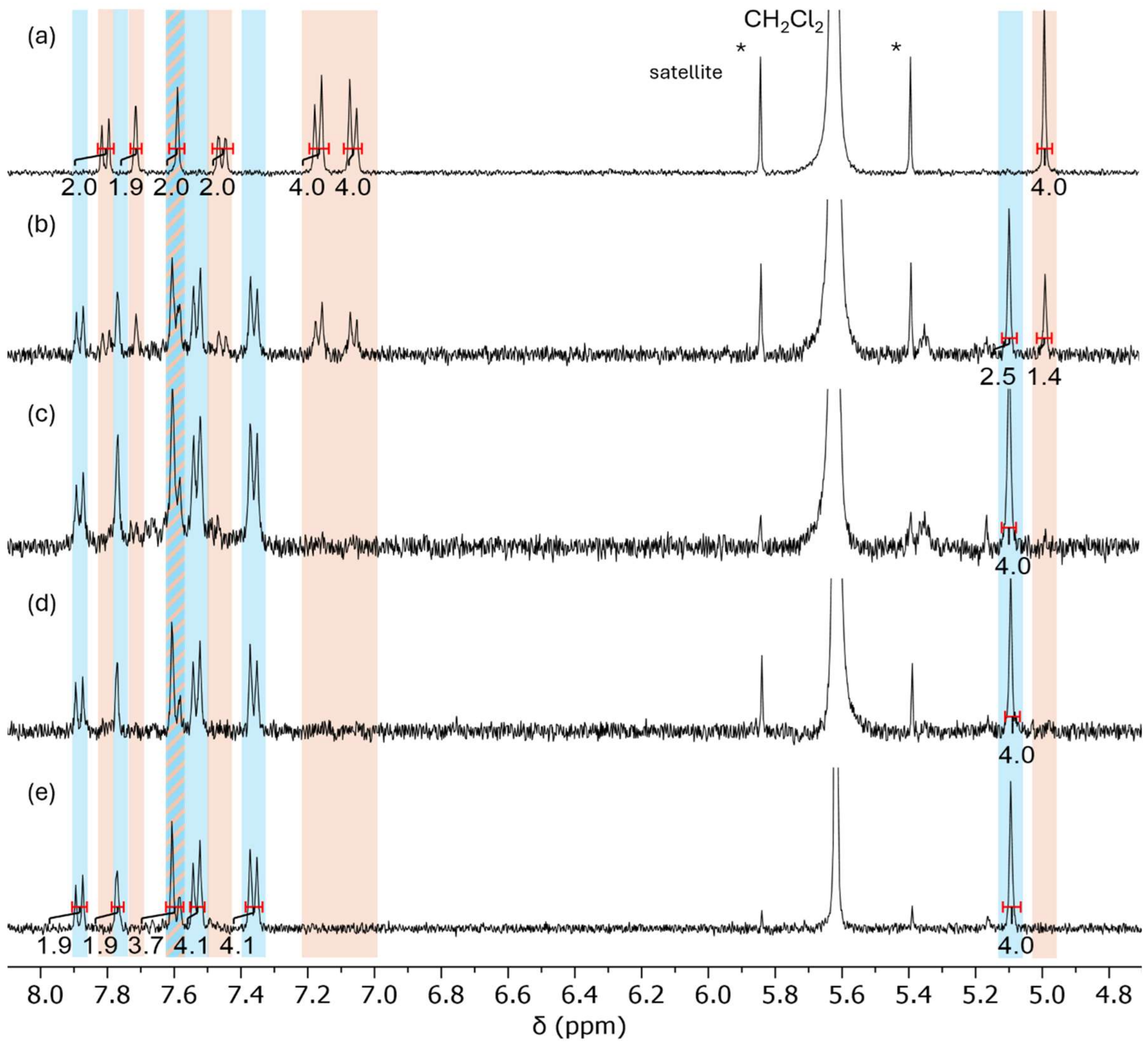


**Figure S16.** Partial $^{1}$H NMR spectra (400 MHz, Acetone-$d_6$) of **PMA3** (a), **PMA3** after 5min ultrasonication (b), **PMA3** after 45 min ultrasonication (c), **PMA3** after 60 min ultrasonication (d), and control polymer **PMA3ap** (e). The spectra provide direct evidence for the U/S-triggered conversion of the parallel mechanophore to its antiparallel diastereomers. Solutions of **PMA3** (2 mg/mL) were subjected to standard ultrasonication conditions with indicated time, concentrated, and precipitated into cold methanol to afford the ultrasonicated polymer sample for NMR analysis. The conversion at 5 min is around 63.7 %, which is consistent with the calculated conversion (69.8%) from the kinetic equation given in Figure S13. Orange shade indicates peaks assigned to **PMA2**, blue shade indicates peaks assigned to **PMA2ap**, and blue+orange shade indicates overlapped peaks.

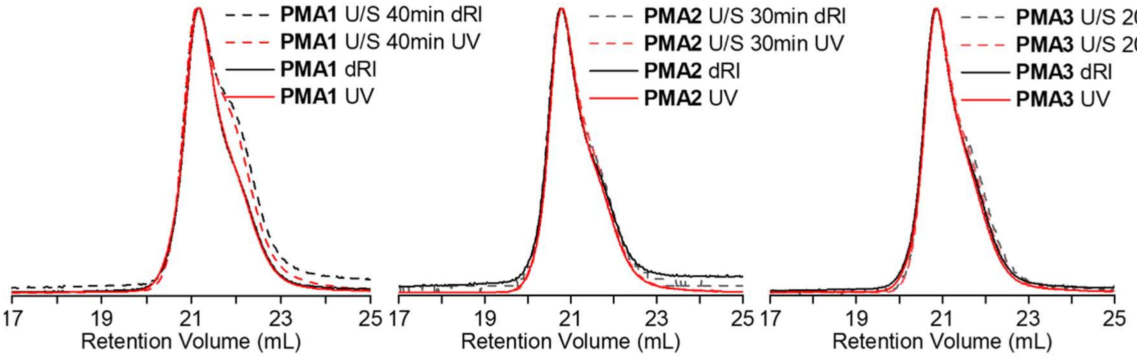


**Figure S17.** GPC chromatograms measured with differential refractive index (dRI) and UV-vis (monitored at 280 nm) detectors for sonicated **PMA1-PMA3.** Sonication timepoint at 40 min (**PMA1**), 30 min (**PMA2**) and 20 min (**PMA3**) were chosen, which are the approximate durations required to completely activate the mechanoresponsive polymers under our sonication conditions (see Figure S13).

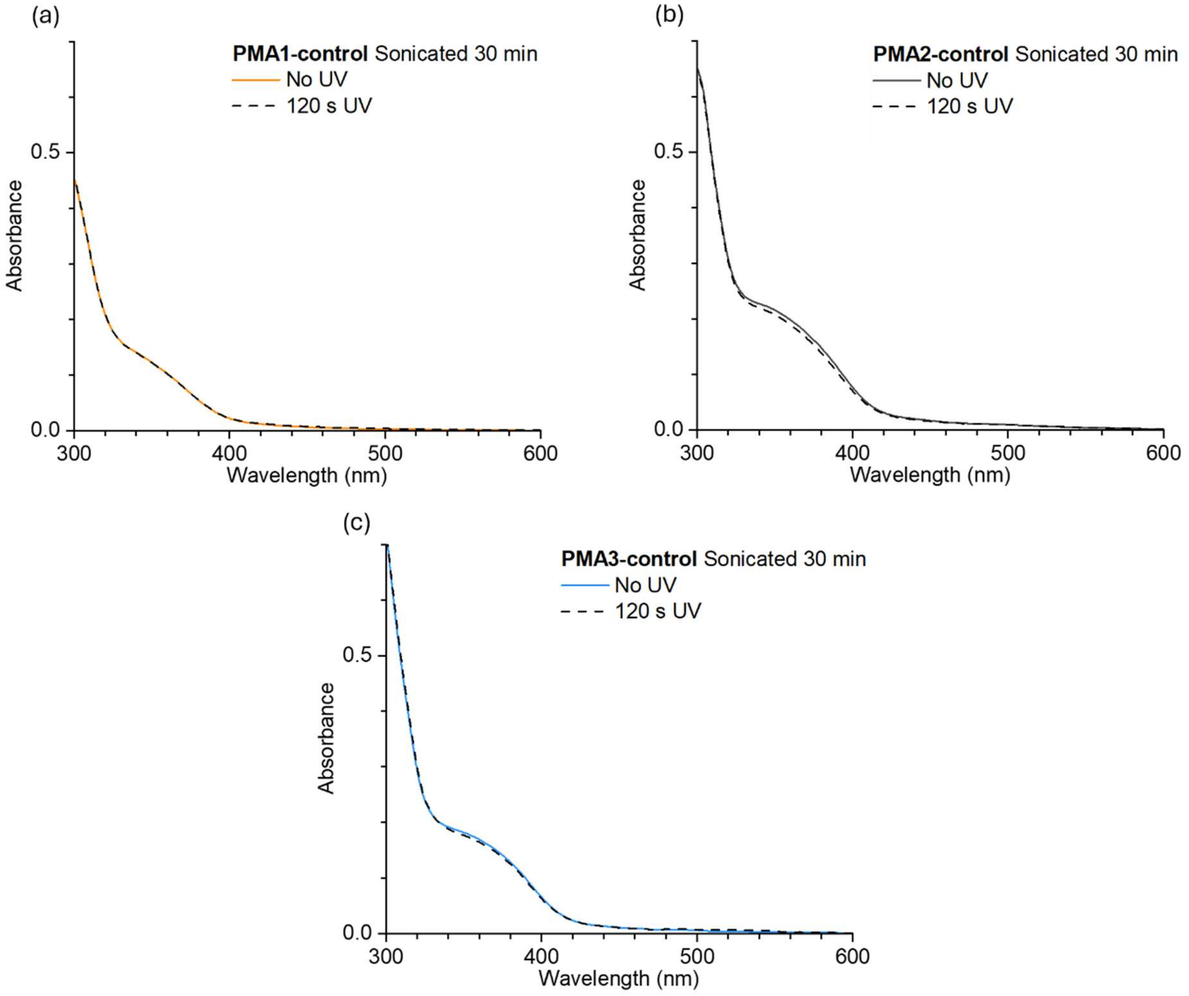


**Figure S18.** UV-vis absorption spectrum of sonicated solutions of chain-end control polymers (a) **PMA1-control**, (b) **PMA2-control**, and (c) **PMA3-control** before (solid curves) and after (dashed curves) UV irradiation. Sonication conditions: 2 mg/mL control polymers in acetonitrile, 30 min sonication. UV irradiation conditions: $\lambda$ = 365 nm, 120 s.

## 6. Investigation of the Thermal Stability of Atropisomeric Diarylethenes

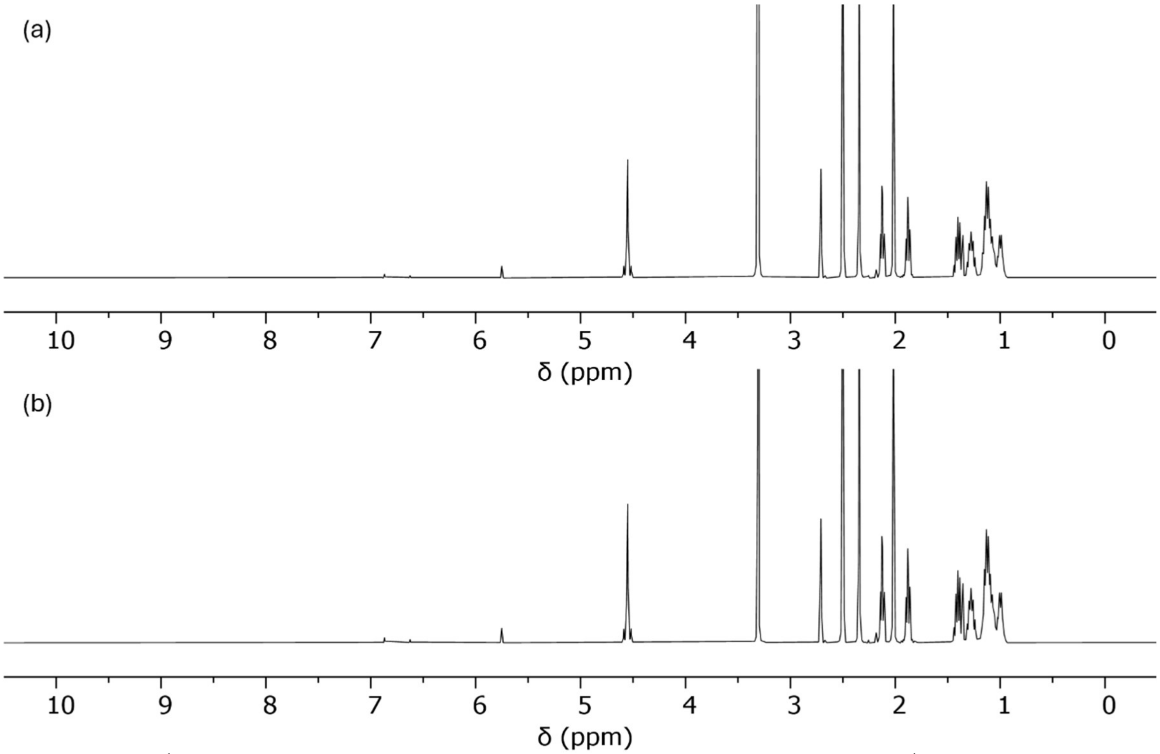


**Figure S19.** (a) $^1$H NMR spectrum (400 MHz, DMSO-$d_6$) of **1-yne-p**; (b) $^1$H NMR spectrum (400 MHz, DMSO-$d_6$) of **1-yne-p** after 100 °C heating for 12 h.

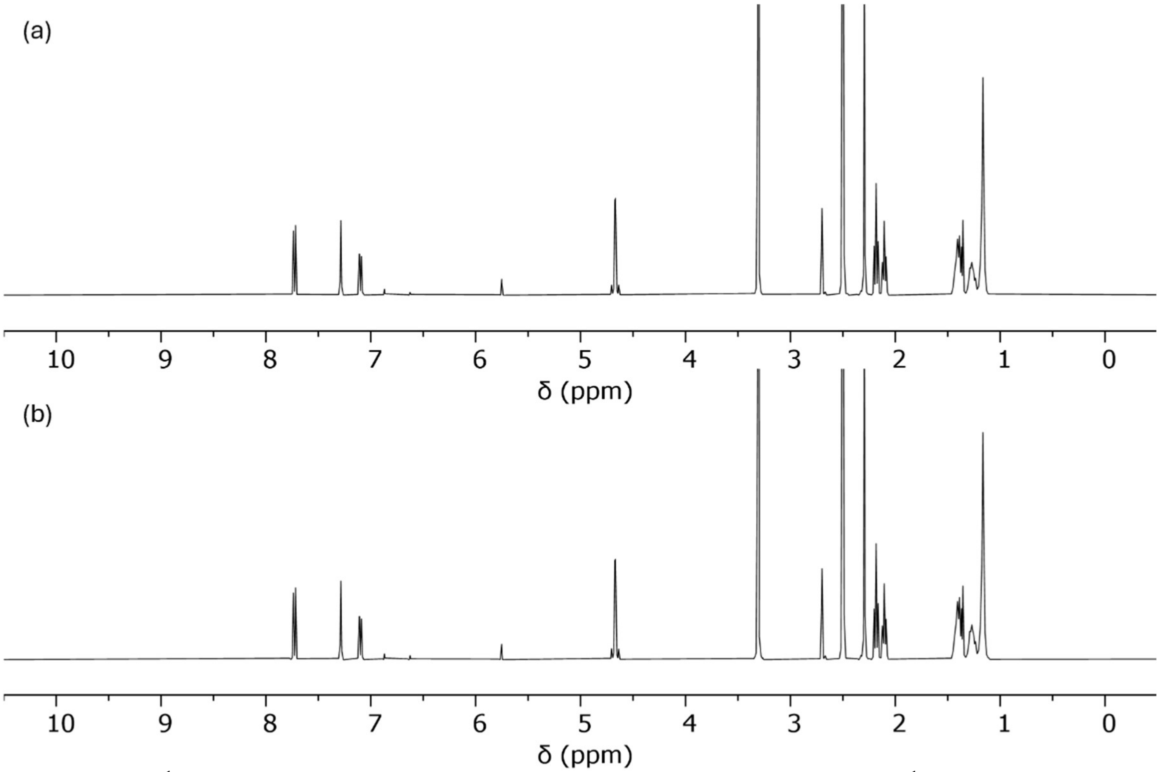


**Figure S20.** (a) $^1$H NMR spectrum (400 MHz, DMSO-$d_6$) of **2-yne-p**; (b) $^1$H NMR spectrum (400 MHz, DMSO-$d_6$) of **2-yne-p** after 100 °C heating for 12 h.

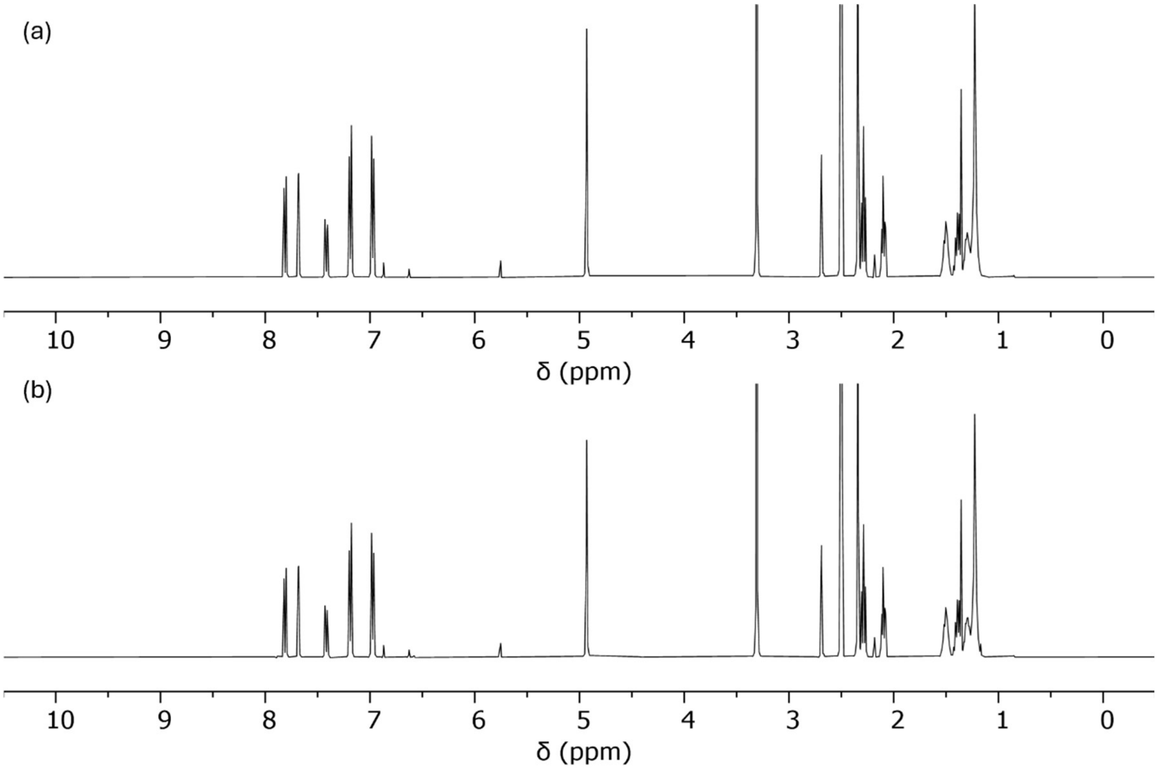


**Figure S21.** (a) $^1$H NMR spectrum (400 MHz, DMSO-$d_6$) of **3-yne-p**; (b) $^1$H NMR spectrum (400 MHz, DMSO-$d_6$) of **3-yne-p** after 100 °C heating for 12 h.

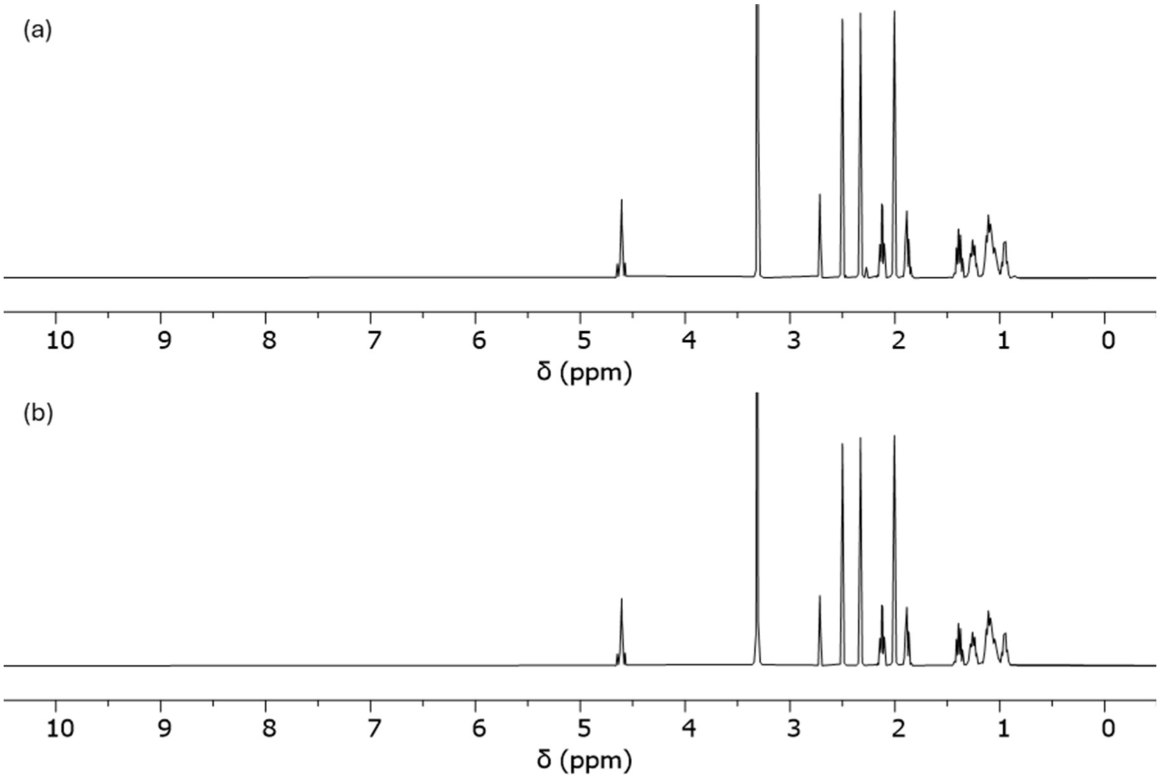


**Figure S22.** (a) $^1$H NMR spectrum (400 MHz, DMSO-$d_6$) of **(±)-1-yne-ap**; (b) $^1$H NMR spectrum (400 MHz, DMSO-$d_6$) of **(±)-1-yne-ap** after heating at 100 °C for 12 h.

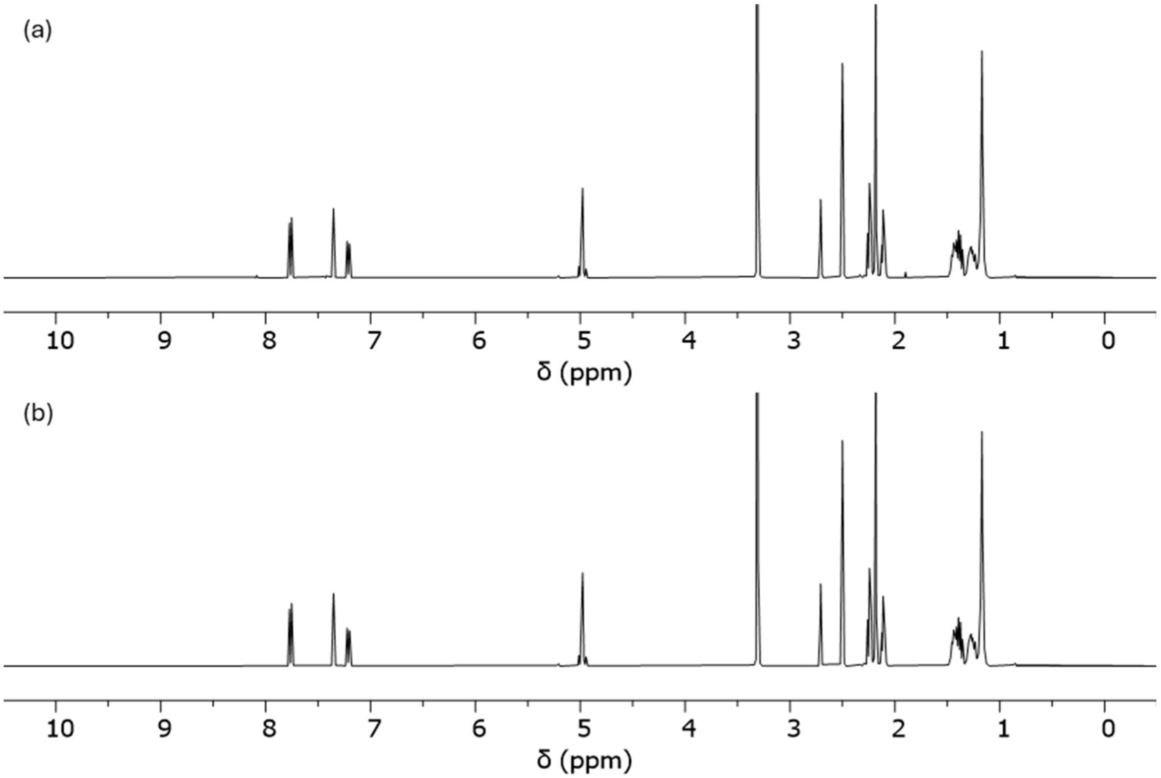


**Figure S23.** (a) $^{1}$H NMR spectrum (400 MHz, DMSO-$d_6$) of **(±)-2-yne-ap**; (b) $^{1}$H NMR spectrum (400 MHz, DMSO-$d_6$) of **(±)-2-yne-ap** after heating at 100 °C for 12 h.

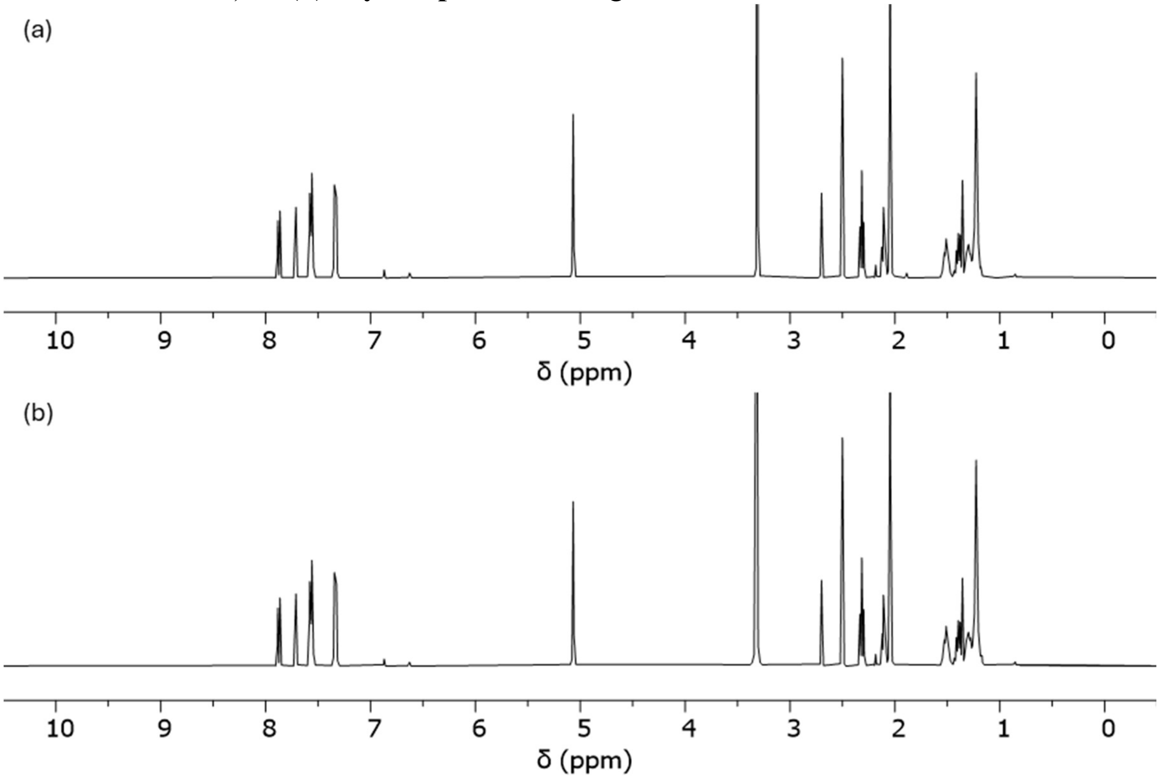


**Figure S24.** (a) $^{1}$H NMR spectrum (400 MHz, DMSO-$d_6$) of **(±)-3-yne-ap**; (b) $^{1}$H NMR spectrum (400 MHz, DMSO-$d_6$) of **(±)-3-yne-ap** after heating at 100 °C for 12 h.

## 7. Synthetic Details

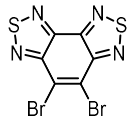


**BBT**

**4,5-Dibromobenzo[1,2-c:3,4-c′]bis[1,2,5]thiadiazole (BBT).** The procedure was optimized based on our previous study (*J. Am. Chem. Soc.* **2023**, *145*, 15668−15673). Benzothiadiazole (0.50 g, 2.6 mmol) was suspended in 48% w/w aq. HBr (10 mL) in a 20 mL pressure reaction vessel equipped with a stir bar. Under dark conditions, bromine (0.5 mL, 9.7 mmol) was added, and the mixture was stirred at 120 °C. After 48 h, the reaction was cooled to room temperature, and another 0.5 mL of bromine was added. The sealed reaction continued at 120 °C in the dark and the same bromine addition procedure was repeated two more times. The reaction was cooled down to room temperature followed by the addition of ice to form yellow solid precipitates, which were collected by filtration, washed with water, and washed multiple times with methanol to remove water. The resultant crude is a mixture of the monobromination and dibrominated products which was carefully recrystallized in methanol, affording the desirable dibrominated product BBT as a light-yellow solid (0.73 g, 80%). $^1$H NMR spectrum shows no proton signal. $^{13}$C NMR (100 MHz, $CDCl_3$) δ 155.03, 145.72, 122.46.

**Scheme S1.** Synthesis of the DAE Intermediate **8** in Isolated Atropisomeric Forms.

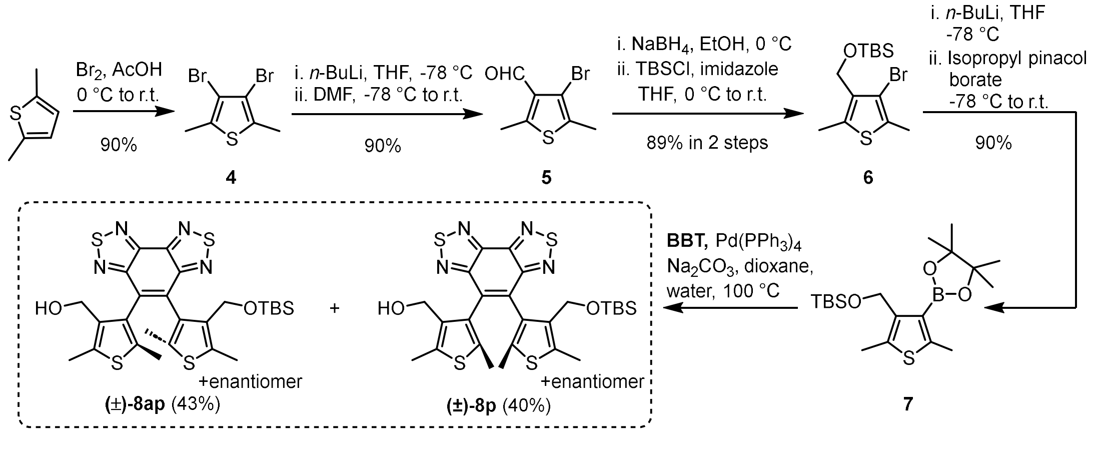


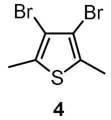


**4**

**Compound 4.** A 250 mL round bottom flask equipped with a stir bar was charged with 2,5-dimethylthiophene (3.0 g, 26.7 mmol) and AcOH (100 mL), cooled to 0 °C in an ice bath, followed by the addition of $Br_2$ (8.53 g, 2.7 mL, 53.4 mmol) dropwise in the dark. The reaction was allowed to warm up to room temperature and stirred overnight in the dark. The solution was poured into water (200 mL) and extracted with hexanes (200 mL). The organic phase was washed with water (200 mL), saturated $Na_2CO_3$ (100 mL) and saturated $NH_4Cl$ (200 mL), dried over $Na_2SO_4$, filtered,

and concentrated under reduced pressure. The crude product was purified by column chromatography (100% hexanes) to yield the title compound as a white solid (6.5 g, 90%). $R_f$ = 0.76 (100% hexanes). $^{1}H$ NMR (400 MHz, Chloroform-*d*) δ 2.43 (s, 6H). $^{13}C$ NMR (100 MHz, $CDCl_3$) δ 131.62, 111.84, 15.91. HRMS (ESI, m/z): calcd. for $[C_6H_6Br_2S]^+$ $M^+$, 267.85515; found, 267.85452.

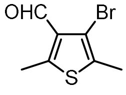


**5**

**Compound 5.** A 250 mL three-neck round bottom flask equipped with a stir bar was charged with **4** (2.5 g, 9.26 mmol) and anhydrous THF (120 mL). The solution was cooled to -78 °C in an acetone/dry ice bath, followed by the dropwise addition n-BuLi (4.1 mL, 2.5 M, 10.2 mmol). The mixture was retained at -78 °C for 1 h before adding DMF (1.1 mL, 1.03 g, 13.8 mmol). The reaction was allowed to warm up to room temperature and stirred overnight, then quenched with saturated $NH_4Cl$ (100 mL). The mixture was extracted with EtOAc (200 mL). The organic phase was washed with saturated $NH_4Cl$ (100 mL×2) and brine (100 mL), dried over $Na_2SO_4$, filtered, and concentrated under reduced pressure. The crude product was purified by column chromatography (0-10% EtOAc/hexanes) to yield the title compound as a colorless oil (1.83 g, 90%). $R_f$ = 0.67 (EtOAc:hexanes 1:5). $^{1}H$ NMR (400 MHz, Chloroform-*d*) δ 10.00 (s, 1H), 2.71 (s, 3H), 2.36 (s, 3H). $^{13}C$ NMR (100 MHz, $CDCl_3$) δ 187.41, 149.29, 131.49, 131.22, 110.88, 15.76, 14.31. HRMS (ESI, m/z): calcd. for $[C_7H_8OSBr]^+$ $(M+H)^+$, 218.94737; found, 218.94688.

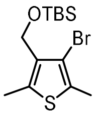


**6**

**Compound 6.** A 250 mL round bottom flask equipped with a stir bar was charged with **5** (1.64 g, 7.5 mmol) and ethanol (50 mL). At 0 °C, $NaBH_4$ (426 mg, 11.25 mmol) was added in several portions, and the mixture was stirred for 1 h. Saturated $NH_4Cl$ (20 mL) was added to the mixture dropwise to quench the reaction at 0 °C. The mixture was extracted with EtOAc (150 mL). The organic phase was washed with saturated $NH_4Cl$ (100 mL×2) and brine (100 mL), dried over $Na_2SO_4$, filtered, and concentrated under reduced pressure. The crude product was directly used for the next step reaction.

A 250 mL round bottom flask equipped with a stir bar was charged with the crude product, imidazole (606 mg, 8.9 mmol) and anhydrous THF (100 mL). The solution was cooled to 0 °C in an ice bath, followed by the addition of a solution of TBSCl (1.34 g, 8.9 mmol) in THF (5 mL). The reaction was allowed to warm up to room temperature and stirred overnight. The reaction was quenched with water (50 mL) and extracted with $Et_2O$ (100 mL). The organic phase was washed with saturated $NH_4Cl$ (100 mL), saturated $NaHCO_3$ (100 mL) and brine (100 mL), dried over $Na_2SO_4$, filtered, and concentrated under reduced pressure. The crude product was purified by column chromatography (0-5% EtOAc/hexanes) to yield the title compound as a colorless oil (2.24 g, 89% in two steps). $R_f$ = 0.30 (100% hexanes). $^{1}H$ NMR (400 MHz, Chloroform-*d*) δ 4.58 (s,

2H), 2.43 (s, 3H), 2.33 (s, 3H), 0.92 (s, 9H), 0.10 (s, 6H). $^{13}$C NMR (100 MHz, $CDCl_3$) δ 135.06, 134.36, 129.99, 111.18, 58.89, 26.13, 18.62, 14.91, 13.93, -5.08. HRMS (ESI, m/z): calcd. for $[C_{13}H_{27}ONBrSSi]^+$ $(M+NH_4)^+$, 352.07605; found, 352.07503.

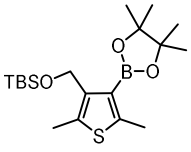


**7**

**Compound 7.** A 250 mL round bottom flask equipped with a stir bar was charged with **6** (4.8 g, 14.3 mmol) and anhydrous THF (150 mL). The solution was cooled to -78 °C in an acetone/dry ice bath, followed by the dropwise addition n-BuLi (6.3 mL, 2.5 M, 15.8 mmol). The mixture was retained at −78 °C for 1 h before adding 2-isopropoxy-4,4,5,5-tetramethyl-1,3,2-dioxaborolane (3.2 g, 17.3 mmol). The reaction was allowed to warm up to room temperature and stirred overnight, then quenched with saturated $NH_4Cl$ (50 mL). The mixture was extracted with $Et_2O$ (100 mL). The organic phase was washed with saturated $NH_4Cl$ (100 mL), saturated $NaHCO_3$ (100 mL) and brine (100 mL), dried over $Na_2SO_4$, filtered, and concentrated under reduced pressure. The crude product was purified by column chromatography (0-10% EtOAc/hexanes, 1% dichloromethane (DCM) constant additive) to yield the title compound as a colorless oil (4.96 g, 90%). $R_f$ = 0.36 (EtOAc:hexanes 5:95). $^1$H NMR (400 MHz, Chloroform-*d*) δ 4.77 (s, 2H), 2.57 (s, 3H), 2.37 (s, 3H), 1.31 (s, 12H), 0.91 (s, 9H), 0.06 (s, 6H). $^{13}$C NMR (100 MHz, $CDCl_3$) δ 148.49, 141.77, 134.36, 82.95, 58.62, 26.25, 25.07, 18.73, 15.93, 12.89, -4.99. HRMS (ESI, m/z): calcd. for $[C_{19}H_{36}O_3BSSi]^+$ $(M+H)^+$, 383.22420; found, 383.22302.

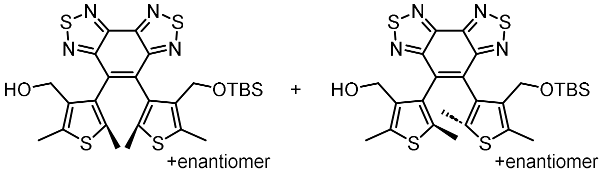


(±)-8p (±)-8ap

**Compound 8.** A 100 mL three-neck round bottom flask equipped with a stir bar and a condenser was charged with **7** (730 mg, 1.91 mmol) and **BBT** (169 mg, 0.48 mmol), 1,4-dioxane (20 mL) and 2 M $Na_2CO_3$ aqueous solution (4 mL). The mixed solution was purged with $N_2$ for 15 min. $Pd(PPh_3)_4$ (83 mg, 0.07 mmol) was added and followed by another 15 min purging. The mixture was heated to 100 °C in the dark for 72 h. Then the mixture was extracted with EtOAc (100 mL). The organic phase was washed with saturated $NH_4Cl$ (50 mL×3), dried over $Na_2SO_4$, filtered, and concentrated under reduced pressure. The crude product was purified by column chromatography (10-30% EtOAc/hexanes) to yield compound **(±)-8p** as a yellow solid (450 mg, 40%) and compound **(±)-8ap** as a red solid (484 mg, 43%).

**(±)-8p**: $R_f$ = 0.76 (EtOAc:hexanes 1:2). $^1$H NMR (400 MHz, Chloroform-*d*) δ 4.27 – 4.04 (m, 4H), 2.49 (s, 4H), 2.36 (s, 3H), 2.04 (s, 3H), 1.97 (s, 3H), 0.49 (s, 9H), -0.23 (s, 3H), -0.42 (s, 3H). $^{13}$C NMR (100 MHz, $CDCl_3$) δ 157.89, 157.46, 147.31, 147.25, 136.97, 136.22, 135.45, 135.36,

134.89, 132.68, 131.59, 131.44, 131.07, 58.67, 57.63, 25.46, 17.96, 15.10, 14.79, 13.60, 13.52, -5.51, -5.70. HRMS (ESI, m/z): calcd. for $[C_{26}H_{36}N_5O_2S_4Si]^+$ $(M+NH_4)^+$, 606.15156; found, 606.15129.

**(±)-8ap** $R_f$ = 0.56 (EtOAc:hexanes 1:2). $^1$H NMR (400 MHz, Chloroform-*d*) δ 4.35 – 4.00 (m, 4H), 2.50 (s, 3H), 2.40 (s, 3H), 2.00 (s, 3H), 1.89 (s, 3H), 0.58 (s, 9H), -0.24 (s, 3H), -0.36 (s, 3H). $^{13}$C NMR (101 MHz, $CDCl_3$) δ 157.96, 157.66, 147.48, 147.21, 136.35, 136.21, 136.12, 134.95, 134.08, 133.46, 133.17, 131.35, 131.19, 131.13, 59.18, 57.91, 25.67, 18.10, 14.87, 14.55, 13.88, 13.49, -5.70, -5.76. HRMS (ESI, m/z): calcd. for $[C_{26}H_{36}N_5O_2S_4Si]^+$ $(M+NH_4)^+$, 606.15156; found, 606.15068.

**Scheme S2.** Synthesis of Materials Containing Parallel Mechanophore **M1**.

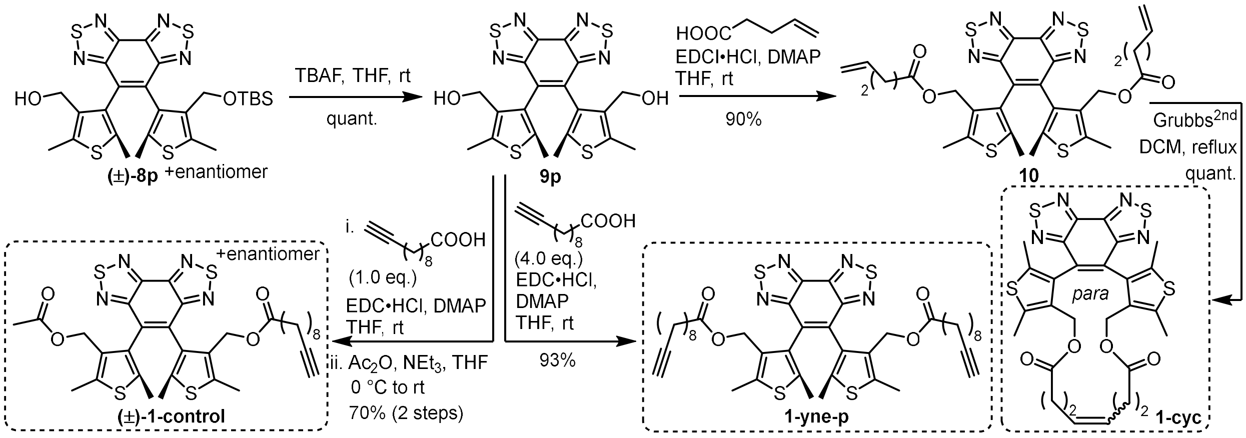


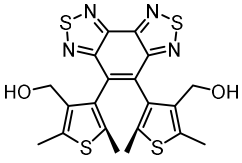

9p

**Compound 9p.** A 20 mL vial equipped with a stir bar was charged with **(±)-8p** (450 mg, 0.76 mmol) and THF (5 mL). 1 mL of 1 M TBAF in THF was added dropwise and the reaction was stirred at room temperature for 2 h. The mixture was extracted with EtOAc (30 mL). The organic phase was washed with saturated $NH_4Cl$ (10 mL×3), dried over $Na_2SO_4$, filtered, and concentrated under reduced pressure. The crude product was purified by column chromatography (10-30% EtOAc/DCM) to yield the title compound as a yellow solid (362 mg, quant.). $R_f$ = 0.31 (EtOAc:DCM 1:5). $^1$H NMR (400 MHz, DMSO-$d_6$) δ 4.41 (t, $J$ = 5.3 Hz, 2H), 4.02 (ABq(d), $\Delta\nu_{AB}$ = 13.3 Hz, $J_{AB}$ = 13.0 Hz, $J$ = 5.7 Hz, 4H), 2.35 (s, 6H), 1.96 (s, 6H). $^{13}$C NMR (100 MHz, DMSO-$d_6$) δ 157.31, 147.02, 137.59, 133.50, 132.05, 132.03, 131.27, 56.28, 14.38, 13.20. HRMS (ESI, m/z): calcd. for $[C_{20}H_{22}N_5O_2S_4]^+$ $(M+NH_4)^+$, 492.06508; found, 492.06546.

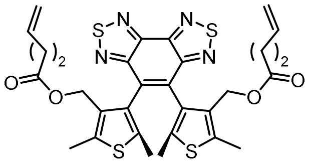

10

**Compound 10.** A 20 mL vial equipped with a stir bar was charged with **9p** (120 mg, 0.253 mmol) and THF (10 mL). EDC·HCl (145 mg, 0.76 mmol), 4-pentenoic acid (76 mg, 0.76 mmol) and DMAP (7 mg, 0.05 mmol) were added respectively. The stirring reaction was allowed to react for 16 h, then quenched with saturated $NH_4Cl$ (20 mL). The mixture was extracted with EtOAc (100 mL). The organic phase was washed with saturated $Na_2CO_3$ (30 mL×2), saturated $NH_4Cl$ (30 mL) and brine (30 mL), dried over $Na_2SO_4$, filtered, and concentrated under reduced pressure. The crude product was purified by column chromatography (10-25% EtOAc/hexanes, 10% DCM constant additive) to yield the title compound as a pale-yellow solid (145 mg, 90%). $R_f$ = 0.45 (EtOAc:hexanes 1:5). $^1$H NMR (400 MHz, Chloroform-*d*) δ 5.70 – 5.55 (m, 2H), 4.95 – 4.85 (m, 4H), 4.62 (ABq, $\Delta\nu_{AB}$ = 27.6 Hz, *J* = 12.8 Hz, 4H), 2.42 (s, 6H), 2.13 – 2.07 (m, 8H), 2.04 (s, 6H). $^{13}$C NMR (100 MHz, $CDCl_3$) δ 172.43, 157.43, 147.39, 136.76, 136.56, 135.44, 131.71, 131.68, 131.15, 115.53, 59.11, 33.16, 28.62, 14.93, 13.76. HRMS (ESI, m/z): calcd. for $[C_{30}H_{34}N_5O_4S_4]^+$ $(M+NH_4)^+$, 656.14881; found, 656.14738.

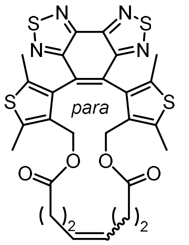

1-cyc

**Compound 1-cyc.** A 250 mL three-neck round bottom flask equipped with a stir bar was charged with **10** (100 mg, 0.16 mmol) and DCM (150 mL) and purged with $N_2$ for 30 min. Then Grubbs II catalyst (8.5 mg, 0.01 mmol) was added, and the mixture was purged for another 30 min. The stirring reaction was heated to reflux and allowed to react for 16 h. Then 1 mL ethyl vinyl ether was added, and the reaction was kept refluxing for 1 h. The solution was cooled to room temperature and concentrated under reduced pressure. The crude product was purified by column chromatography (10-25% EtOAc/hexanes, 10% DCM constant additive) to yield the title compound as a yellow solid (95 mg, quant.). The product consisted of a 1:20 (E:Z) isomeric mixture, which was used directly in the next step. $R_f$ = 0.70 (EtOAc:hexanes 1:2). $^1$H NMR (400 MHz, Chloroform-*d*) δ 5.22 – 5.17 (m, 2H), 4.93 (d, *J* = 12.9 Hz, 0.1H), 4.78 (d, *J* = 13.0 Hz, 2H), 4.52 (d, *J* = 12.9 Hz, 0.1H), 4.46 (d, *J* = 13.0 Hz, 2H), 2.44 (s, 6H), 2.17 – 1.91 (m, 8H). $^{13}$C NMR (100 MHz, $CDCl_3$) δ 172.47, 157.70, 147.41, 136.70, 135.17, 132.00, 131.63, 131.55, 129.93, 59.32, 34.07, 27.72, 14.90, 13.82. HRMS (ESI, m/z): calcd. for $[C_{28}H_{27}N_4O_4S_4]^+$ $(M+H)^+$, 611.09096; found, 611.09010.

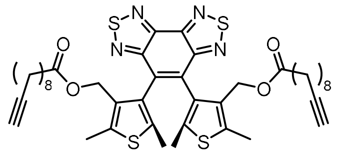

1-yne-p

**Compound 1-yne-p.** A 20 mL vial equipped with a stir bar was charged with **9p** (70 mg, 0.147 mmol) and THF (5 mL). EDC·HCl (113 mg, 0.588 mmol), undec-10-ynoic acid (107 mg, 0.588 mmol) and DMAP (4 mg 0.03 mmol) were added respectively. The stirring reaction was allowed to react for 16 h, then quenched with saturated $NH_4Cl$ (20 mL). The mixture was extracted with EtOAc (30 mL). The organic phase was washed with saturated $Na_2CO_3$ (30 mL×2), saturated $NH_4Cl$ (30 mL) and brine (30 mL), dried over $Na_2SO_4$, filtered, and concentrated under reduced pressure. The crude product was purified by column chromatography (10-25% EtOAc/hexanes, 10% DCM constant additive) to yield the title compound as a pale-yellow solid (110 mg, 93%). $R_f$ = 0.39 (EtOAc:hexanes 1:5). $^1$H NMR (400 MHz, Chloroform-*d*) δ 4.58 (ABq, $\Delta\nu_{AB}$ = 39.5 Hz, *J* = 12.8 Hz, 4H), 2.42 (s, 6H), 2.16 (td, *J* = 7.1, 2.6 Hz, 4H), 2.04 (s, 6H), 2.00 – 1.91 (m, 6H), 1.54 – 1.44 (m, 4H), 1.41 – 1.27 (m, 8H), 1.27 – 1.07 (m, 12H). $^{13}$C NMR (100 MHz, $CDCl_3$) δ 173.18, 157.46, 147.40, 136.66, 135.41, 131.72, 131.29, 84.86, 68.25, 58.99, 33.97, 29.17, 29.14, 29.00, 28.78, 28.56, 24.77, 18.51, 14.94, 13.76. HRMS (ESI, m/z): calcd. for $[C_{42}H_{54}N_5O_4S_4]^+$ $(M+NH_4)^+$, 820.30531; found, 820.30358.

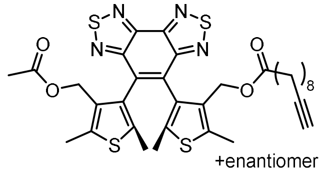

(±)-1-control

**Compound (±)-1-control.** A 20 mL vial equipped with a stir bar was charged with **9p** (70 mg, 0.147 mmol) and THF (5 mL). EDC·HCl (28 mg, 0.147 mmol), undec-10-ynoic acid (27 mg, 0.147 mmol) and DMAP (4 mg 0.03 mmol) were added respectively. The stirring reaction was allowed to react for 16 h, then quenched with saturated $NH_4Cl$ (20 mL). The mixture was extracted with EtOAc (30 mL). The organic phase was washed with saturated $Na_2CO_3$ (30 mL×2), saturated $NH_4Cl$ (30 mL) and brine (30 mL), dried over $Na_2SO_4$, filtered, and concentrated under reduced pressure. The crude product was not purified and directly used for the next reaction.

A 20 mL vial equipped with a stir bar was charged with the crude and anhydrous THF (5 mL). The solution was cooled to 0 °C in an ice bath, followed by the addition of triethylamine (0.5 mL). Acetic anhydride (0.5 mL) was added dropwise to the mixture. The stirring reaction was warmed up to room temperature overnight and quenched by saturated $NH_4Cl$ (10 mL). The mixture was extracted with EtOAc (30 mL). The organic phase was washed with saturated $Na_2CO_3$ (30 mL×2), saturated $NH_4Cl$ (30 mL) and brine (30 mL), dried over $Na_2SO_4$, filtered, and concentrated under reduced pressure. The crude product was purified by column chromatography (10-25% EtOAc/hexanes, 10% DCM constant additive) to yield the title compound as a yellow solid (70 mg, 70%). $R_f$ = 0.21 (EtOAc:hexanes 1:5). $^1$H NMR (400 MHz, Chloroform-*d*) δ 4.62 – 4.42 (m, 4H), 2.36 (s, 3H), 2.35 (s, 3H), 2.10 (td, *J* = 7.1, 2.6 Hz, 2H), 1.97 (s, 6H), 1.95 – 1.79 (m, 3H),

1.69 (s, 3H), 1.42 (p, $J$ = 7.1 Hz, 2H), 1.30 – 1.10 (m, 10H). $^{13}$C NMR (100 MHz, $CDCl_3$) δ 173.23, 170.48, 157.47, 147.41, 136.89, 136.62, 135.44, 131.84, 131.74, 131.66, 131.27, 131.17, 84.87, 68.25, 59.15, 59.00, 33.97, 29.14, 29.12, 28.98, 28.77, 28.56, 24.76, 20.62, 18.51, 14.95, 14.93, 13.75. HRMS (ESI, m/z): calcd. for $[C_{33}H_{40}N_5O_4S_4]^+$ $(M+NH_4)^+$, 698.19576; found, 698.19437.

**Scheme S3.** Synthesis of a **(±)-1-yne-ap** Containing the Photoactive Antiparallel DAE.

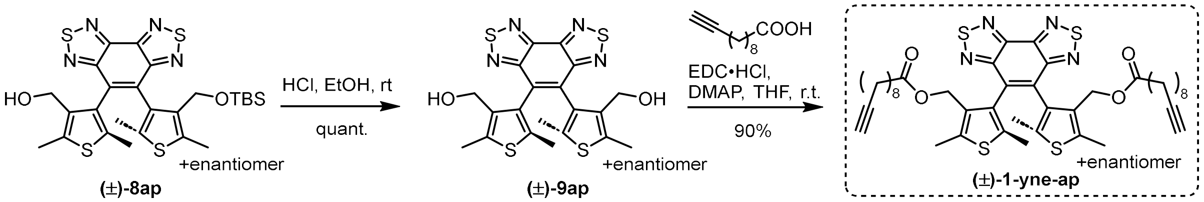


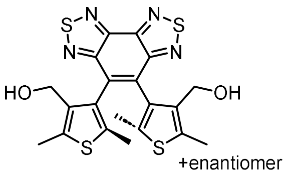


(±)-9ap

**Compound (±)-9ap.** A 20 mL vial equipped with a stir bar was charged with **(±)-8ap** (370 mg, 0.63 mmol) and THF (5 mL). 1 mL of 1 M TBAF in THF was added dropwise and the reaction was stirred at room temperature for 2 h. The mixture was extracted with EtOAc (30 mL). The organic phase was washed with saturated $NH_4Cl$ (10 mL×3), dried over $Na_2SO_4$, filtered, and concentrated under reduced pressure. The crude product was purified by column chromatography (10-30% EtOAc/DCM) to yield the title compound as a red solid (298 mg, quant.). $R_f$ = 0.52 (EtOAc:DCM 1:5). $^1$H NMR (400 MHz, DMSO-$d_6$) δ 4.27 (t, $J$ = 5.2 Hz, 2H), 4.01 (ABq(d), $\Delta\nu_{AB}$ = 21.9 Hz, $J_{AB}$ = 12.6 Hz, $J$ = 4.8 Hz, 4H), 2.34 (s, 6H), 1.96 (s, 6H). $^{13}$C NMR (100 MHz, DMSO-$d_6$) δ 157.33, 147.01, 137.64, 133.08, 132.00, 131.92, 131.39, 56.47, 14.36, 13.24. HRMS (ESI, m/z): calcd. for $[C_{20}H_{22}N_5O_2S_4]^+$ $(M+NH_4)^+$, 492.06508; found, 492.06451.

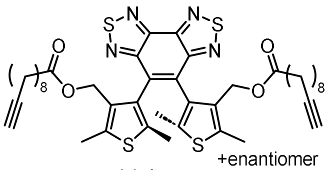


(±)-1-yne-ap

**Compound (±)-1-yne-ap.** A 7 mL vial equipped with a stir bar was charged with **(±)-9ap** (40 mg, 0.084 mmol) and THF (3.5 mL). EDC·HCl (65 mg, 0.337 mmol), undec-10-ynoic acid (67 mg, 0.337 mmol) and DMAP (4 mg 0.03 mmol) were added respectively. The stirring reaction was allowed to react for 16 h, then quenched with saturated $NH_4Cl$ (20 mL). The mixture was extracted with EtOAc (30 mL). The organic phase was washed with saturated $Na_2CO_3$ (30 mL×2), saturated $NH_4Cl$ (30 mL) and brine (30 mL), dried over $Na_2SO_4$, filtered, and concentrated under reduced pressure. The crude product was purified by column chromatography

(10-25% EtOAc/hexanes, 10% DCM constant additive) to yield the title compound as a red solid (61 mg, 90%). $R_f$ = 0.52 (EtOAc:hexanes 1:5). $^1$H NMR (400 MHz, Chloroform-*d*) δ 4.61 (ABq, $\Delta\nu_{AB}$ = 8.7 Hz, *J* = 12.5 Hz, 4H), 2.40 (s, 6H), 2.16 (td, *J* = 7.1, 2.6 Hz, 4H), 2.07 (s, 6H), 2.05 – 1.87 (m, 6H), 1.55 – 1.43 (m, 4H), 1.38 – 1.24 (m, 8H), 1.23 – 1.04 (m, 12H). $^{13}$C NMR (101 MHz, $CDCl_3$) δ 173.46, 157.40, 147.44, 136.74, 135.20, 131.77, 131.69, 131.23, 84.85, 68.27, 59.18, 34.06, 29.12, 29.09, 28.98, 28.75, 28.54, 24.79, 18.51, 14.97, 13.73. HRMS (ESI, m/z): calcd. for $[C_{42}H_{54}N_5O_4S_4]^+$ $(M+NH_4)^+$, 820.30531; found, 820.30450.

**Scheme S4.** Synthesis of Materials Containing Mechanophore **M2**.

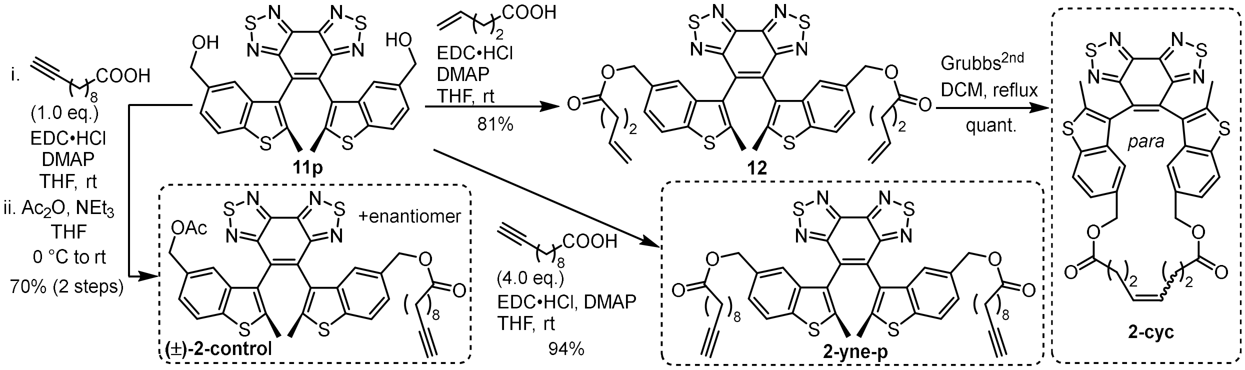


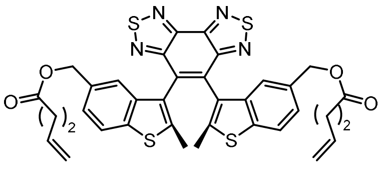

**12**

**Compound 12.** A 100 mL round bottom flask equipped with a stir bar was charged with **11p** (730 mg, 1.34 mmol) (reported in *J. Am. Chem. Soc.* **2023**, *145*, 15668−15673) and THF (30 mL). EDC·HCl (613 mg, 3.2 mmol), 4-pentenoic acid (400 mg, 4.0 mmol) and DMAP (33 mg, 0.27 mmol) were added respectively. The stirring reaction was allowed to react for 16 h, then quenched with saturated $NH_4Cl$ (20 mL). The mixture was extracted with EtOAc (100 mL). The organic phase was washed with saturated $Na_2CO_3$ (30 mL×2), saturated $NH_4Cl$ (30 mL) and brine (30 mL), dried over $Na_2SO_4$, filtered, and concentrated under reduced pressure. The crude product was purified by column chromatography (5-20% EtOAc/hexanes, 10% DCM constant additive) to yield the title compound as a yellow solid (772 mg, 81%). $R_f$ = 0.73 (EtOAc:hexanes 1:2). $^1$H NMR (400 MHz, Chloroform-*d*) δ 7.61 (d, *J* = 8.2 Hz, 2H), 7.14 (dd, *J* = 8.3, 1.6 Hz, 2H), 7.04 (d, *J* = 1.7 Hz, 2H), 5.73 (ddt, *J* = 16.7, 10.5, 6.2 Hz, 2H), 5.02 – 4.88 (m, 4H), 4.70 (s, 4H), 2.36 – 2.22 (m, 14H). $^{13}$C NMR (100 MHz, $CDCl_3$) δ 172.70, 156.86, 147.89, 141.56, 139.14, 137.99, 136.71, 132.13, 130.98, 127.24, 124.46, 122.71, 122.22, 115.63, 66.03, 33.56, 28.88, 15.91. HRMS (ESI, m/z): calcd. for $[C_{36}H_{34}N_5O_4S_4]^+$ $(M+NH_4)^+$, 728.14881; found, 728.14763.

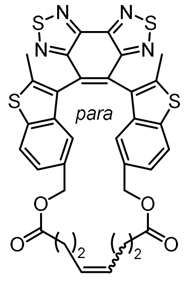

**2-cyc**

**Compound 2-cyc.** A 250 mL three-neck round bottom flask equipped with a stir bar was charged with **12** (148 mg, 0.21 mmol) and DCM (150 mL) and purged with $N_2$ for 30 min. Then Grubbs II catalyst (8.5 mg, 10 μmol) was added, and the mixture was purged with $N_2$ for another 30 min. The stirring reaction was heated to reflux and allowed to react for 16 h. Then 1 mL ethyl vinyl ether was added, and the reaction was kept refluxing for 1 h. The solution was cooled to room temperature and concentrated under reduced pressure. The crude product was purified by column chromatography (5-20% EtOAc/hexanes, 10% DCM constant additive) to yield the title compound as a yellow solid (141 mg, quant.). The product consisted of a 1:4.6 (E:Z) isomeric mixture, which was used directly in the next step. $R_f$ = 0.18 (EtOAc:hexanes 1:5) $^1$H NMR (400 MHz, Chloroform-*d*) δ 7.62 (d, *J* = 8.4 Hz, 2H), 7.17 (s, 1.1H), 7.14 (s, 2.5H), 7.07 (s, 0.3H), 5.46 – 5.37 (m, 1.6H), 5.37 – 5.32 (m, 0.4H), 4.87 – 4.59 (m, 4H), 2.36 – 2.16 (m, 14H). $^{13}$C NMR (100 MHz, $CDCl_3$) δ 172.80, 156.87, 147.92, 141.45, 139.03, 137.87, 132.24, 130.98, 129.79, 127.32, 124.42, 122.95, 122.23, 65.97, 34.54, 27.86, 15.89. HRMS (ESI, m/z): calcd. for $[C_{34}H_{30}N_5O_4S_4]^+$ $(M+NH_4)^+$, 700.11751; found, 700.11632.

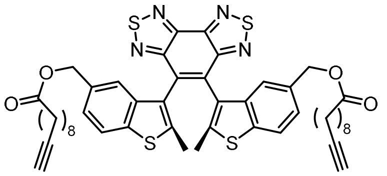

**2-yne-p**

**Compound 2-yne-p.** A 20 mL vial equipped with a stir bar was charged with **11p** (70 mg, 0.128 mmol) and THF (5 mL). EDC·HCl (98 mg, 0.512 mmol), undec-10-ynoic acid (93 mg, 0.512 mmol) and DMAP (0.9 mg 7 μmol) were added respectively. The stirring reaction was allowed to react for 16 h, then quenched with saturated $NH_4Cl$ (20 mL). The mixture was extracted with EtOAc (30 mL). The organic phase was washed with saturated $Na_2CO_3$ (30 mL×2), saturated $NH_4Cl$ (30 mL) and brine (30 mL), dried over $Na_2SO_4$, filtered, and concentrated under reduced pressure. The crude product was purified by column chromatography (10-25% EtOAc/hexanes, 10% DCM constant additive) to yield the title compound as a yellow solid (105 mg, 94%). $R_f$ = 0.76 (EtOAc:hexanes 1:2). $^1$H NMR (400 MHz, Chloroform-*d*) δ 7.61 (d, *J* = 8.2 Hz, 2H), 7.14 (dd, *J* = 8.3, 1.6 Hz, 2H), 7.04 (d, *J* = 1.6 Hz, 2H), 4.69 (s, 4H), 2.28 (s, 6H), 2.23 – 2.12 (m, 8H), 1.92 (t, *J* = 2.7 Hz, 2H), 1.58 – 1.43 (m, 8H), 1.40 – 1.29 (m, 4H), 1.28-1.19 (m, 12H). $^{13}$C NMR (101 MHz, $CDCl_3$) δ 173.43, 156.83, 147.85, 141.50, 139.09, 137.90, 132.22, 130.95, 127.21, 124.41, 122.69, 122.18, 84.84, 68.25, 65.83, 34.28, 29.20, 29.15, 28.99, 28.76, 28.54, 24.93, 18.48, 15.89. HRMS (ESI, m/z): calcd. for $[C_{48}H_{54}N_5O_4S_4]^+$ $(M+NH_4)^+$, 892.30531; found, 892.30384.

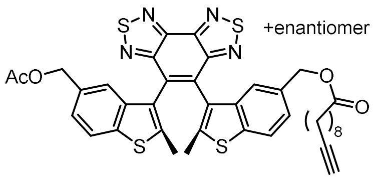


**(±)-2-control**

**Compound (±)-2-control.** A 20 mL vial equipped with a stir bar was charged with **11p** (70 mg, 0.128 mmol) and THF (5 mL). EDC·HCl (25 mg, 0.128 mmol), undec-10-ynoic acid (24 mg, 0.128 mmol) and DMAP (3 mg 0.026 mmol) were added respectively. The stirring reaction was allowed to react for 16 h, then quenched with saturated $NH_4Cl$ (20 mL). The mixture was extracted with EtOAc (30 mL). The organic phase was washed with saturated $Na_2CO_3$ (30 mL×2), saturated $NH_4Cl$ (30 mL) and brine (30 mL), dried over $Na_2SO_4$, filtered, and concentrated under reduced pressure. The crude product was not purified and directly used for the next reaction.

A 20 mL vial equipped with a stir bar was charged with the crude and anhydrous THF (5 mL). The solution was cooled to 0 °C in an ice bath, followed by the addition of triethylamine (0.5 mL). Acetic anhydride (0.5 mL) was added dropwise to the mixture. The stirring reaction was warmed up to room temperature overnight. The reaction was quenched by saturated $NH_4Cl$ (10 mL). The mixture was extracted with EtOAc (30 mL). The organic phase was washed with saturated $Na_2CO_3$ aqueous solution (30 mL×2), saturated $NH_4Cl$ (30 mL) and brine (30 mL), dried over $Na_2SO_4$, filtered, and concentrated under reduced pressure. The crude product was purified by column chromatography (10-25% EtOAc/hexanes, 10% DCM constant additive) to yield the title compound as a yellow solid (67 mg, 70%). $R_f$ = 0.64 (EtOAc:hexanes 1:2). $^1H$ NMR (400 MHz, Chloroform-*d*) δ 7.62 (d, *J* = 8.2 Hz, 2H), 7.14 (dt, *J* = 8.3, 2.1 Hz, 2H), 7.04 (dd, *J* = 5.4, 1.6 Hz, 2H), 4.69 (s, 4H), 2.28 (s, 6H), 2.23 – 2.11 (m, 4H), 1.96 (s, 3H), 1.92 (t, *J* = 2.6 Hz, 1H), 1.55 – 1.44 (m, 4H), 1.40 – 1.18 (m, 8H). $^{13}C$ NMR (100 MHz, $CDCl_3$) δ 173.49, 170.73, 156.85, 147.89, 141.58, 141.53, 139.14, 139.11, 138.03, 137.93, 132.24, 132.02, 130.98, 130.95, 127.22, 124.56, 124.42, 122.83, 122.68, 122.23, 122.20, 84.88, 68.24, 66.15, 65.86, 34.30, 29.20, 29.16, 29.00, 28.76, 28.55, 24.94, 21.09, 18.50, 15.91. HRMS (ESI, m/z): calcd. for $[C_{39}H_{40}N_5O_4S_4]^+$ $(M+NH_4)^+$, 770.19576; found, 770.19424.

**Scheme S5.** Synthesis of a **(±)-2-yne-ap** Containing the Photoactive Antiparallel DAE.

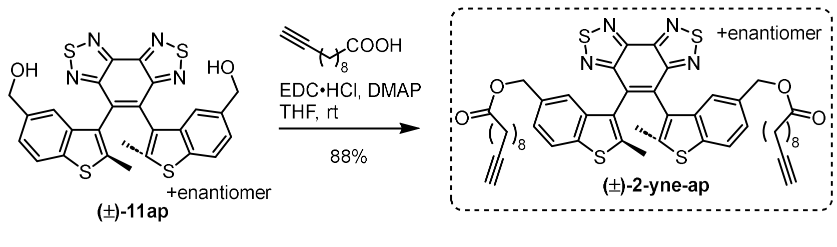

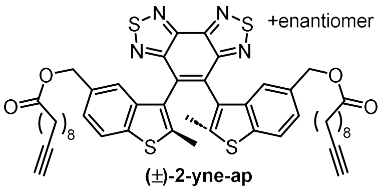


(±)-2-yne-ap

**Compound (±)-2-yne-ap.** A 7 mL vial equipped with a stir bar was charged with **(±)-11ap** (20 mg, 0.037 mmol) (reported in *J. Am. Chem. Soc.* **2023**, *145*, 15668−15673) and THF (2 mL). EDC·HCl (28 mg, 0.146 mmol), undec-10-ynoic acid (29 mg, 0.146 mmol) and DMAP (0.6 mg, 5 μmol) were added respectively. The stirring reaction was allowed to react for 16 h, then quenched with saturated $NH_4Cl$ (20 mL). The mixture was extracted with EtOAc (30 mL). The organic phase was washed with saturated $Na_2CO_3$ (30 mL×2), saturated $NH_4Cl$ (30 mL) and brine (30 mL), dried over $Na_2SO_4$, filtered, and concentrated under reduced pressure. The crude product was purified by column chromatography (10-25% EtOAc/hexanes, 10% DCM constant additive) to yield the title compound as a red solid (28 mg, 88%). $R_f$ = 0.39 (EtOAc:hexanes 1:5). $^1$H NMR (400 MHz, Chloroform-*d*) δ 7.67 (d, *J* = 8.2 Hz, 2H), 7.23 (d, *J* = 8.3 Hz, 2H), 7.10 (s, 2H), 5.09 – 4.97 (m, 4H), 2.24 (t, *J* = 7.5 Hz, 4H), 2.16 (td, *J* = 7.1, 2.7 Hz, 4H), 2.10 (s, 6H), 1.92 (t, *J* = 2.7 Hz, 2H), 1.58 – 1.43 (m, 8H), 1.39 – 1.29 (m, 4H), 1.27 – 1.22 (m, 12H). $^{13}$C NMR (101 MHz, $CDCl_3$) δ 173.55, 156.93, 147.84, 141.59, 139.31, 138.14, 132.04, 131.33, 126.87, 124.59, 122.66, 122.39, 84.84, 68.26, 66.29, 34.38, 29.18, 29.14, 28.99, 28.75, 28.53, 24.97, 18.49, 16.17. HRMS (ESI, m/z): calcd. for $[C_{48}H_{54}N_5O_4S_4]^+$ $(M+NH_4)^+$, 892.30531; found, 892.30444.

**Scheme S6.** Synthesis of the DAE Intermediate **17** in Isolated Atropisomeric Forms.

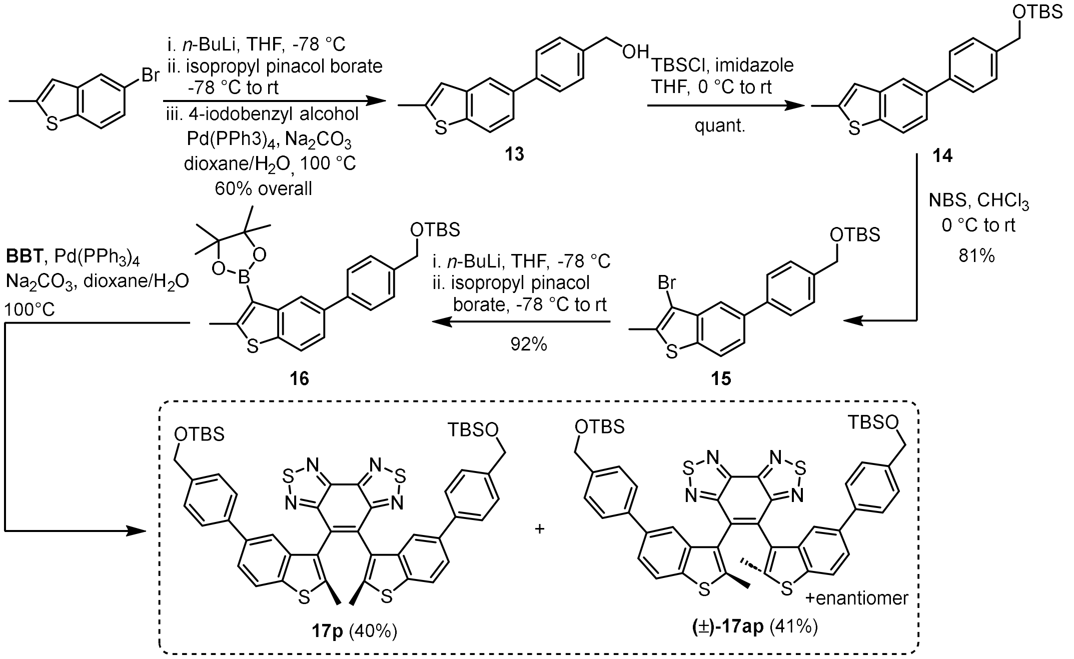

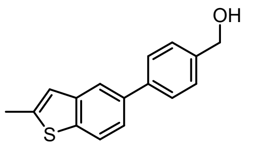

**13**

**Compound 13.** A 500 mL round bottom flask equipped with a stir bar was charged with 5-bromo-2-methylbenzothiophene (4.3 g, 18.9 mmol) and anhydrous THF (200 mL). The solution was cooled to -78 °C in an acetone/dry ice bath, followed by the dropwise addition n-BuLi (9 mL, 2.5 M, 22.5 mmol). The stirring reaction was retained at -78 °C for 1 h before adding 2-isopropoxy-4,4,5,5-tetramethyl-1,3,2-dioxaborolane (4.8 g, 25.8 mmol). The reaction was warmed to room temperature overnight, then quenched with saturated $NH_4Cl$ (100 mL). The mixture was extracted with $Et_2O$ (200 mL). The organic phase was washed with saturated $NH_4Cl$ (100 mL), saturated $NaHCO_3$ (100 mL) and brine (100 mL), dried over $Na_2SO_4$, filtered, and concentrated under reduced pressure. The crude product was directly used for the next step reaction.

A 250 mL three-neck round bottom flask equipped with a stir bar was charged with the crude product, 4-iodobenzyl alcohol (4.7 g, 20.1 mmol), 1,4-dioxane (100 mL) and 2 M $Na_2CO_3$ aqueous solution (20 mL). The mixture was purged by $N_2$ for 30 min. $Pd(PPh_3)_4$ (115 mg, 0.1 mmol) was added then, followed by another 30 min purging. The mixture was heated to 100 °C for 16 h. Then the mixture was extracted with EtOAc (200 mL). The organic phase was washed with saturated $NH_4Cl$ (100 mL×3), dried over $Na_2SO_4$, filtered, and concentrated under reduced pressure. The crude product was purified by column chromatography (10-30% EtOAc/hexanes) followed by recrystallization in hexanes/EtOAc (2:1) to yield the title compound as a white solid (2.88 g, 60% in two steps). $R_f$ = 0.42 (EtOAc:hexanes 1:2). $^1H$ NMR (400 MHz, Chloroform-*d*) δ 7.85 (d, *J* = 1.8 Hz, 1H), 7.80 (d, *J* = 8.3 Hz, 1H), 7.69 – 7.61 (m, 2H), 7.52 – 7.43 (m, 3H), 7.05 – 6.99 (m, 1H), 4.83 – 4.68 (m, 2H), 2.61 (d, *J* = 1.2 Hz, 3H), 1.65 (s, 1H). $^{13}C$ NMR (100 MHz, $CDCl_3$) δ 141.91, 141.20, 141.15, 139.78, 138.99, 137.30, 127.68, 123.02, 122.40, 121.92, 121.06, 65.34, 16.40. HRMS (ESI, m/z): calcd. for $[C_{16}H_{14}OS]^+$ $M^+$, 254.07599; found, 254.07534.

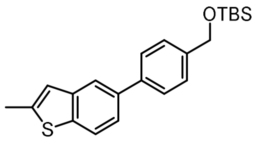

**14**

**Compound 14.** A 250 mL round bottom flask equipped with a stir bar was charged with **13** (3.0 g, 11.8 mmol), imidazole (1.02 g, 15 mmol) and anhydrous THF (100 mL). The solution was cooled to 0 °C in an ice bath, followed by the dropwise addition TBSCl (2.26 g, 15 mmol) solution in THF (10 mL). The stirring reaction was warmed up to room temperature overnight. The reaction was quenched with water (40 mL). The mixture was extracted with $Et_2O$ (100 mL). The organic phase was washed with saturated $NH_4Cl$ (100 mL), saturated $NaHCO_3$ (100 mL) and brine (100 mL), dried over $Na_2SO_4$, filtered, and concentrated under reduced pressure. The crude product was purified by column chromatography (0-5% EtOAc/hexanes) to yield the title compound as a white solid (4.35 g, quant.). $R_f$ = 0.45 (EtOAc:hexanes 5:95). $^1H$ NMR (400 MHz, Chloroform-*d*) δ 7.85 (d, *J* = 1.8 Hz, 1H), 7.79 (dd, *J* = 8.2, 0.9 Hz, 1H), 7.64 – 7.58 (m, 2H), 7.49 (dd, *J* = 8.3, 1.8 Hz,

1H), 7.41 (d, $J$ = 8.1 Hz, 2H), 7.04 – 6.98 (m, 1H), 4.80 (s, 2H), 2.61 (d, $J$ = 1.2 Hz, 3H), 0.97 (s, 9H), 0.14 (s, 6H). $^{13}$C NMR (100 MHz, $CDCl_3$) δ 141.75, 141.18, 140.43, 140.31, 138.78, 137.59, 127.32, 126.66, 123.07, 122.32, 121.94, 121.02, 64.93, 26.14, 18.61, 16.39, -5.05. HRMS (ESI, m/z): calcd. for $[C_{22}H_{32}NOSSi]^+$ $(M+NH_4)^+$, 386.19684; found, 386.19582.

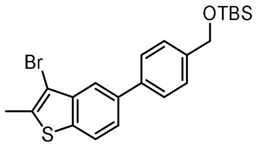


**15**

**Compound 15.** A 250 mL round bottom flask equipped with a stir bar was charged with **14** (4.2 g, 11.4 mmol) and chloroform (80 mL). The mixture was cooled to 0 °C in an ice bath, followed by the addition of N-bromosuccinimide (NBS, 2.03 g, 11.4 mmol) in several portions in the dark. The reaction was warmed up to room temperature overnight in the dark. The solution was poured into water (100 mL) and extracted with $Et_2O$ (200 mL). The organic phase was washed with water (200 mL), saturated $Na_2CO_3$ (100 mL), and saturated $NH_4Cl$ (200 mL), dried over $Na_2SO_4$, filtered, and concentrated under reduced pressure. The crude product was purified by column chromatography (100% hexanes) to yield the title compound as a white solid (4.2 g, 81%). $R_f$ = 0.45 (EtOAc:hexanes 5:95). $^1$H NMR (400 MHz, Chloroform-*d*) δ 7.91 (d, $J$ = 1.7 Hz, 1H), 7.80 – 7.75 (m, 1H), 7.69 – 7.63 (m, 2H), 7.58 (dd, $J$ = 8.3, 1.8 Hz, 1H), 7.47 – 7.42 (m, 2H), 4.82 (s, 2H), 2.58 (s, 3H), 0.98 (s, 9H), 0.15 (s, 6H). $^{13}$C NMR (100 MHz, $CDCl_3$) δ 140.80, 139.80, 139.11, 138.50, 136.21, 136.03, 127.41, 126.71, 124.41, 122.56, 121.02, 106.96, 64.89, 26.14, 18.60, 15.74, -5.05. HRMS (ESI, m/z): calcd. for $[C_{22}H_{31}NOBrSSi]^+$ $(M+NH_4)^+$, 464.10735; found, 464.10618.

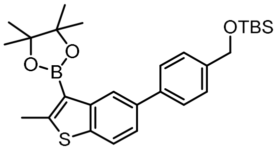


**16**

**Compound 16.** A 250 mL round bottom flask equipped with a stir bar was charged with **15** (4.5 g, 10.1 mmol) and anhydrous THF (150 mL). The solution was cooled to -78 °C in an acetone/dry ice bath, followed by the dropwise addition n-BuLi (4.4 mL, 2.5 M, 11.1 mmol). The stirring reaction was retained at -78 °C for 1 h before adding 2-isopropoxy-4,4,5,5-tetramethyl-1,3,2-dioxaborolane (2.8 g, 15.2 mmol). The reaction was warmed up to room temperature overnight, then quenched with saturated $NH_4Cl$ (50 mL). The mixture was extracted with $Et_2O$ (100 mL). The organic phase was washed with saturated $NH_4Cl$ (100 mL), saturated $NaHCO_3$ (100 mL) and brine (100 mL), dried over $Na_2SO_4$, filtered, and concentrated under reduced pressure. The crude product was purified by column chromatography (0-5% EtOAc/hexanes, 1% DCM constant additive) to yield the title compound as a white solid (4.6 g, 92%). $R_f$ = 0.36 (EtOAc:hexanes 5:95). $^1$H NMR (400 MHz, Chloroform-*d*) δ 8.54 (d, $J$ = 1.8 Hz, 1H), 7.79 (d, $J$ = 8.3 Hz, 1H), 7.70 – 7.63 (m, 2H), 7.50 (dd, $J$ = 8.3, 1.8 Hz, 1H), 7.45 (d, $J$ = 7.9 Hz, 2H), 4.83 (s, 2H), 2.84 (s, 3H), 1.41 (s, 12H), 1.00 (s, 9H), 0.98 (d, $J$ = 1.6 Hz, 1H), 0.17 (s, 6H). $^{13}$C NMR (100 MHz, $CDCl_3$) δ 155.84, 145.39, 140.93, 140.14, 138.62, 137.48, 127.43, 126.62, 123.36, 123.03, 121.59, 83.27,

64.98, 26.16, 25.15, 18.63, 16.99, -5.06. HRMS (ESI, m/z): calcd. for $[C_{28}H_{43}NO_3BSSi]^+$ $(M+NH_4)^+$, 512.28205; found, 512.28083.

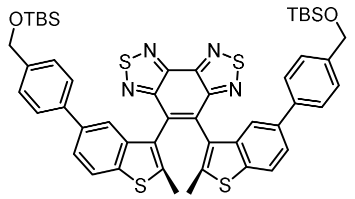

**17p**

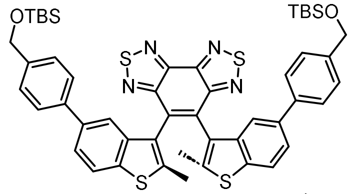

+enantiomer

**(±)-17ap**

**Compound 17.** A 100 mL three-neck round bottom flask equipped with a stir bar was charged with **16** (1.98 g, 4.0 mmol) , **BBT** (352 mg, 1.0 mmol), 1,4-dioxane (100 mL) and 2 M $Na_2CO_3$ aqueous solution (20 mL). The mixed solution was purged by $N_2$ for 30 min. $Pd(PPh_3)_4$ (83 mg, 0.07 mmol) was added and followed by another 30 min purging. The mixture was heated to 100 °C for 72 h then extracted with EtOAc (150 mL). The organic phase was washed with saturated $NH_4Cl$ (100 mL×3), dried over $Na_2SO_4$, filtered, and concentrated under reduced pressure. The crude product was purified by column chromatography (0-15% EtOAc/hexanes) to yield the title compound as a yellow solid (370 mg, 40%). We also obtained the antiparallel stereoisomer **(±)-17ap** (380 mg) which contained about 35% impurities that were difficult to remove. Compound **(±)-17ap** was used in the following step without further purification.

**17p**: $R_f$ = 0.28 (EtOAc:hexanes 1:9). $^1$H NMR (400 MHz, Chloroform-*d*) δ 7.69 (d, *J* = 8.3 Hz, 1H), 7.35 (dd, *J* = 8.4, 1.7 Hz, 1H), 7.17 (d, *J* = 1.7 Hz, 1H), 7.04 (d, *J* = 8.0 Hz, 2H), 6.94 (d, *J* = 8.2 Hz, 2H), 4.60 (s, 2H), 2.28 (s, 3H), 0.92 (s, 10H), 0.06 (d, *J* = 1.6 Hz, 6H). $^{13}$C NMR (100 MHz, $CDCl_3$) δ 157.08, 147.84, 141.48, 140.08, 139.67, 139.55, 137.83, 137.09, 131.13, 127.44, 127.11, 126.31, 123.75, 122.39, 120.74, 64.75, 26.11, 18.55, 16.11, -5.11. HRMS (ESI, m/z): calcd. for $[C_{50}H_{58}N_5O_2S_4Si_2]^+$ $(M+NH_4)^+$, 944.30064; found, 944.29935.

**(±)-17ap**: $R_f$ = 0.32 (EtOAc:hexanes 1:9). $^1$H NMR (400 MHz, Chloroform-*d*) δ 8.08 (s, 1.1H)*, 7.91 (d, *J* = 8.3 Hz, 1.1H)*, 7.76 (d, *J* = 8.4 Hz, 2H), 7.58 (dd, *J* = 8.3, 1.8 Hz, 1.3H)*, 7.48 (dd, *J* = 8.4, 1.7 Hz, 2H), 7.45 (s, 2H), 7.43 (s, 1.1 H)*, 7.38 (d, *J* = 8.2 Hz, 4H), 7.30 (d, *J* = 8.0 Hz, 4H), 7.30 (d, *J* = 8.0 Hz, 1.3H)*, 4.73 (s, 4H), 4.72 (s, 2.3H)*, 2.51 (s, 3.5H)*, 1.97 (s, 6H), 0.93 (s, 18H), 0.92 (s, 9.5H)*, 0.09 (s, 12H), 0.08 (s, 6.4H)*. HRMS (ESI, m/z): calcd. For $[C_{50}H_{58}N_5O_2S_4Si_2]^+$ $(M+NH_4)^+$, 944.30064; found, 944.29956.

**Scheme S7.** Synthesis of Materials Containing Mechanophore **M3**.

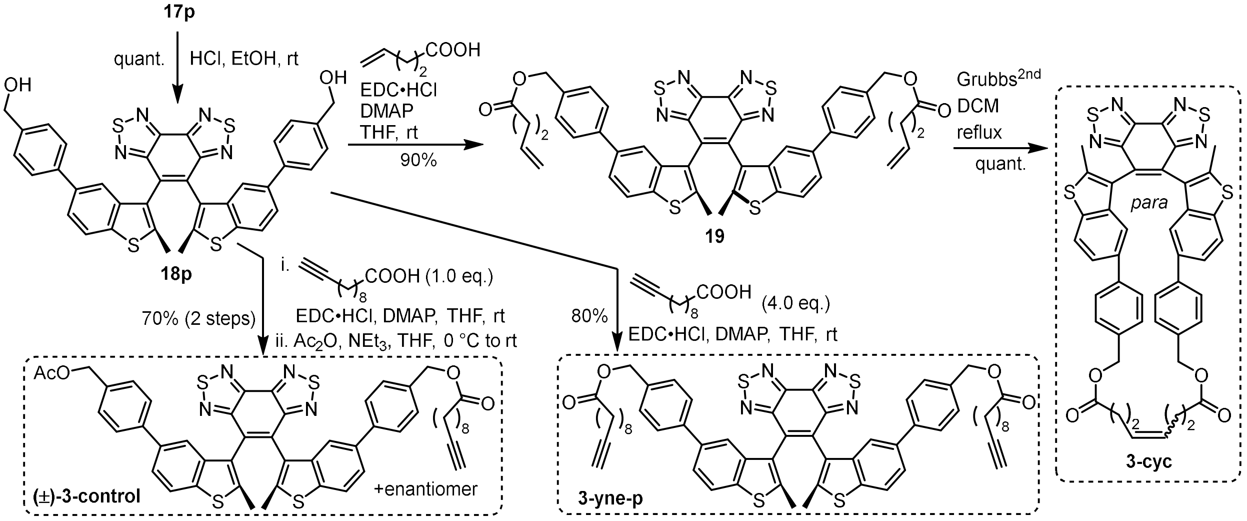


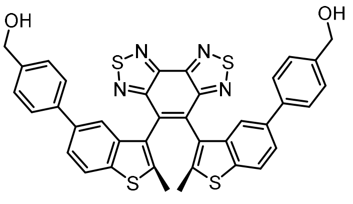

**18p**

**Compound 18p.** A 20 mL vial equipped with a stir bar was charged with **17p** (370 mg, 0.40 mmol) and 10 mL of 1 wt.% HCl in ethanol . The reaction was allowed to stir at room temperature for 2 h. The mixture was extracted with EtOAc (30 mL). The organic phase was washed with saturated $NH_4Cl$ (20 mL), saturated $NaHCO_3$ (20 mL) and brine (20 mL), dried over $Na_2SO_4$, filtered, and concentrated under reduced pressure. The crude product was purified by column chromatography (0-30% EtOAc/DCM) to yield the title compound as a yellow solid (278 mg, quant.). $R_f$ = 0.26 (EtOAc:DCM 1:5). $^1$H NMR (400 MHz, Chloroform-*d*) δ 7.72 – 7.64 (m, 2H), 7.31 (dd, *J* = 8.3, 1.7 Hz, 2H), 7.24 (d, *J* = 1.7 Hz, 2H), 7.00 (s, 8H), 4.51 (s, 4H), 2.35 (s, 6H). $^{13}$C NMR (100 MHz, $CDCl_3$) δ 157.01, 147.85, 141.40, 140.31, 139.69, 139.55, 137.32, 137.27, 131.32, 127.42, 127.35, 123.57, 122.41, 120.65, 64.98, 16.17. HRMS (ESI, m/z): calcd. for $[C_{38}H_{30}N_5O_2S_4]^+$ $(M+NH_4)^+$, 716.12768; found, 716.12632.

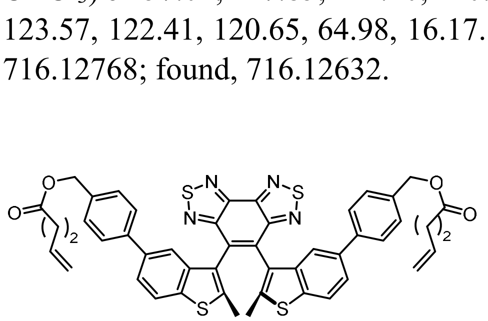

**19**

**Compound 19.** A 20 mL vial equipped with a stir bar was charged with **18p** (100 mg, 0.143 mmol) and THF (5 mL). EDC·HCl (73 mg, 0.38 mmol), 4-pentenoic acid (38 mg, 0.38 mmol) and DMAP

(4 mg 0.03 mmol) were added respectively. The stirring reaction was allowed to react for 16 h, then quenched with saturated $NH_4Cl$ (20 mL). The mixture was extracted with EtOAc (30 mL). The organic phase was washed with saturated $Na_2CO_3$ (30 mL×2), saturated $NH_4Cl$ (30 mL) and brine (30 mL), dried over $Na_2SO_4$, filtered, and concentrated under reduced pressure. The crude product was purified by column chromatography (10-25% EtOAc/hexanes, 10% DCM constant additive) to yield the title compound as a yellow solid (110 mg, 90%). $R_f$ = 0.27 (EtOAc:hexanes 1:5). $^1$H NMR (400 MHz, Chloroform-*d*) δ 7.70 (d, *J* = 8.3 Hz, 2H), 7.32 (dd, *J* = 8.3, 1.7 Hz, 2H), 7.30 (s, 1H), 7.18 (d, *J* = 1.7 Hz, 2H), 7.06 (d, *J* = 7.9 Hz, 4H), 6.97 (d, *J* = 7.9 Hz, 4H), 5.81 (ddt, *J* = 16.3, 10.1, 6.0 Hz, 2H), 5.09 – 4.93 (m, 8H), 2.47 – 2.32 (m, 8H), 2.30 (s, 6H). $^{13}$C NMR (100 MHz, $CDCl_3$) δ 172.90, 156.99, 147.83, 141.70, 140.94, 139.55, 137.40, 137.25, 136.71, 134.60, 131.07, 128.47, 127.39, 127.37, 123.59, 122.46, 120.75, 115.72, 65.95, 33.62, 28.95, 16.11. HRMS (ESI, m/z): calcd. for $[C_{48}H_{42}N_5O_4S_4]^+$ $(M+NH_4)^+$, 880.21141; found, 880.20970.

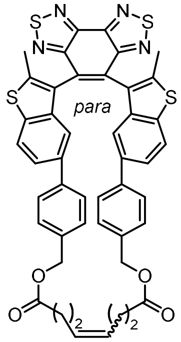


**3-cyc**

**Compound 3-cyc.** A 250 mL three-neck round bottom flask equipped with a stir bar was charged with **19** (110 mg, 0.127 mmol) and DCM (150 mL) and purged by $N_2$ for 30 min. Then Grubbs II catalyst (8.5 mg, 0.01 mmol) was added, and the mixture was purged for another 30 min. The stirring reaction was heated to reflux and allowed to react for 16 h. Then 1 mL ethyl vinyl ether was added, and the reaction was kept refluxing for 1 h. The solution was cooled and concentrated under reduced pressure. The crude product was purified by column chromatography (10-25% EtOAc/hexanes, 10% DCM constant additive) to yield the title compound as a yellow solid (106 mg, quant.). The product consisted of a 1:4.8 (E:Z) isomeric mixture, which was used directly in the next step. $R_f$ = 0.15 (EtOAc:hexanes 1:5). $^1$H NMR (400 MHz, Chloroform-*d*) δ 7.71 (d, *J* = 8.3 Hz, 2H), 7.34 (dd, *J* = 8.4, 1.7 Hz, 2H), 7.20 – 7.12 (m, 2H), 7.12 – 7.05 (m, 4H), 7.02 – 6.92 (m, 4H), 5.56 – 5.44 (m, 1.5H), 5.43 – 5.38 (m, 0.3H), 4.99 (s, 3.2H), 4.98 (s, 0.8H), 2.45 – 2.21 (m, 14H). $^{13}$C NMR (100 MHz, $CDCl_3$) δ 173.03, 157.04, 147.82, 141.82, 140.98, 139.54, 137.41, 137.40, 134.62, 131.00, 129.74, 128.57, 128.14, 127.41, 123.68, 122.47, 120.89, 65.94, 34.45, 27.89, 16.10. HRMS (ESI, m/z): calcd. for $[C_{46}H_{38}N_5O_4S_4]^+$ $(M+NH_4)^+$, 852.18011; found, 852.17869.

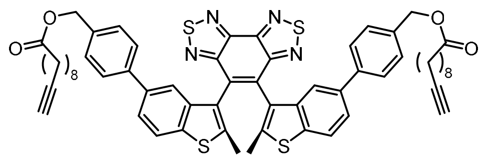

**3-yne-p**

**Compound 3-yne-p.** A 20 mL vial equipped with a stir bar was charged with **18p** (100 mg, 0.143 mmol) and THF (5 mL). EDC·HCl (110 mg, 0.572 mmol), undec-10-ynoic acid (104 mg, 0.572 mmol) and DMAP (4 mg 0.03 mmol) were added respectively. The stirring reaction was allowed to react for 16 h, then quenched with saturated $NH_4Cl$ (20 mL). The mixture was extracted with EtOAc (30 mL). The organic phase was washed with saturated $Na_2CO_3$ (30 mL×2), saturated $NH_4Cl$ (30 mL) and brine (30 mL), dried over $Na_2SO_4$, filtered, and concentrated under reduced pressure. The crude product was purified by column chromatography (10-25% EtOAc/hexanes, 10% DCM constant additive) to yield the title compound as a yellow solid (117 mg, 80%). $R_f$ = 0.27 (EtOAc:hexanes 1:5). $^1$H NMR (400 MHz, Chloroform-*d*) δ 7.69 (d, *J* = 8.3 Hz, 2H), 7.32 (dd, *J* = 8.4, 1.7 Hz, 2H), 7.17 (d, *J* = 1.7 Hz, 2H), 7.06 (d, *J* = 8.2 Hz, 4H), 6.99 – 6.92 (m, 4H), 4.97 (s, 4H), 2.31 (d, *J* = 6.9 Hz, 10H), 2.16 (td, *J* = 7.1, 2.6 Hz, 4H), 1.92 (t, *J* = 2.6 Hz, 2H), 1.69 – 1.55 (m, 4H), 1.55 – 1.45 (m, 4H), 1.42 – 1.24 (m, 16H). $^{13}$C NMR (100 MHz, $CDCl_3$) δ 173.66, 156.99, 147.83, 141.68, 140.88, 139.54, 137.38, 137.25, 134.72, 131.07, 128.43, 127.39, 127.36, 123.58, 122.45, 120.74, 84.83, 68.26, 65.79, 34.38, 29.24, 29.20, 29.02, 28.77, 28.54, 25.02, 18.49, 16.10. HRMS (ESI, m/z): calcd. for $[C_{60}H_{62}N_5O_4S_4]^+$ $(M+NH_4)^+$, 1044.36791; found, 1044.36615.

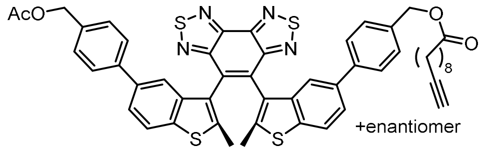


**(±)-3-control**

**Compound (±)-3-control.** A 20 mL vial equipped with a stir bar was charged with **18p** (100 mg, 0.143 mmol) and THF (5 mL). EDC·HCl (27 mg, 0.143 mmol), undec-10-ynoic acid (26 mg, 0.143 mmol) and DMAP (3 mg 0.026 mmol) were added respectively. The stirring reaction was allowed to react for 16 h, then quenched with saturated $NH_4Cl$ (20 mL). The mixture was extracted with EtOAc (30 mL). The organic phase was washed with saturated $Na_2CO_3$ (30 mL×2), saturated $NH_4Cl$ (30 mL) and brine (30 mL), dried over $Na_2SO_4$, filtered, and concentrated under reduced pressure. The crude product was not purified and directly used for the next reaction.

A 20 mL vial equipped with a stir bar was charged with the crude and anhydrous THF (5 mL). The solution was cooled to 0 °C in an ice bath, followed by the addition of triethylamine (0.5 mL). Acetic anhydride (0.5 mL) was added dropwise to the mixture. The stirring reaction was warmed up to room temperature overnight. The reaction was quenched by saturated $NH_4Cl$ (10 mL). The mixture was extracted with EtOAc (30 mL). The organic phase was washed with saturated $Na_2CO_3$ (30 mL×2), saturated $NH_4Cl$ (30 mL) and brine (30 mL), dried over $Na_2SO_4$, filtered, and concentrated under reduced pressure. The crude product was purified by column chromatography (10-25% EtOAc/hexanes, 10% DCM constant additive) to yield the title compound as a yellow solid (91 mg, 70%). $R_f$ = 0.21 (EtOAc:hexanes 1:5). $^1$H NMR (400 MHz, Chloroform-*d*) δ 7.70

(d, $J$ = 8.3 Hz, 2H), 7.36 – 7.28 (m, 2H), 7.17 (s, 2H), 7.10 – 7.03 (m, 4H), 7.01 – 6.92 (m, 4H), 4.98 (s, 2H), 4.97 (s, 2H), 2.35 – 2.27 (m, 8H), 2.16 (td, $J$ = 7.0, 2.7 Hz, 2H), 2.06 (s, 3H), 1.92 (t, $J$ = 2.6 Hz, 1H), 1.69 – 1.59 (m, 2H), 1.54 – 1.44 (m, 2H), 1.42 – 1.26 (m, 8H). $^{13}$C NMR (100 MHz, $CDCl_3$) δ 173.69, 170.90, 157.01, 147.84, 141.70, 140.99, 140.92, 139.59, 139.53, 137.41, 137.28, 137.26, 134.74, 134.54, 131.09, 128.52, 128.45, 127.40, 123.61, 122.48, 120.78, 120.75, 84.85, 68.26, 66.03, 65.81, 34.39, 29.24, 29.21, 29.03, 28.78, 28.55, 25.03, 21.12, 18.50, 16.13, 16.11. HRMS (ESI, m/z): calcd. for $[C_{51}H_{48}N_5O_4S_4]^+$ $(M+NH_4)^+$, 922.25836; found, 922.25690.

**Scheme S8.** Synthesis of a **(±)-3-yne-ap** Containing the Photoactive Antiparallel DAE.

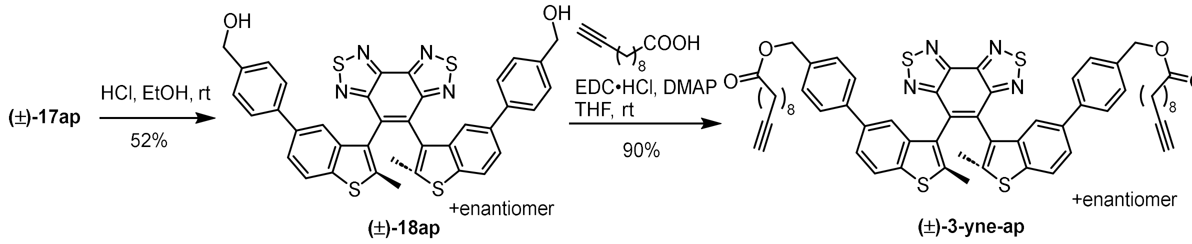


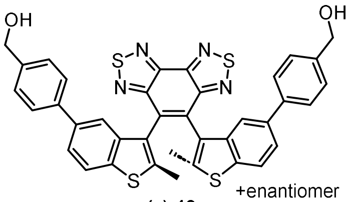
(±)-18ap

**Compound (±)-18ap.** A 20 mL vial equipped with a stir bar was charged with crude **(±)-17ap** (100 mg, 0.108 mmol) and 5 mL of 1 wt.% HCl in ethanol . The reaction was allowed to stir at room temperature for 2 h. The mixture was extracted with EtOAc (30 mL). The organic phase was washed with saturated $NH_4Cl$ (20 mL), saturated $NaHCO_3$ (20 mL) and brine (20 mL), dried over $Na_2SO_4$, filtered, and concentrated under reduced pressure. The crude product was purified by column chromatography (0-30% EtOAc/DCM) to yield the title compound as a red solid (39 mg, 52%). $R_f$ = 0.31 (EtOAc:DCM 1:5). $^1$H NMR (400 MHz, Chloroform-*d*) δ 7.76 (d, $J$ = 8.3 Hz, 2H), 7.48 (dd, $J$ = 8.4, 1.7 Hz, 2H), 7.44 – 7.32 (m, 8H), 7.27 (d, $J$ = 1.7 Hz, 2H), 4.70 (s, 4H), 1.99 (s, 6H), 1.64 (s, 2H). $^{13}$C NMR (101 MHz, $CDCl_3$) δ 157.05, 147.88, 141.64, 140.82, 139.89, 139.85, 137.40, 137.27, 131.56, 127.61, 127.52, 127.04, 123.64, 122.55, 121.00, 65.18, 16.20. HRMS (ESI, m/z): calcd. for $[C_{38}H_{30}N_5O_2S_4]^+$ $(M+NH_4)^+$, 716.12768; found, 716.12673.

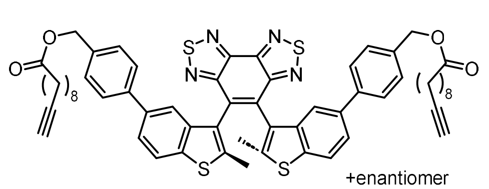
(±)-3-yne-ap

**Compound (±)-3-yne-ap.** A 7 mL vial equipped with a stir bar was charged with **(±)-18ap** (30 mg, 0.043 mmol) and THF (3 mL). EDC·HCl (33 mg, 0.172 mmol), undec-10-ynoic acid (34 mg, 0.172 mmol) and DMAP (1 mg, 8 μmol) were added respectively. The stirring reaction was allowed to react for 16 h, then quenched with saturated $NH_4Cl$ (20 mL). The mixture was extracted with

EtOAc (30 mL). The organic phase was washed with saturated $Na_2CO_3$ (30 mL×2), saturated $NH_4Cl$ (30 mL) and brine (30 mL), dried over $Na_2SO_4$, filtered, and concentrated under reduced pressure. The crude product was purified by column chromatography (10-25% EtOAc/hexanes, 10% DCM constant additive) to yield the title compound as a red solid (40 mg, 90%). $R_f$ = 0.35 (EtOAc:hexanes 1:5). $^1$H NMR (400 MHz, Chloroform-*d*) δ 7.78 (d, *J* = 8.3 Hz, 2H), 7.48 (dd, *J* = 8.4, 1.7 Hz, 2H), 7.43 – 7.30 (m, 9H), 7.26 (s, 2H), 5.10 (s, 4H), 2.33 (t, *J* = 7.5 Hz, 4H), 2.16 (td, *J* = 7.1, 2.7 Hz, 4H), 1.97 (s, 6H), 1.92 (t, *J* = 2.7 Hz, 2H), 1.69 – 1.58 (m, 4H), 1.53 – 1.44 (m, 4H), 1.41 – 1.23 (m, 16H). $^{13}$C NMR (101 MHz, $CDCl_3$) δ 173.78, 157.03, 147.89, 141.63, 141.32, 139.92, 137.50, 137.15, 135.18, 131.57, 128.74, 127.59, 127.03, 123.63, 122.60, 121.09, 84.85, 68.26, 65.89, 34.43, 29.21, 29.19, 29.02, 28.77, 28.55, 25.03, 18.51, 16.15. HRMS (ESI, m/z): calcd. for $[C_{60}H_{62}N_5O_4S_4]^+$ $(M+NH_4)^+$, 1044.36791; found, 1044.36755.

**General Procedure A for Ring-opening Metathesis Polymerizations (ROMPs)**

ROMP conditions were adopted from literature (*J. Am. Chem. Soc.* **2024**, *146*, 95-100; *Nat. Commun.* **2016**, *7*, 13433). We found that when our mechanoresponsive cyclic monomers and an equal molar amount of 1,2-epoxy-5-cyclooctene were subjected to standard ROMP conditions, the cyclooctene was quickly consumed with minimal incorporation of the mechanophore. Additionally, homopolymerization of cyclic mechanophores failed. Those results collectively suggest the low reactivity of the mechanoresponsive cyclic monomers, which could be explained by the bulky mechanophore structures. Elling and Xia (*J. Am. Chem. Soc.* **2015**, *137*, 31, 9922–9926) have demonstrated the successful alternating ROMP of sterically hindered ROMP monomers with comonomers. Inspired by their work, we reasoned that the slow addition of the more reactive and less bulky 1,2-epoxy-5-cyclooctene comonomer into a concentrated solution of the cyclic mechanophores would promote the incorporation of the mechanophores through copolymerization (homopolymerization of the cyclic mechanophores is sterically hindered). At the same time, the slow addition ensures that the concentration of the 1,2-epoxy-5-cyclooctene comonomer was maintained relatively low for enhanced mechanophore content in the resulting copolymer.

A 7 mL vial equipped with a stir bar was charged with the diarylethene cycloolefin (1 equivalent) and DCM (0.2 mL). From a 2 mg/mL stock solution of Grubbs II catalyst (2.4 mmol/mL) in DCM, 0.1 mL of the freshly prepared catalyst solution was added to the vial. To the stirred reaction mixture was added a 50 μL stock solution of 1,2-epoxy-5-cyclooctene in DCM (40 mg/mL, 0.32 mmol/mL) every 15 minutes. After every four additions, the solvent was removed under reduced pressure to reduce the volume to approximately 0.3 mL, ensuring the appropriate monomer concentration for polymerization. The progress of the reaction was monitored by NMR. After 16-18 hours, when the consumption rate of diarylethene cycloolefin significantly decreased, 0.1 mL of ethyl vinyl ether was added to react for an additional hour. The polymer solution was precipitated sequentially in cold methanol (50 mL), cold diethyl ether (50 mL), and cold hexanes (50 mL). The isolated polymer was thoroughly dried under vacuum. **P1-P3** were obtained for subsequent SMFS studies.

**Scheme S9.** Synthesis of copolymers **P1-P3** by ROMP.

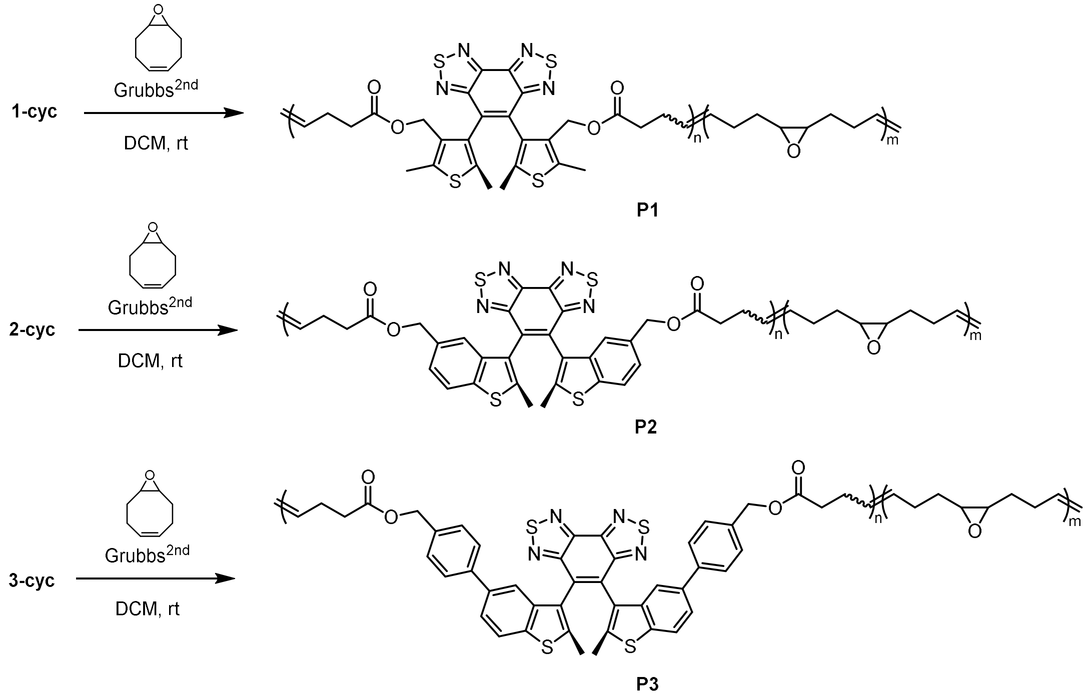


**P1.** Synthesized using General Procedure A with **1-cyc** (30 mg, 0.050 mmol), Grubbs II catalyst (0.2 mg, 0.24 μmol) and 1,2-epoxy-5-cyclooctene (140 mg in total). The title polymer was isolated as a gummy pale-yellow solid (105 mg, 62%). Diarylethene/Epoxide ratio n/m=1/20.6, $Mn^{GPC}$ = 57.9 kg/mol, $Đ$ = 1.91.

**P2.** Synthesized using General Procedure A with **2-cyc** (35 mg, 0.050 mmol), Grubbs II catalyst (0.2 mg, 0.24 μmol) and 1,2-epoxy-5-cyclooctene (140 mg in total). The title polymer was isolated as a gummy yellow solid (98 mg, 56%). Diarylethene/Epoxide ratio n/m=1/24.5, $Mn^{GPC}$ = 64.8 kg/mol, $Đ$ = 2.47.

**P3.** Synthesized using General Procedure A with **3-cyc** (41 mg, 0.050 mmol), Grubbs II catalyst (0.2 mg, 0.24 μmol) and 1,2-epoxy-5-cyclooctene (140 mg in total). The title polymer was isolated as a gummy orange-yellow solid (80 mg, 44%). Diarylethene/Epoxide ratio n/m=1/15.5, $Mn^{GPC}$ = 47.4 kg/mol, $Đ$ = 1.76.

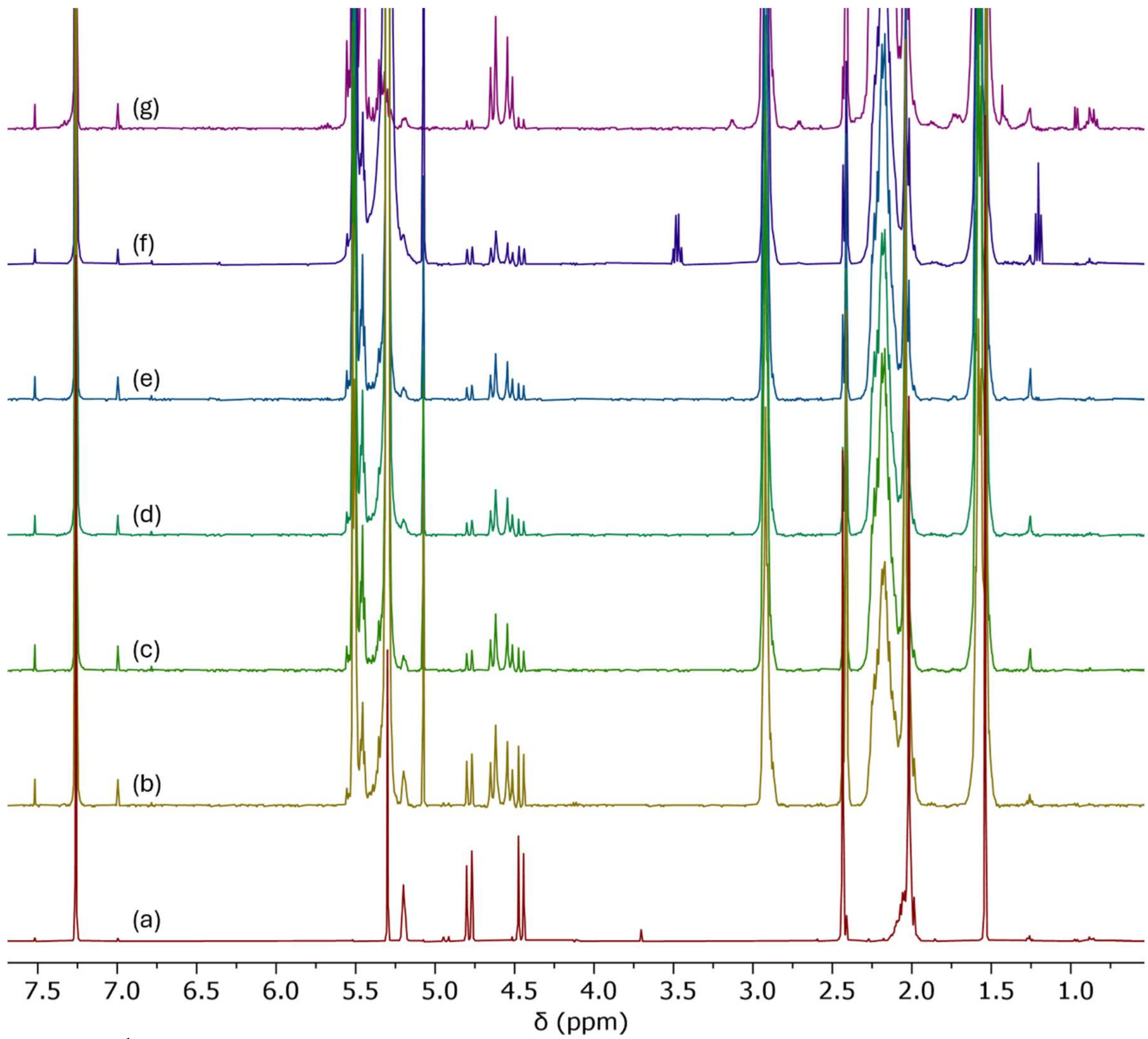


**Figure S25.** $^{1}$H NMR spectra (400 MHz, Chloroform-*d*) demonstrating the ROMP process of **P1**. From the bottom to the top: (a) **1-cyc**; (b)-(f) Reaction mixture at 3 h, 6 h, 9 h, 12 h, 15 h after the reaction started; (g) Isolated **P1**.

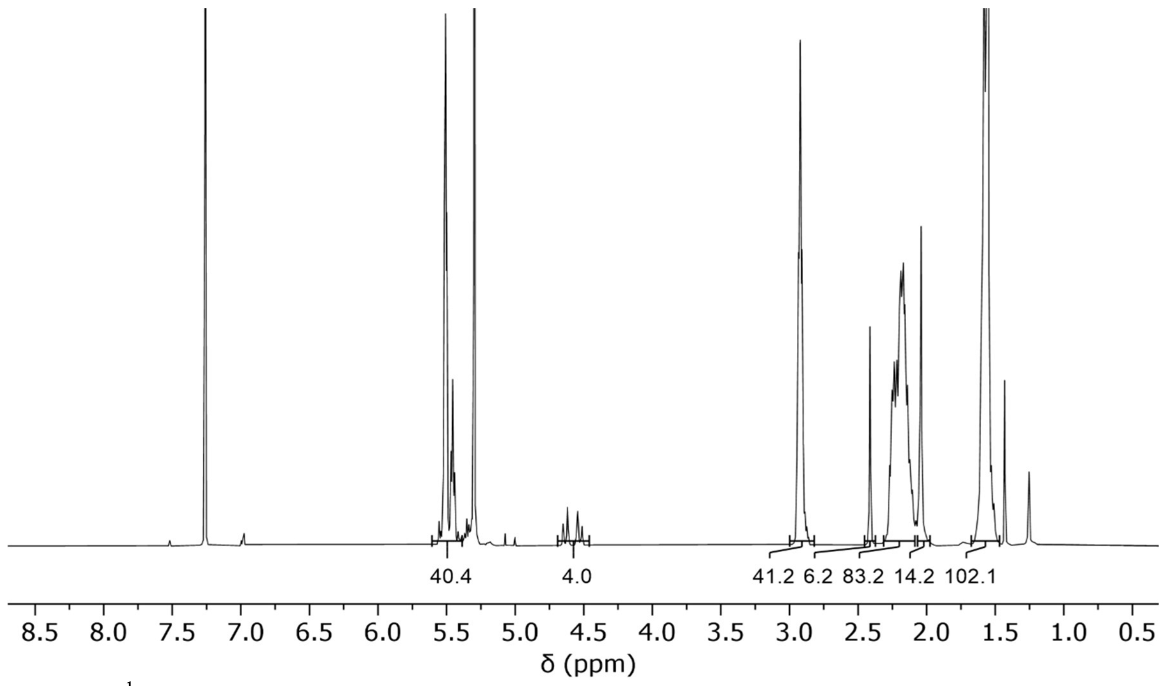


**Figure S26.** $^{1}$H NMR spectrum (400 MHz, Chloroform-*d*) for **P1**. Mechanophore mol % = 4.6 %.

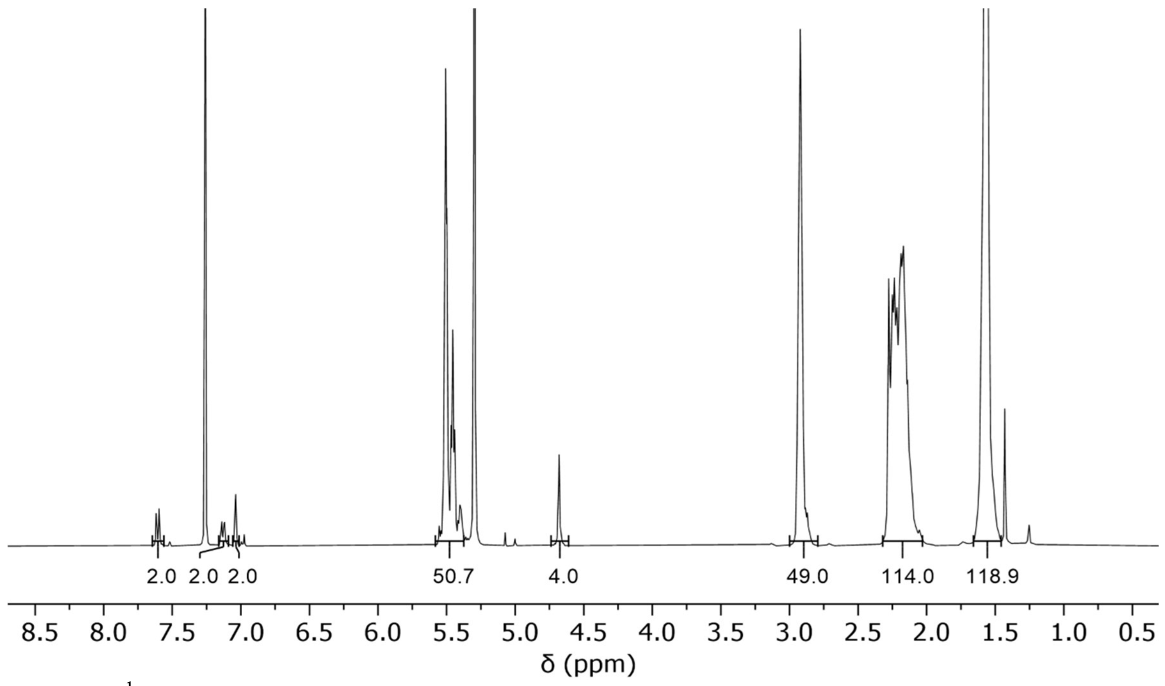


**Figure S27.** $^{1}$H NMR spectrum (400 MHz, Chloroform-*d*) for **P2**. Mechanophore mol % = 3.9 %.

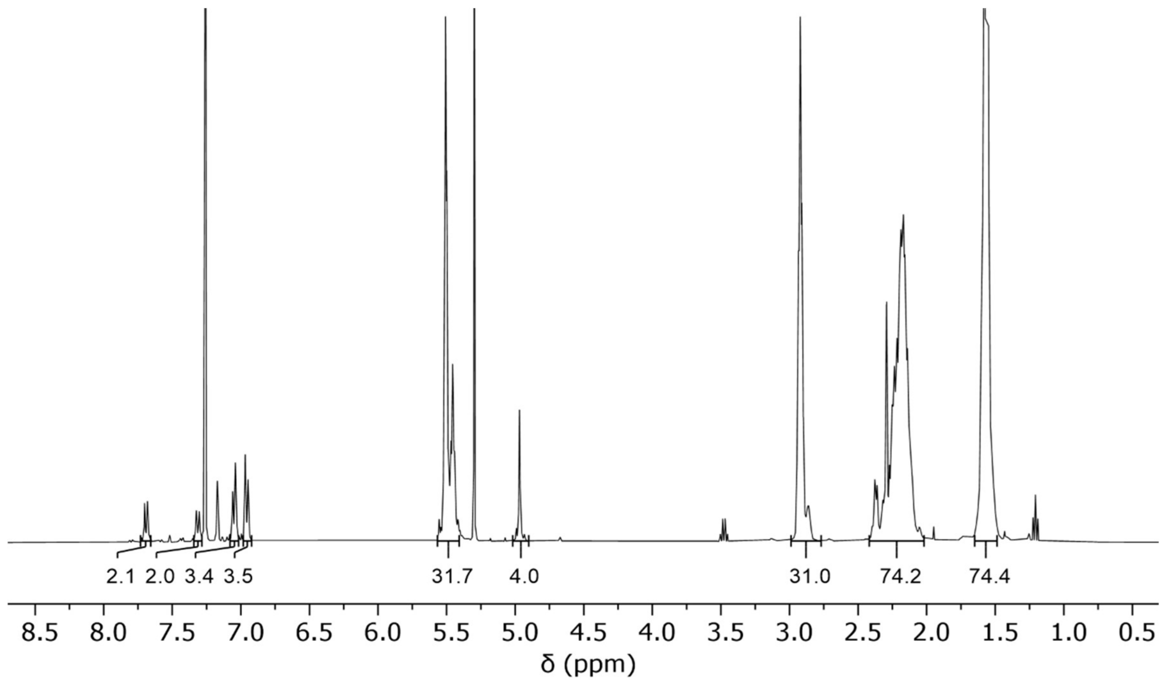


**Figure S28.** $^{1}$H NMR spectrum (400 MHz, Chloroform-*d*) for **P3**. Mechanophore mol % = 6.1 %.

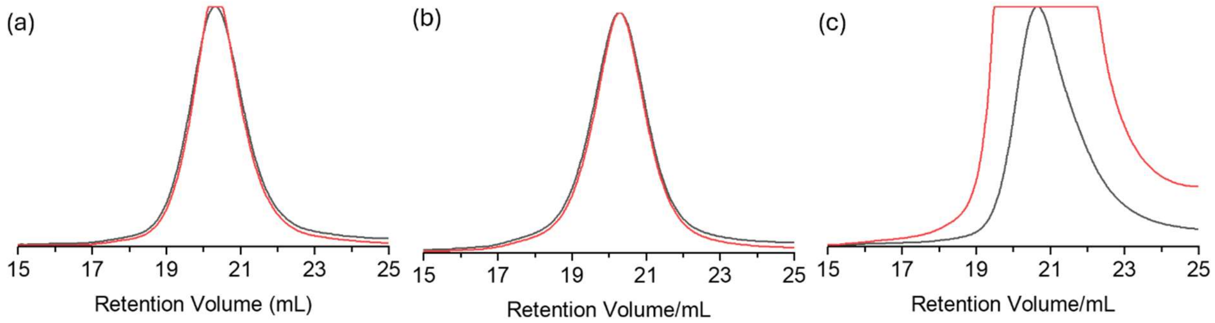


**Figure S29.** GPC chromatograms measured with dRI (black trace) and UV-vis (red trace, monitored at 280 nm) detectors for **P1-P3**. (a) **P1**; (b) **P2**; (c) **P3**.

**General Procedure B for the Synthesis of Poly(Methyl Acrylate) (PMA) Polymers Incorporating a Terminal Azide**

A flame-dried Schlenk flask equipped with a stir bar was charged with 8-azidooctyl 2-bromo-2-methylpropanoate (initiator), Tris[2-(dimethylamino)ethyl]amine ($Me_6TREN$), DMSO and methyl acrylate. The flask was sealed, and the solution was degassed via three freeze-pump-thaw cycles, then backfilled with $N_2$ and 1 cm of 20-gauge copper wire was added. After 2 more cycles of freeze-pump-thaw, the flask was back filled with $N_2$ and warmed to room temperature. And the reaction was stirred at room temperature for 1 h. Upon completion of polymerization, the flask was opened to atmosphere and diluted with a minimal amount of DCM. The polymer was precipitated (3x) into methanol cooled with dry ice and then dried under vacuum. $Mn^{NMR}$ was used to guide the following experiments as molecular weight of polymers.

**Scheme S10.** Synthesis of PMA incorporating a terminal azide.

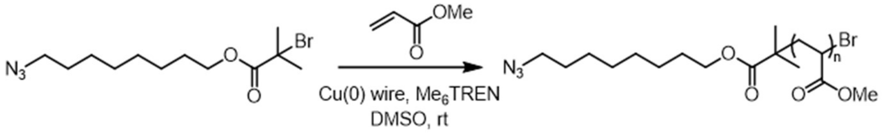


**PMA-Azide$_{35kDa}$.** Synthesized using General Procedure B with 8-azidooctyl 2-bromo-2-methylpropanoate (24 mg,0.076 mmol), $Me_6TREN$ (18 mg, 0.076 mmol), DMSO (3.4 mL) and methyl acrylate (3.27 g, 38 mmol, 3.4 mL). The title polymer was obtained as a tacky white solid (2.13 g, 65%). $Mn^{GPC}$ = 26.1 kg/mol, $Đ$ = 1.11. $Mn^{NMR}$ = 34.7 kg/mol.

**PMA-Azide$_{39kDa}$.** Synthesized using General Procedure B with 8-azidooctyl 2-bromo-2-methylpropanoate (70 mg, 0.222 mmol), $Me_6TREN$ (53 mg, 0.222 mmol), DMSO (10 mL) and methyl acrylate (9.6 g, 112 mmol, 10 mL). The title polymer was obtained as a tacky white solid (6.12 g, 64%). $Mn^{GPC}$ = 31.6 kg/mol, $Đ$ = 1.19. $Mn^{NMR}$ = 39.3 kg/mol.

**PMA-Azide$_{75kDa}$.** Synthesized using General Procedure B with 8-azidooctyl 2-bromo-2-methylpropanoate (24 mg,0.076 mmol), $Me_6TREN$ (18 mg, 0.076 mmol), DMSO (6.8 mL) and methyl acrylate (6.5 g, 76 mmol, 6.8 mL). The title polymer was obtained as a tacky white solid (3.7 g, 57%). $Mn^{GPC}$ = 37.6 kg/mol, $Đ$ = 1.13. $Mn^{NMR}$ = 74.6 kg/mol.

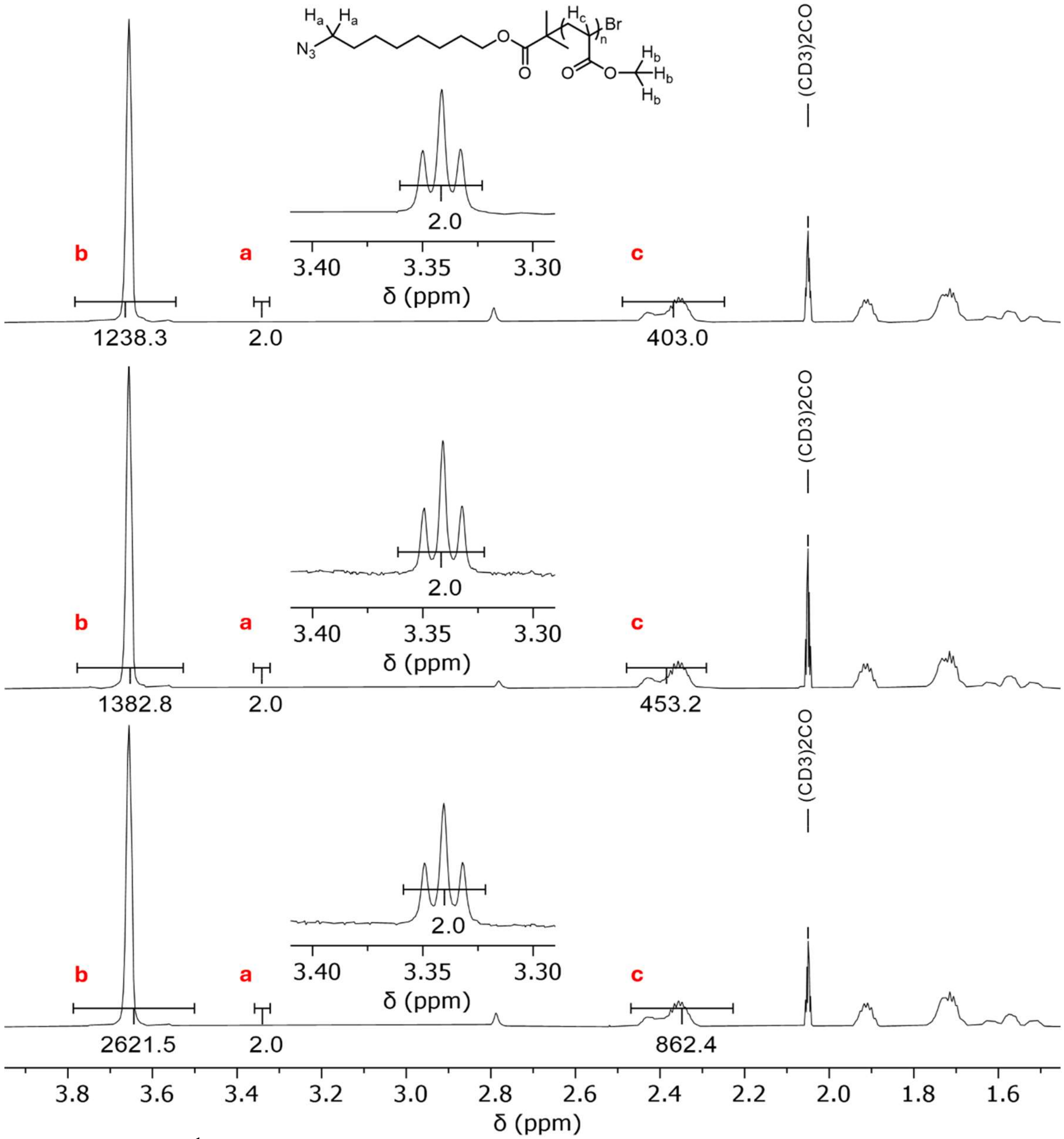


**Figure S30.** Partial $^{1}$H NMR spectra of (a) **PMA-Azide**$_{\mathbf{35kDa}}$ (800 MHz, Acetone-$d_6$). The PMA's degree of polymerization (DP) was estimated to be 404; (a) **PMA-Azide**$_{\mathbf{39kDa}}$ (800 MHz, Acetone-$d_6$). The PMA's DP was estimated to be 457; (c) **PMA-Azide**$_{\mathbf{75kDa}}$ (800 MHz, Acetone-$d_6$). The PMA's DP was estimated to be 868. Peaks a, b, and c correspond to protons in the polymer structure shown above.

## General Procedure C for Tethering Polymers to Mechanophores via Cu-catalyzed Azide-alkyne Cycloaddition (CuAAC).

Cu-catalyzed azide-alkyne cycloaddition (CuAAC) reactions were conducted following published protocols (*Polym. Chem.* **2023**, *14*, 2717-2723). A flame-dried Schlenk flask equipped with a stir bar was charged with the alkyne, pentamethyldiethylenetriamine (PMDETA) and **PMA-Azide**.

Additional THF was introduced to achieve a final polymer concentration of 50 mg/mL. The solution was deoxygenated via three freeze-pump-thaw cycles, followed by the addition of copper(I) bromide. The solution was stirred at room temperature under $N_2$ for 72 h, filtered through a plug of basic alumina, and concentrated under reduced pressure. The crude polymer was precipitated three times into cold methanol and then dried under vacuum.

**Scheme S11.** Synthesis of **PMA1-PMA3** polymers via CuAAC.

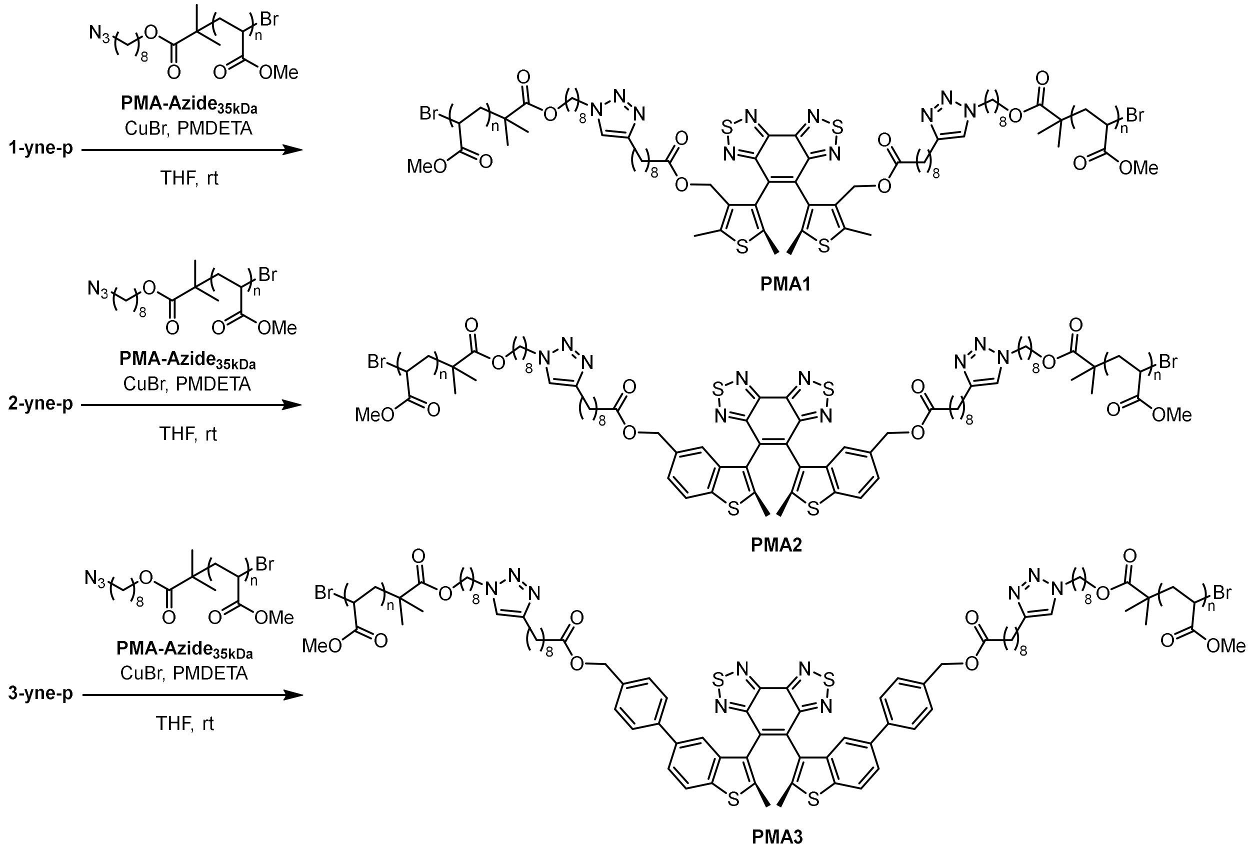


**PMA1.** Synthesized using General Procedure C with **1-yne-p** (5.0 mg, 6.2 µmol), **PMA-Azide$_{35kDa}$** (476 mg, 0.014 mmol), PMDETA (11 mg, 0.063 mmol), CuBr (5.4 mg, 0.037 mmol). The title polymer was obtained as a tacky pale-yellow solid (280 mg, 66%). $Mn^{GPC}$ = 41.9 kg/mol, $Đ$ =1.18.

**PMA2.** Synthesized using General Procedure C with **2-yne-p** (5.0 mg, 5.7 µmol), **PMA-Azide$_{35kDa}$** (436 mg, 0.013 mmol), PMDETA (10 mg, 0.057 mmol), CuBr (5 mg, 0.035 mmol). The title polymer was obtained as a tacky yellow solid (265 mg, 67%). $Mn^{GPC}$ = 42.8 kg/mol, $Đ$ = 1.16.

**PMA3.** Synthesized using General Procedure C with **3-yne-p** (5.0 mg, 4.9 µmol), **PMA-Azide$_{35kDa}$** (372 mg, 0.0107 mmol), PMDETA (8.5 mg, 0.049 mmol), CuBr (4.2 mg, 0.029 mmol) The title polymer was obtained as a tacky yellow solid (232 mg, 68%). $Mn^{GPC}$ = 40.9 kg/mol, $Đ$ =1.16.

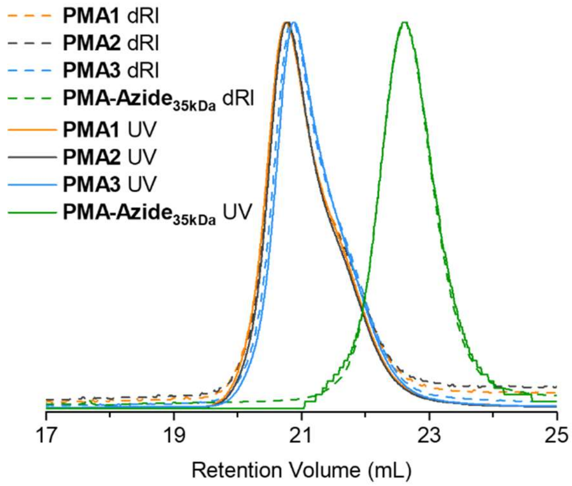


**Figure S31.** GPC chromatograms of **PMA1-PMA3** and **PMA-Azide$_{35kDa}$** measured with dRI and UV-vis (monitored at 254 nm or 280 nm) detectors. Normalized dRI signal (dashed): **PMA1** (orange), **PMA2** (dark grey), **PMA3** (light blue), **PMA-Azide$_{35kDa}$** (green); Normalized UV signal (solid traces), **PMA1** (orange, 280 nm), **PMA2** (dark grey, 280 nm), **PMA3** (light blue, 280 nm), **PMA-Azide$_{35kDa}$** (green, 254 nm).

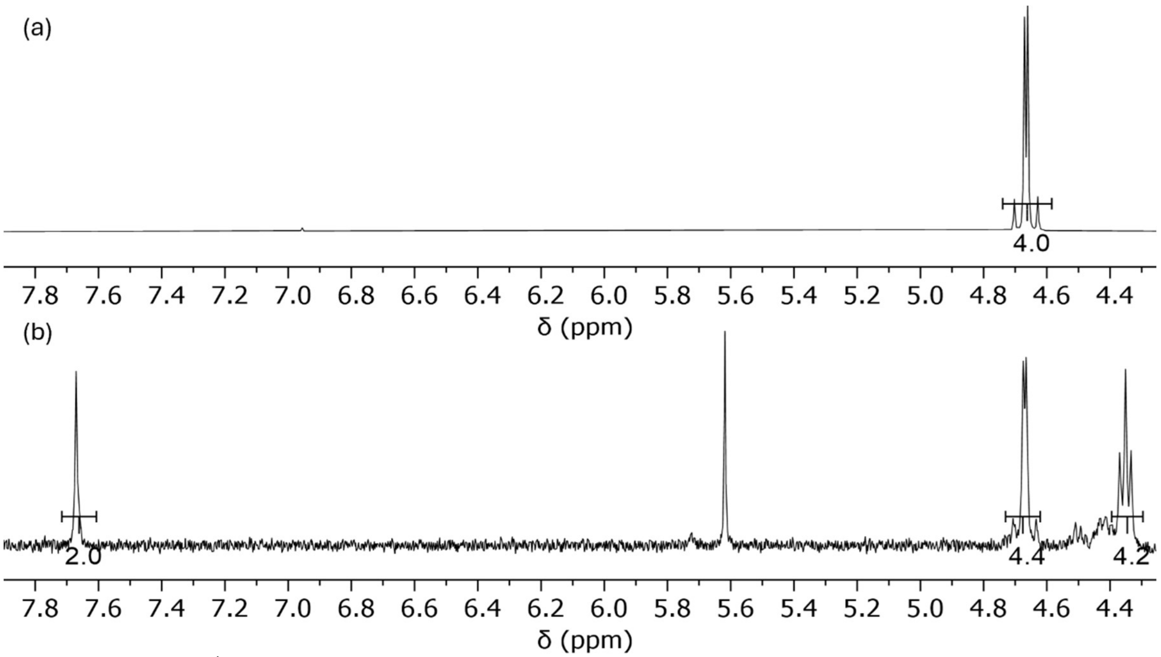


**Figure S32.** Partial $^{1}$H NMR (400 MHz, Acetone-$d_6$) spectra of (a) small molecule bis-alkyne **1-yne-p**, and (b) polymer **PMA1** demonstrating successful coupling between the diarylethene unit and the polymer chains. A new singlet (7.67 ppm, 2H) is observed, corresponding to the triazole structure in the product.

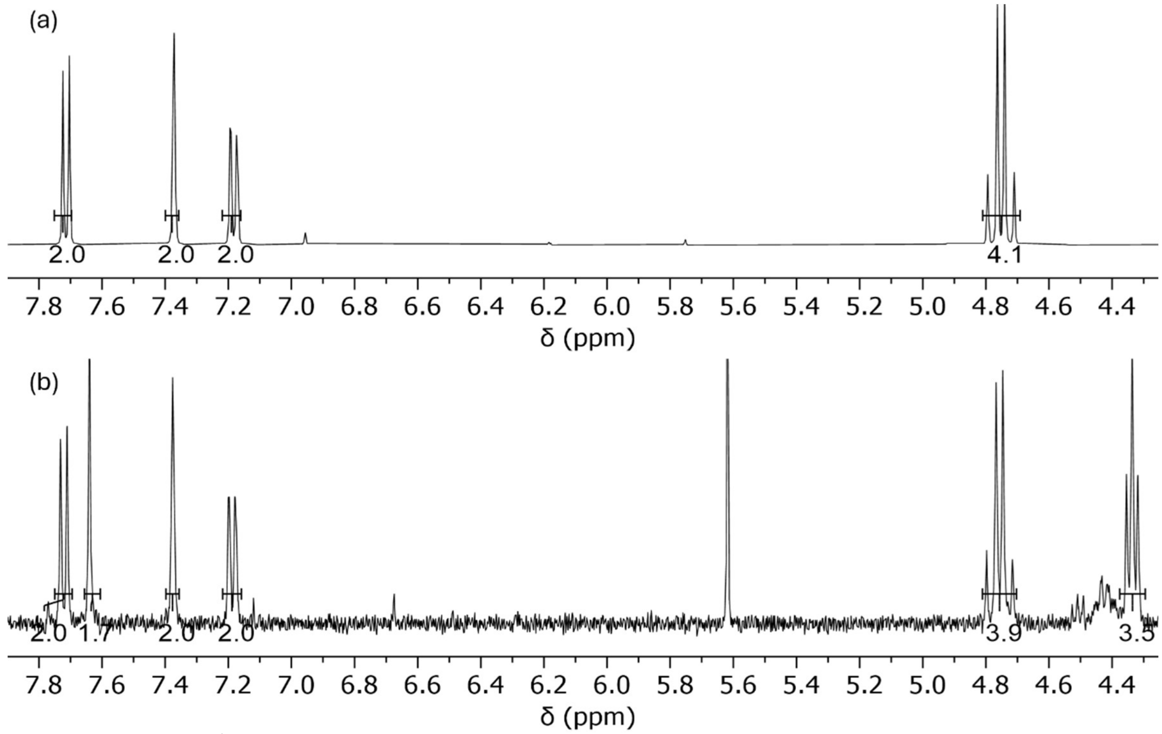


**Figure S33.** Partial $^{1}$H NMR (400 MHz, Acetone-$d_6$) spectra of (a) small molecule bis-alkyne **2-yne-p**, and (b) polymer **PMA2** demonstrating successful coupling between the diarylethene unit and the polymer chains. A new singlet (7.64 ppm, 2H) is observed, corresponding to the triazole structure in the product.

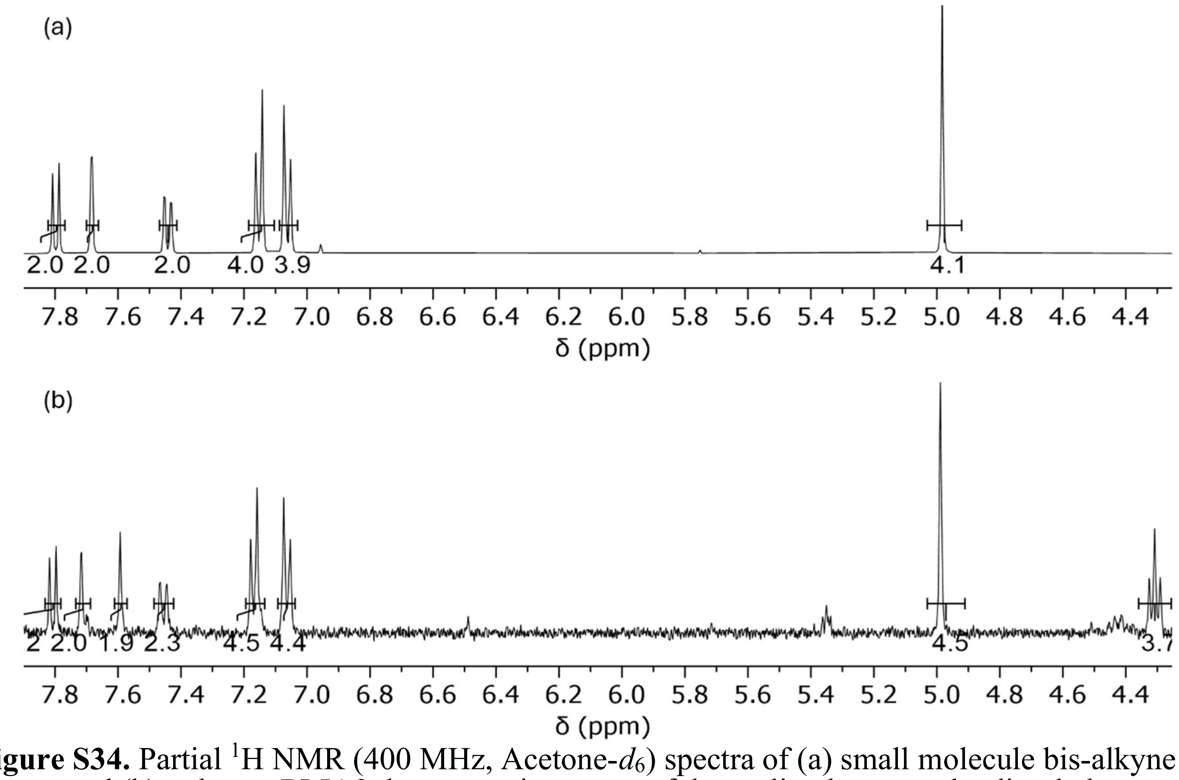


**Figure S34.** Partial $^{1}$H NMR (400 MHz, Acetone-$d_6$) spectra of (a) small molecule bis-alkyne **3-yne-p**, and (b) polymer **PMA3** demonstrating successful coupling between the diarylethene unit

and the polymer chains. A new singlet (7.59 ppm, 2H) is observed, corresponding to the triazole structure in the product.

**Scheme S12.** Synthesis of **PMAap** polymers via CuAAC.

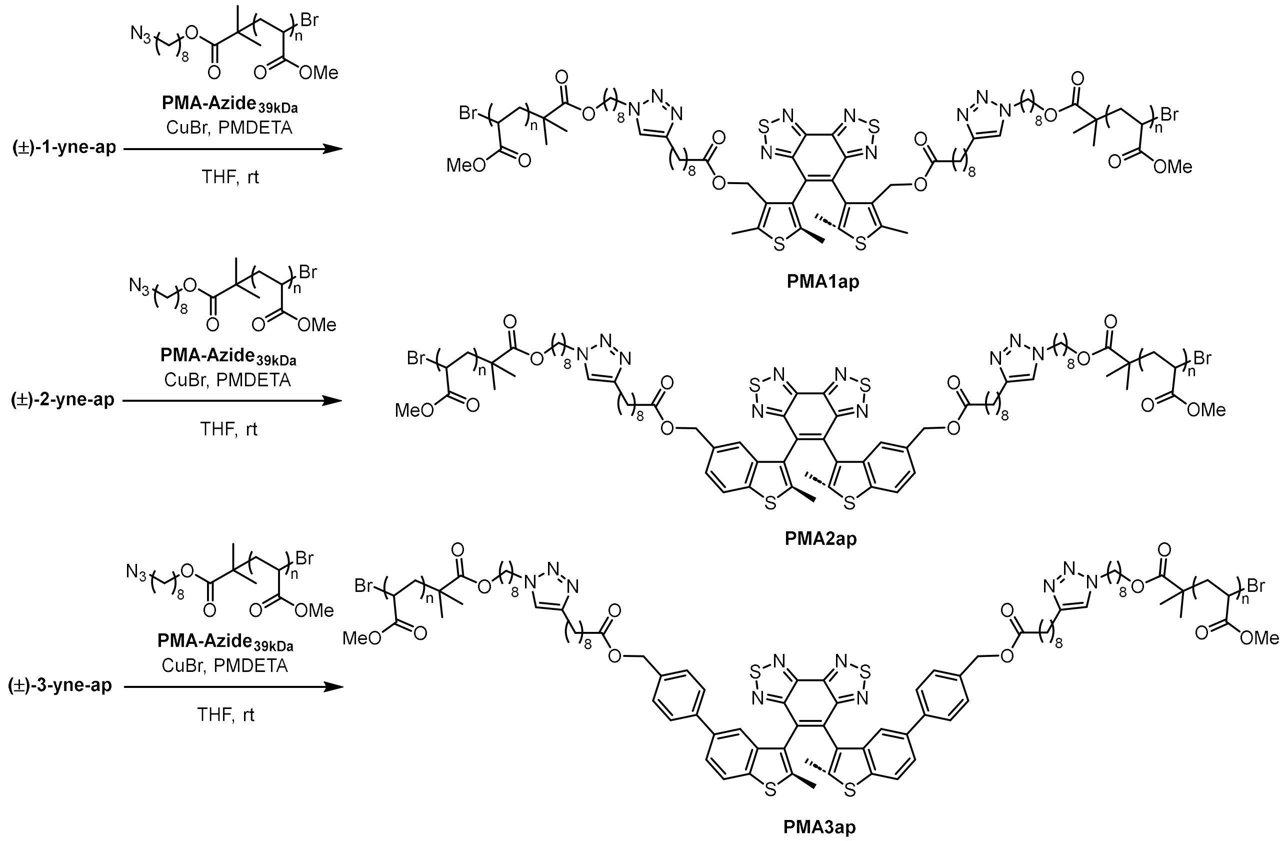


**PMA1ap.** Synthesized using General Procedure C with **(±)-1-yne-ap** (4.6 mg, 5.7 µmol), **PMA-Azide$_{39kDa}$** (470 mg, 12 µmol), PMDETA (10 mg, 0.057 mmol), CuBr (5 mg, 0.035 mmol). The title polymer was obtained as a tacky orange solid (280 mg, 64%). Mn$^{GPC}$ = 38.9 kg/mol, *Đ* =1.28.

**PMA2ap.** Synthesized using General Procedure C with **(±)-2-yne-ap** (3.9 mg, 4.5 µmol), **PMA-Azide$_{39kDa}$** (370 mg, 9.4 µmol), PMDETA (8 mg, 0.045 mmol), CuBr (4 mg, 0.028 mmol). The title polymer was obtained as a tacky red solid (265 mg, 63%). Mn$^{GPC}$ = 39.7 kg/mol, *Đ* = 1.23.

**PMA3ap.** Synthesized using General Procedure C with **(±)-3-yne-ap** (4.6 mg, 4.5 µmol), **PMA-Azide$_{39kDa}$** (370 mg, 9.4 µmol), PMDETA (8 mg, 0.045 mmol), CuBr (4 mg, 0.028 mmol) The title polymer was obtained as a tacky red solid (232 mg, 66%). Mn$^{GPC}$ = 38.5 kg/mol, *Đ* =1.22.

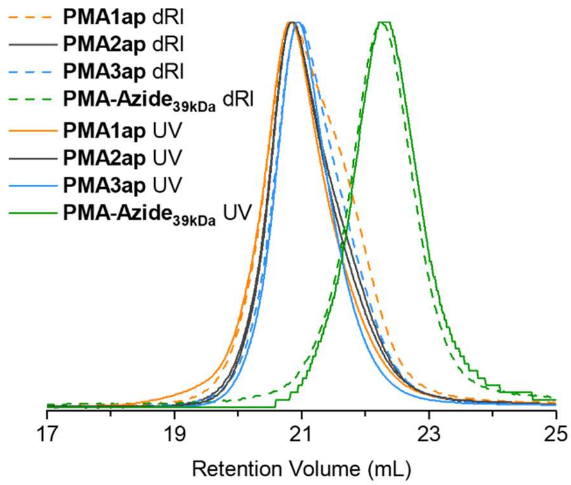


**Figure S35.** GPC chromatograms of **PMAap** polymers and **PMA-Azide$_{35kDa}$** measured with dRI and UV-vis (monitored at 254 nm or 280 nm) detectors. Normalized dRI signal (dashed): **PMA1ap** (orange), **PMA2ap** (dark grey), **PMA3ap** (light blue), **PMA-Azide$_{39kDa}$** (green); Normalized UV signal (solid traces), **PMA1ap** (orange, 280 nm), **PMA2ap** (dark grey, 280 nm), **PMA3ap** (light blue, 280 nm), **PMA-Azide$_{39kDa}$** (green, 254 nm).

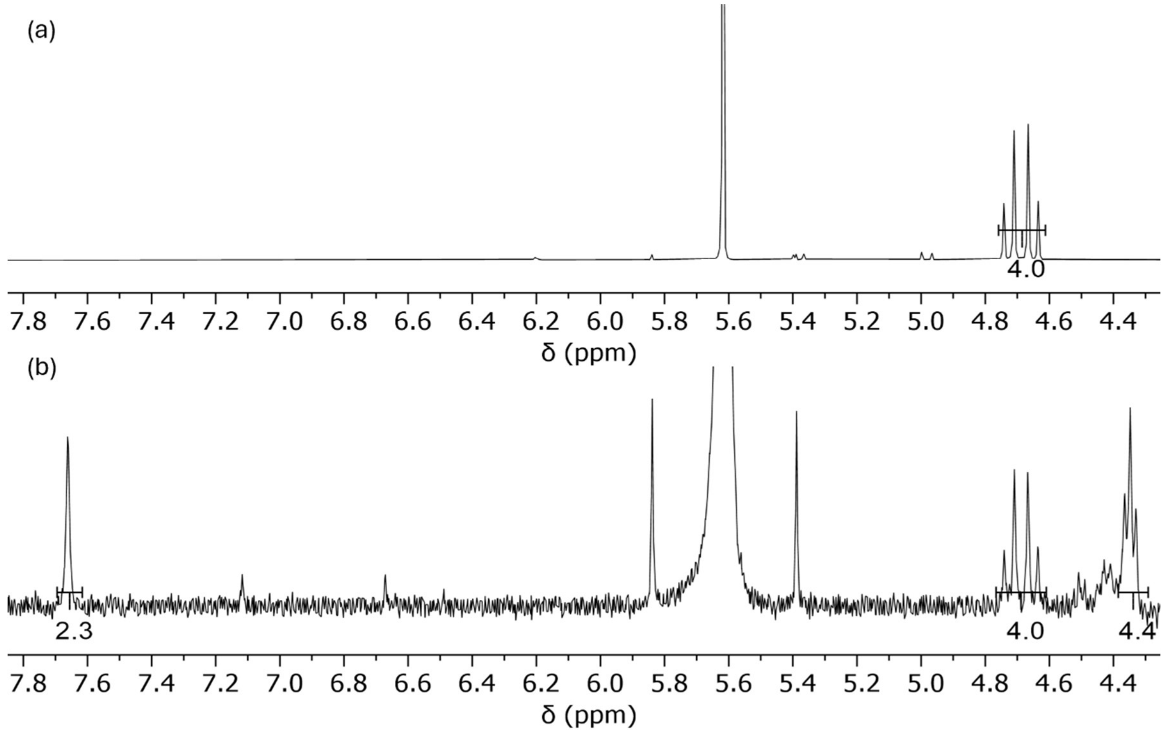


**Figure S36.** Partial $^{1}$H NMR (400 MHz, Acetone-$d_6$) spectra of (a) small molecule bis-alkyne **(±)-1-yne-ap**, and (b) polymer **PMA1ap** demonstrating successful coupling between the diarylethene unit and the polymer chains. A new singlet (7.66 ppm, 2H) is observed, corresponding to the triazole structure in the product.

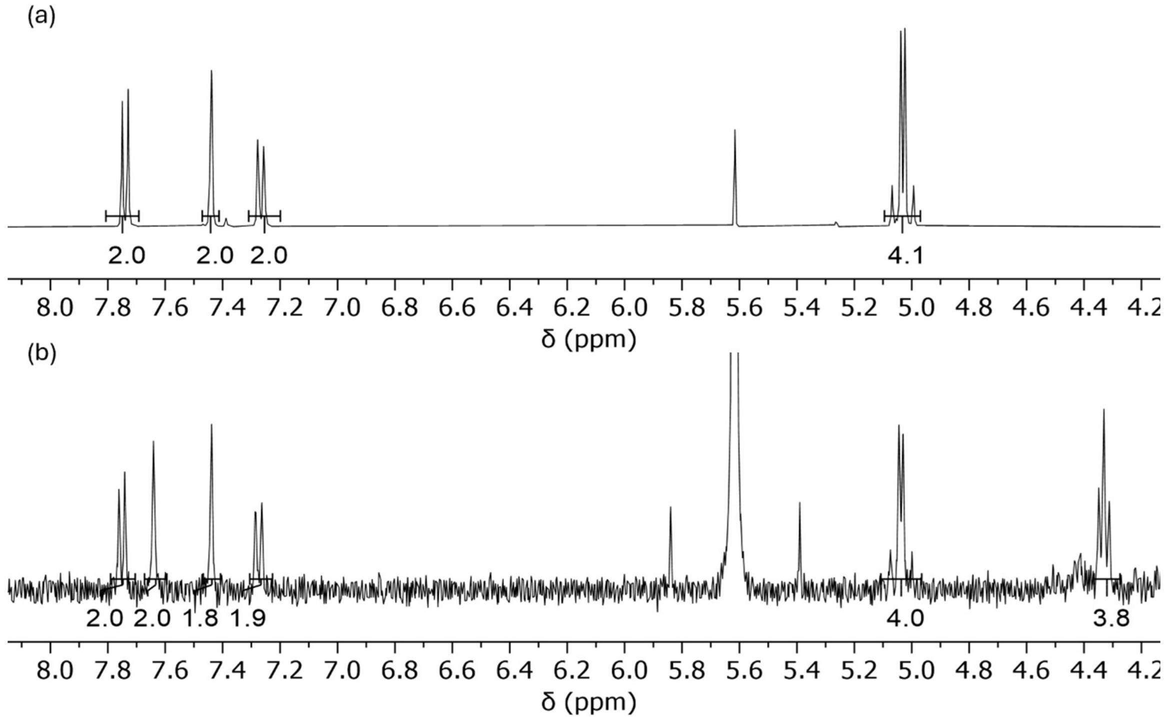


**Figure S37.** Partial $^{1}H$ NMR (400 MHz, Acetone-$d_6$) spectra of (a) small molecule bis-alkyne **(±)-2-yne-ap**, and (b) polymer **PMA2ap** demonstrating successful coupling between the diarylethene unit and the polymer chains. A new singlet (7.64 ppm, 2H) is observed, corresponding to the triazole structure in the product.

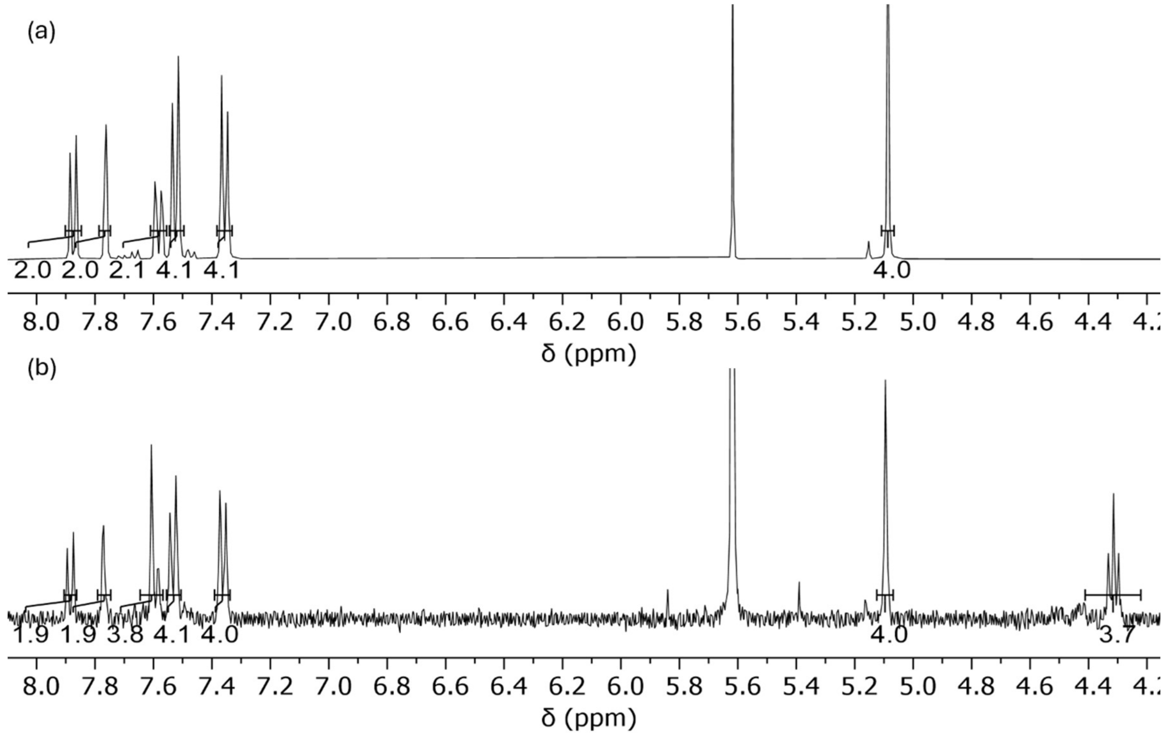


**Figure S38.** Partial $^{1}H$ NMR (400 MHz, Acetone-$d_6$) spectra of (a) small molecule bis-alkyne **(±)-3-yne-ap**, and (b) polymer **PMA3ap** demonstrating successful coupling between the

diarylethene unit and the polymer chains. A new singlet (7.64 ppm, 2H) is observed, corresponding to the triazole structure in the product.

**Scheme S13.** Synthesis of **PMA-control** polymers via CuAAC**.**

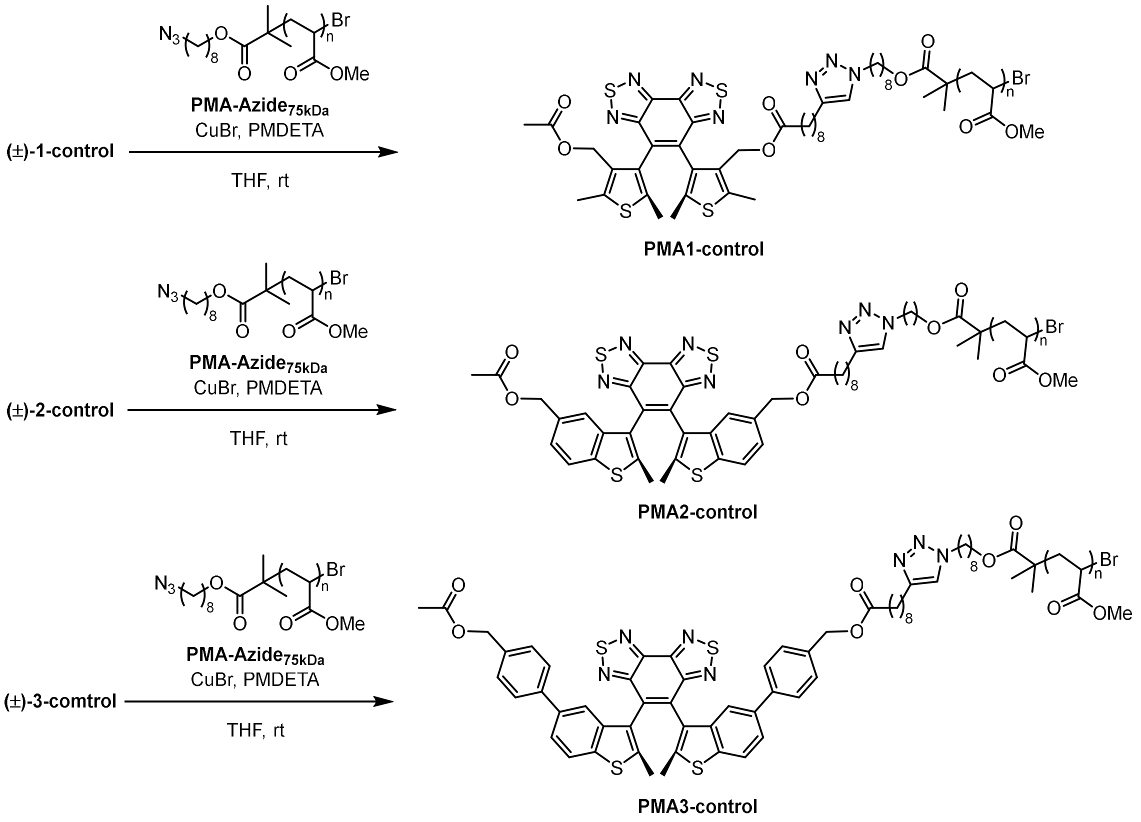


**PMA1-control.** Synthesized using General Procedure C with **(±)-1-control** (3.9 mg, 5.7 μmol), **PMA-Azide$_{75kDa}$** (354 mg, 4.75 μmol), PMDETA (10 mg, 0.057 mmol), CuBr (5 mg, 0.034 mmol). The title polymer was obtained as a tacky pale-yellow solid (246 mg, 70%). $Mn^{GPC}$ = 32.6 kg/mol, $Đ$ =1.15.

**PMA2-control.** Synthesized using General Procedure C with **(±)-2-control** (4.3 mg, 5.7 μmol), **PMA-Azide$_{75kDa}$** (354 mg, 4.75 μmol), PMDETA (10 mg, 0.057 mmol), CuBr (5 mg, 0.034 mmol). The title polymer was obtained as a tacky yellow solid (238 mg, 67%). $Mn^{GPC}$ = 32.7 kg/mol, $Đ$ = 1.17.

**PMA3-control.** Synthesized using General Procedure C with **(±)-3-control** (5.2 mg, 5.7 μmol), **PMA-Azide$_{75kDa}$** (354 mg, 4.75 μmol), PMDETA (10 mg, 0.057 mmol), CuBr (5 mg, 0.034 mmol). The title polymer was obtained as a tacky yellow solid (250 mg, 71%). $Mn^{GPC}$ = 31.2 kg/mol, $Đ$ =1.13.

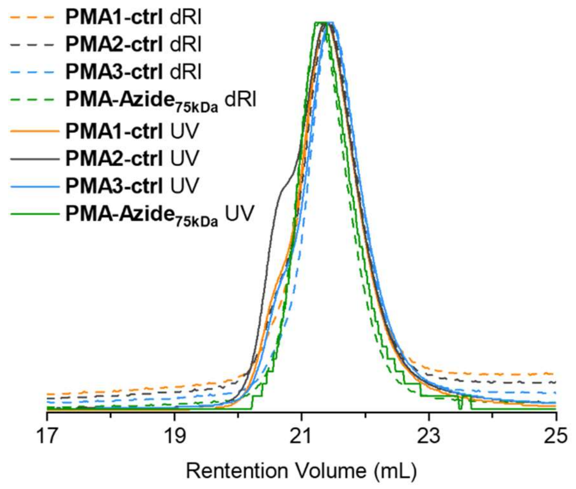


**Figure S39.** GPC chromatograms **PMA-control** polymers and **PMA-Azide$_{75kDa}$** measured with dRI and UV-vis (monitored at 254 nm or 280 nm) detectors. Normalized dRI signal (dashed traces): **PMA1-control** (orange), **PMA2-control** (dark grey), **PMA3-control** (light blue), **PMA-Azide$_{75kDa}$** (green); Normalized UV signal (solid traces): **PMA1-control** (orange, 280 nm), **PMA2-control** (dark grey, 280 nm), **PMA3-control** (light blue, 280 nm), **PMA-Azide$_{75kDa}$** (green, 254 nm).

## 8. NMR Spectra

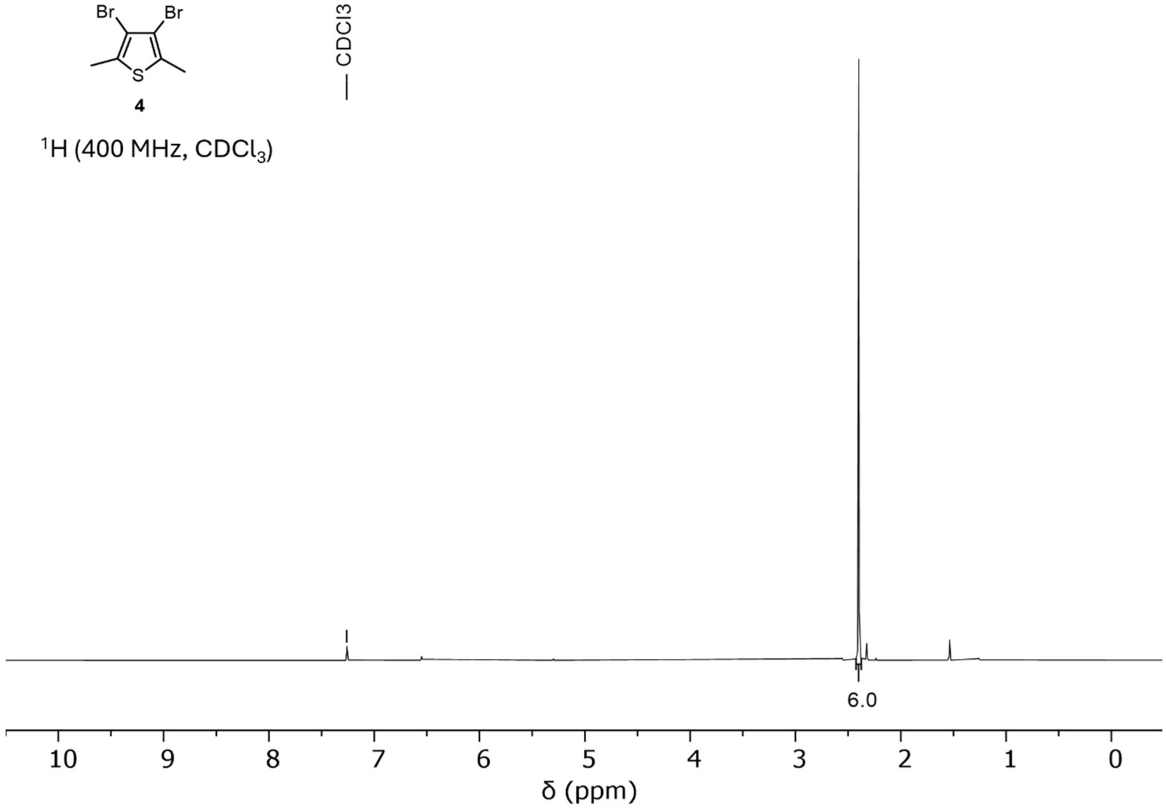


**4**

$^{1}H$ (400 MHz, $CDCl_3$)

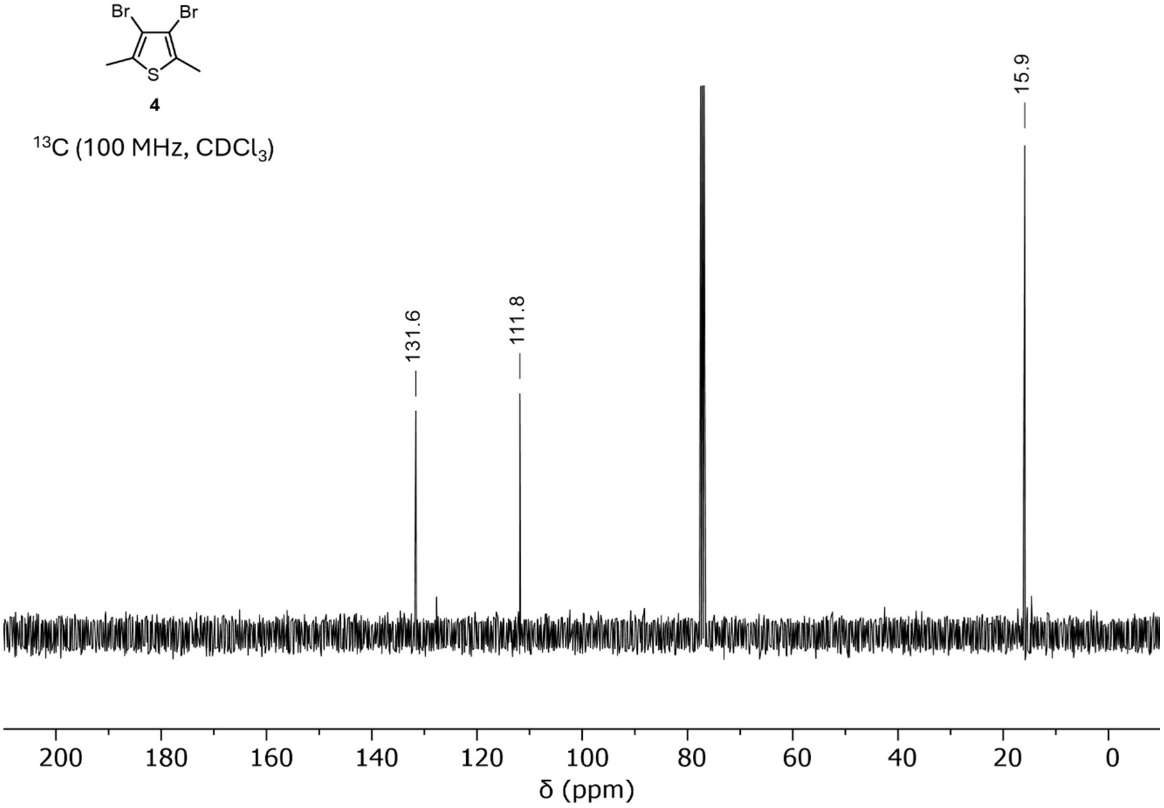


**4**

$^{13}C$ (100 MHz, $CDCl_3$)

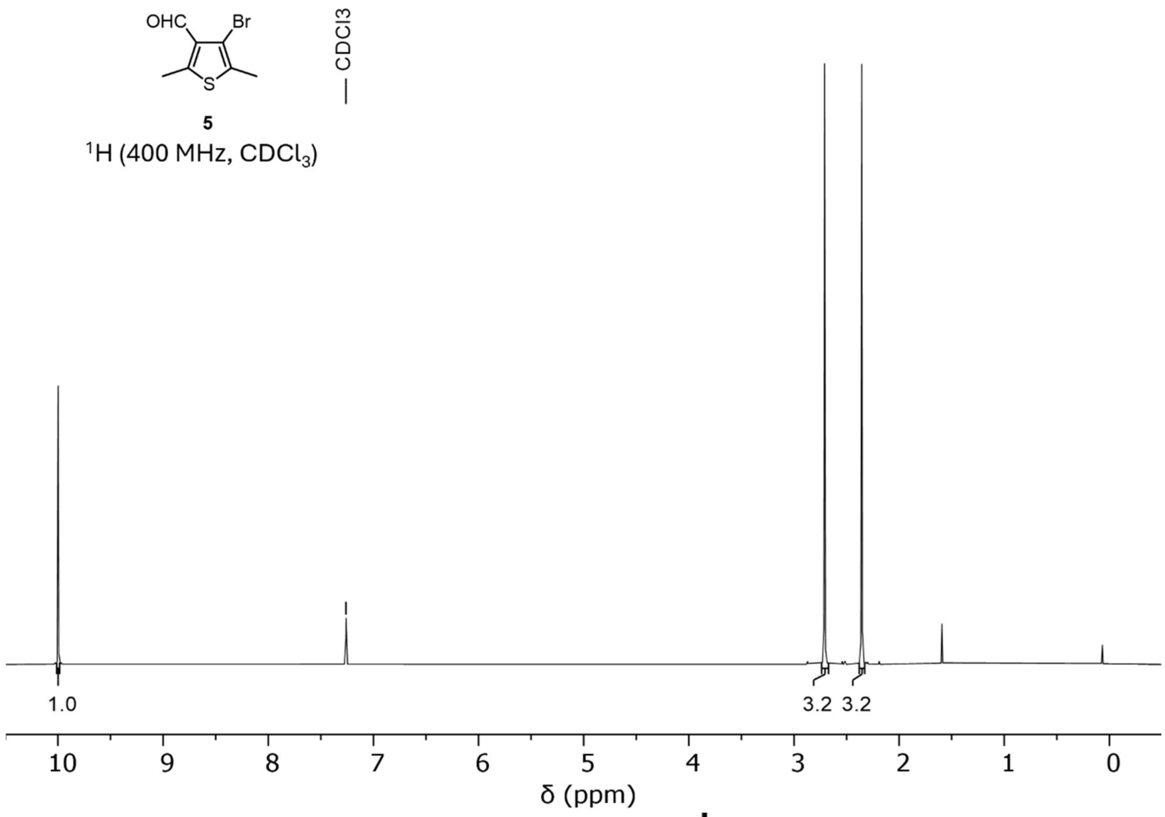

OHC
Br
S
5
CDCl3
1H (400 MHz, CDCl3)
1.0
3.2
3.2
10
9
8
7
6
5
4
3
2
1
0
δ (ppm)


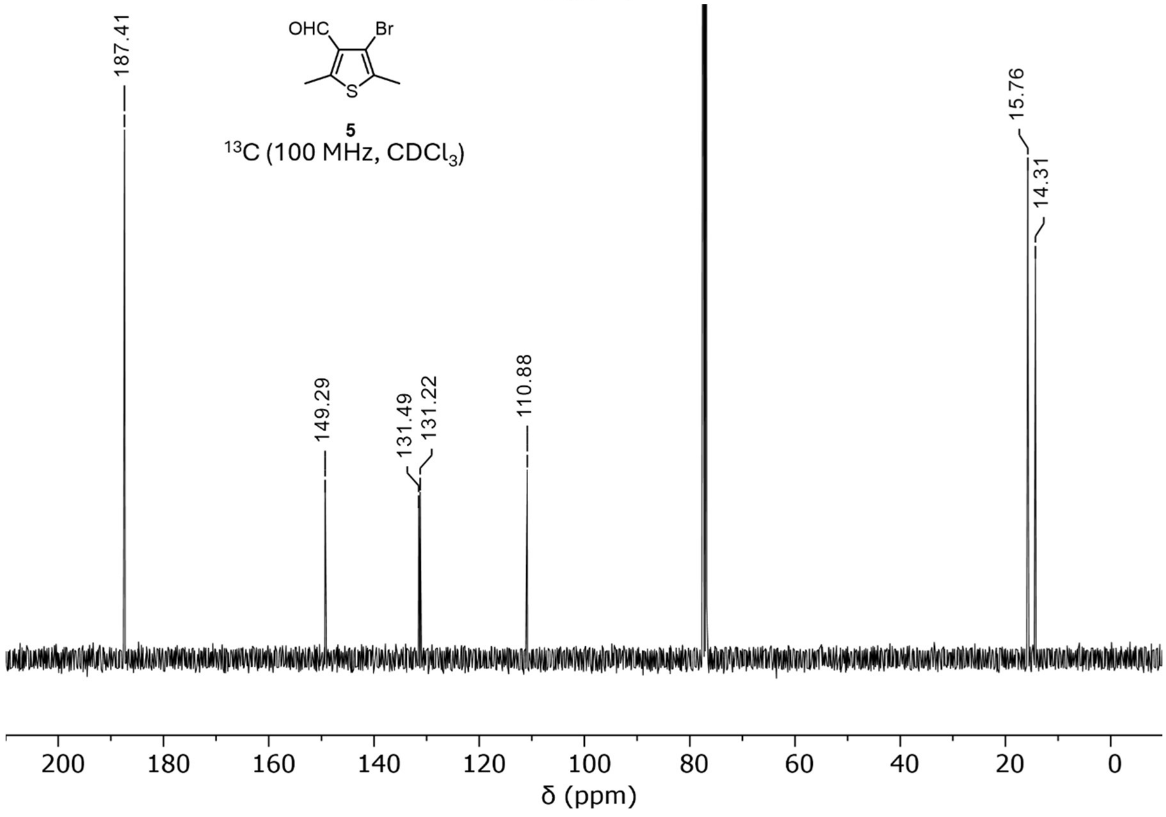

187.41
OHC
Br
S
5
13C (100 MHz, CDCl3)
15.76
14.31
149.29
131.49
131.22
110.88
200
180
160
140
120
100
80
60
40
20
0
δ (ppm)

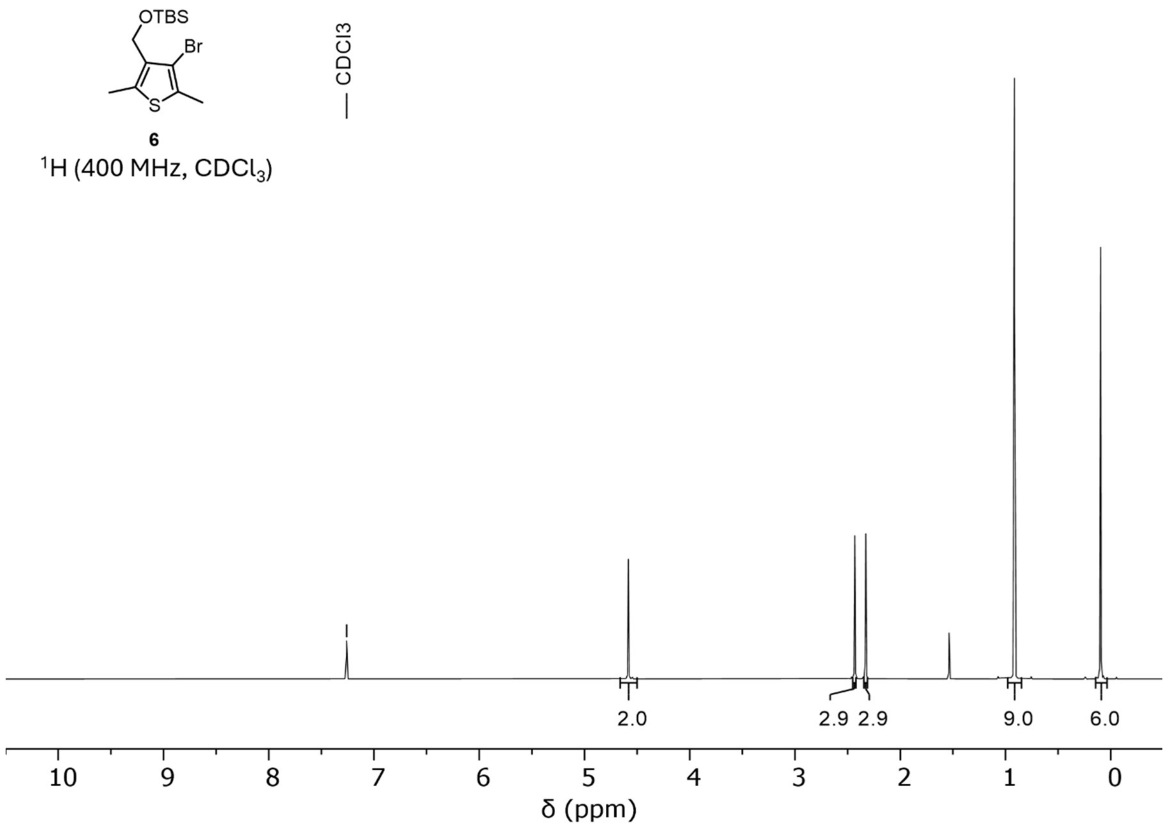
OTBS
Br
S
6
1H (400 MHz, CDCl3)
CDCl3
2.0
2.9 2.9
9.0
6.0
10 9 8 7 6 5 4 3 2 1 0
δ (ppm)

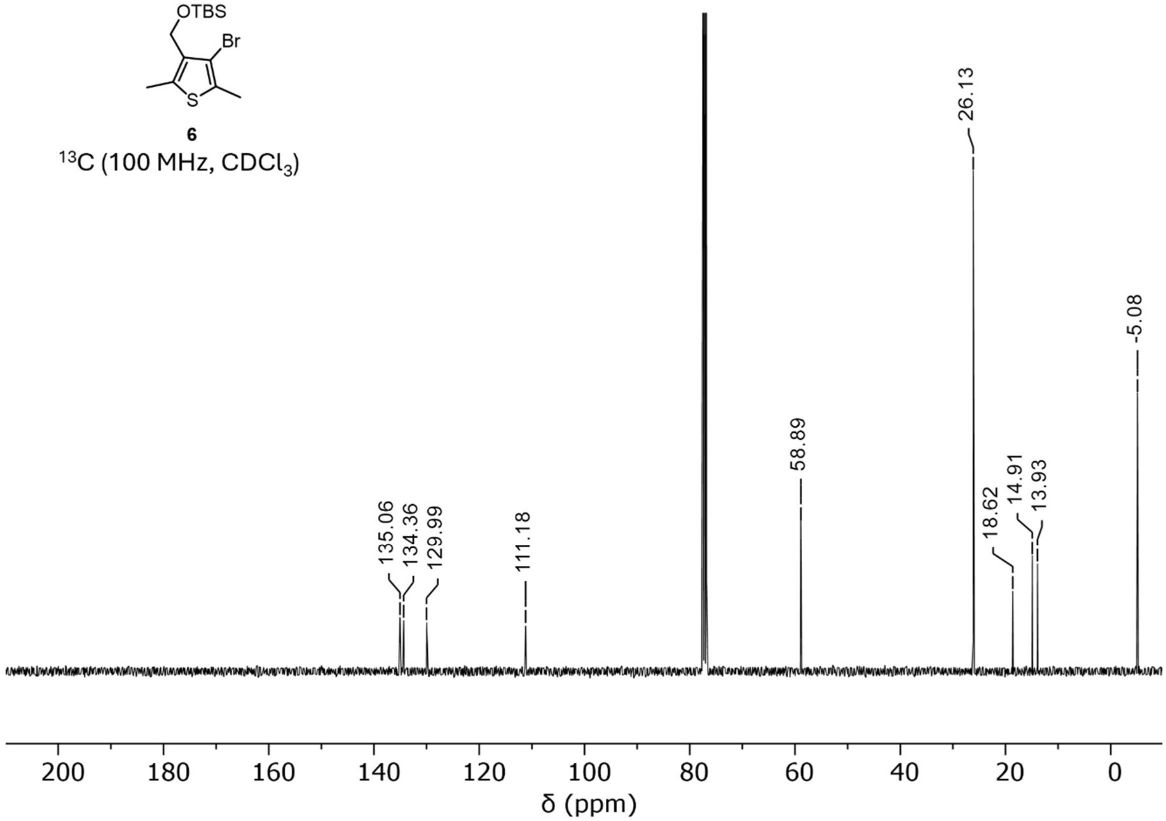
OTBS
Br
S
6
13C (100 MHz, CDCl3)
135.06
134.36
129.99
111.18
58.89
26.13
18.62
14.91
13.93
-5.08
200 180 160 140 120 100 80 60 40 20 0
δ (ppm)

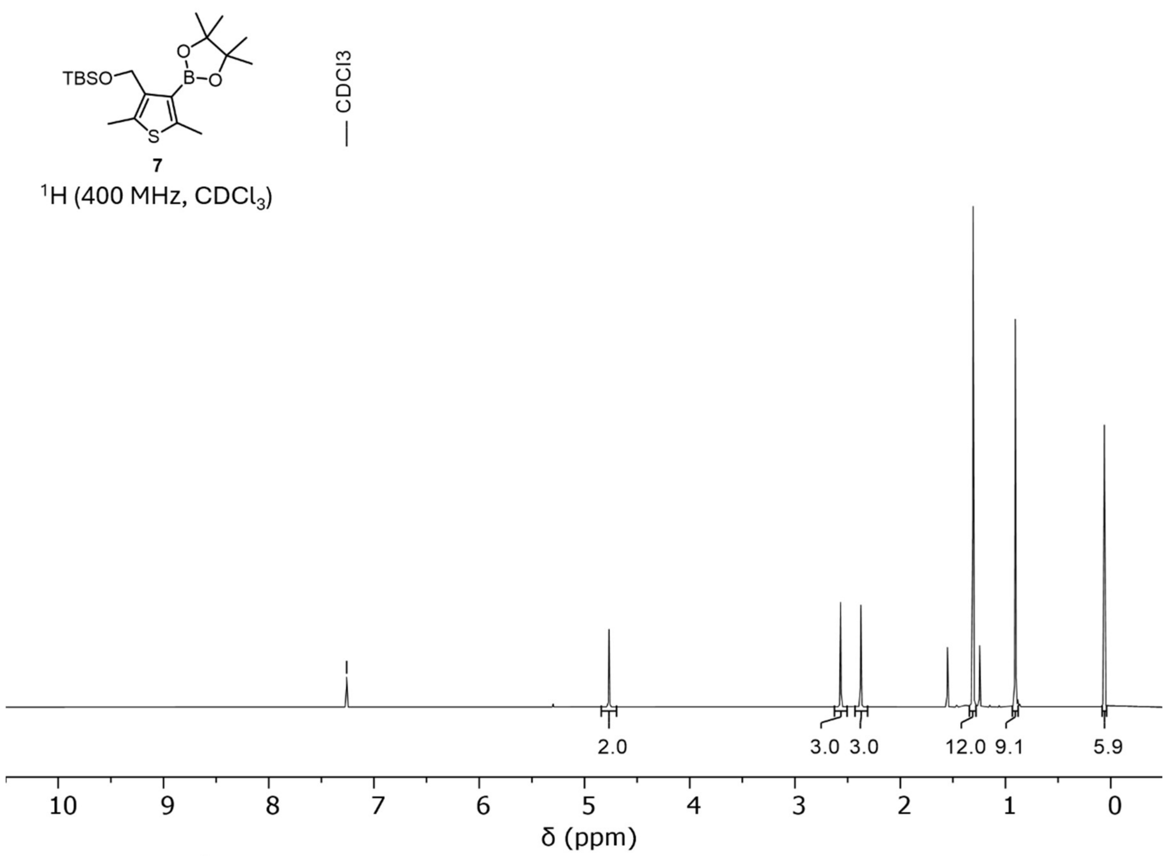
TBSO
7
¹H (400 MHz, CDCl₃)
CDCl3
2.0
3.0
3.0
12.0
9.1
5.9
10 9 8 7 6 5 4 3 2 1 0
δ (ppm)

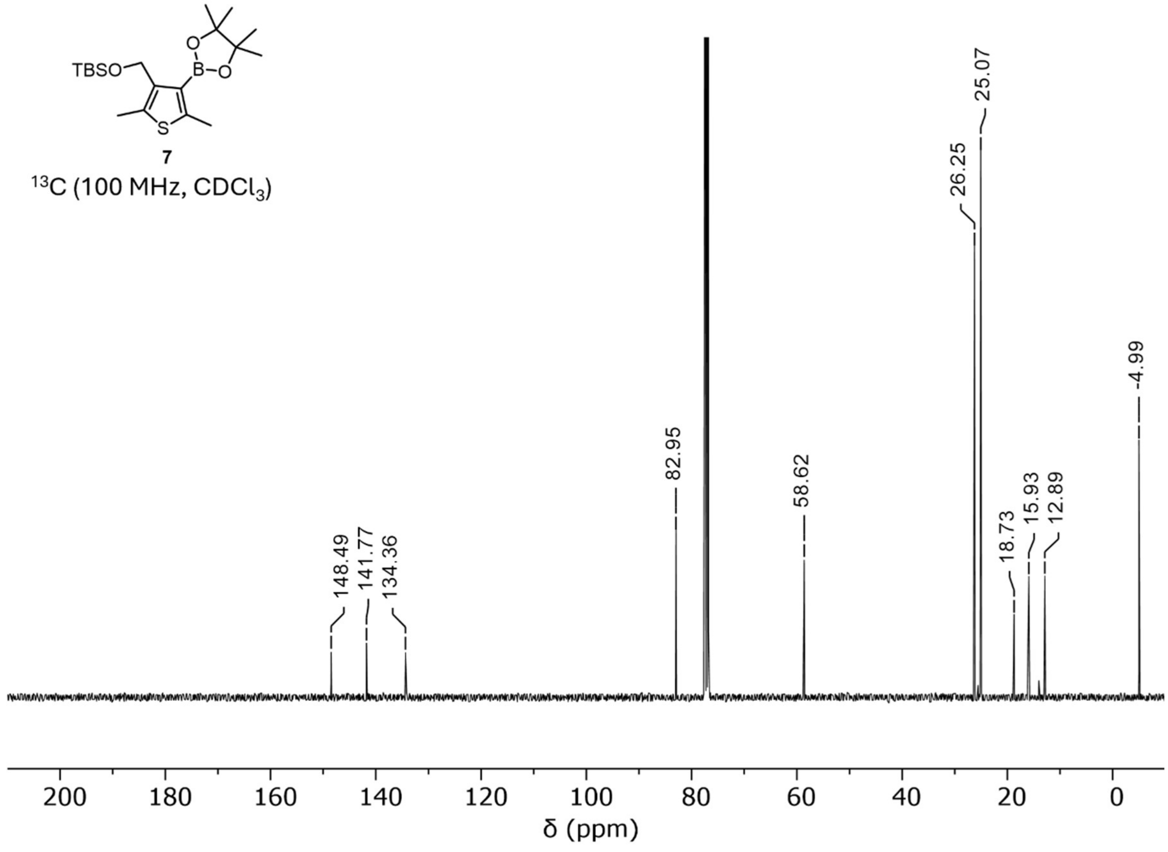
TBSO
7
¹³C (100 MHz, CDCl₃)
148.49
141.77
134.36
82.95
58.62
26.25
25.07
18.73
15.93
12.89
-4.99
200 180 160 140 120 100 80 60 40 20 0
δ (ppm)

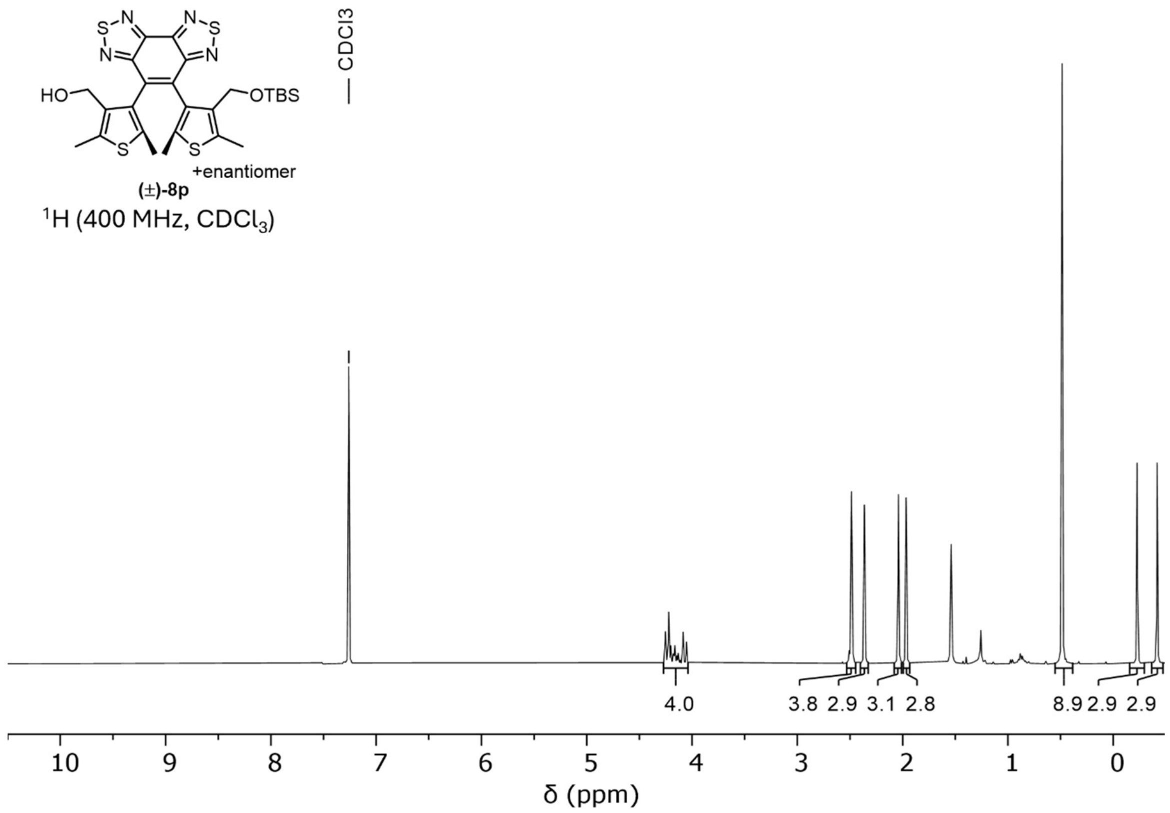
HO
OTBS
+enantiomer
(±)-8p
1H (400 MHz, CDCl3)
CDCl3
4.0
3.8 2.9 3.1 2.8
8.9 2.9 2.9
10 9 8 7 6 5 4 3 2 1 0
δ (ppm)

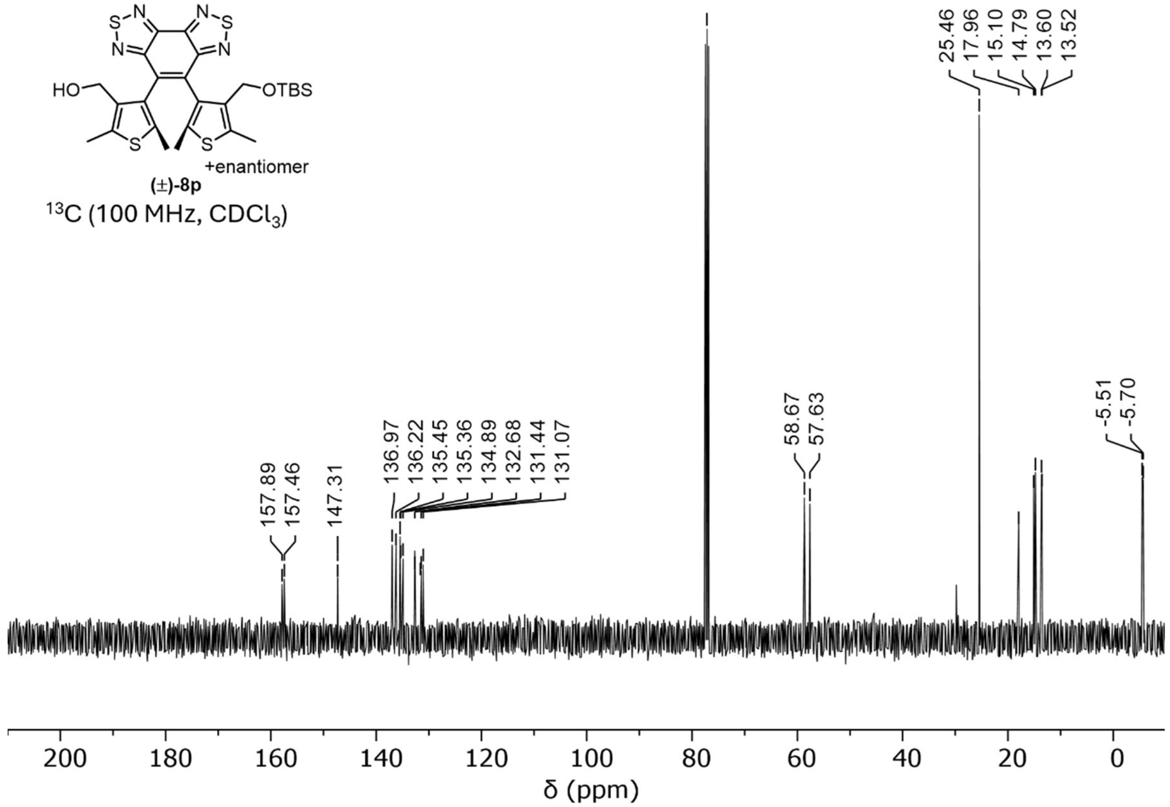
HO
OTBS
+enantiomer
(±)-8p
13C (100 MHz, CDCl3)
157.89
157.46
147.31
136.97
136.22
135.45
135.36
134.89
132.68
131.44
131.07
58.67
57.63
25.46
17.96
15.10
14.79
13.60
13.52
-5.51
-5.70
200 180 160 140 120 100 80 60 40 20 0
δ (ppm)

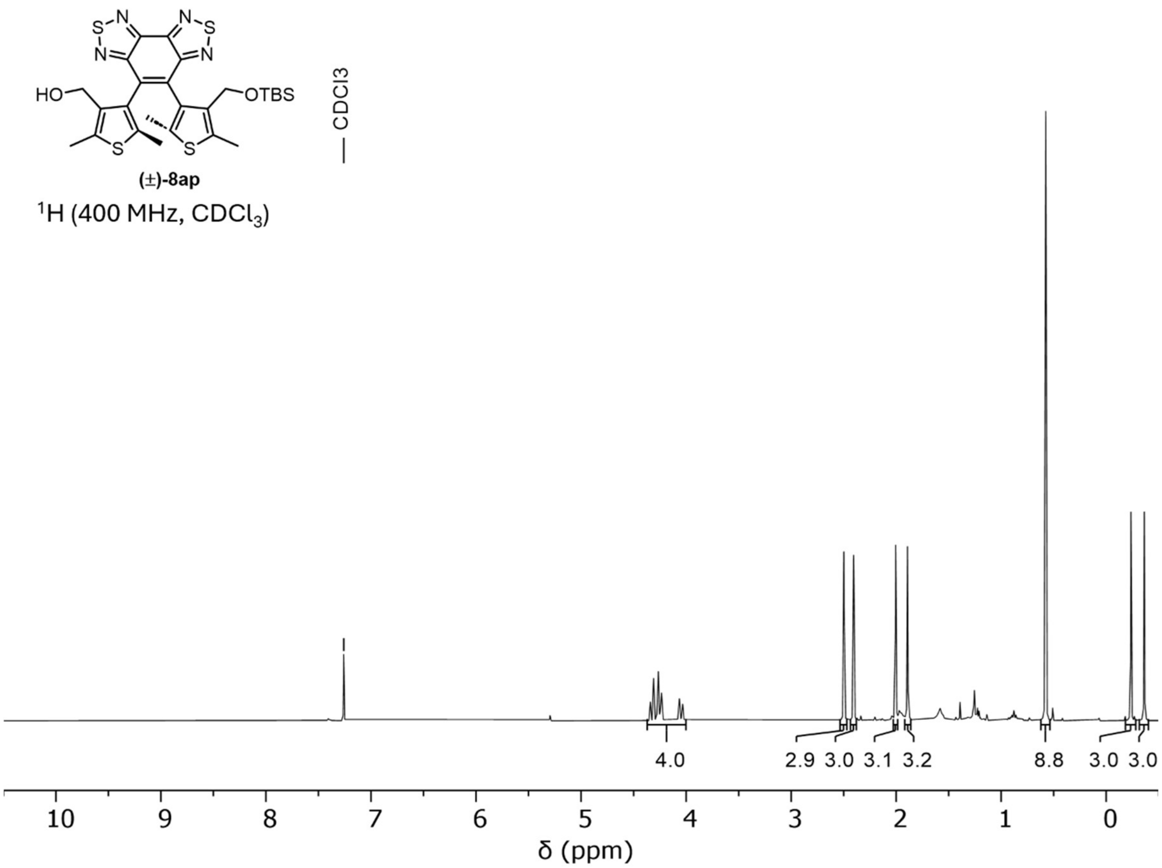
HO
OTBS
(±)-8ap
1H (400 MHz, CDCl3)
CDCl3
4.0
2.9 3.0 3.1 3.2
8.8 3.0 3.0
10 9 8 7 6 5 4 3 2 1 0
δ (ppm)

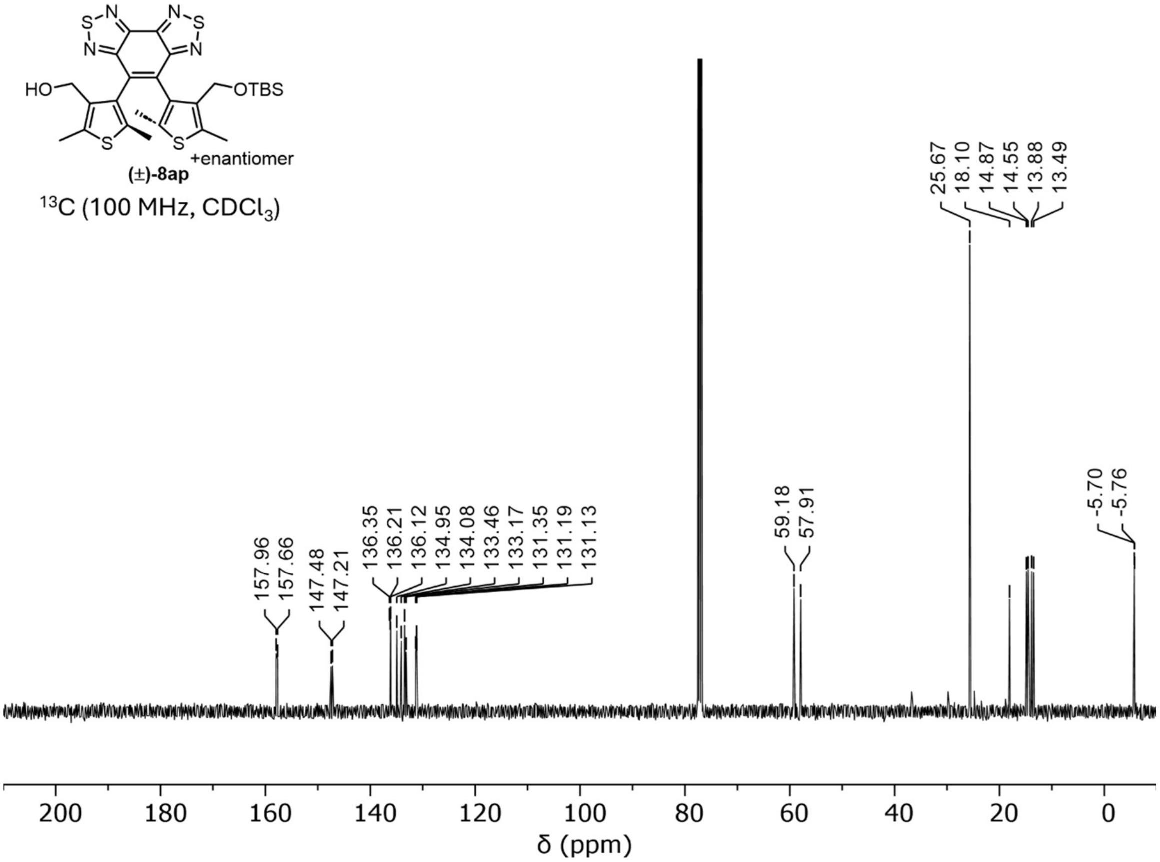
HO
OTBS
+enantiomer
(±)-8ap
13C (100 MHz, CDCl3)
157.96
157.66
147.48
147.21
136.35
136.21
136.12
134.95
134.08
133.46
133.17
131.35
131.19
131.13
59.18
57.91
25.67
18.10
14.87
14.55
13.88
13.49
-5.70
-5.76
200 180 160 140 120 100 80 60 40 20 0
δ (ppm)

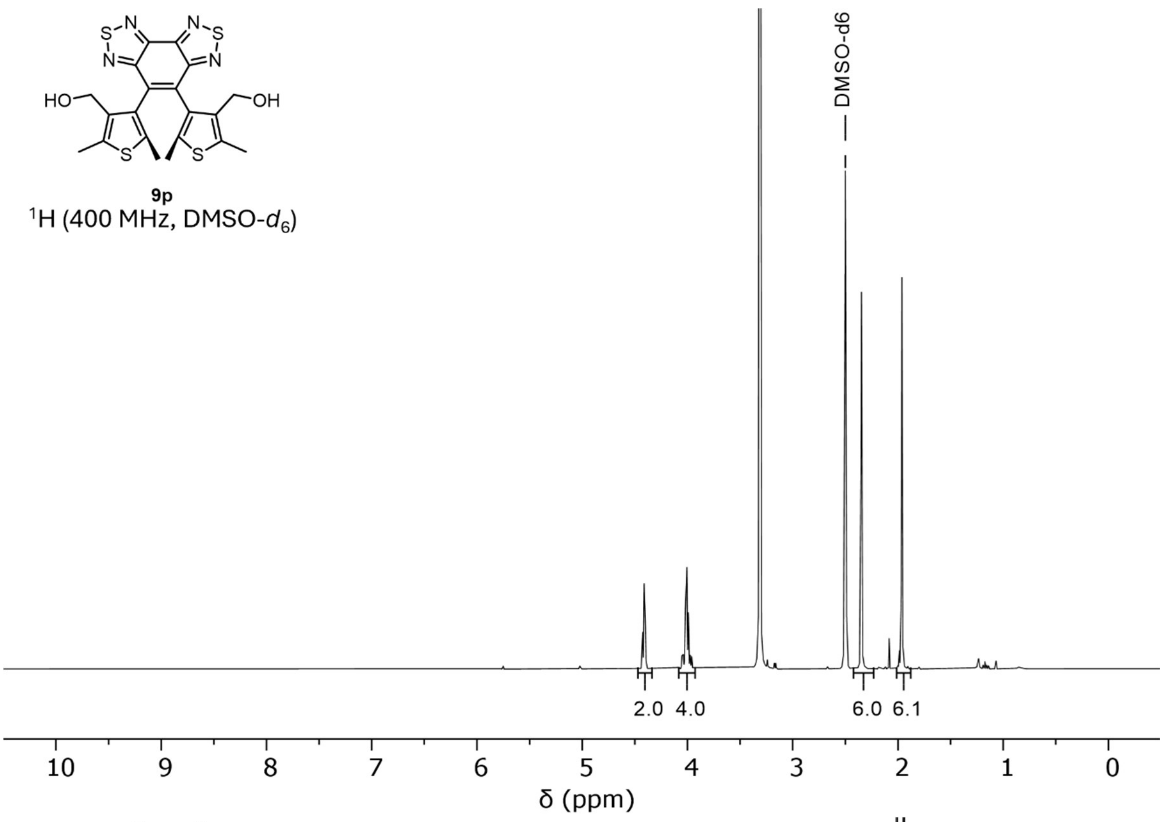


**9p**
$^{1}$H (400 MHz, DMSO-$d_6$)

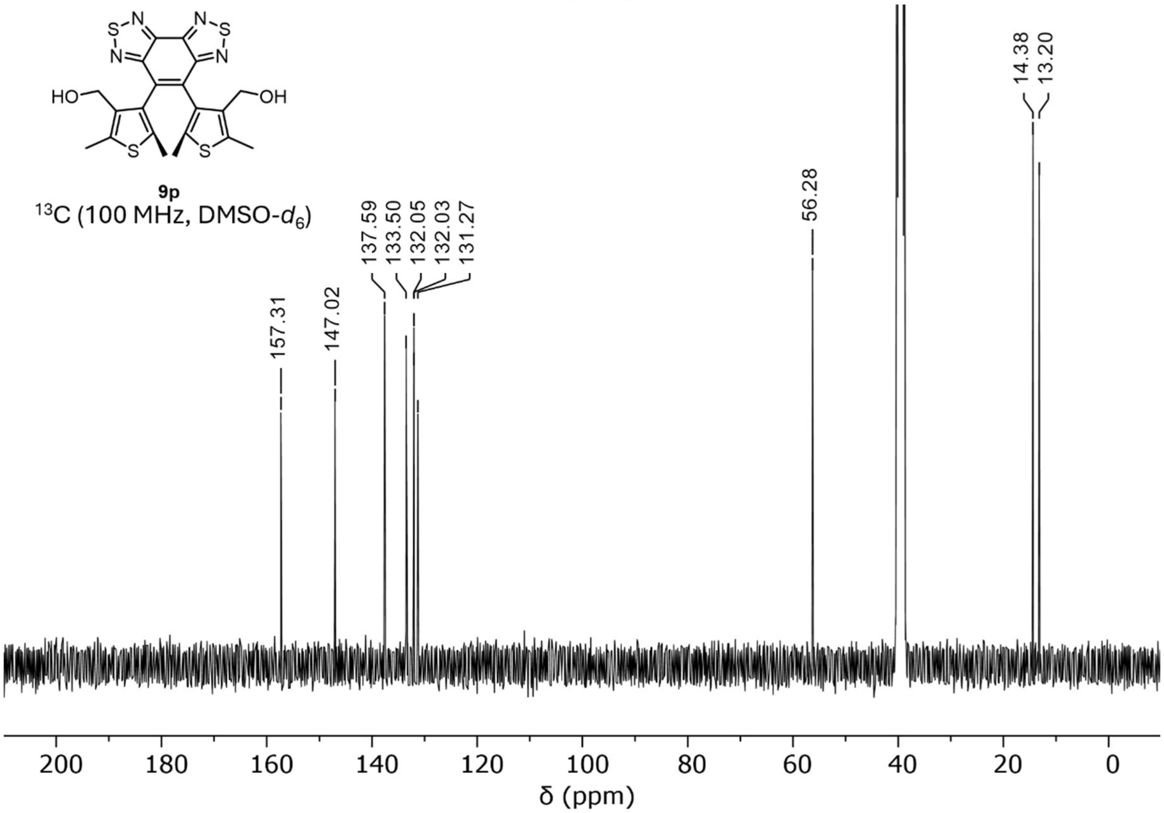


**9p**
$^{13}$C (100 MHz, DMSO-$d_6$)

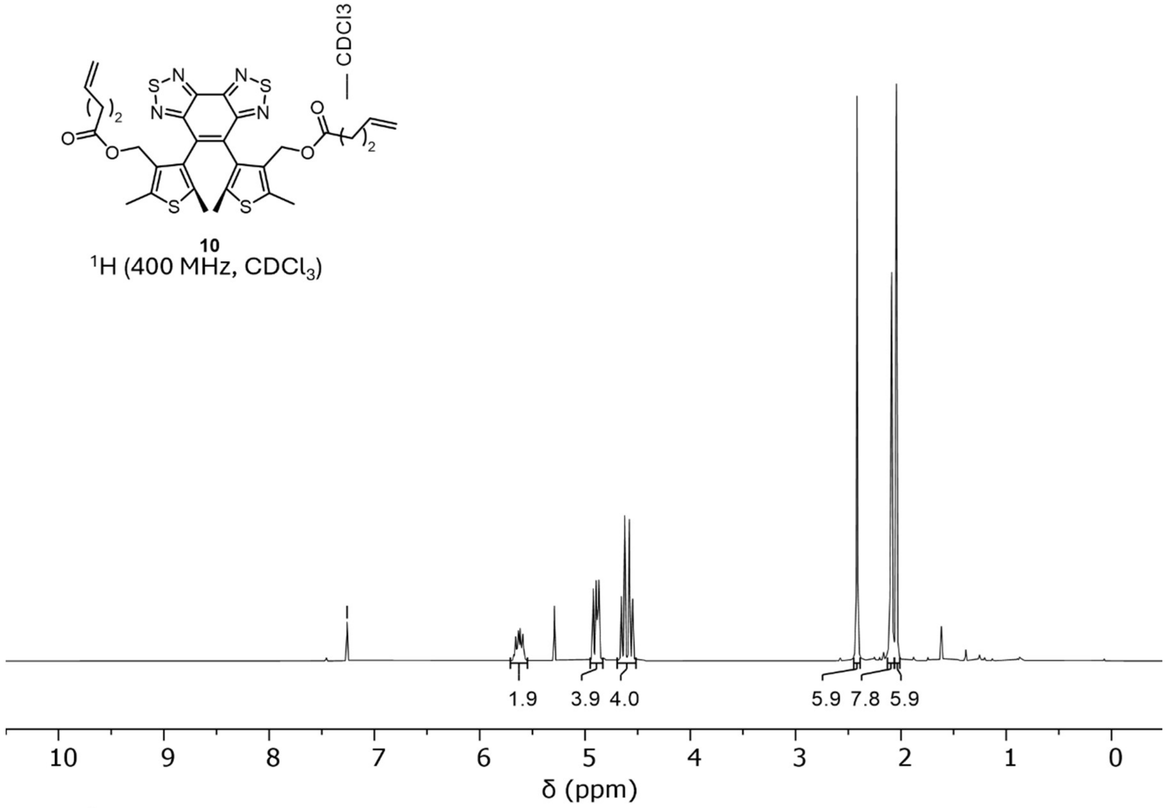
CDCl3
10
1H (400 MHz, CDCl3)
1.9 3.9 4.0
5.9 7.8 5.9
10 9 8 7 6 5 4 3 2 1 0
δ (ppm)

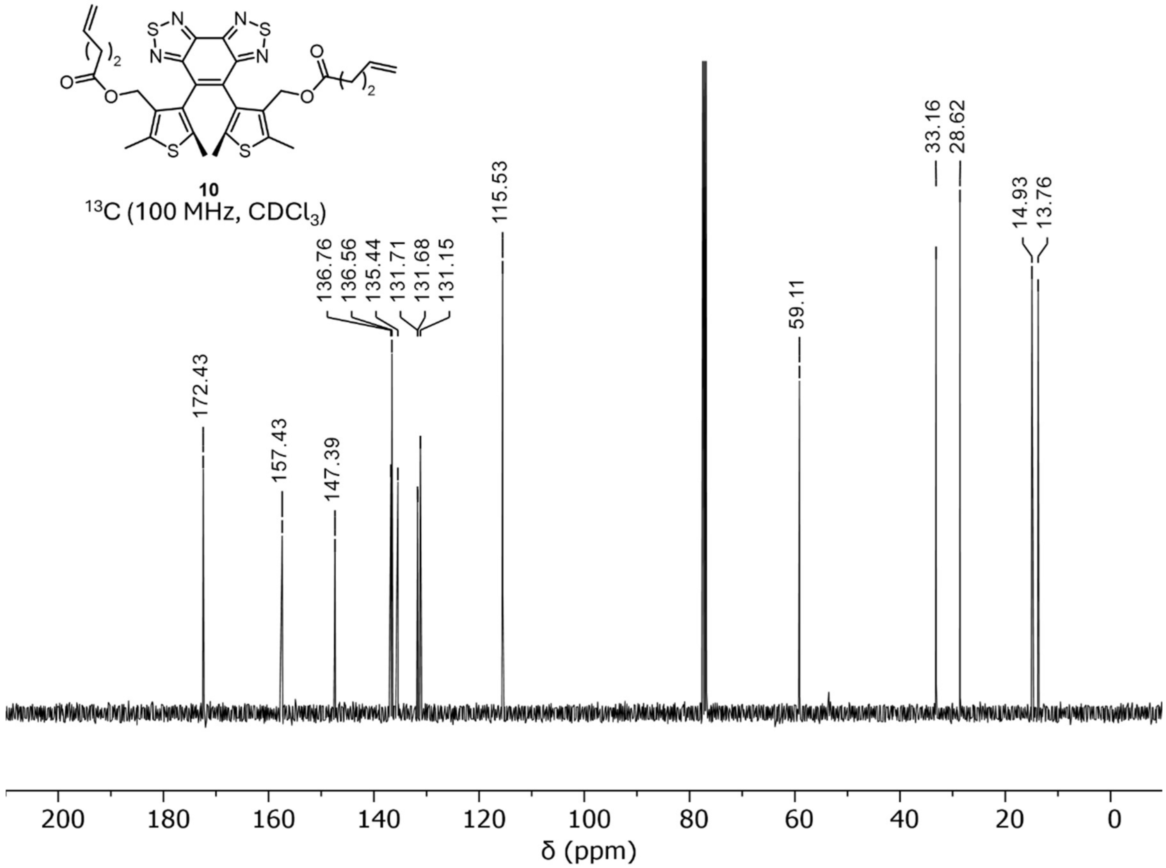
10
13C (100 MHz, CDCl3)
172.43
157.43
147.39
136.76
136.56
135.44
131.71
131.68
131.15
115.53
59.11
33.16
28.62
14.93
13.76
200 180 160 140 120 100 80 60 40 20 0
δ (ppm)

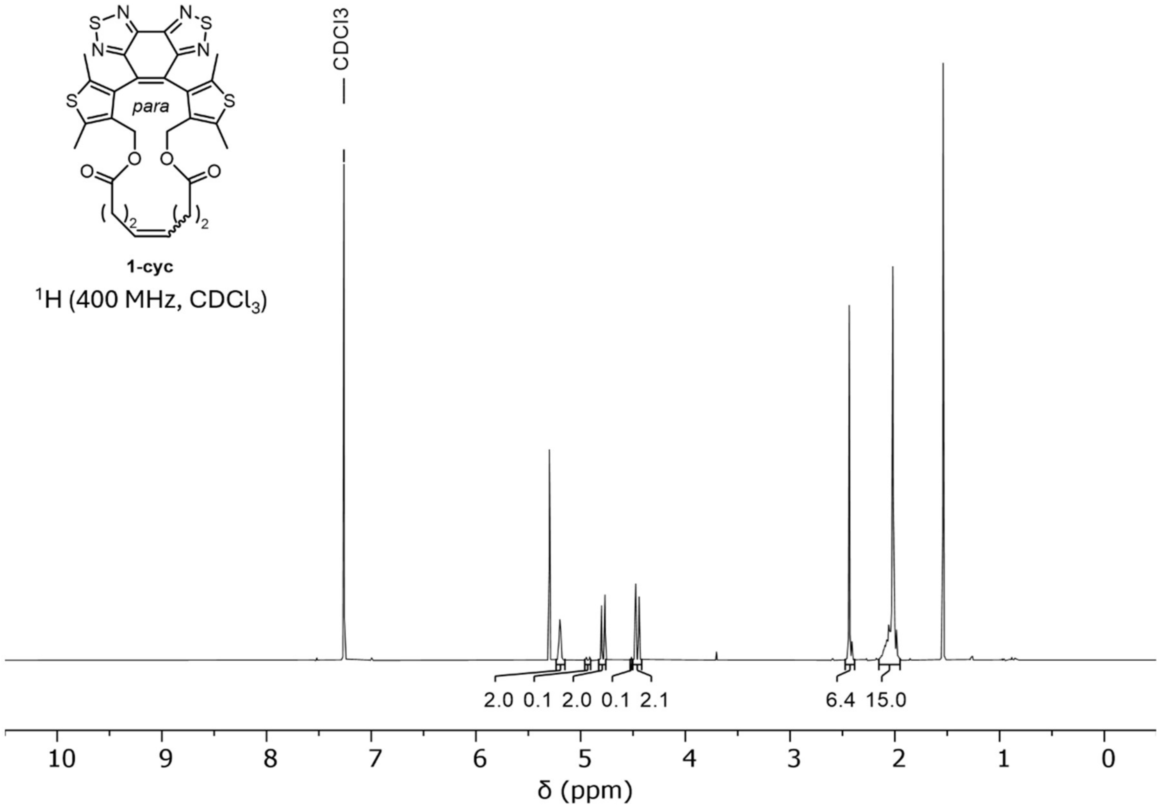
1-cyc
$^1$H (400 MHz, CDCl$_3$)
para
CDCl3
2.0 0.1 2.0 0.1 2.1
6.4 15.0
10 9 8 7 6 5 4 3 2 1 0
δ (ppm)

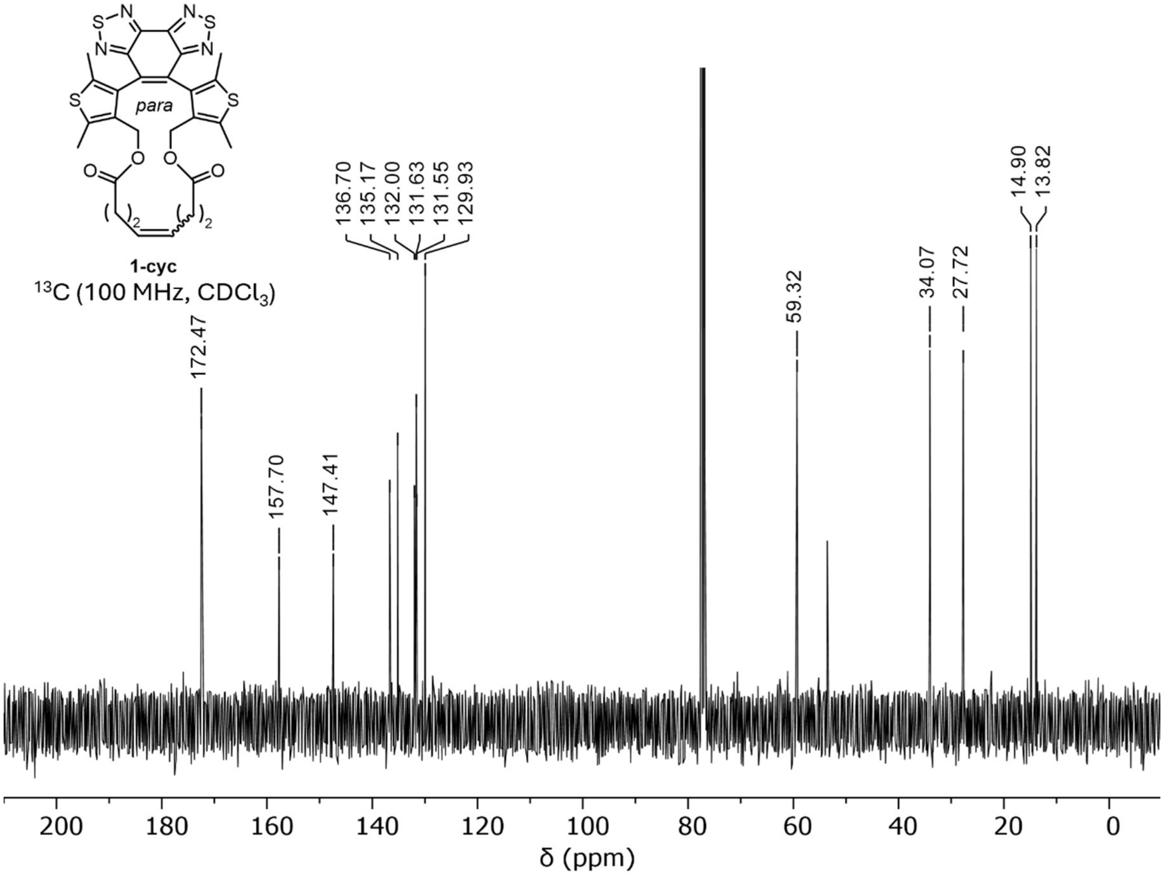
1-cyc
$^{13}$C (100 MHz, CDCl$_3$)
para
172.47
157.70
147.41
136.70
135.17
132.00
131.63
131.55
129.93
59.32
34.07
27.72
14.90
13.82
200 180 160 140 120 100 80 60 40 20 0
δ (ppm)

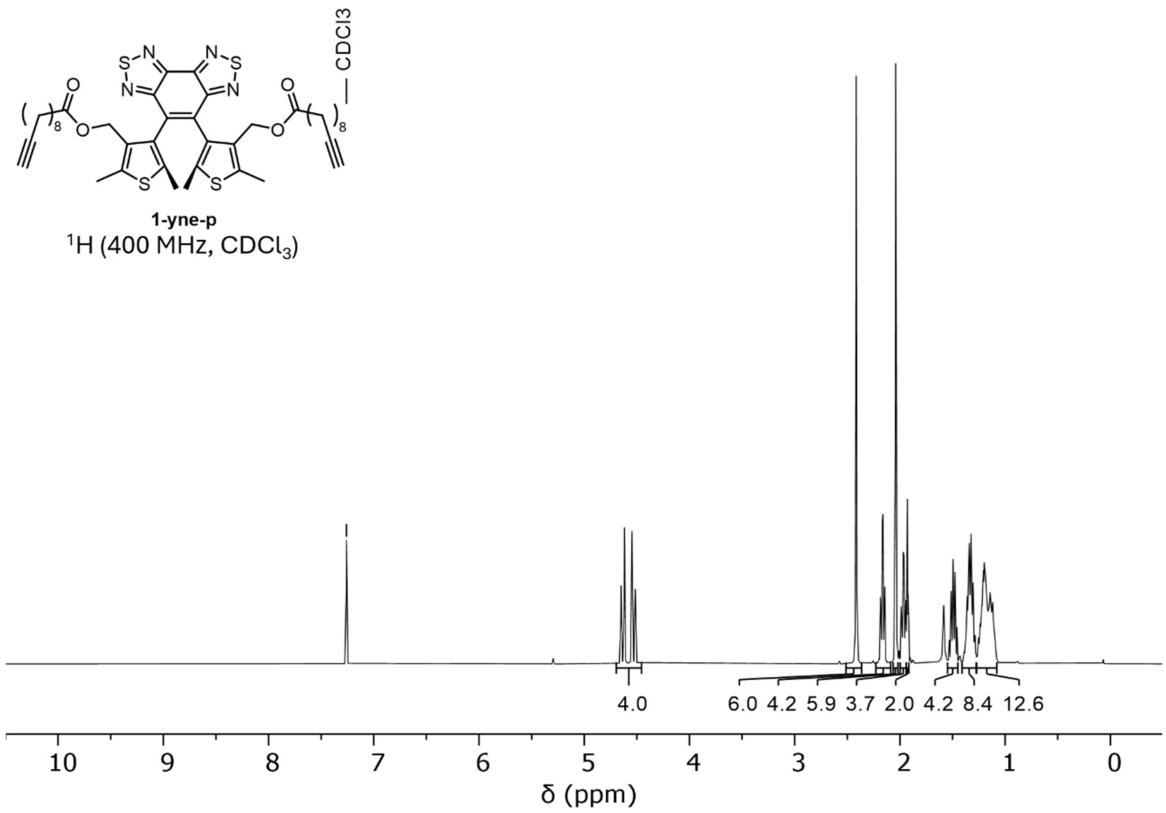
CDCl3
1-yne-p
$^1$H (400 MHz, CDCl$_3$)
4.0
6.0 4.2 5.9 3.7 2.0 4.2 8.4 12.6
10 9 8 7 6 5 4 3 2 1 0
δ (ppm)

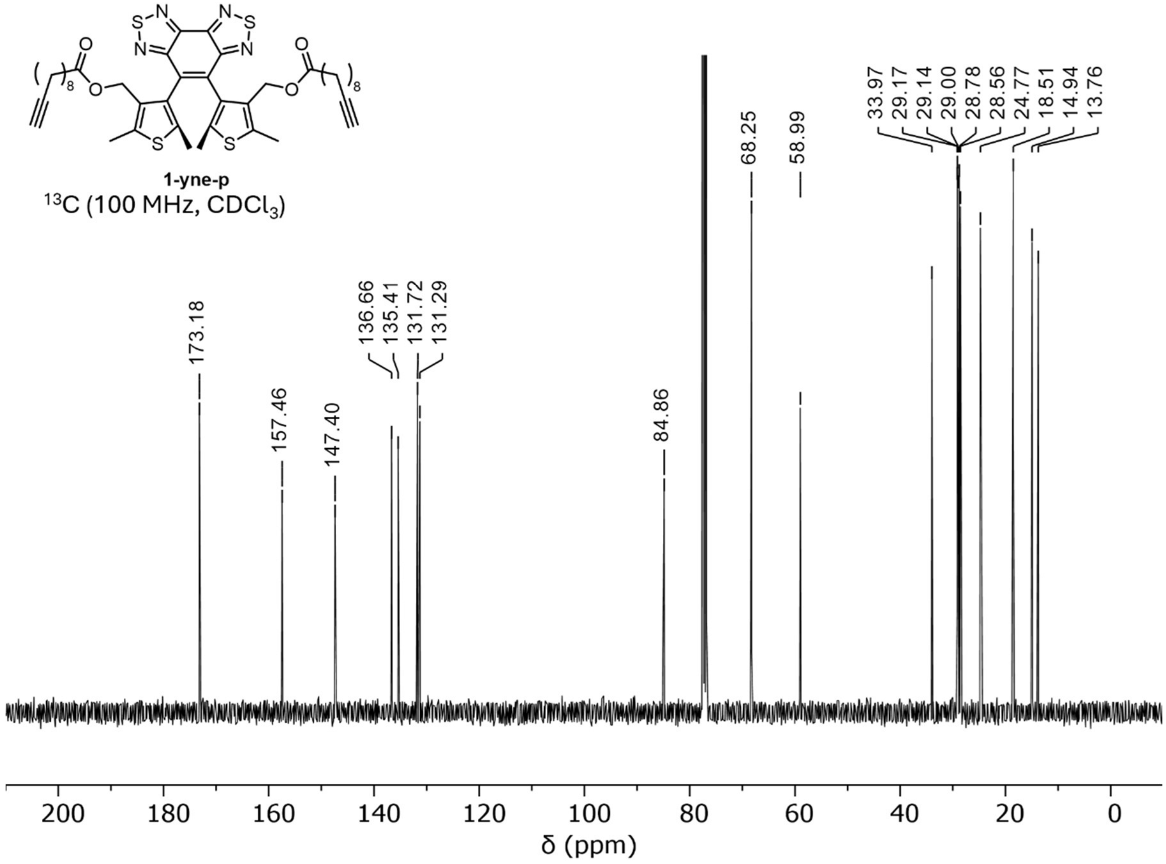
1-yne-p
$^{13}$C (100 MHz, CDCl$_3$)
173.18
157.46
147.40
136.66
135.41
131.72
131.29
84.86
68.25
58.99
33.97
29.17
29.14
29.00
28.78
28.56
24.77
18.51
14.94
13.76
200 180 160 140 120 100 80 60 40 20 0
δ (ppm)

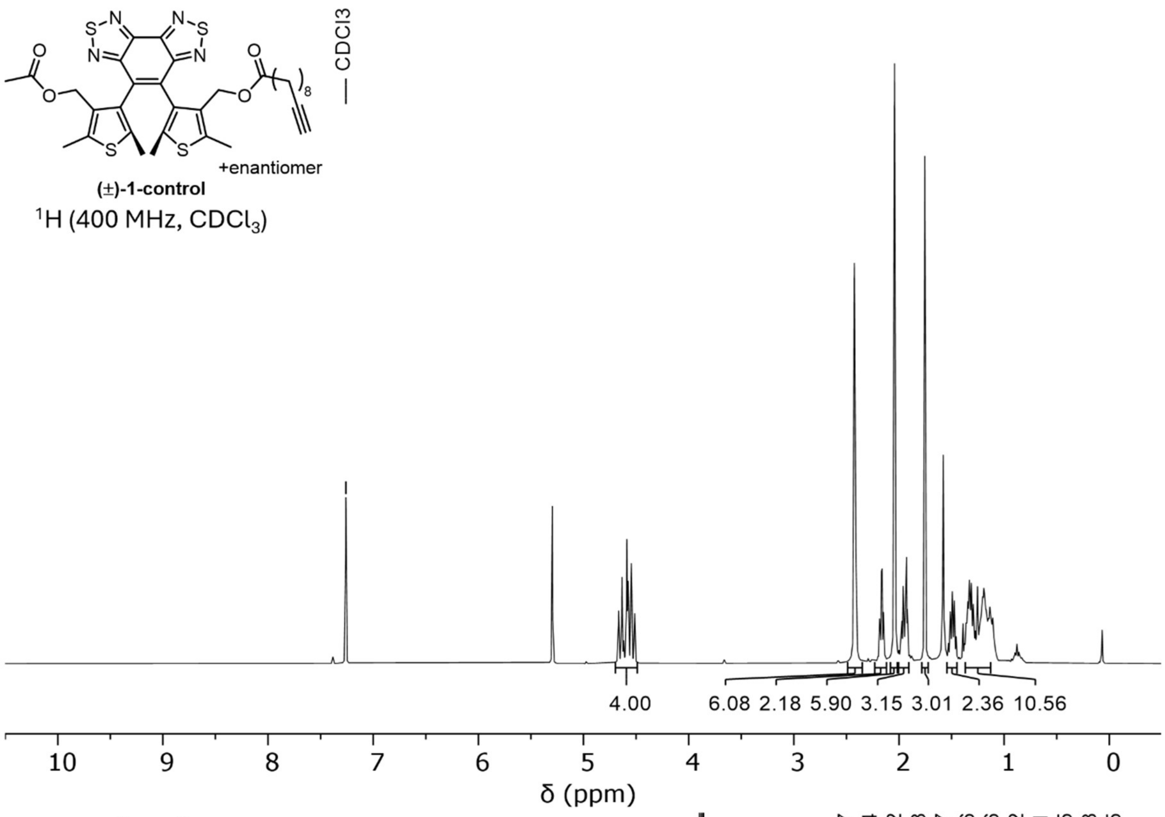

(±)-1-control
+enantiomer
¹H (400 MHz, CDCl₃)
CDCl3
4.00
6.08 2.18 5.90 3.15 3.01 2.36 10.56
δ (ppm)


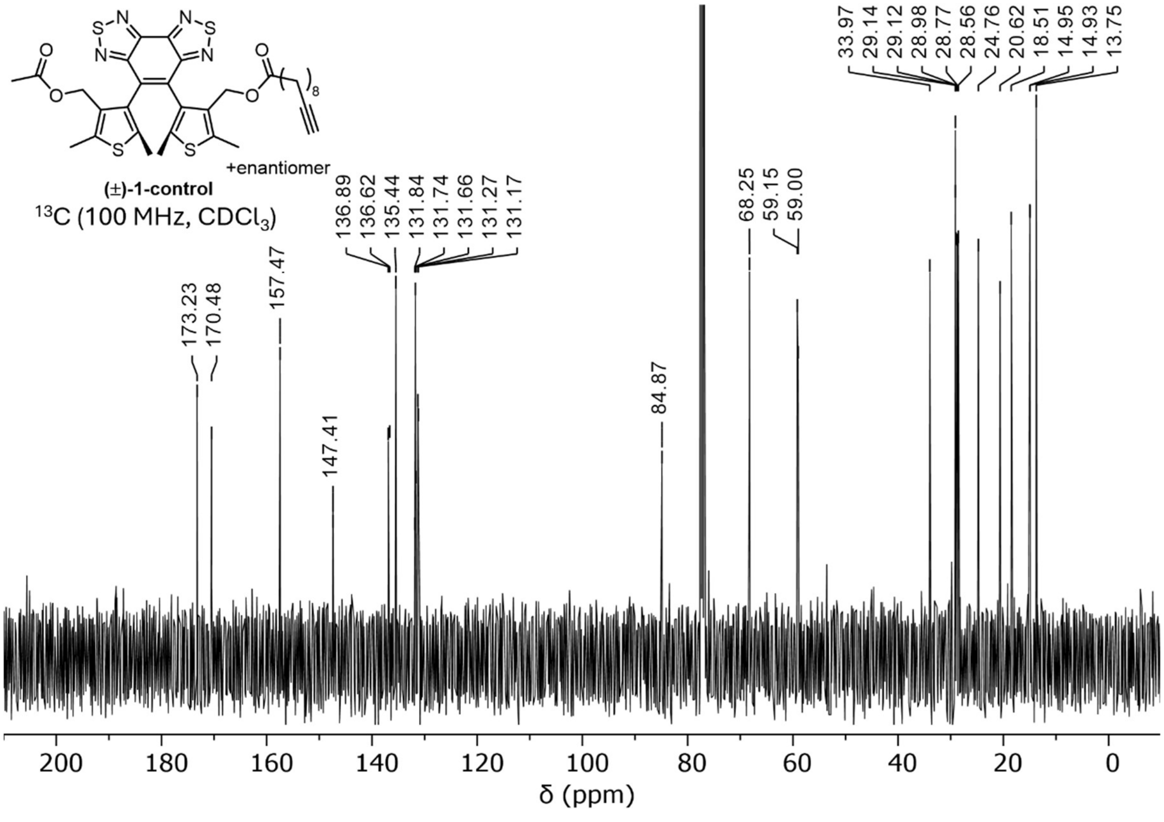

(±)-1-control
+enantiomer
¹³C (100 MHz, CDCl₃)
173.23
170.48
157.47
147.41
136.89
136.62
135.44
131.84
131.74
131.66
131.27
131.17
84.87
68.25
59.15
59.00
33.97
29.14
29.12
28.98
28.77
28.56
24.76
20.62
18.51
14.95
14.93
13.75
δ (ppm)

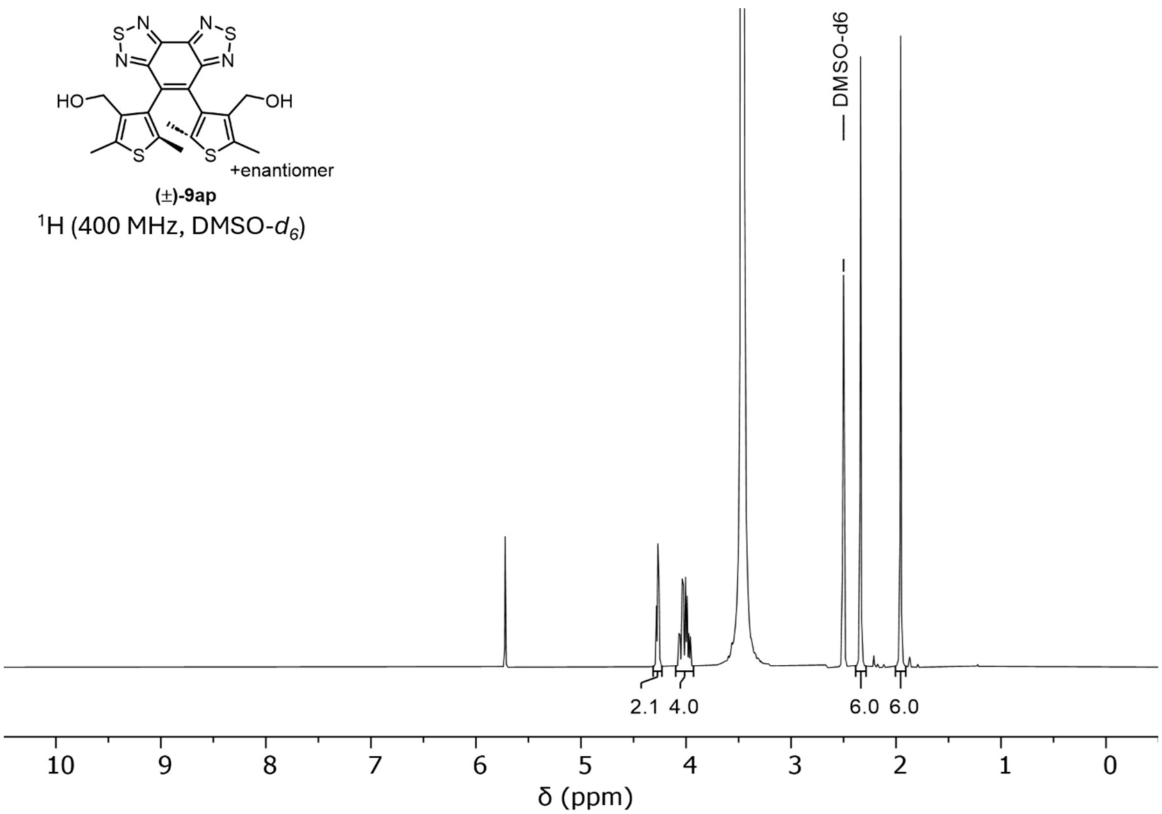
HO
OH
+enantiomer
(±)-9ap
1H (400 MHz, DMSO-d6)
DMSO-d6
2.1 4.0
6.0 6.0
10 9 8 7 6 5 4 3 2 1 0
δ (ppm)

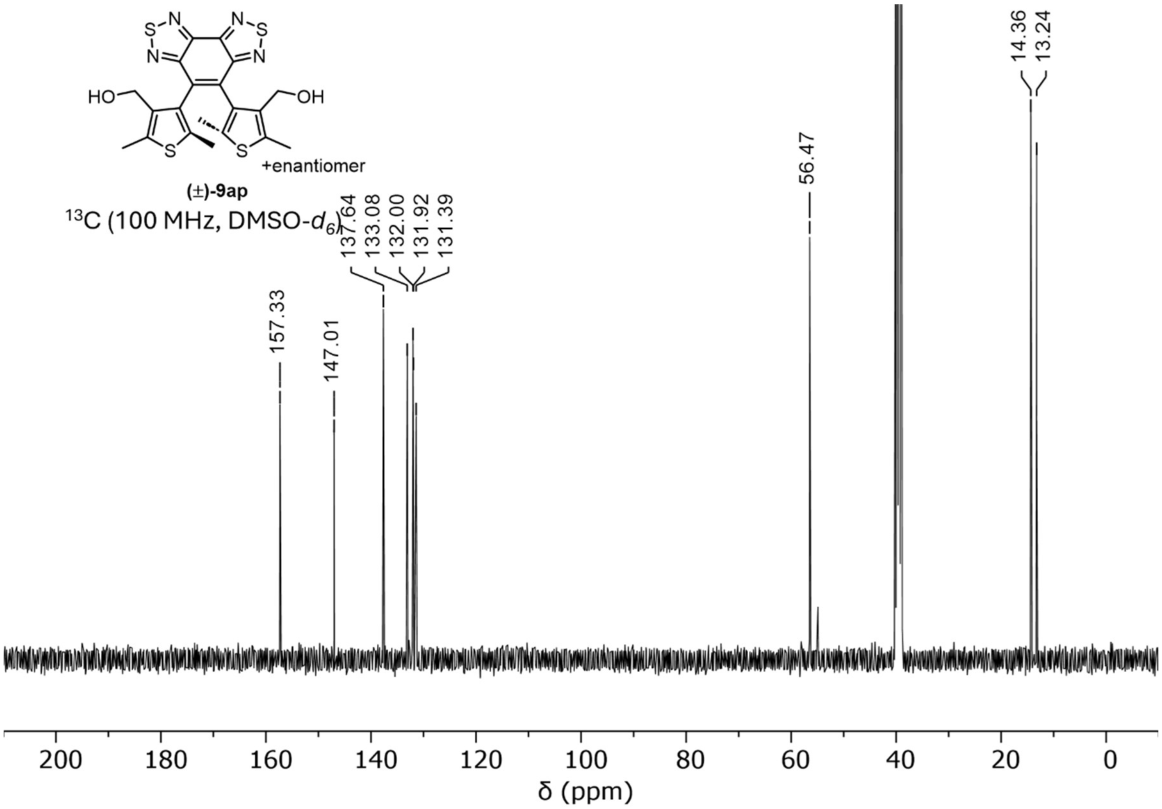
HO
OH
+enantiomer
(±)-9ap
13C (100 MHz, DMSO-d6)
157.33
147.01
137.64
133.08
132.00
131.92
131.39
56.47
14.36
13.24
200 180 160 140 120 100 80 60 40 20 0
δ (ppm)

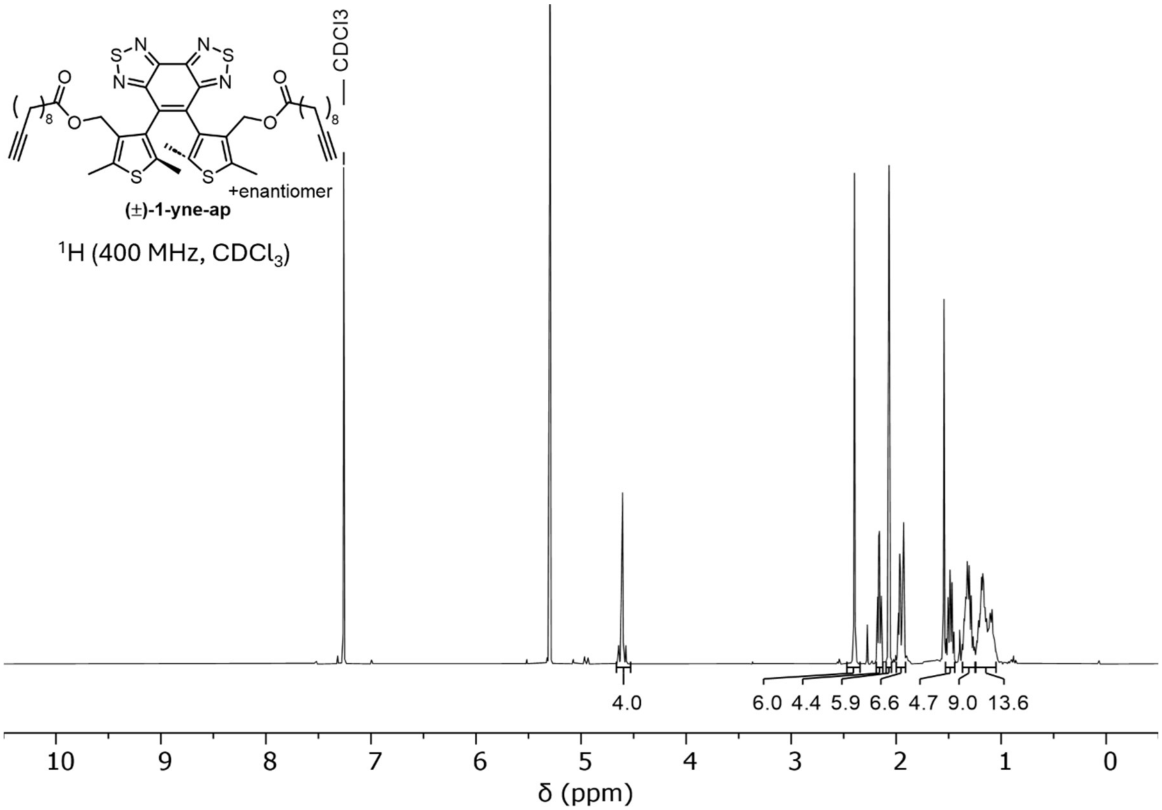
CDCl3
(±)-1-yne-ap
+enantiomer
$^{1}$H (400 MHz, CDCl3)
4.0
6.0 4.4 5.9 6.6 4.7 9.0 13.6
δ (ppm)

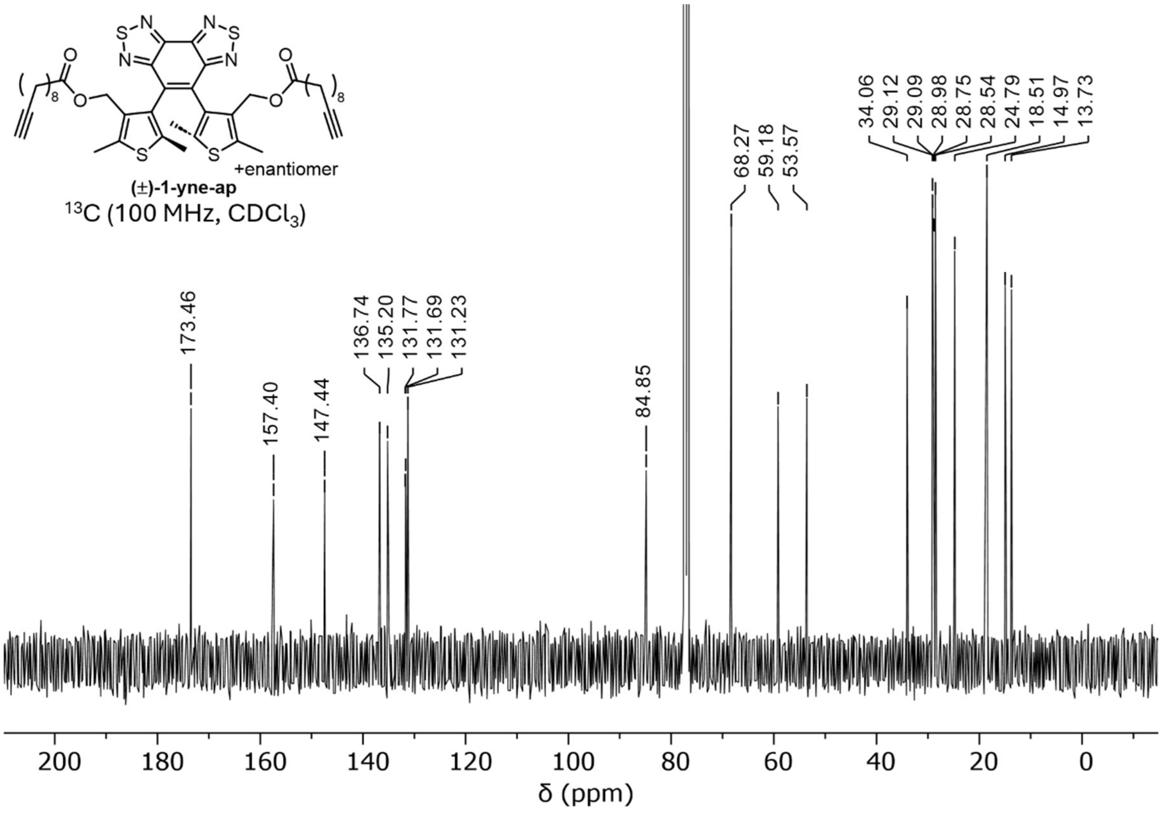
(±)-1-yne-ap
+enantiomer
$^{13}$C (100 MHz, CDCl3)
173.46
157.40
147.44
136.74
135.20
131.77
131.69
131.23
84.85
68.27
59.18
53.57
34.06
29.12
29.09
28.98
28.75
28.54
24.79
18.51
14.97
13.73
δ (ppm)

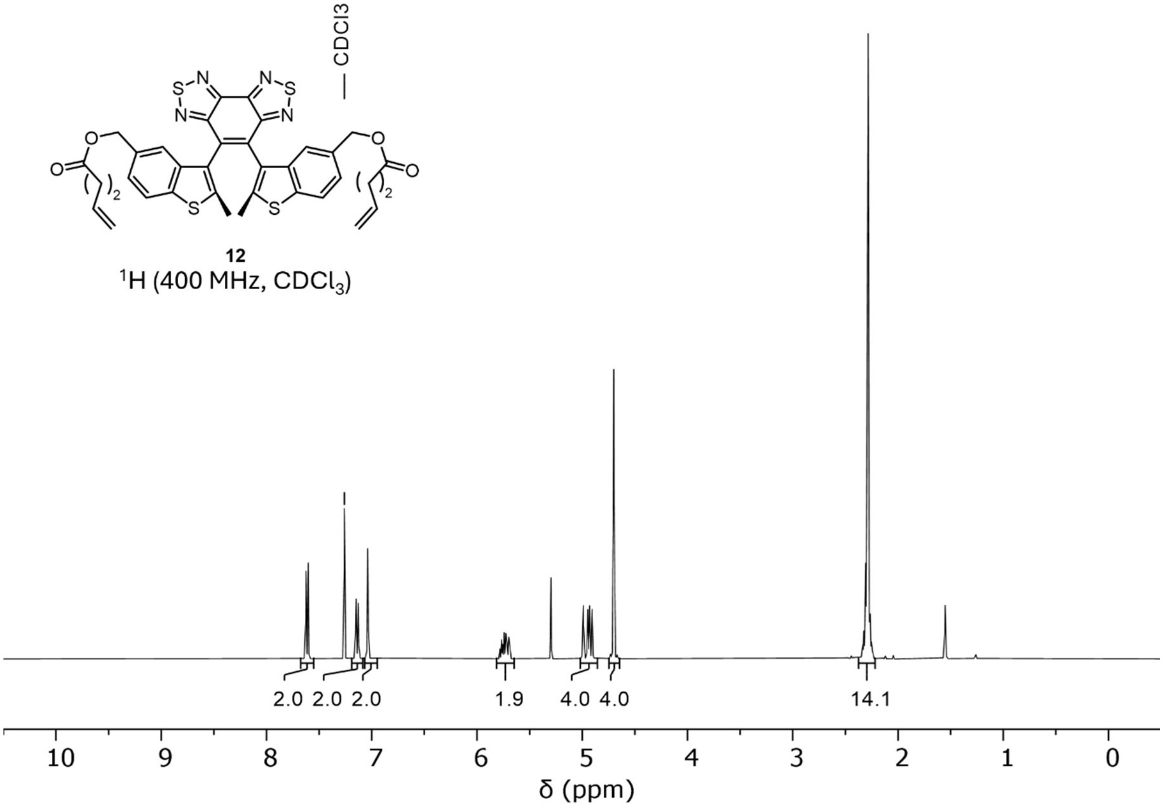
CDCl3
12
$^{1}$H (400 MHz, $CDCl_3$)
2.0 2.0 2.0
1.9
4.0 4.0
14.1
10 9 8 7 6 5 4 3 2 1 0
δ (ppm)

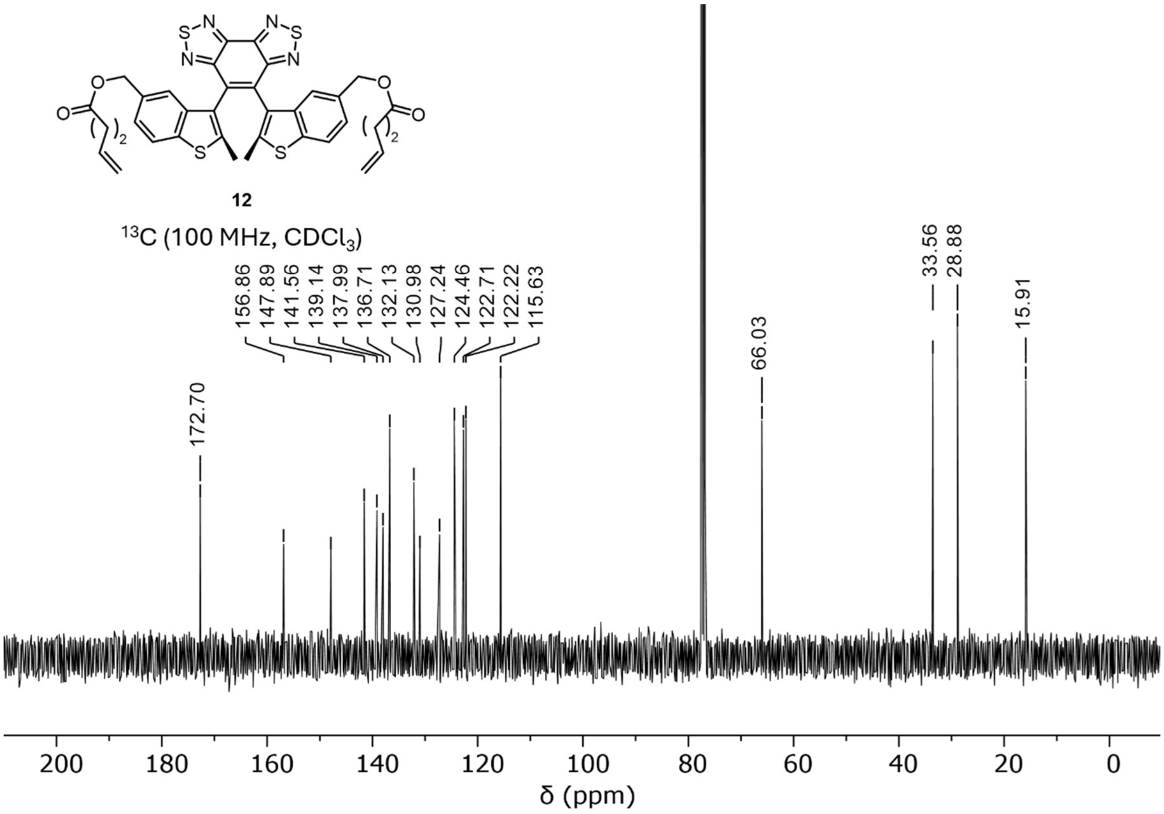
12
$^{13}$C (100 MHz, $CDCl_3$)
172.70
156.86
147.89
141.56
139.14
137.99
136.71
132.13
130.98
127.24
124.46
122.71
122.22
115.63
66.03
33.56
28.88
15.91
200 180 160 140 120 100 80 60 40 20 0
δ (ppm)

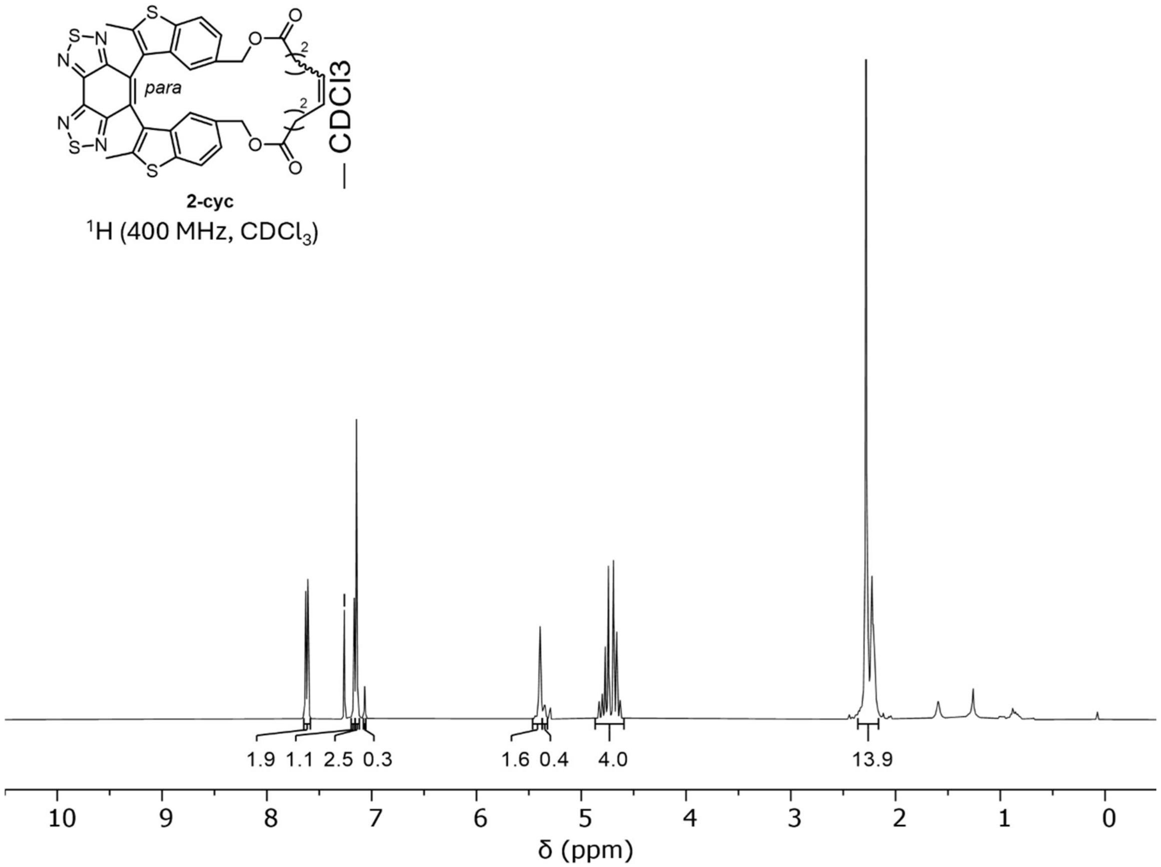

2-cyc
$^{1}$H (400 MHz, CDCl$_3$)
para
CDCl3
1.9 1.1 2.5 0.3
1.6 0.4
4.0
13.9
δ (ppm)


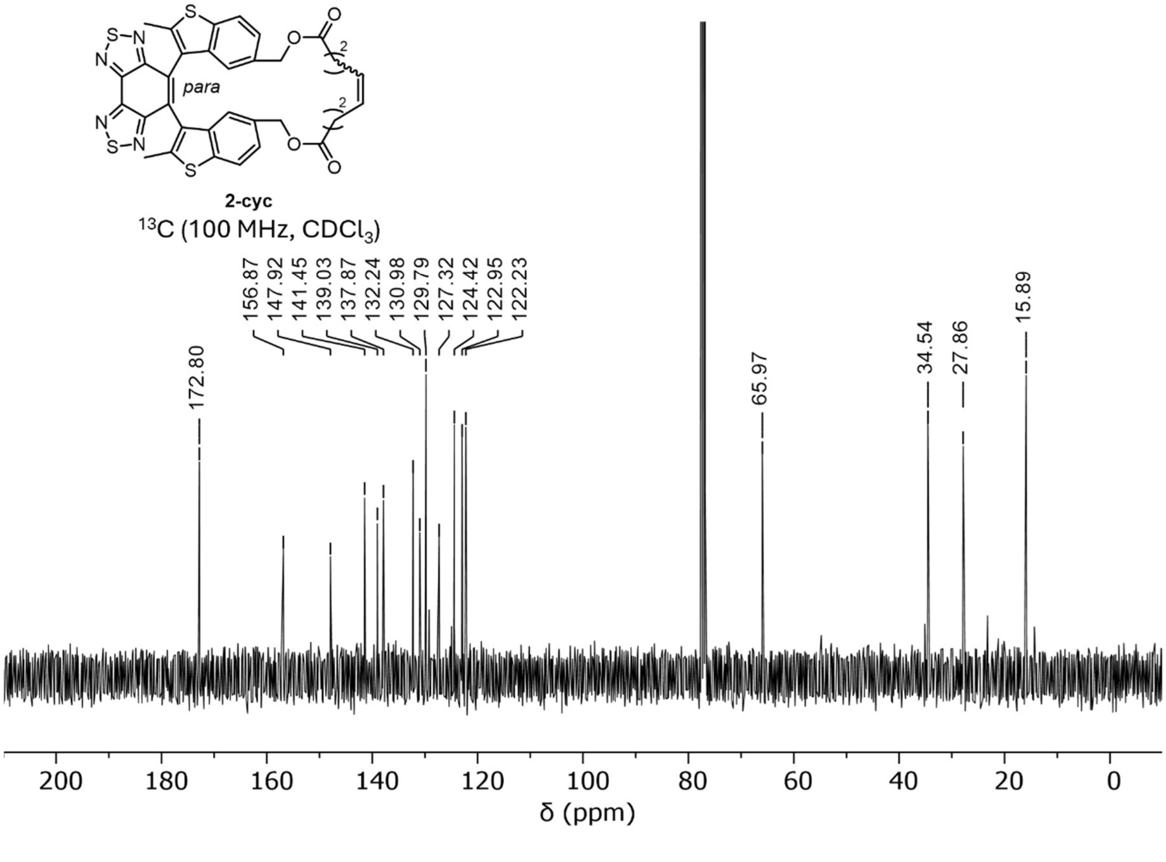

2-cyc
$^{13}$C (100 MHz, CDCl$_3$)
para
172.80
156.87
147.92
141.45
139.03
137.87
132.24
130.98
129.79
127.32
124.42
122.95
122.23
65.97
34.54
27.86
15.89
δ (ppm)

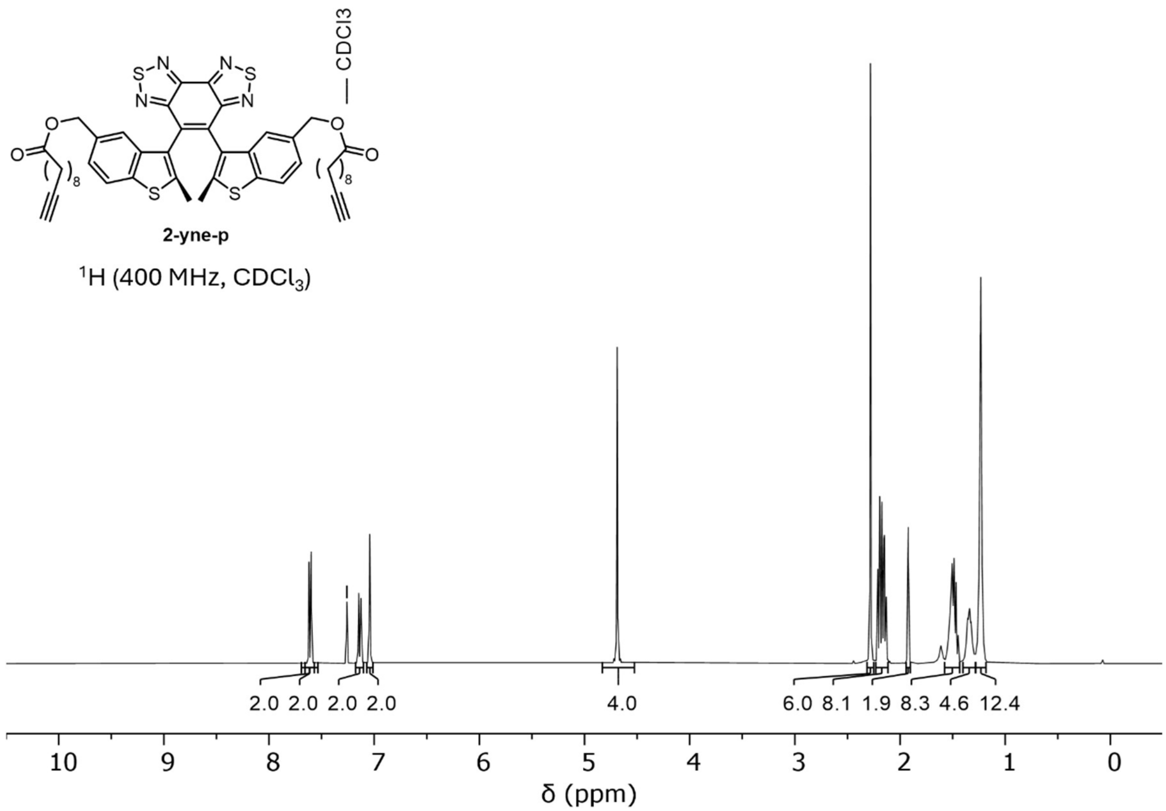
CDCl3
2-yne-p
1H (400 MHz, CDCl3)
2.0 2.0 2.0 2.0
4.0
6.0 8.1 1.9 8.3 4.6 12.4
10 9 8 7 6 5 4 3 2 1 0
δ (ppm)

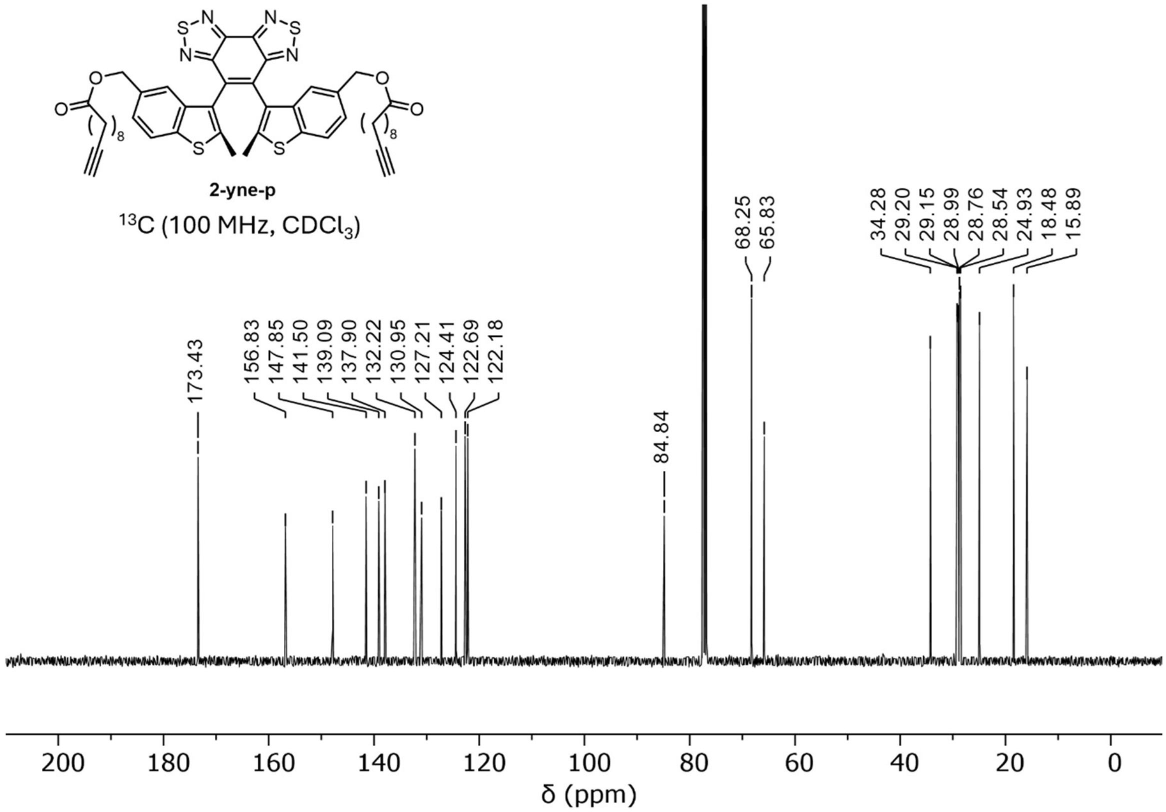
2-yne-p
13C (100 MHz, CDCl3)
173.43
156.83 147.85 141.50 139.09 137.90 132.22 130.95 127.21 124.41 122.69 122.18
84.84
68.25 65.83
34.28 29.20 29.15 28.99 28.76 28.54 24.93 18.48 15.89
200 180 160 140 120 100 80 60 40 20 0
δ (ppm)

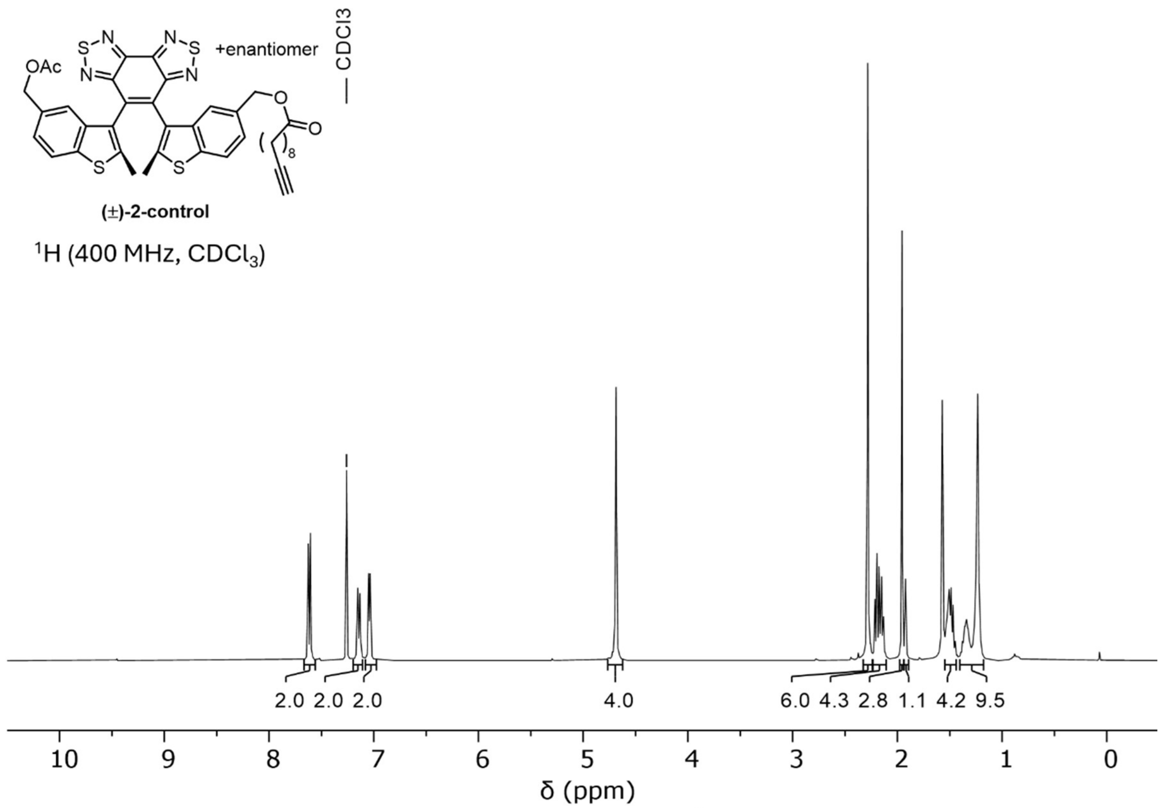

OAc
+enantiomer
CDCl3
(±)-2-control
$^1$H (400 MHz, CDCl$_3$)
2.0 2.0 2.0
4.0
6.0 4.3 2.8 1.1 4.2 9.5
10 9 8 7 6 5 4 3 2 1 0
δ (ppm)

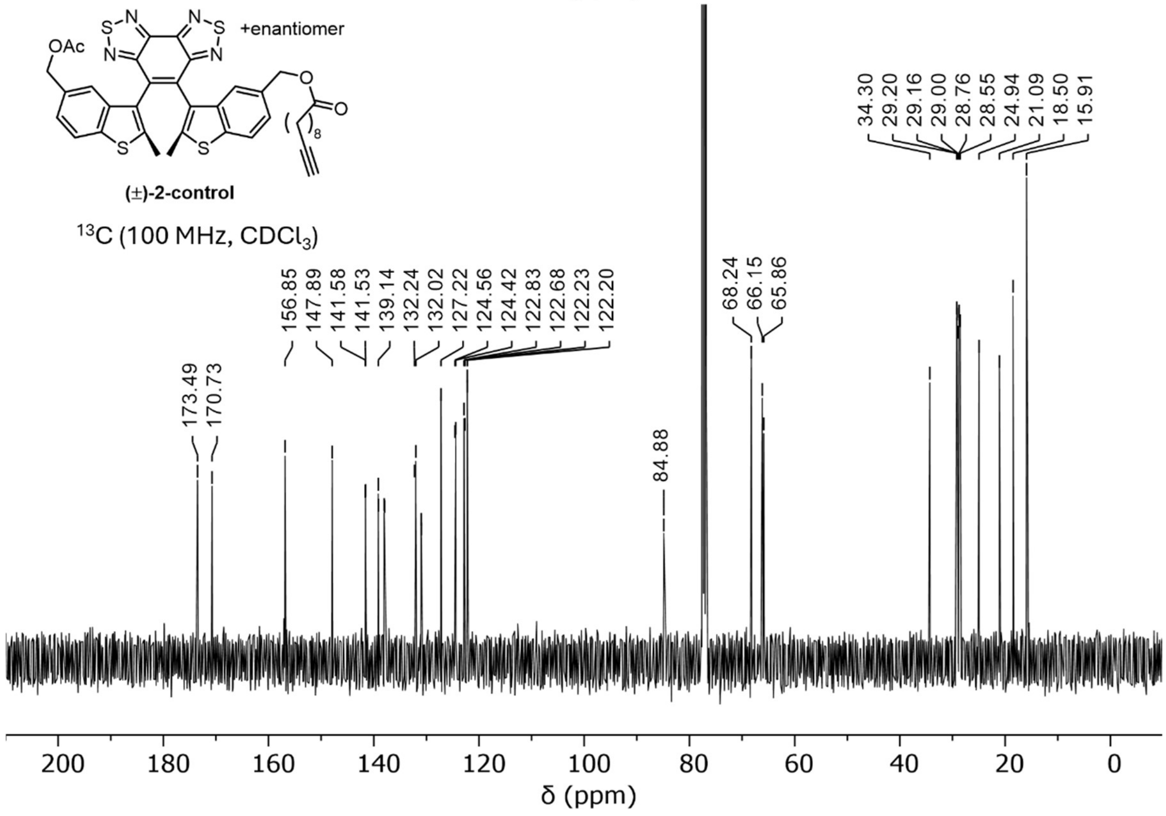

OAc
+enantiomer
(±)-2-control
$^{13}$C (100 MHz, CDCl$_3$)
173.49
170.73
156.85
147.89
141.58
141.53
139.14
132.24
132.02
127.22
124.56
124.42
122.83
122.68
122.23
122.20
84.88
68.24
66.15
65.86
34.30
29.20
29.16
29.00
28.76
28.55
24.94
21.09
18.50
15.91
200 180 160 140 120 100 80 60 40 20 0
δ (ppm)

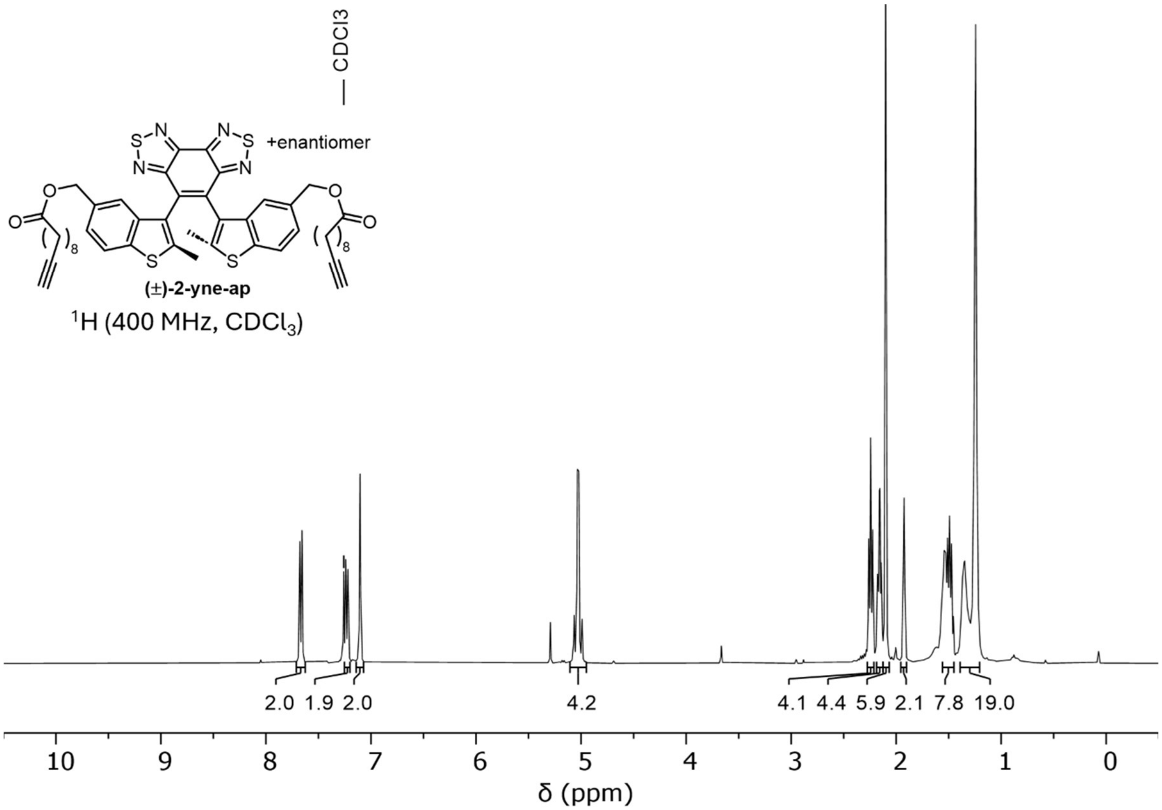
CDCl3
+enantiomer
(±)-2-yne-ap
1H (400 MHz, CDCl3)
2.0 1.9 2.0
4.2
4.1 4.4 5.9 2.1 7.8 19.0
δ (ppm)

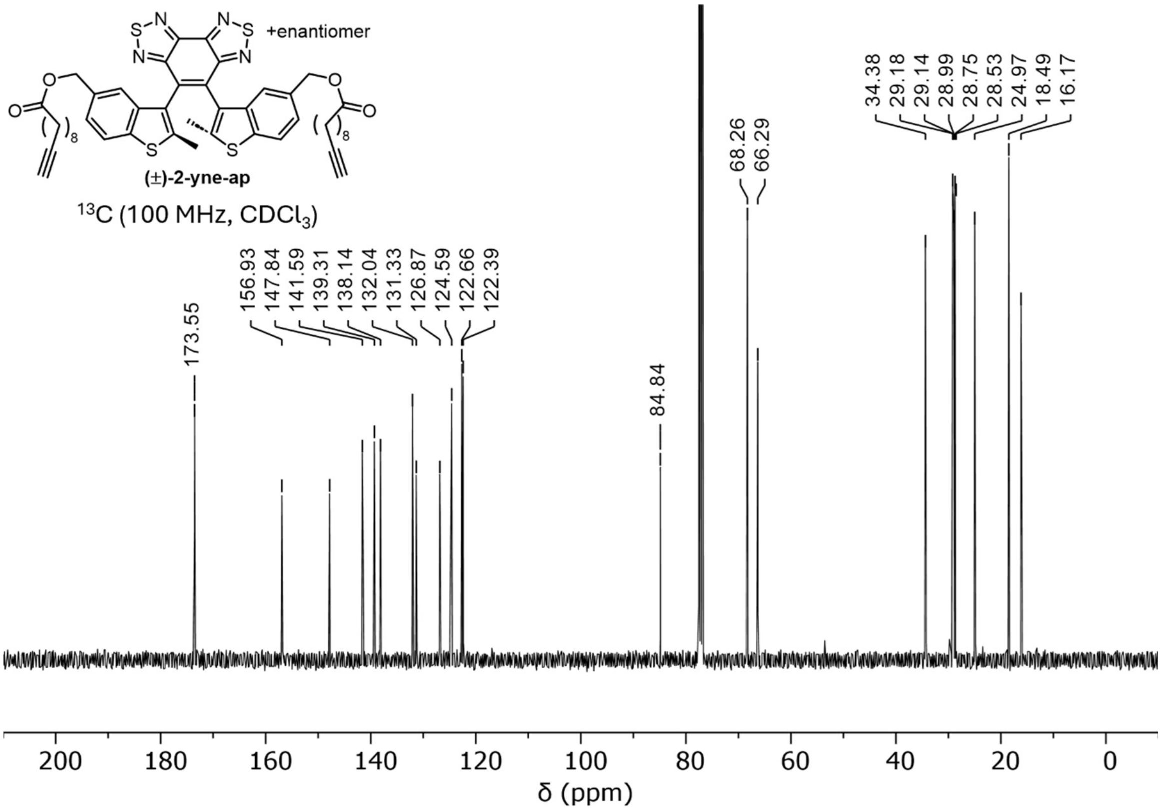
+enantiomer
(±)-2-yne-ap
13C (100 MHz, CDCl3)
173.55
156.93 147.84 141.59 139.31 138.14 132.04 131.33 126.87 124.59 122.66 122.39
84.84
68.26 66.29
34.38 29.18 29.14 28.99 28.75 28.53 24.97 18.49 16.17
δ (ppm)

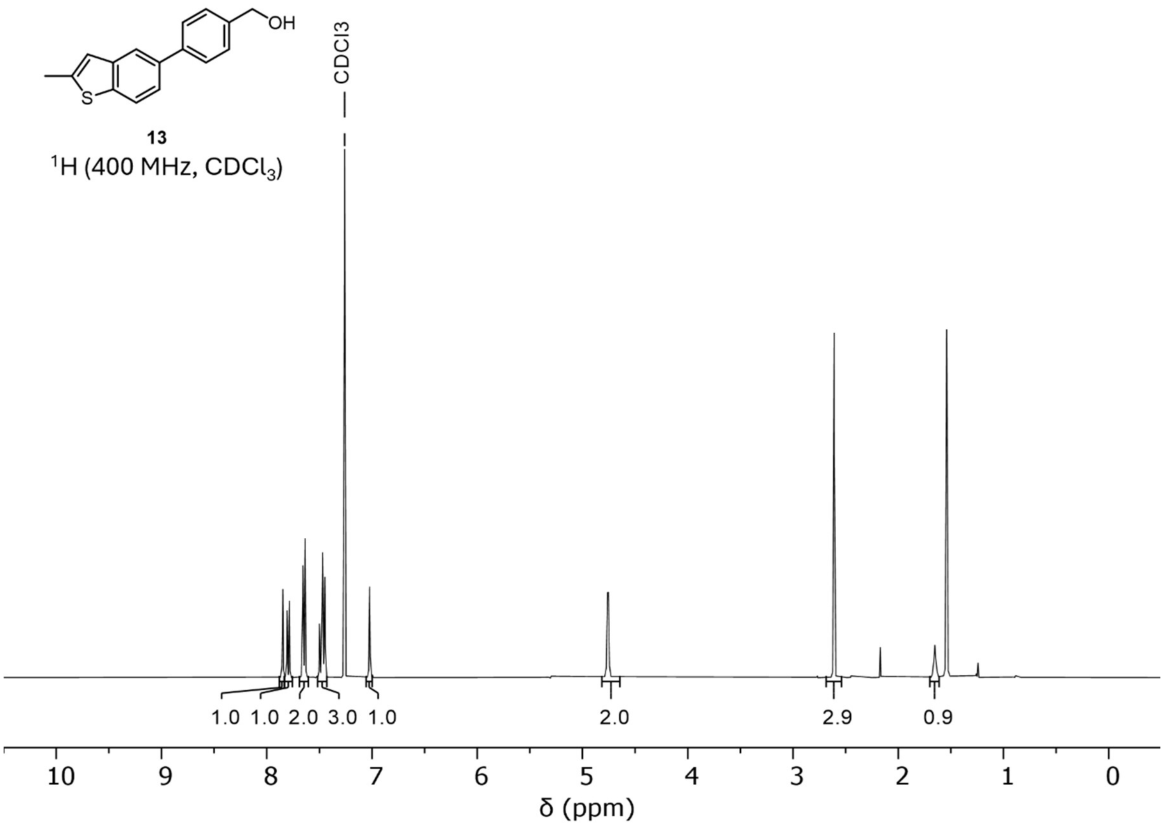
OH
S
13
$^1$H (400 MHz, CDCl$_3$)
CDCl3
1.0 1.0 2.0 3.0 1.0
2.0
2.9
0.9
10 9 8 7 6 5 4 3 2 1 0
δ (ppm)

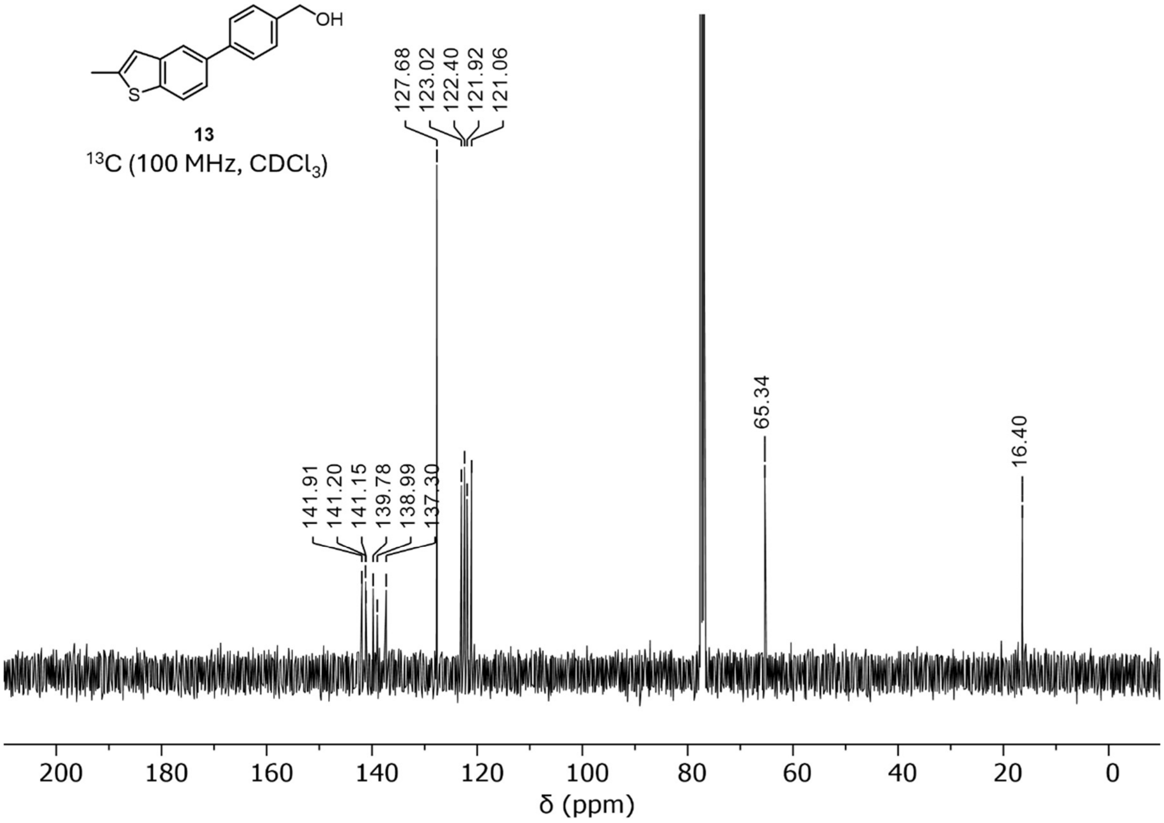
OH
S
13
$^{13}$C (100 MHz, CDCl$_3$)
127.68
123.02
122.40
121.92
121.06
141.91
141.20
141.15
139.78
138.99
137.30
65.34
16.40
200 180 160 140 120 100 80 60 40 20 0
δ (ppm)

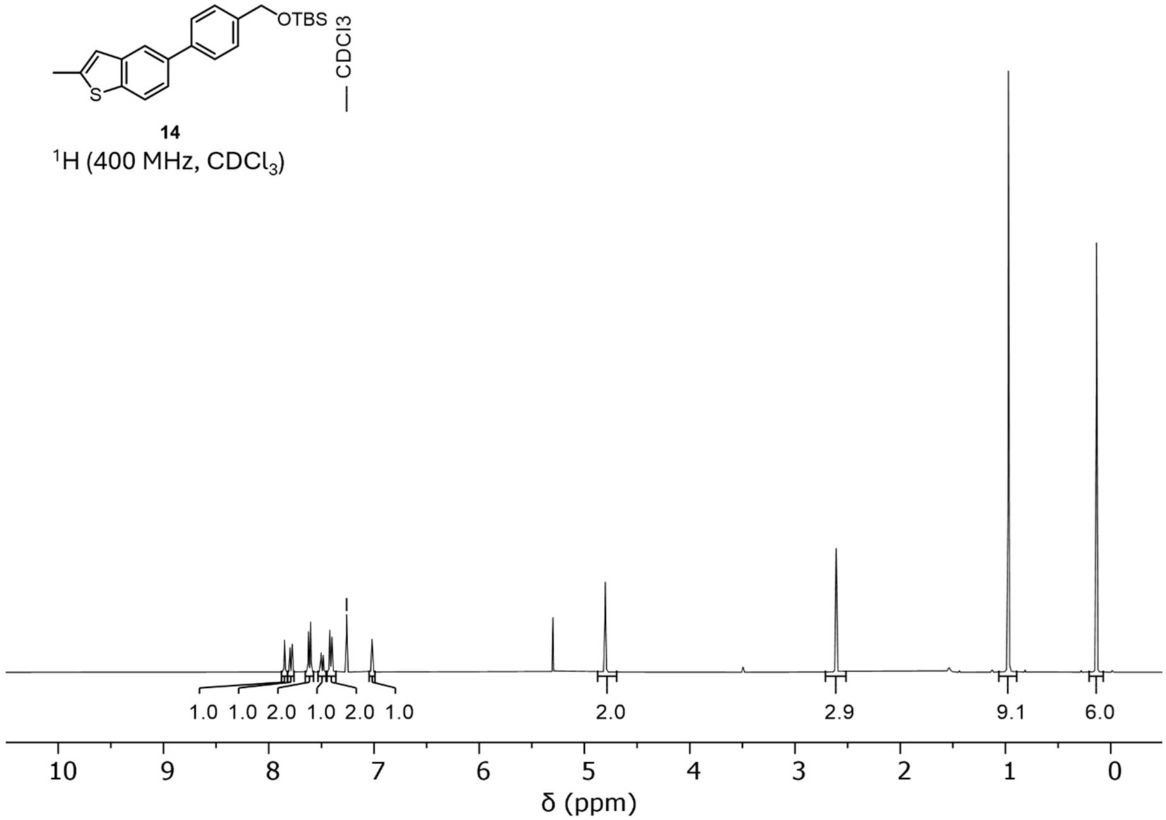
OTBS
S
CDCl3
14
1H (400 MHz, CDCl3)
1.0 1.0 2.0 1.0 2.0 1.0
2.0
2.9
9.1
6.0
10 9 8 7 6 5 4 3 2 1 0
δ (ppm)

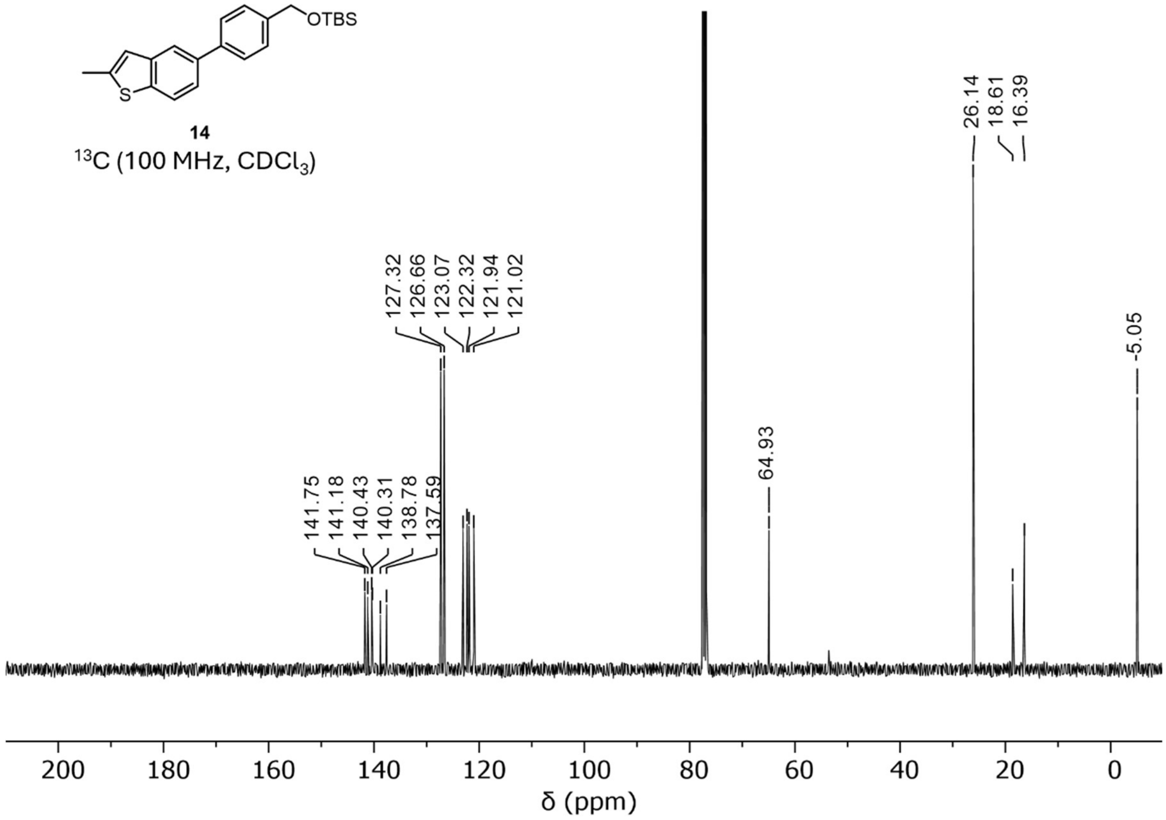
OTBS
S
14
13C (100 MHz, CDCl3)
26.14
18.61
16.39
127.32
126.66
123.07
122.32
121.94
121.02
-5.05
64.93
141.75
141.18
140.43
140.31
138.78
137.59
200 180 160 140 120 100 80 60 40 20 0
δ (ppm)

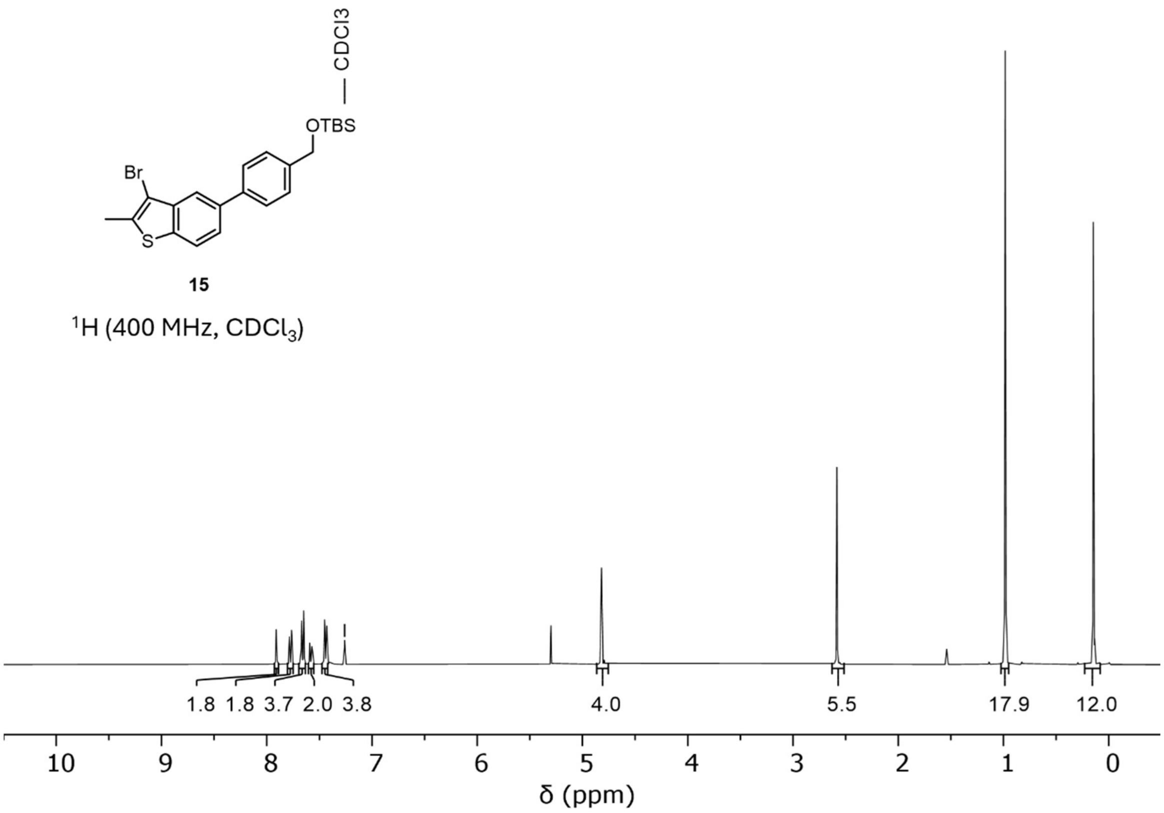

CDCl3
OTBS
Br
S
15
1H (400 MHz, CDCl3)
1.8 1.8 3.7 2.0 3.8
4.0
5.5
17.9
12.0
10 9 8 7 6 5 4 3 2 1 0
δ (ppm)


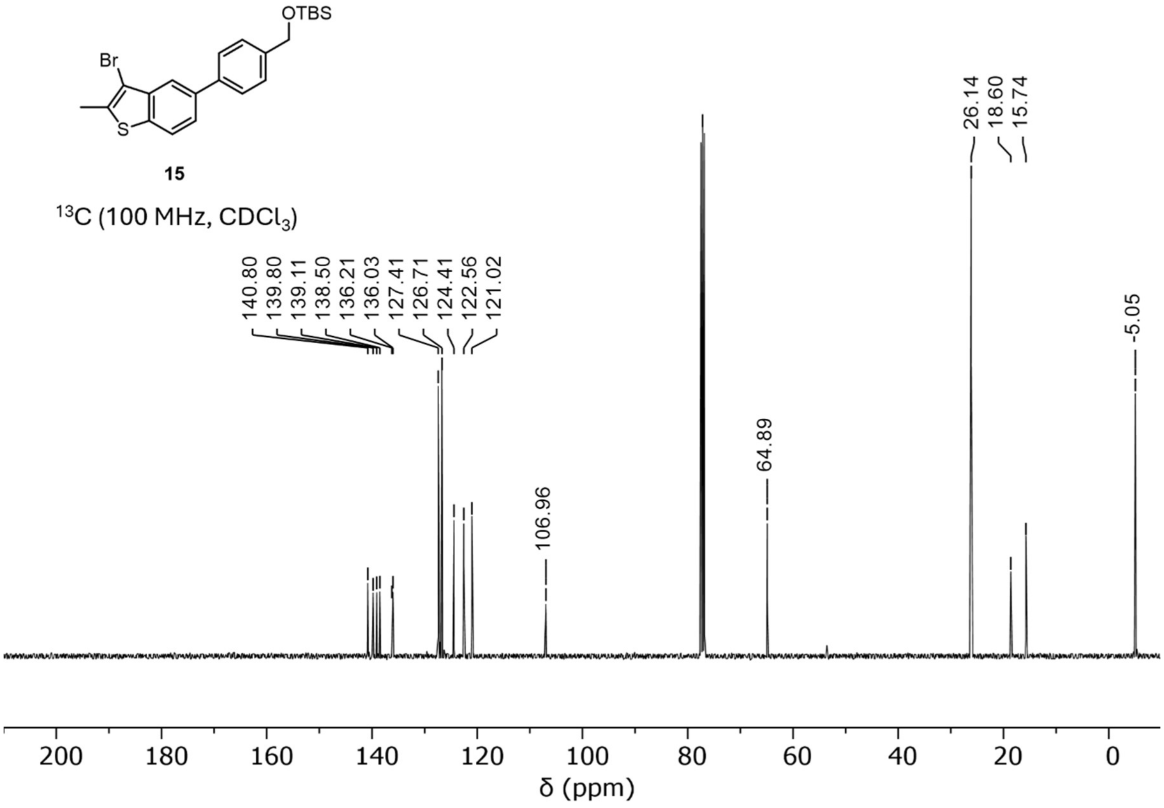

OTBS
Br
S
15
13C (100 MHz, CDCl3)
140.80 139.80 139.11 138.50 136.21 136.03 127.41 126.71 124.41 122.56 121.02
106.96
64.89
26.14 18.60 15.74
-5.05
200 180 160 140 120 100 80 60 40 20 0
δ (ppm)

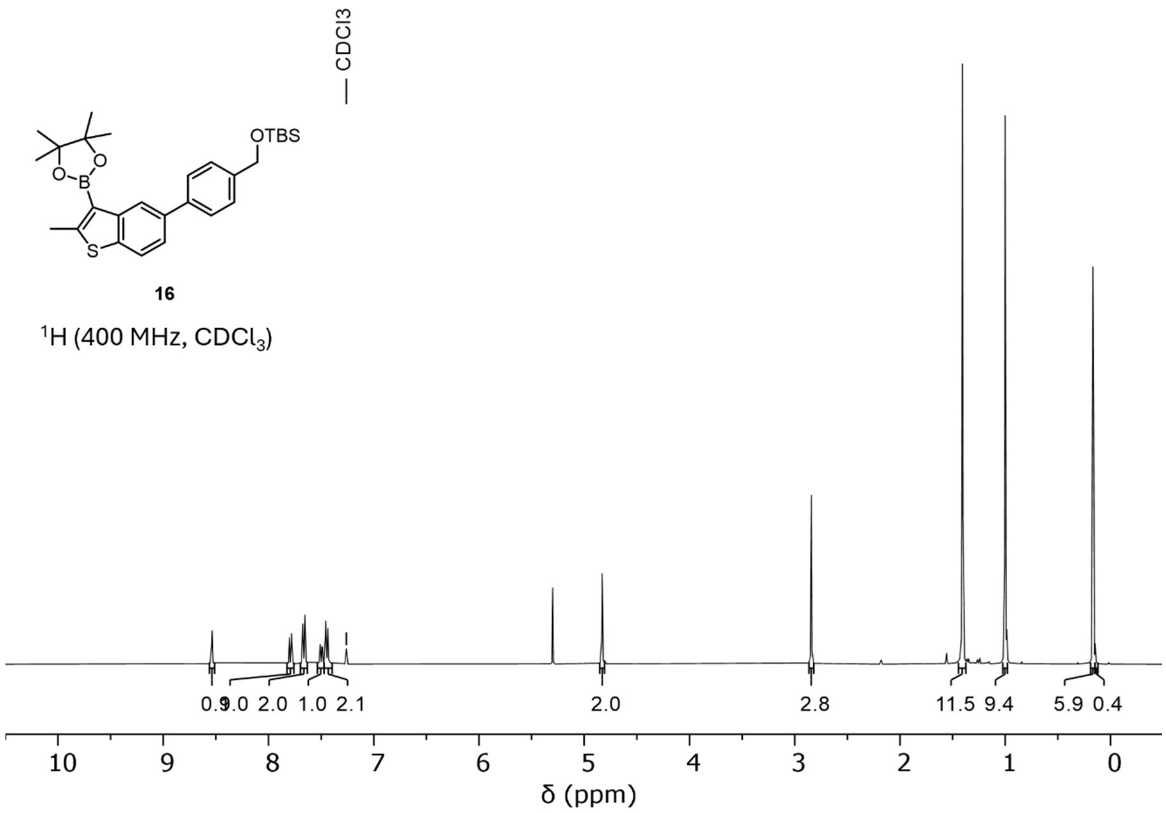
OTBS
16
$^1$H (400 MHz, CDCl$_3$)
CDCl3
0.9 1.0 2.0 1.0 2.1
2.0
2.8
11.5 9.4
5.9 0.4
10 9 8 7 6 5 4 3 2 1 0
δ (ppm)

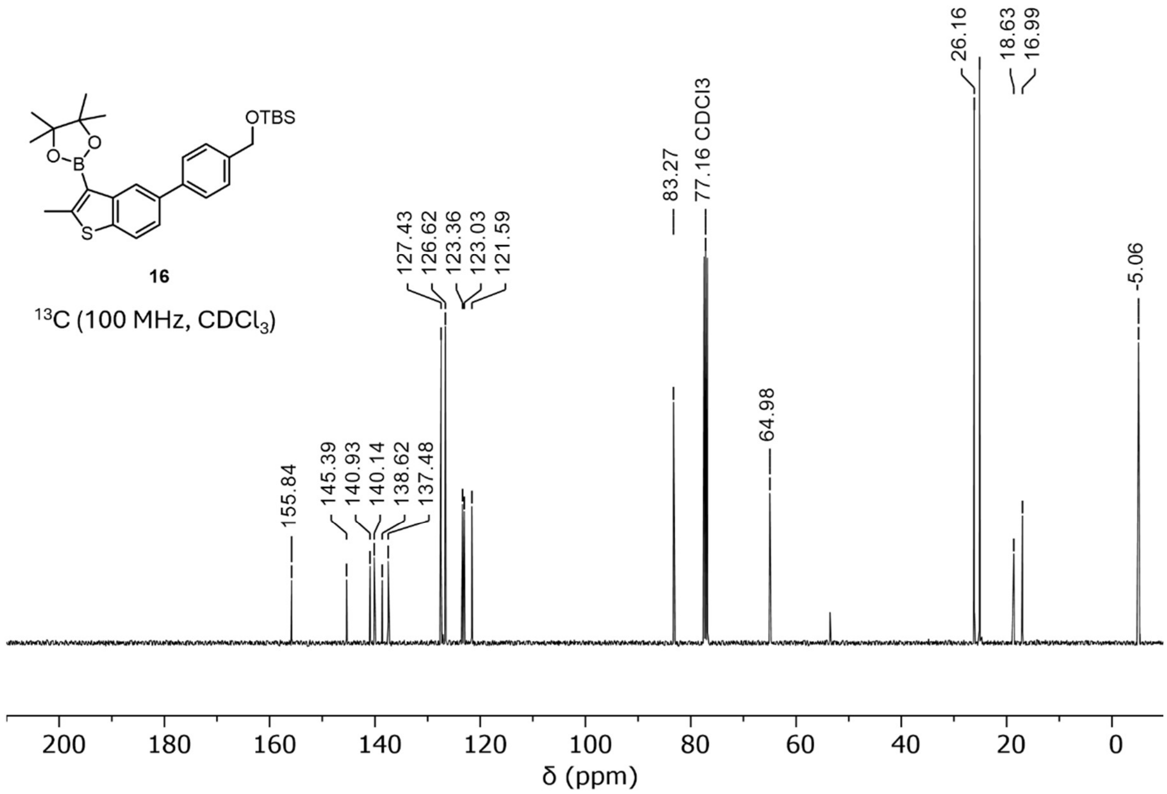
OTBS
16
$^{13}$C (100 MHz, CDCl$_3$)
155.84
145.39
140.93
140.14
138.62
137.48
127.43
126.62
123.36
123.03
121.59
83.27
77.16 CDCl3
64.98
26.16
18.63
16.99
-5.06
200 180 160 140 120 100 80 60 40 20 0
δ (ppm)

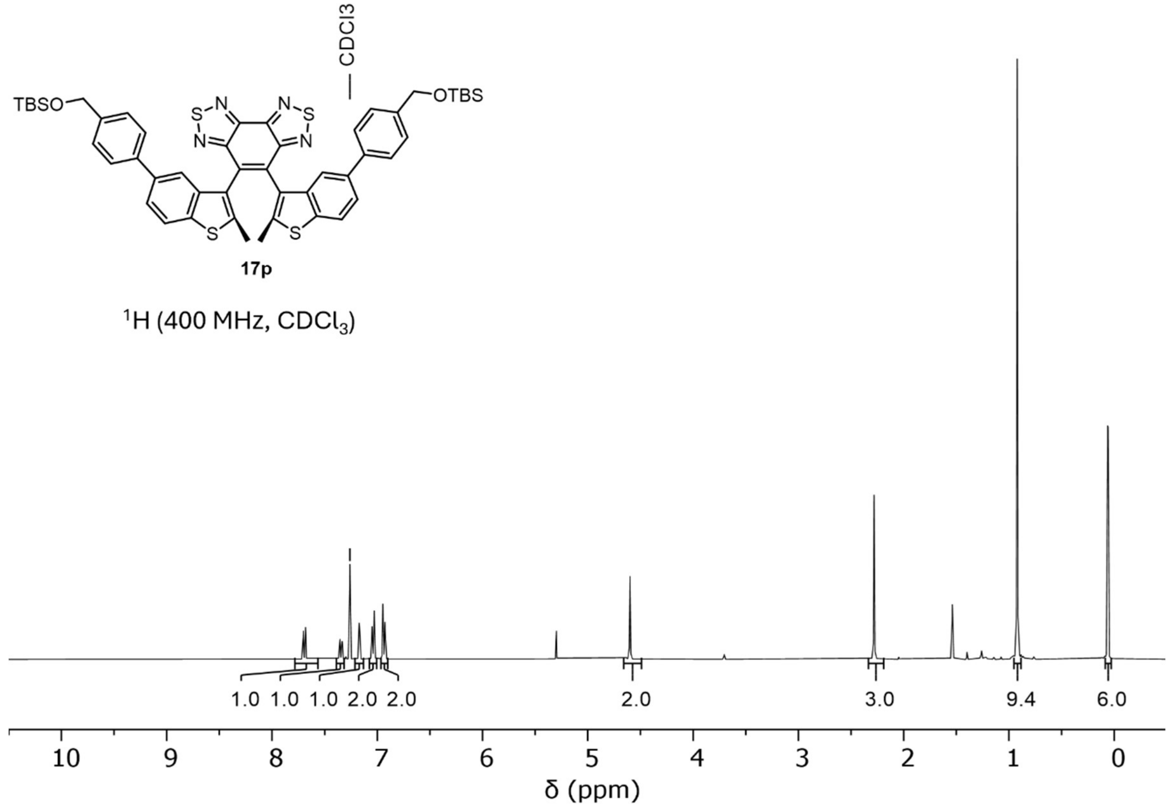
TBSO
OTBS
CDCl3
17p
1H (400 MHz, CDCl3)
1.0 1.0 1.0 2.0 2.0
2.0
3.0
9.4
6.0
10 9 8 7 6 5 4 3 2 1 0
δ (ppm)

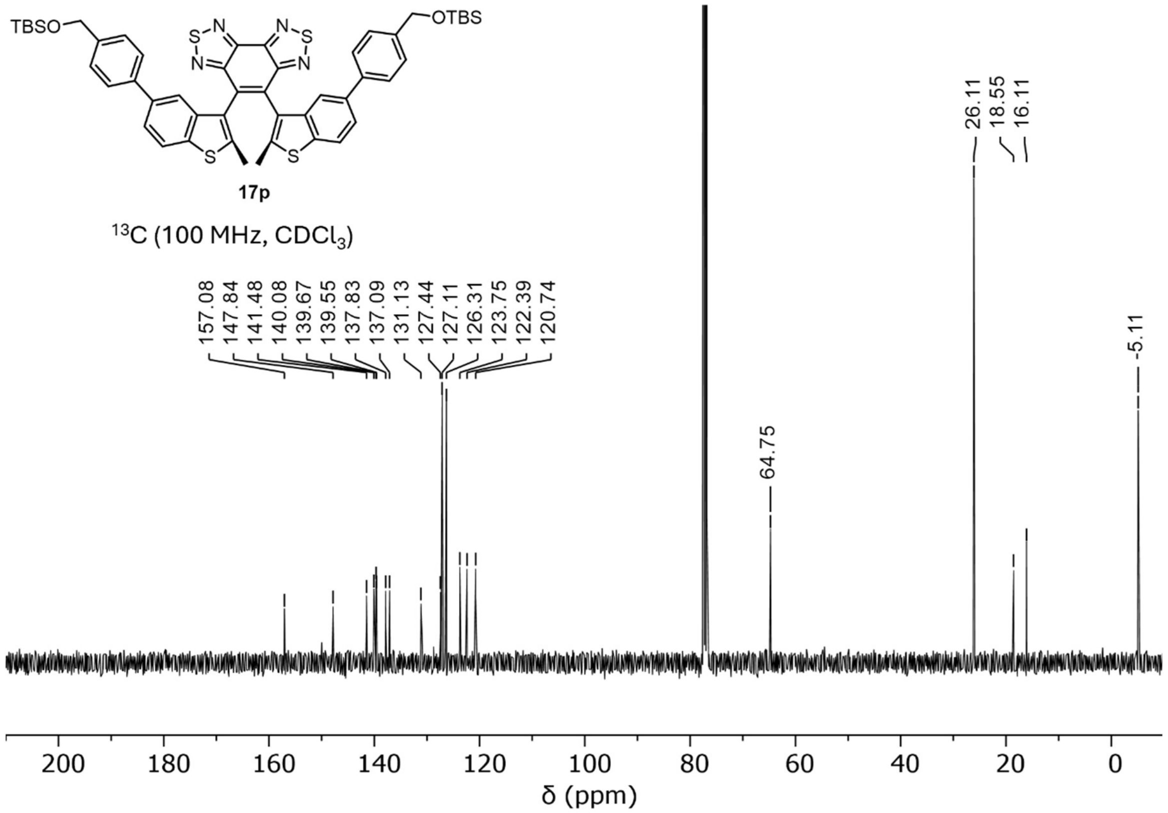
TBSO
OTBS
17p
13C (100 MHz, CDCl3)
157.08
147.84
141.48
140.08
139.67
139.55
137.83
137.09
131.13
127.44
127.11
126.31
123.75
122.39
120.74
64.75
26.11
18.55
16.11
-5.11
200 180 160 140 120 100 80 60 40 20 0
δ (ppm)

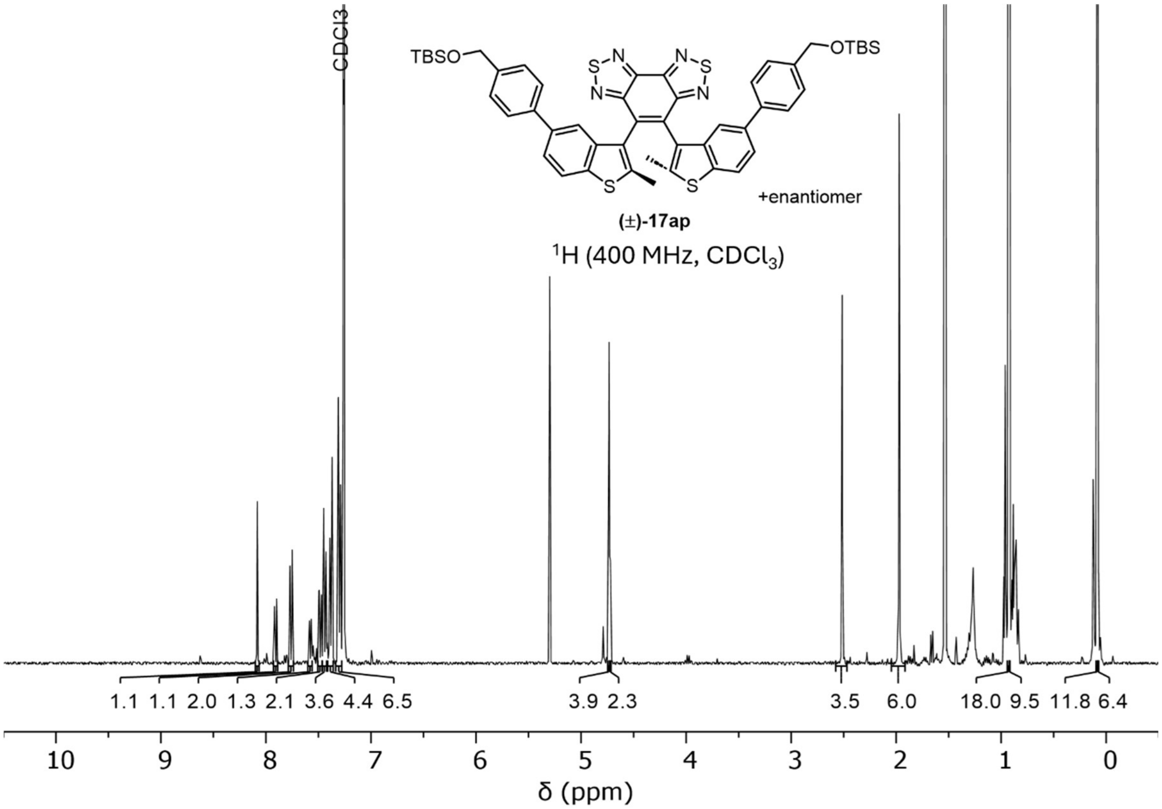


**(±)-17ap** contains impurities [*: impurity peaks]. We attribute the major impurity as the mono-substitution byproduct. $^1$H NMR (400 MHz, Chloroform-*d*) δ 8.08 (s, 1.1H)*, 7.91 (d, *J* = 8.3 Hz, 1.1H)*, 7.76 (d, *J* = 8.4 Hz, 2H), 7.58 (dd, *J* = 8.3, 1.8 Hz, 1.3H)*, 7.48 (dd, *J* = 8.4, 1.7 Hz, 2H), 7.45 (s, 2H), 7.43 (s, 1.1 H)*, 7.38 (d, *J* = 8.2 Hz, 4H), 7.30 (d, *J* = 8.0 Hz, 4H), 7.30 (d, *J* = 8.0 Hz, 1.3H)*, 4.73 (s, 4H), 4.72 (s, 2.3H)*, 2.51 (s, 3.5H)*, 1.97 (s, 6H), 0.93 (s, 18H), 0.92 (s, 9.5H)*, 0.09 (s, 12H), 0.08 (s, 6.4H)*.

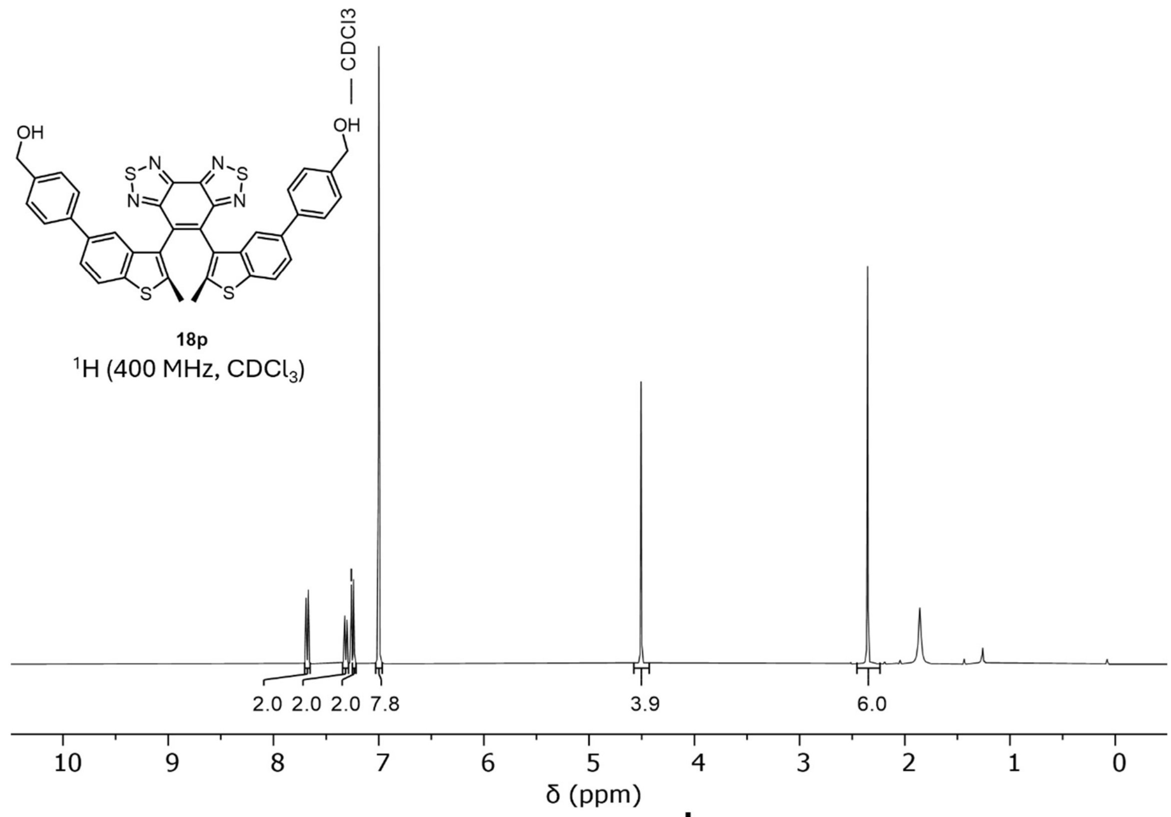
CDCl3
OH
OH
18p
$^{1}$H (400 MHz, CDCl$_3$)
2.0 2.0 2.0 7.8
3.9
6.0
10 9 8 7 6 5 4 3 2 1 0
δ (ppm)

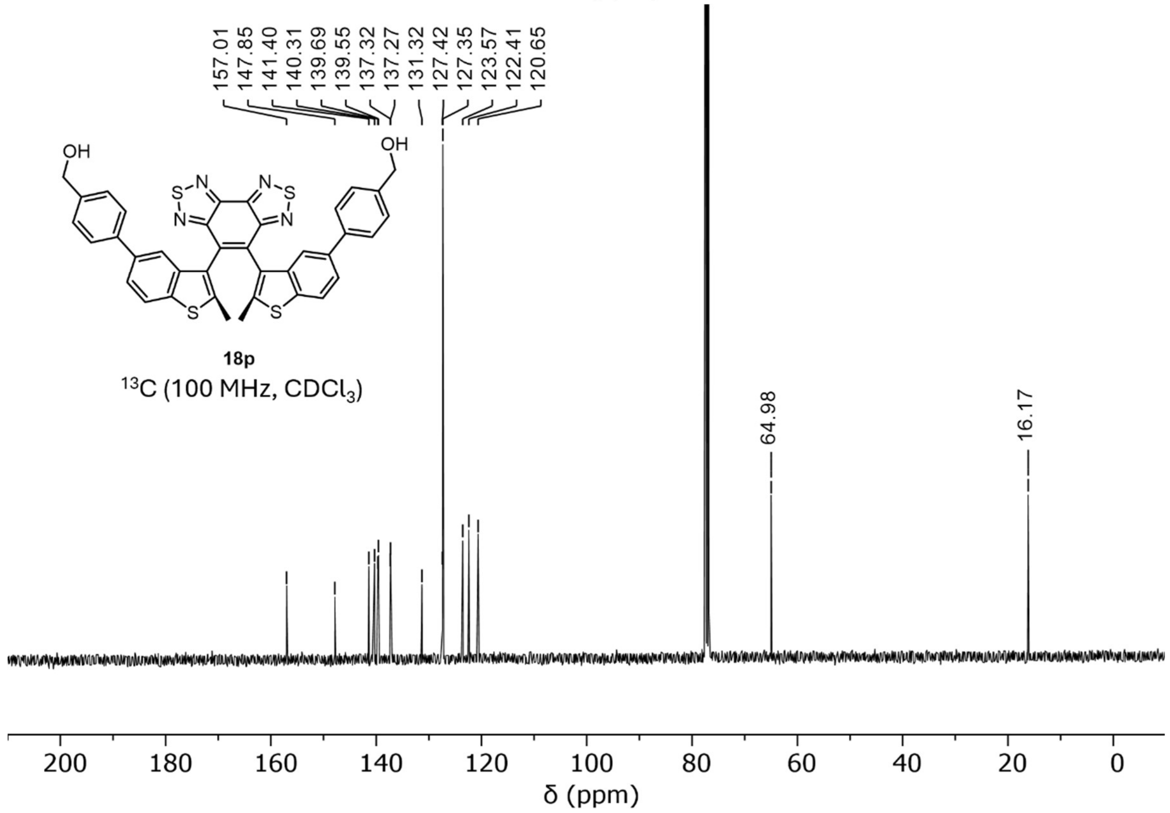
157.01
147.85
141.40
140.31
139.69
139.55
137.32
137.27
131.32
127.42
127.35
123.57
122.41
120.65
OH
OH
18p
$^{13}$C (100 MHz, CDCl$_3$)
64.98
16.17
200 180 160 140 120 100 80 60 40 20 0
δ (ppm)

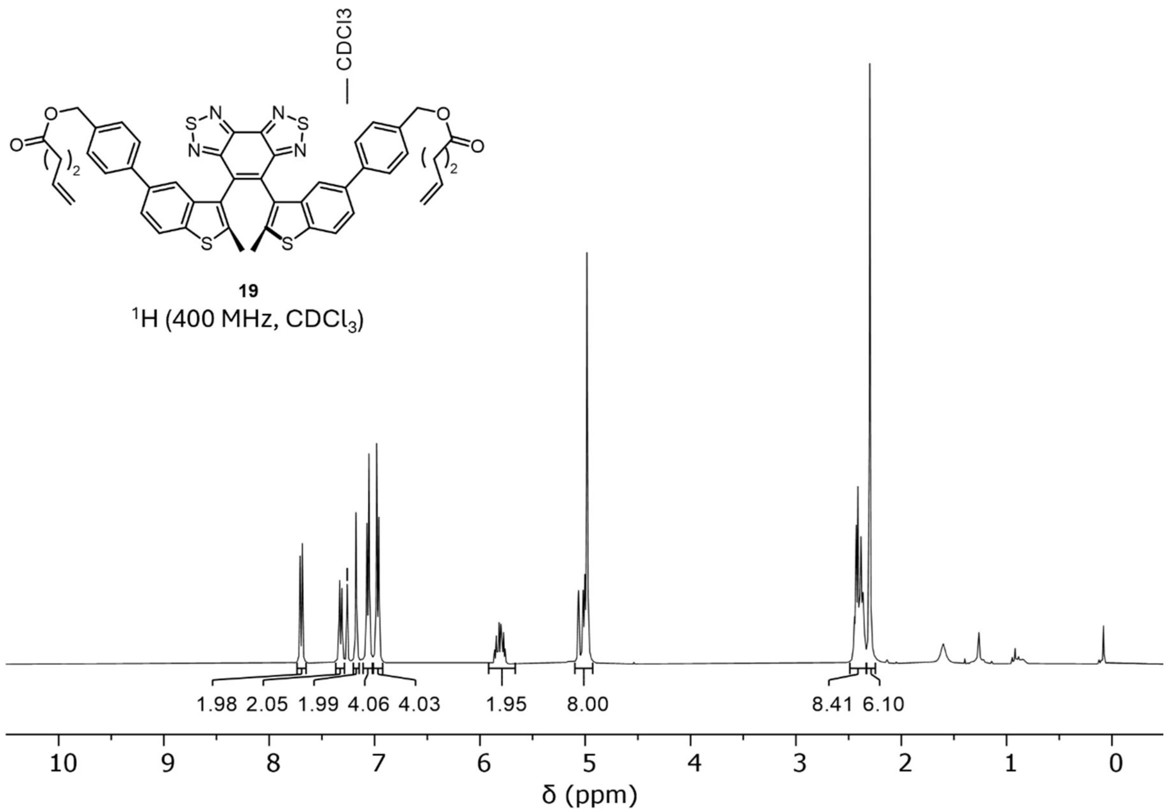
CDCl3
19
$^{1}$H (400 MHz, $CDCl_3$)
1.98 2.05 1.99 4.06 4.03
1.95
8.00
8.41 6.10
10 9 8 7 6 5 4 3 2 1 0
δ (ppm)

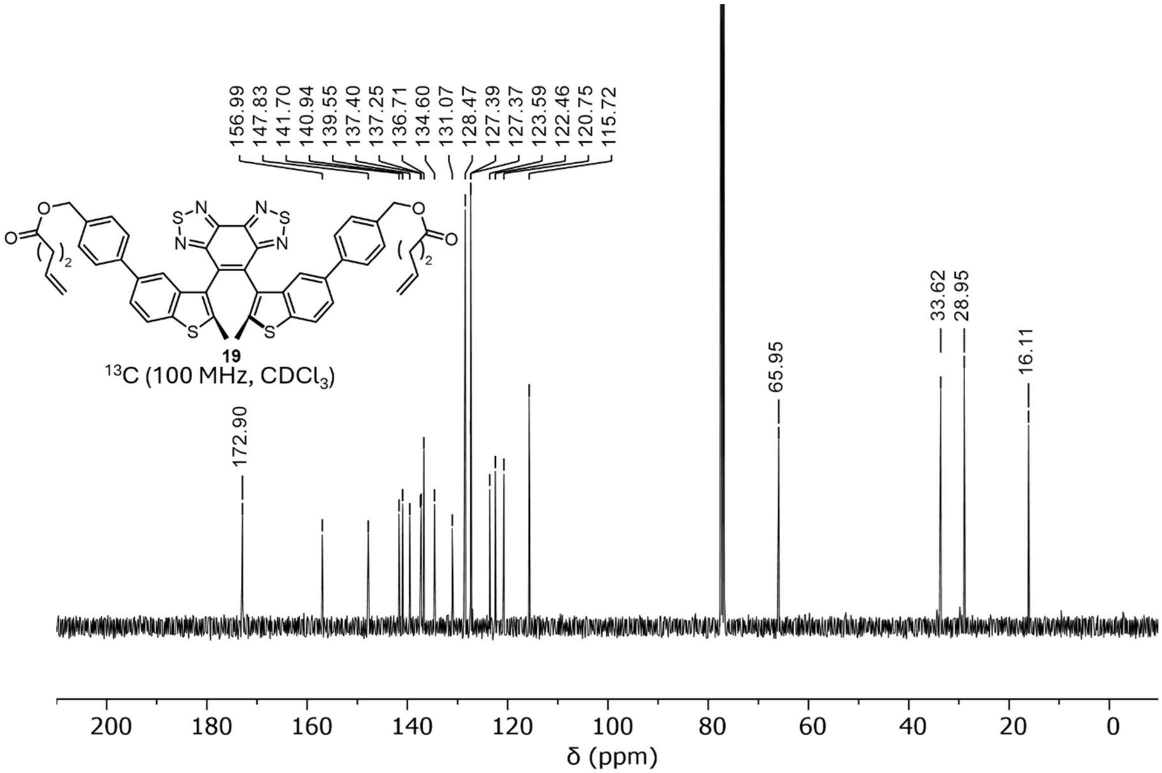
156.99
147.83
141.70
140.94
139.55
137.40
137.25
136.71
134.60
131.07
128.47
127.39
127.37
123.59
122.46
120.75
115.72
172.90
65.95
33.62
28.95
16.11
19
$^{13}$C (100 MHz, $CDCl_3$)
200 180 160 140 120 100 80 60 40 20 0
δ (ppm)

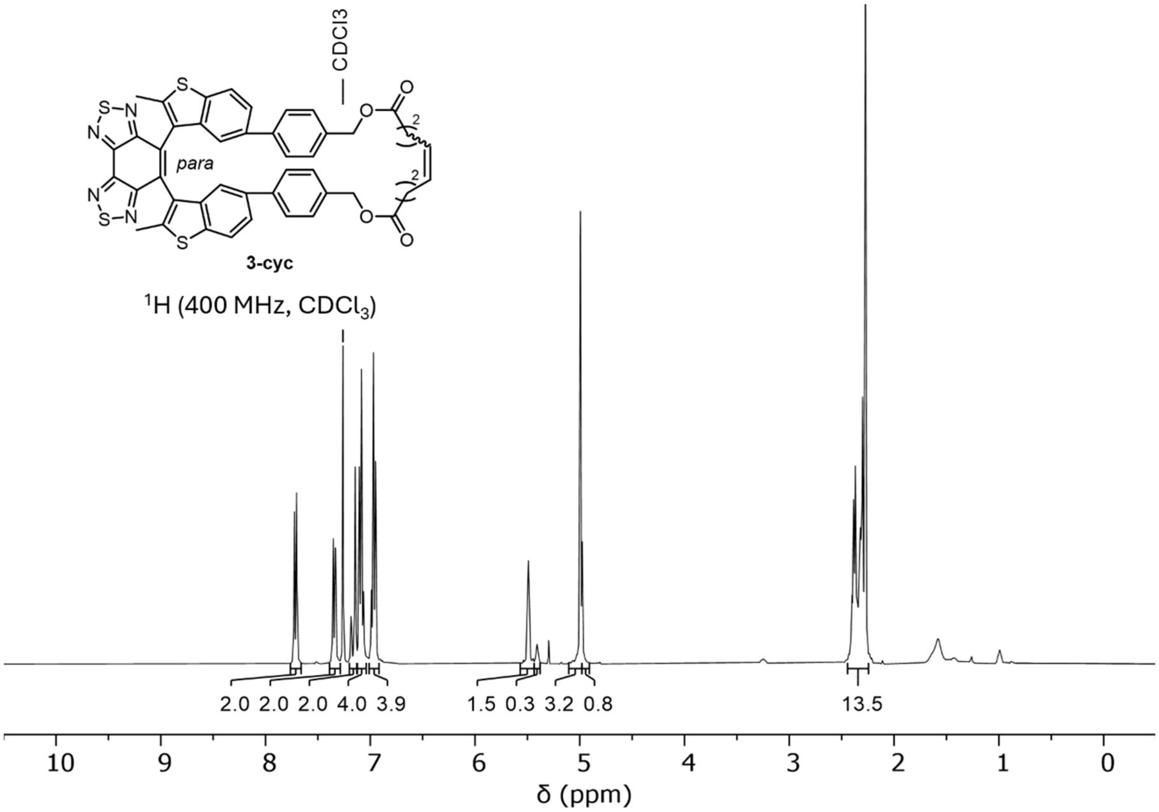
CDCl3
para
3-cyc
1H (400 MHz, CDCl3)
2.0 2.0 2.0 4.0 3.9
1.5 0.3 3.2 0.8
13.5
10 9 8 7 6 5 4 3 2 1 0
δ (ppm)

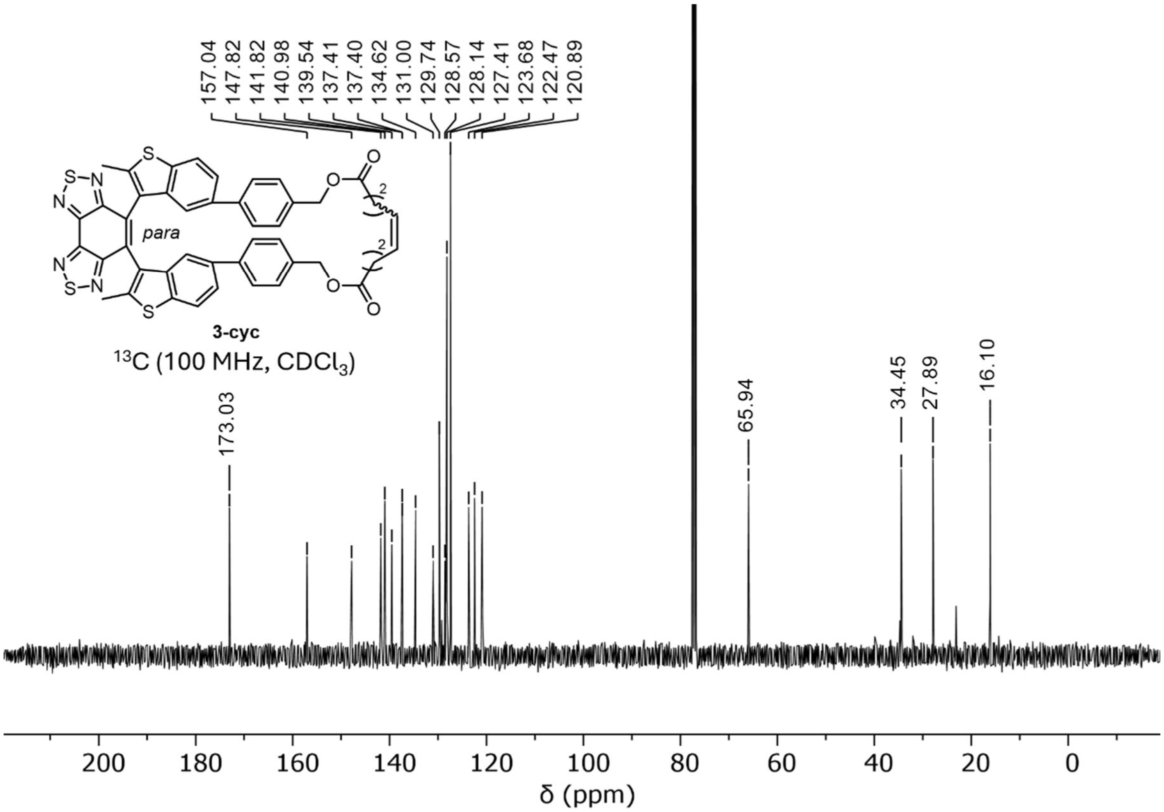
157.04
147.82
141.82
140.98
139.54
137.41
137.40
134.62
131.00
129.74
128.57
128.14
127.41
123.68
122.47
120.89
para
3-cyc
13C (100 MHz, CDCl3)
173.03
65.94
34.45
27.89
16.10
200 180 160 140 120 100 80 60 40 20 0
δ (ppm)

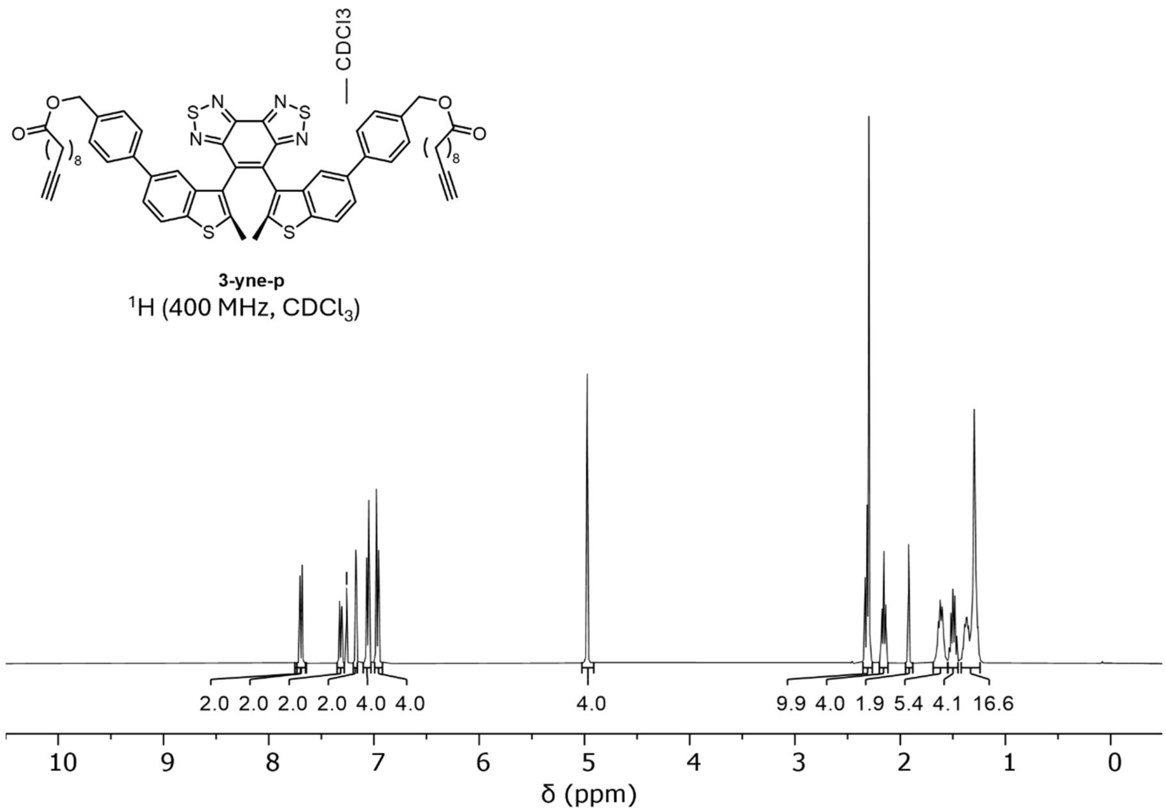
CDCl3
3-yne-p
$^{1}$H (400 MHz, CDCl$_3$)
2.0 2.0 2.0 2.0 4.0 4.0
4.0
9.9 4.0 1.9 5.4 4.1 16.6
10 9 8 7 6 5 4 3 2 1 0
δ (ppm)

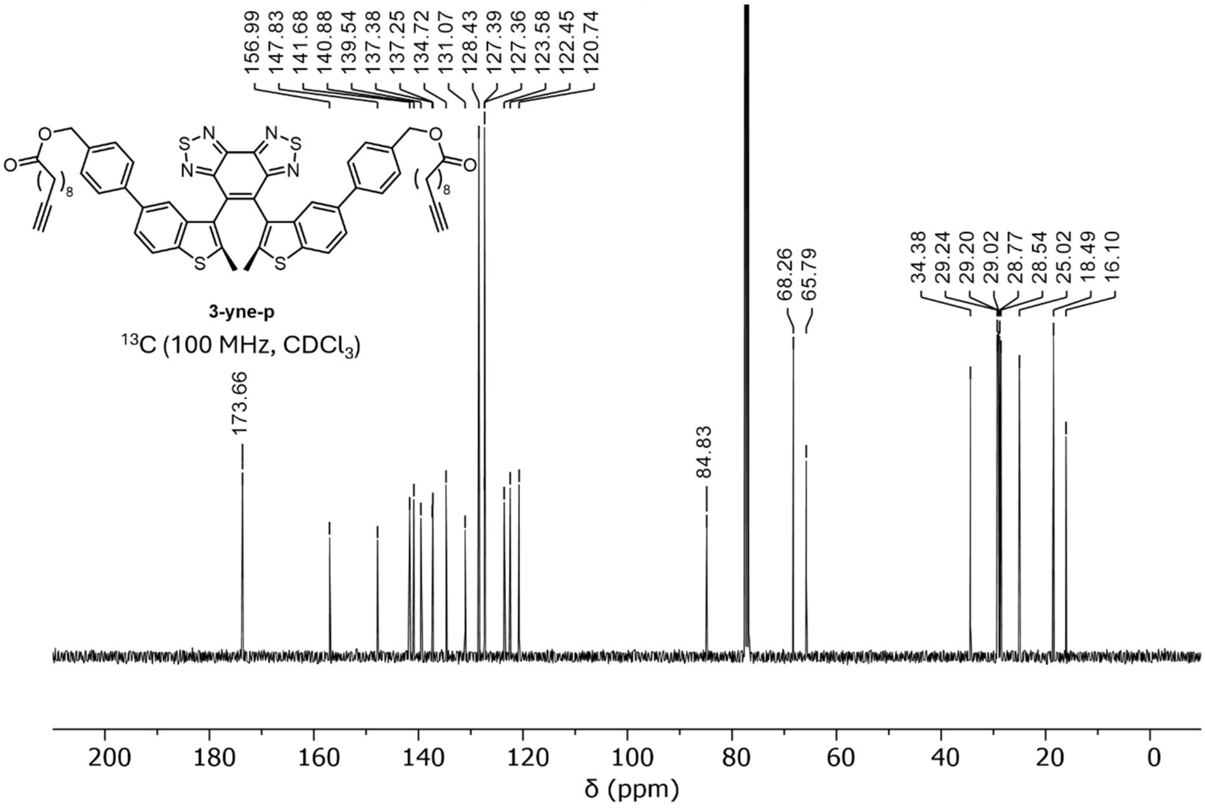
156.99 147.83 141.68 140.88 139.54 137.38 137.25 134.72 131.07 128.43 127.39 127.36 123.58 122.45 120.74
3-yne-p
$^{13}$C (100 MHz, CDCl$_3$)
173.66
84.83
68.26 65.79
34.38 29.24 29.20 29.02 28.77 28.54 25.02 18.49 16.10
200 180 160 140 120 100 80 60 40 20 0
δ (ppm)

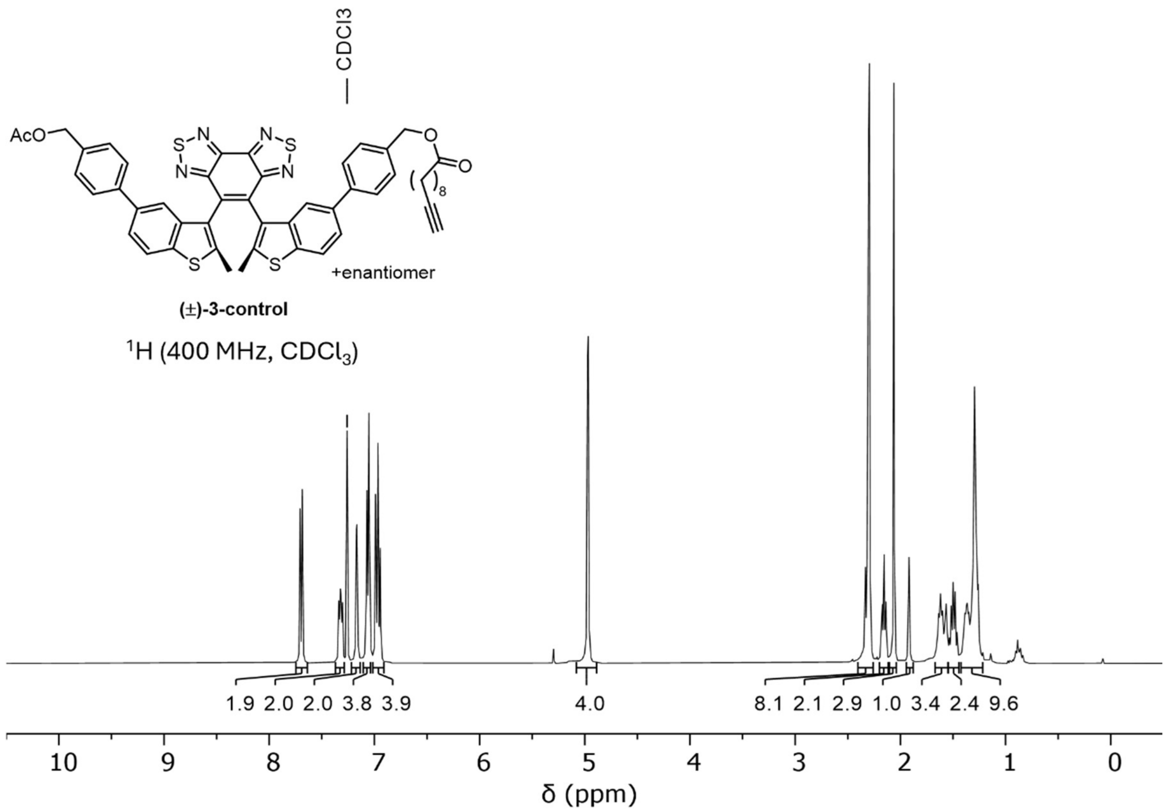
CDCl3
AcO
(±)-3-control
+enantiomer
1H (400 MHz, CDCl3)
1.9 2.0 2.0 3.8 3.9
4.0
8.1 2.1 2.9 1.0 3.4 2.4 9.6
δ (ppm)

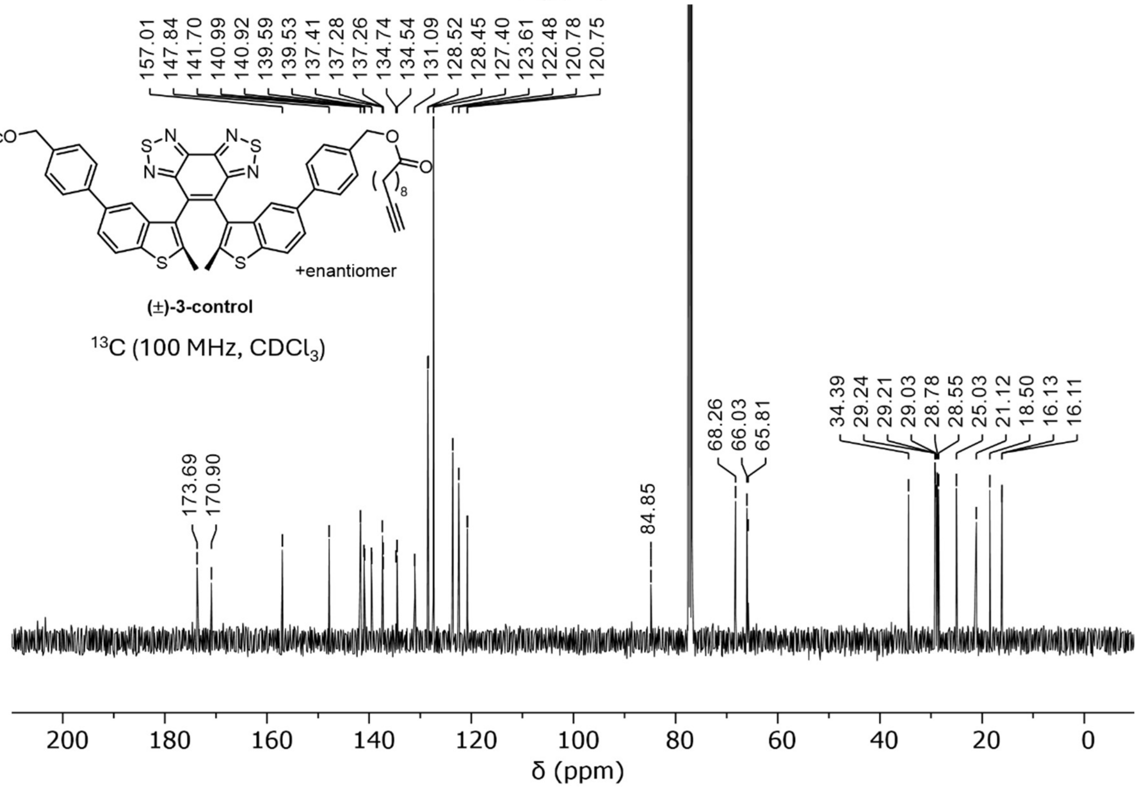
157.01 147.84 141.70 140.99 140.92 139.59 139.53 137.41 137.28 137.26 134.74 134.54 131.09 128.52 128.45 127.40 123.61 122.48 120.78 120.75
AcO
(±)-3-control
+enantiomer
13C (100 MHz, CDCl3)
173.69 170.90
84.85
68.26 66.03 65.81
34.39 29.24 29.21 29.03 28.78 28.55 25.03 21.12 18.50 16.13 16.11
δ (ppm)

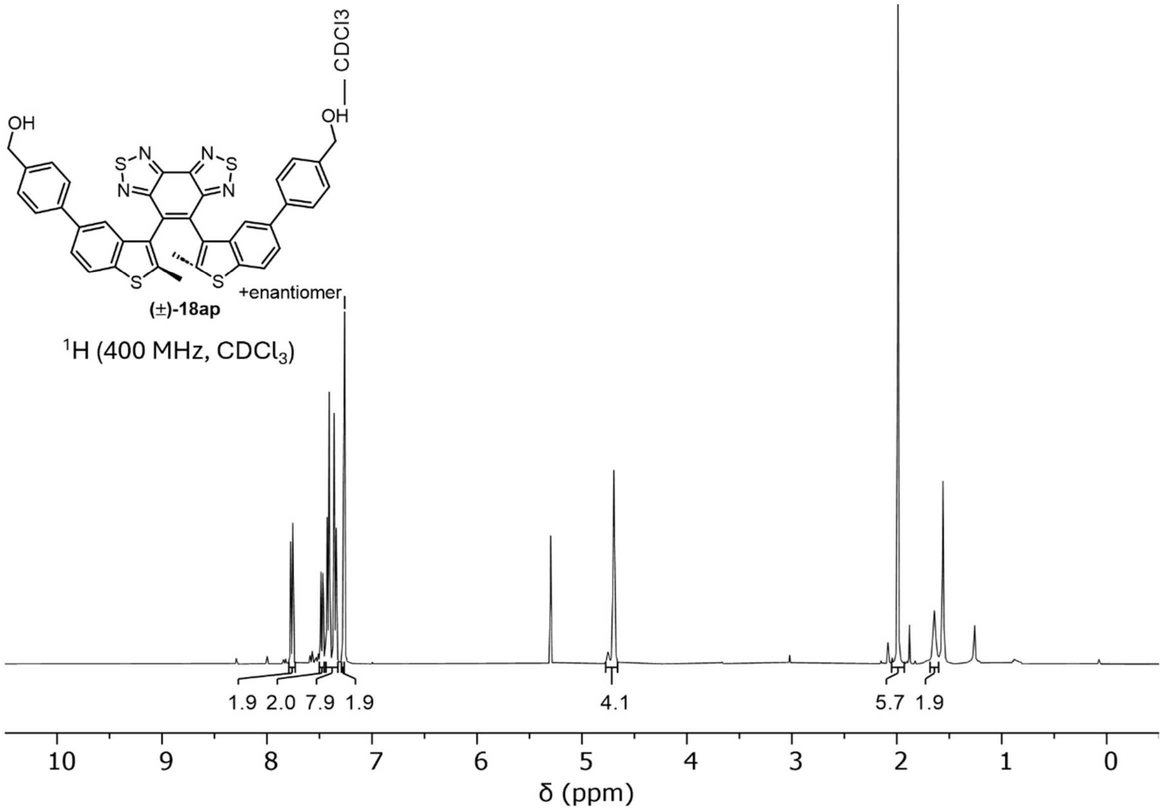
CDCl3
OH
OH
S-N
N-S
N
N
S
S
+enantiomer
(±)-18ap
$^{1}H$ (400 MHz, $CDCl_3$)
1.9 2.0 7.9 1.9
4.1
5.7 1.9
10 9 8 7 6 5 4 3 2 1 0
δ (ppm)

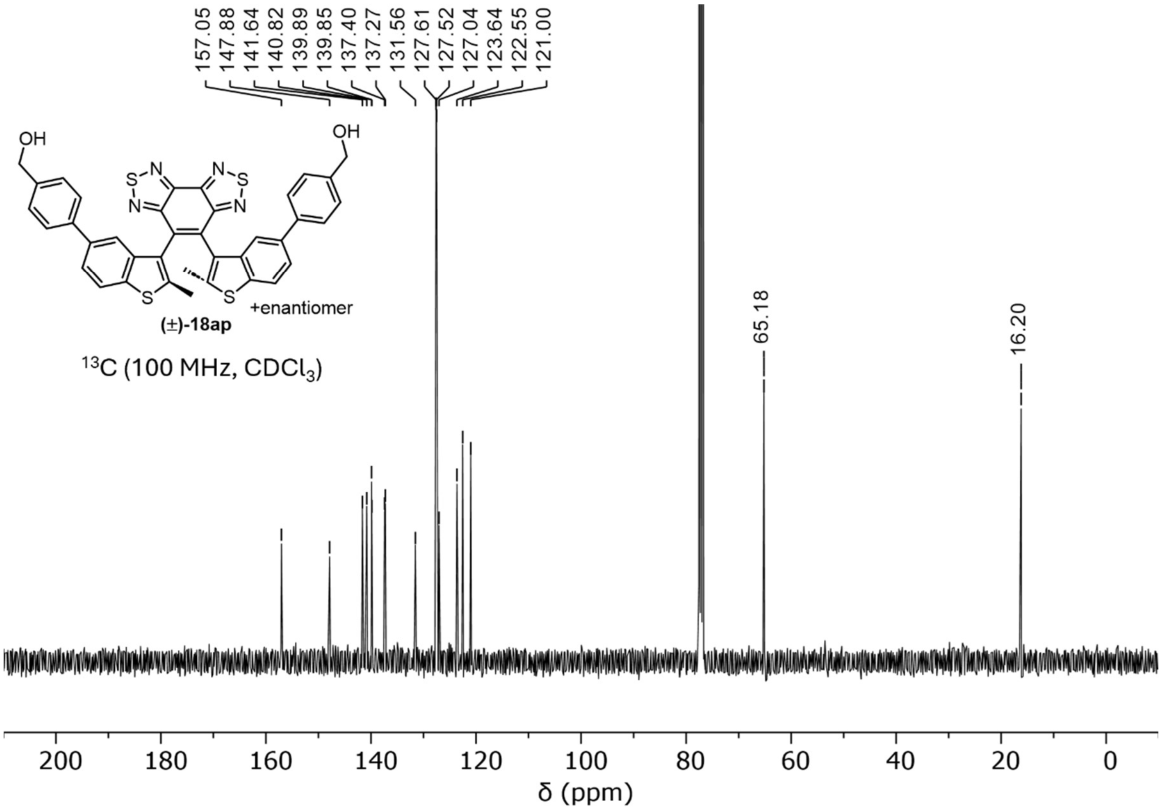
157.05
147.88
141.64
140.82
139.89
139.85
137.40
137.27
131.56
127.61
127.52
127.04
123.64
122.55
121.00
65.18
16.20
OH
OH
S-N
N-S
N
N
S
S
+enantiomer
(±)-18ap
$^{13}C$ (100 MHz, $CDCl_3$)
200 180 160 140 120 100 80 60 40 20 0
δ (ppm)

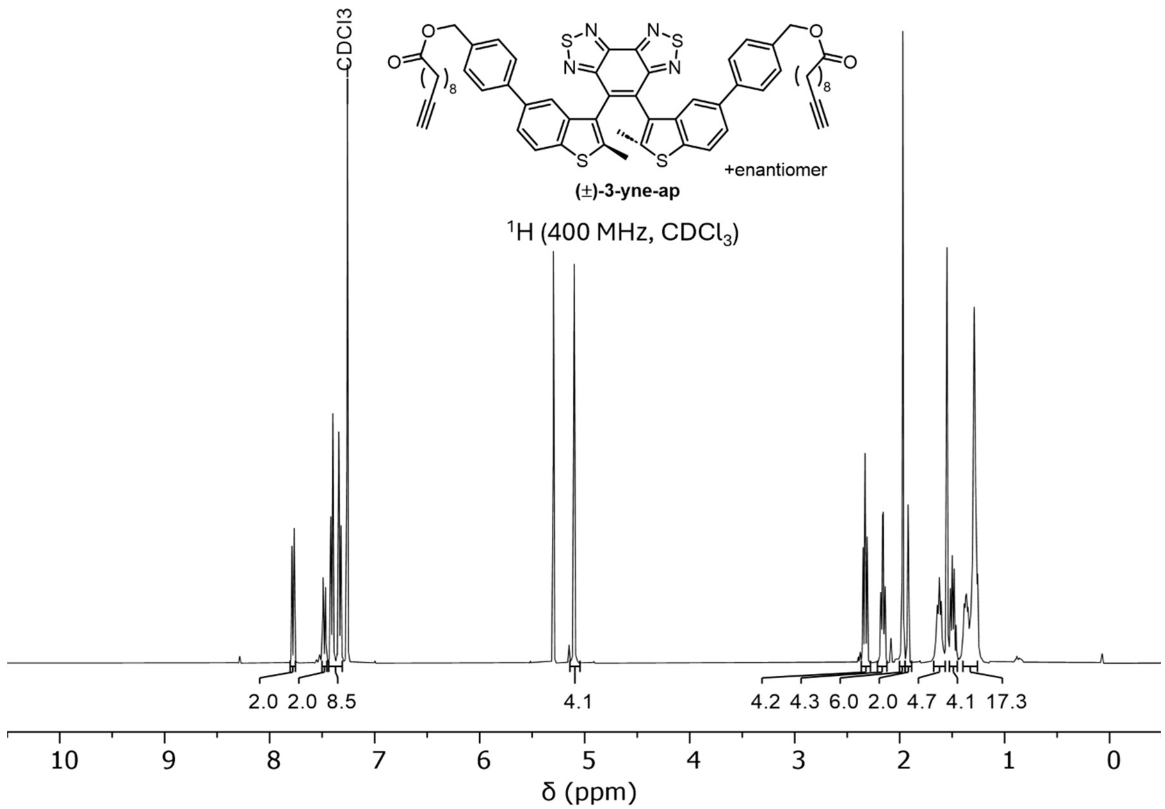
CDCl3
+enantiomer
(±)-3-yne-ap
1H (400 MHz, CDCl3)
2.0 2.0 8.5
4.1
4.2 4.3 6.0 2.0 4.7 4.1 17.3
10 9 8 7 6 5 4 3 2 1 0
δ (ppm)

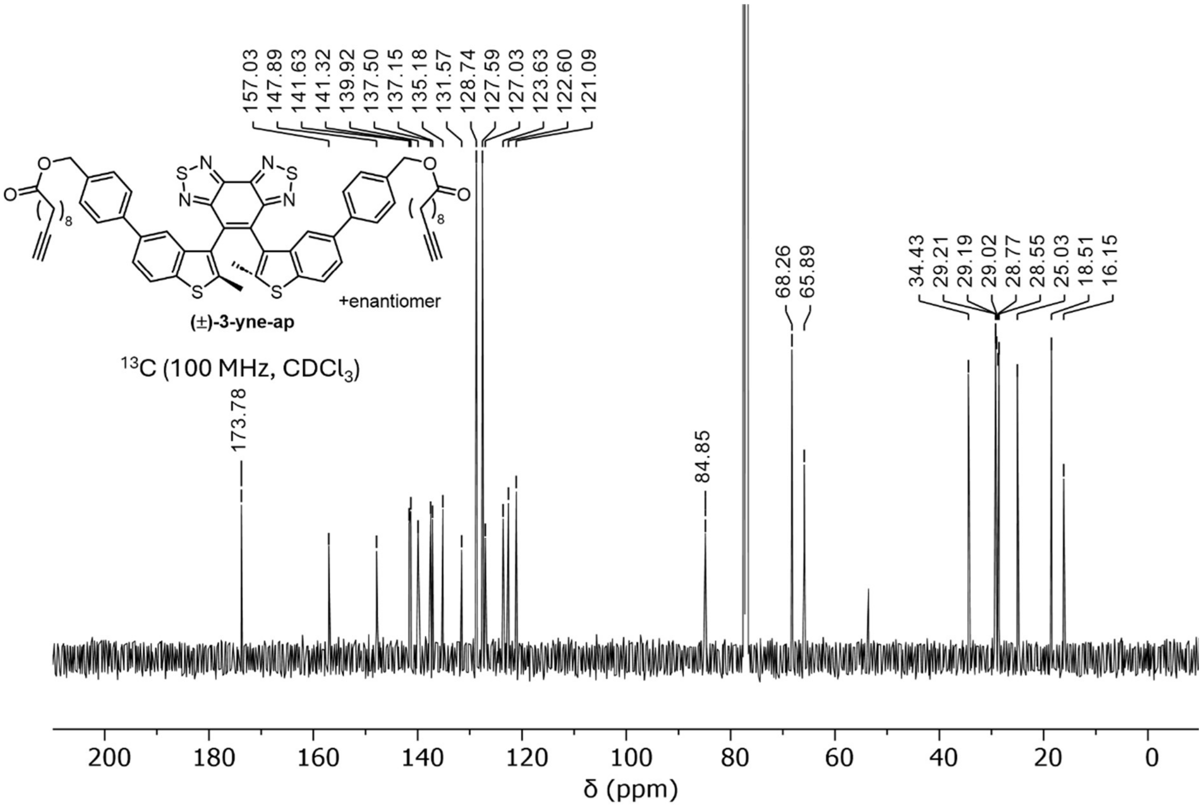
157.03 147.89 141.63 141.32 139.92 137.50 137.15 135.18 131.57 128.74 127.59 127.03 123.63 122.60 121.09
+enantiomer
(±)-3-yne-ap
13C (100 MHz, CDCl3)
173.78
84.85
68.26 65.89
34.43 29.21 29.19 29.02 28.77 28.55 25.03 18.51 16.15
200 180 160 140 120 100 80 60 40 20 0
δ (ppm)
